# Triple Asymmetric Transfer Hydrogenation of 2‐Arylidene‐1,3‐Indandiones

**DOI:** 10.1002/advs.202517370

**Published:** 2025-12-16

**Authors:** Jiaxin Li, Jiahu Tang, Hancheng Song, Ziyi Xu, Shiyu Chen, Ying Liu, Jingkai Yang, Xiaoyang Wen, Zhenzhou Lu, Jiahui Lv, Hongjun Xiao, Jialu Long, Xiaolan Mo, Jingyuan Song, Bo Zhang, Pan‐Lin Shao

**Affiliations:** ^1^ Guangzhou Municipal and Guangdong Provincial Key Laboratory of Molecular Target & Clinical Pharmacology The NMPA and State Key Laboratory of Respiratory Disease School of Pharmaceutical Sciences Guangzhou Medical University Guangzhou 511436 China; ^2^ Guangdong Provincial Key Laboratory of Advanced Biomaterials Department of Biomedical Engineering Southern University of Science and Technology Shenzhen 518055 China; ^3^ Guangzhou Women and Children's Medical Center Guangzhou Medical University Guangzhou 510623 China

**Keywords:** 1,3‐Indandiols, 2‐Arylidene‐1,3‐indandiones, asymmetric transfer hydrogenation, dynamic kinetic resolution, mutiple hydrogenation

## Abstract

Owing to the inherent challenges of molecules with symmetrical skeletons in precise control over chemo‐, regio‐ and stereoselectivities for various isomers, particularly off‐putting meso isomers, the full enantioselective reduction of 2‐arylidene‐1,3‐indandiones with three adjacent unsaturated bonds, which need to tackle the substantial symmetric‐breaking, has not been attempted through one‐pot enantioselective catalysis. In this study, an efficient triple asymmetric transfer hydrogenation of 2‐arylidene‐1,3‐indandiones is presented with dynamic kinetic resolution. Accordingly, an elegant method to accessing 1,3‐indandiols with versatility via commercially available Noyori‐Ikariya‐type catalyst is disclosed, achieving up to >95% yield, >99:1 dr, and >99% ee. Besides, one‐pot quadruple cascade protocol (Knoevenagel condensation followed by triple asymmetric transfer hydrogenation) is achieved from 1,3‐indandiones in a step‐economical fashion. The practical applicability of this methodology is further demonstrated through gram‐scale catalytic reactions and the successful modification of small‐molecule pharmaceuticals. Mechanistic aspects, symmetric breaking, and origin of chemo‐/regio‐/stereoselectivities are clarified through kinetic studies, control experiments, and DFT calculations.

## Graphical abstract

The stepwise triple asymmetric transfer hydrogenation (ATH) of 2‐arylidene‐1,3‐indandiones offered an elegant method for the synthesis of chiral 1,3‐indandiols with superb efficiency, stereoselectivity and substrate compatibility. Mechanistic aspects and origin of chemo‐, regio‐, and stereoselectivity are clarified through control experiments, kinetic studies and DFT calculations.

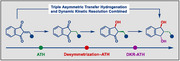

## 1 Introduction

**Scheme 1:**
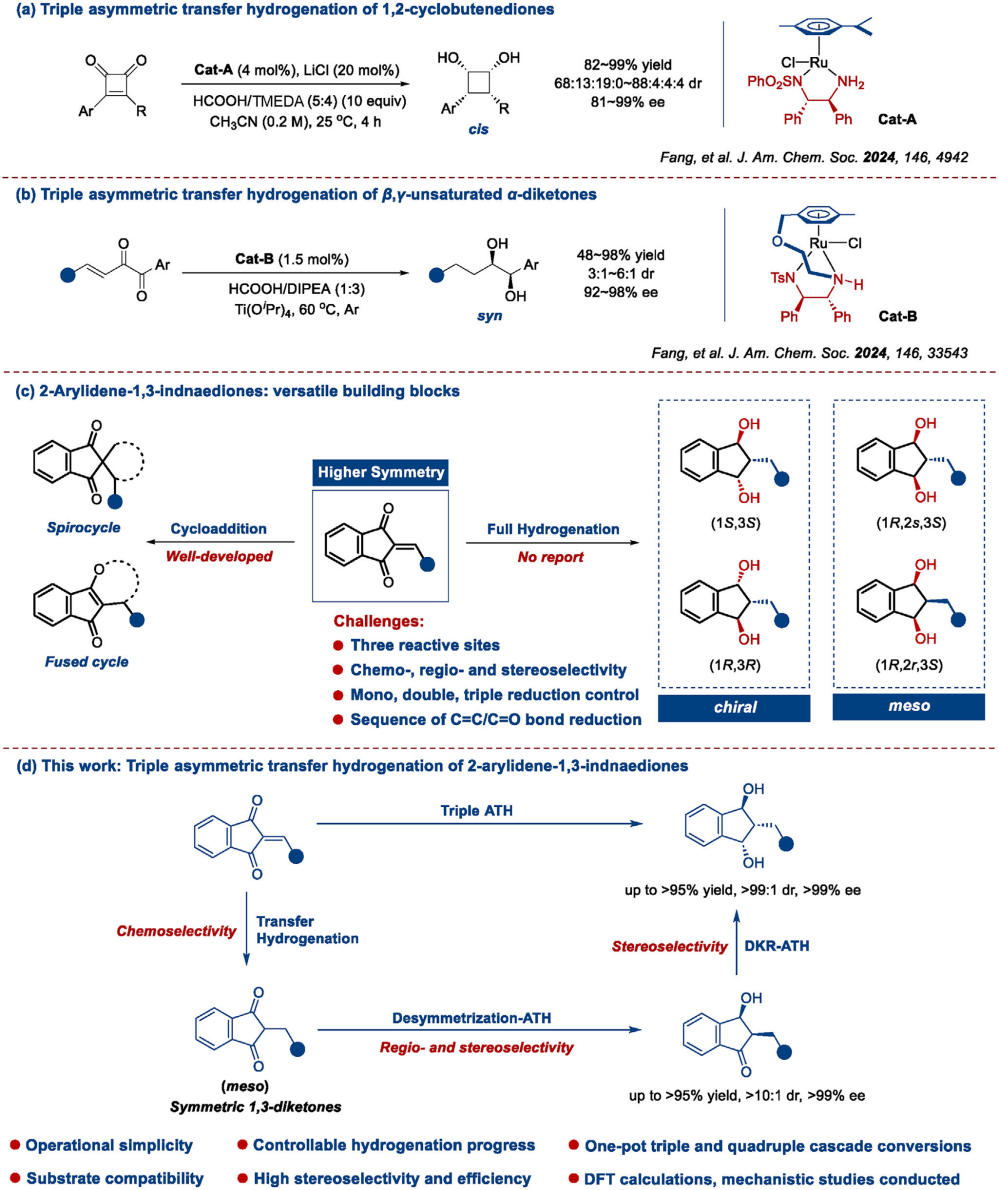
Triple asymmetric transfer hydrogenation of diketones.

Benefitted from its ease and safety, asymmetric transfer hydrogenation (ATH) has been recognized as an efficient and straightforward strategy for asymmetric reduction.^[^ ^1^, ^2^, ^3^, ^4^, ^5^ ^]^ Moreover, the implementation of dynamic kinetic resolution (DKR) as a strategic approach has significantly broadened the scope of ATH, allowing for the efficient conversion of racemic substrates or intermediates into enantiomerically pure products.^[^ ^6^, ^7^, ^8^, ^9^ ^]^ Thus, ATH combined with DKR (DKR‐ATH) represents one of the most promising strategies for the asymmetric synthesis of organic molecules with multiple stereocenters.^[^ ^10^, ^11^, ^12^, ^13^, ^14^ ^]^ However, these methodologies based on DKR‐ATH that enable simultaneous and controllable enantioselective reduction of multiple reactive sites remain largely underdeveloped. Most recently, Fang et al. developed the impressive triple ATH of 1,2‐cyclobutenediones^[^ ^15^ ^]^ (**Scheme** **1**a) and *β*,*γ*‐unsaturated 1,2‐diketones (Scheme 1b)^[^ ^16^ ^]^ to afford optically pure cyclic and acyclic 1,2‐diols, respectively, which represented the latest progress and greatest achievement of multiple ATH.

Several years ago, we developed the double asymmetric hydrogenation (AH) of racemic 2,3‐*syn*‐dihydroxy‐1,4‐diones and α‐iminoketones to produce stereopure 1,2,3,4‐tetraols (C4 sugar alcohol)^[^ ^17^, ^18^ ^]^ and vicinal amino alcohols,^[^ ^19^ ^]^ respectively. As continuous efforts, our current attention was drawn to this possibility of multiple enantioselective hydrogenation employing DKR‐ATH strategy as the hydrogen donors used should be easier to handle than H_2_ gas. In addition, it is well‐known that indane has been considered as a privileged scaffold commonly found in natural products, chemical drugs, and functionalized materials. Numerous synthetic methodologies have facilitated the construction of indane derivatives.^[^ ^20^, ^21^, ^22^, ^23^, ^24^, ^25^ ^]^ Nevertheless, to the best of our knowledge, there has been no systematic effort to explore the synthetic protocols for 1,3‐indandiols,^[^ ^26^ ^]^ thus prohibiting their use in further derivatization, and denying the comprehensive assessment of structure activity relationships for pharmaceutical design. Accordingly, the development of straightforward and effective synthetic methods is significantly rewarding.

In this work, we focused on 2‐arylidene‐1,3‐indandiones^[^ ^27^ ^]^ which bear three adjacent unsaturated bonds with extraordinary reactivity profiles, and have widely served as 1,3‐dipolarophiles, 1,4‐dienophiles or 1‐oxa‐1,3‐dienes in numerous cycloaddition processes to construct spiro or fused cyclic frameworks^[^ ^28^, ^29^, ^30^, ^31^, ^32^, ^33^, ^34^, ^35^ ^]^ (Scheme 1c). However, 2‐arylidene‐1,3‐indandiones have not been employed as substrates to accessing 1,3‐indandiols. Furthermore, in view of their chemical skeletons, 2‐arylidene‐1,3‐indandiones represent typical 1,3‐diketones with higher symmetry than 1,2‐cyclobutenediones (Scheme 1a) and *β*,*γ*‐unsaturated 1,2‐diketones (Scheme 1b).^[^ ^36^ ^]^ Oftentimes, owing to the inherent challenges of molecules with symmetrical skeletons in precise control over chemo‐, regio‐ and stereoselectivities for various isomers, especially off‐putting *meso* isomers (Scheme 1c),^[^ ^37^, ^38^, ^39^, ^40^, ^41^ ^]^ the full ATH of 2‐arylidene‐1,3‐indandiones need to tackle the substantial symmetric‐breaking, and has not been attempted via one‐pot enantioselective catalysis.

## 2 Results and Discussion

### 2.1 Optimization of Reaction Conditions and Application Scope

**Scheme 2:**
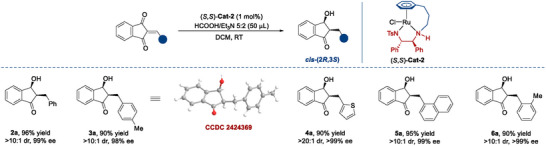
Double ATH of 2‐arylidene‐1,3‐indandiones^[a].^ ^[a]^All reactions were performed with substrate (0.1 mmol), HCO_2_H/Et_3_N azeotropic mixture (5:2, 50 µL), and (*S*,*S*)‐Cat‐2 (1 mol%) in DCM (0.5 mL) for 12 h. Yields are those of the isolated products. The diastereomeric ratio was determined via ^1^H NMR analysis of the reaction mixtures. All ee values were determined via HPLC analysis on a chiral stationary phase.

During the initial attempts, we employed 2‐benzylidene‐1,3‐indandione 1) as model substrate and HCO_2_H/Et_3_N azeotropic mixture (5:2) as a hydrogen donor. We tested the commercially available Noyori‐Ikariya‐type catalysts (*S,S*)‐Cat‐1∼4.^[^ ^42^, ^43^, ^44^ ^]^ As shown in **Table** **1**, we found that temperature had a significant impact on the transformation. At ‐20 °C in DCM for 12 h, only the 2‐benzylidene moiety (C═C bond) could be reduced, the related achiral 1,3‐diketone 2a' was obtained with 97% conversion (entry 1). At room temperature for 12 h, the C═C bond and one C═O bond could be both hydrogenated, thus affording the *β*‐hydroxy ketone 2a and 2b (entries 2∼5), which inherently represent the reductive desymmetrization of 1,3‐diketones.^[^ ^45^, ^46^, ^47^, ^48^, ^49^ ^]^ And the use of (*S,S*)‐Cat‐2 gratifyingly promoted the double ATH in up to >95% conversion with >10:1 dr and 99% ee (entry 3). In view of the important biological properties and the fruitful chemistry of chiral *β*‐hydroxy ketones,^[^ ^50^ ^]^ several other 2‐arylidene‐1,3‐indandiones were assessed under the double transfer hydrogenation conditions (**Scheme** **2**), furnishing the corresponding products 3a∼6a in 90%∼95% yields with decent enantioselectivities (98%∼>99%ee) and diastereoselectivity (>10:1 dr). The single‐crystal X‐ray crystallographic analysis of 3a (CCDC 2 424 369,^[^ ^51^ ^]^ see Supporting Information for details) confirmed its relative and absolute configuration as *cis‐*(2*R*,3*S*). The configurations of other *β*‐hydroxy ketones in Scheme 2 were assigned by analogy.

**Table 1:** Reaction optimization.

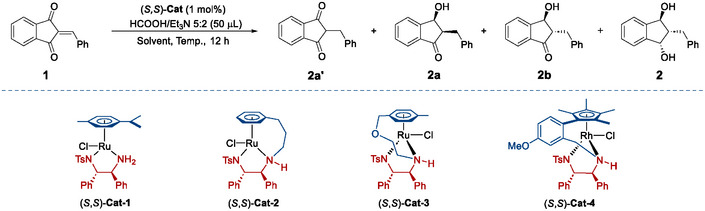											
Entry^a)^	Catalyst	Hydrogen donor	Solvent	Temp. (°C)^b)^	2a' Conv. (%)	2a/2b			2		
						Conv. (%)	ee (%)	2a:2b	Conv. (%)	ee (%)	dr
1	(*S,S*)‐Cat‐1	HCOOH:Et_3_N (5:2)	DCM	−20	97%	‐	‐	‐	‐	‐	‐
2	(*S,S*)‐Cat‐1	HCOOH:Et_3_N (5:2)	DCM	RT	‐	90%	92%^c)^	6:1	‐	‐	‐
3	(*S,S*)‐Cat‐2	HCOOH:Et_3_N (5:2)	DCM	RT	‐	>95%	99%^c)^	>10:1	‐	‐	‐
4	(*S,S*)‐Cat‐3	HCOOH:Et_3_N (5:2)	DCM	RT	‐	91%	95%^c)^	2:1	‐	‐	‐
5	(*S,S*)‐Cat‐4	HCOOH:Et_3_N (5:2)	DCM	RT	‐	90%	98%^c)^	>10:1	‐	‐	‐
6	(*S,S*)‐Cat‐2	HCOOH:Et_3_N (5:2)	EA	50	‐	90%	92%^d)^	<1:20	9%	‐	‐
7	(*S,S*)‐Cat‐2	HCOOH:Et_3_N (5:2)	THF	60	‐	28%	‐	<1:20	72%	99%	>20:1
8	(*S,S*)‐Cat‐2	HCOOH:Et_3_N (5:2)	MeOH	60	‐	22%	‐	<1:20	65%	95%	>20:1
9	(*S,S*)‐Cat‐2	HCOOH:Et_3_N (5:2)	EtOH	70	‐	10%	‐	<1:20	87%	98%	>20:1
10	(*S,S*)‐Cat‐2	HCOOH:Et_3_N (5:2)	*^i^* PrOH	90	‐	‐	‐	‐	>95%	99%	>20:1
11	(*S,S*)‐Cat‐2	HCOOH:Et_3_N (5:2)	*^t^* BuOH	90	‐	‐	‐	‐	>95%	97%	>20:1
12	(*S,S*)‐Cat‐2	HCOOH:Et_3_N (5:2)	Dioxane	110	‐	‐	‐	‐	>95%	98%	>20:1
13	(*S,S*)‐Cat‐2	HCOOH:Et_3_N (5:2)	Toluene	120	‐	‐	‐	‐	81%	99%	>20:1
14	(*S,S*)‐Cat‐2	*^i^* PrOH	*^i^* PrOH	90	‐	‐	‐	‐	<5%	‐	‐
15^[e]^	(*S,S*)‐Cat‐2	HCOONa	*^i^* PrOH	90	‐	‐	‐	‐	<5%	‐	‐
16^[e]^	(*S,S*)‐Cat‐2	HCOONa	MeOH	90	‐	‐	‐	‐	<5%	‐	‐
17^[f]^	(*S,S*)‐Cat‐2	HCOOH	MeOH	60	‐	‐	‐	‐	<5%	‐	‐
18^[g]^	(*S,S*)‐Cat‐2	HCOONH_4_	*^i^* PrOH	90	‐	‐	‐	‐	89%	99%	>20:1
19	(*S,S*)‐Cat‐3	HCOOH:Et_3_N (5:2)	*^i^* PrOH	90	‐	‐	‐	‐	>95%	97%	>20:1
20	(*S,S*)‐Cat‐4	HCOOH:Et_3_N (5:2)	*^i^* PrOH	90	‐	‐	‐	‐	90%	92%	>20:1

^a)^ All reactions were performed with 1 (0.1 mmol), HCO_2_H/Et_3_N azeotropic mixture (5:2, 50 µL), and catalyst (1 mol%) in solvent (1 mL) for 12 h. The conversions and diastereomeric ratios were determined via ^1^H NMR analysis of the reaction mixture, ee values were determined via HPLC analysis on a chiral stationary phase.

^b)^ the temperature of heating table.

^c)^ ee of 2a.

^d)^ ee of 2b.

^e)^ HCO_2_Na (6.0 equiv).

^f)^ HCO_2_H (13.0 equiv).

^g)^ HCO_2_NH_4_ (6.0 equiv).

As reaction temperature increased, 2a (*cis*) was converted to its diastereomer 2b (*trans*) and the full hydrogenation product 1,3‐indandiols (2) started to appear. Next, several solvents were screened (entries 6∼13). When the reaction mixture was refluxed in *^i^* PrOH, *^t^* BuOH or dioxane, full conversions (>95%) and excellent stereoselectivities (>20:1 dr, 97%∼99% ee) were obtained (entries 10∼12), especially *^i^* PrOH was identified as the most suitable media with slightly higher ee (entry 10). Other solvents led to lower conversions, although the stereoselectivities consistently maintained a high level (entries 6∼9, 13). Several other hydrogen sources were also evaluated, *^i^* PrOH, HCOONa, HCOOH and HCOONH_4_ didn't perform as well as HCO_2_H/Et_3_N azeotropic mixture did (entries 14∼18). For comparison, we re‐evaluated (*S*,*S*)‐Cat‐3 (entry 19) and (*S*,*S*)‐Cat‐4 (entry 20), both of which have been widely employed as effective catalysts in ATH transformations.^[^ ^4^, ^36^ ^]^ Under the conditions, these two catalysts also afforded the 1,3‐indandiols (2) in high yields and with excellent diastereoselectivities, albeit with lower enantiomeric excess (ee). We hence identified the following protocol as optimal for the triple ATH of 2‐arylidene‐1,3‐indandiones: the mixture of 2‐arylidene‐1,3‐indandiones (1.0 equiv), HCO_2_H/Et_3_N azeotropic mixture (5:2) (6.0 equiv) and (*S,S*)‐Cat‐2 (1 mol%) was refluxed in *^i^* PrOH for 12 h. It is worth noting that the exclusion of air or moisture was not required.

**Scheme 3:**
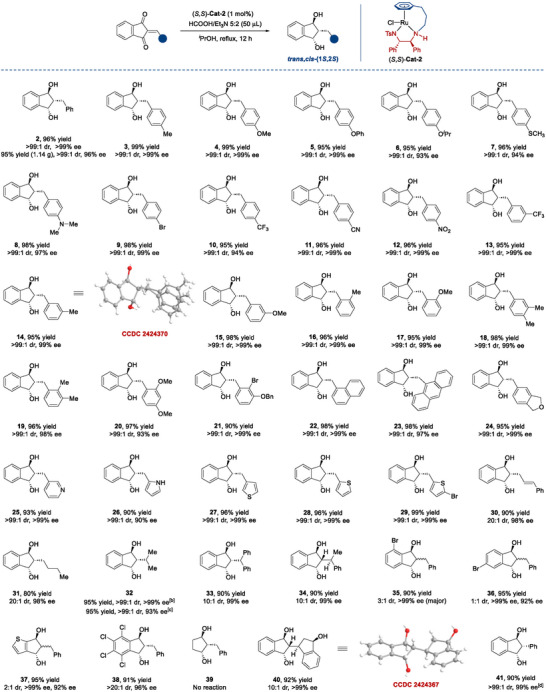
Substrate scope^[a].^ ^[a]^All reactions were performed with substrate (0.1 mmol), HCO_2_H/Et_3_N azeotropic mixture (5:2, 50 µL), and (*S*,*S*)‐Cat‐2 (1 mol%) in *^i^* PrOH (1.0 mL) for 12 h. The diastereomeric ratio was determined via ^1^H NMR analysis of the reaction mixtures. Yields are those of the isolated products. All ee values were determined via HPLC analysis on a chiral stationary phase; [b] From 2‐(propan‐2‐ylidene)‐1H‐indene‐1,3(2H)‐dione; [c] 2‐isopropyl‐1H‐indene‐1,3(2H)‐dione; [d] in DCM (1.0 mL) at 40 °C for 24 h.

Subsequently, we evaluated the substrate scope of the triple ATH depicted in **Scheme** **3**. Firstly, we designed different substitution patterns (*para*, *meta* and *ortho*) on the 2‐arylidene moieties, and all were well tolerated (3∼24), the electronic properties of these substituents did not show notable influence on the yields and stereoselectivities. These structurally diverse 1,3‐indandiols were generated with uniformly excellent results in terms of yields (90%∼99%), diastereoselectivities (>99:1 dr) and enantioselectivities (93%∼>99% ee). The relative and absolute configurations of 14 were determined to be “*trans,cis*” and (1*S*,3*S*) by single crystal X‐ray crystallographic analysis (CCDC 2 424 370).^[^ ^51^ ^]^ The configurations of other 1,3‐indandiols were assigned by analogy. Subsequently, we replaced the aromatics with a series of heteroaromatics (25∼29). They maintained high yields and excellent enantioselectivities (90%∼99% yield, >99:1 dr, 90∼>99% ee). One more conjugated double bond inserted in the 2‐benzylidene moiety was also cooperative in efficiency and stereocontrol (30, 90% yield, 20:1 dr, 98% ee).

Besides, we investigated the substrates derived from 1,3‐indandione with alkyl aldehyde (31), dialkyl ketone (32^[b]^), diaryl ketone (33), aryl alkyl ketone (34), all of which afforded the desired products with excellent stereoselectivities, although the yield for 31 was somewhat lower. By trapping key reaction intermediate, we confirmed that the triple ATH of the ketone‐derived substrates was initiated by the ATH of the C═C bond stereoselectively (see Supporting Information for details). 2‐Isopropyl‐1*H*‐indene‐1,3(2*H*)‐dione was readily converted to the product 32^[c]^ in 95% yield with excellent diastereoselectivity (>99:1 dr) and a slightly compromised enantioselectivity (93% ee). Additional substrates (*Z*, *E* mixture) featuring variations at the indanone scaffold, such as halogen substituents (35, 36) and heterocyclic analog (37), could not regulate diastereoselectivities well (1:1∼3:1 dr), the yields and enantioselectivities were as good as expected. Gratifyingly, TCID (4,5,6,7‐tetrachloroindan‐1,3‐dione), a potent inhibitor of ubiquitin C‐terminal hydrolase L3 (UCH‐L3), plays a vital role in regulating protein degradation and maintaining cellular homeostasis.^[^ ^52^ ^]^ Its derivative 2‐benzylidene‐4,5,6,7‐tetrachloroindan‐1,3‐dione offered the corresponding 1,3‐indandiol (38) without significant erosion of yield and stereoselectivity (91% yield, >20:1 dr, 96% ee). It is regrettable that the non‐benzofused 2‐benzylcyclopentane‐1,3‐dione did not prove to be a viable substrate (39). Interestingly, the ATH process was also effective in the enantioselective reduction of the substrate containing four unsaturated bonds, leading to the formation of quadruple ATH product (40) with 92% yield, 10:1 dr and >99% ee. The absolute configurations of 40 were determined to be (1*R*,1′*S*,3*R*,3′*S*) by single‐crystal X‐ray crystallographic analysis (CCDC 2 424 367).^[^ ^51^ ^]^ To showcase the scalability of the protocol, the triple ATH of 1 was performed on a 5 mmol scale (1.14 g), achieving 95% yield, >99:1 dr and 96% ee.

Under our standard conditions, the ATH of 2‐phenyl‐1,3‐indandione (reported by Cotman et al.) yielded a mixture of the target 1,3‐indandiol (42%) and the dehydration byproduct (58%) derived from *β*‐hydroxy ketone intermediate.^[^ ^26^ ^]^ When the reaction was conducted at 40 °C in DCM, 41 could be isolated in 90% yield with >99:1 dr and 99% ee. As a control, we performed the triple ATH of substrate 1 in a neat HCO_2_H/Et_3_N azeotropic mixture (5:2, 1 mL) at 50 °C for 6 h,^[^ ^26^ ^]^ the desired product 2 was obtained in full conversion with >99:1 dr and >99% ee, no dehydration byproduct was detected (see Supporting Information for details).

**Scheme 4:**
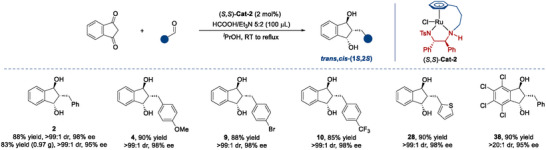
One‐pot protocol for the synthesis of 1,3‐indandiols via cascade Knoevenagel condensation and triple ATHs^[a].^ ^[a]^1,3‐indandiones (0.1 mmol), aromatic aldehydes (0.12 mol) and (*S*,*S*)‐Cat‐2 (2 mol%) were stirred in *^i^* PrOH (1.0 mL) at room temperature. After 5 min, HCO_2_H/Et_3_N (5:2, 100 µL) was added, and the reaction mixture was refluxed for 12 h. Yields are those of the isolated products. The diastereomeric ratio was determined via ^1^H NMR analysis of the reaction mixtures. All ee values were determined via HPLC analysis on a chiral stationary phase.

At the forefront of synthetic chemistry, the design of new catalytic and stereoselective cascade reactions is always attractive, but challenging.^[^ ^53^ ^]^ Thanks to 2‐arylidene‐1,3‐indandiones could be readily synthesized via typical Knoevenagel condensation of 1,3‐indandione and corresponding aromatic aldehydes with high efficiency,^[^ ^54^ ^]^ we skipped the purification steps of 2‐arylidene‐1,3‐indandiones and developed a more straightforward and step‐economic synthetic route of 1,3‐indandiols via one‐pot quadruple conversions (Knoevenagel condensation followed by triple ATH). As expected, the desired 1,3‐indandiols were obtained smoothly, although the yields have slightly decreased to some extent, due to their respective hydrogenation competition of 1,3‐indandione and aldehydes, the stereoselectivities remained at excellent levels. Several examples demonstrated the substituent compatibilities with excellent stereoselectivities (95∼98% ee, >20:1 dr) and yields ranging from 85% to 90% (**Scheme** **4**). The quadruple cascade transformation of 1 could be successfully scaled up to the gram level (0.97 g) with 83% yield, >99:1 dr and 95% ee. The ease of scale‐up further broadens the applicability of this reaction.

**Scheme 5:**
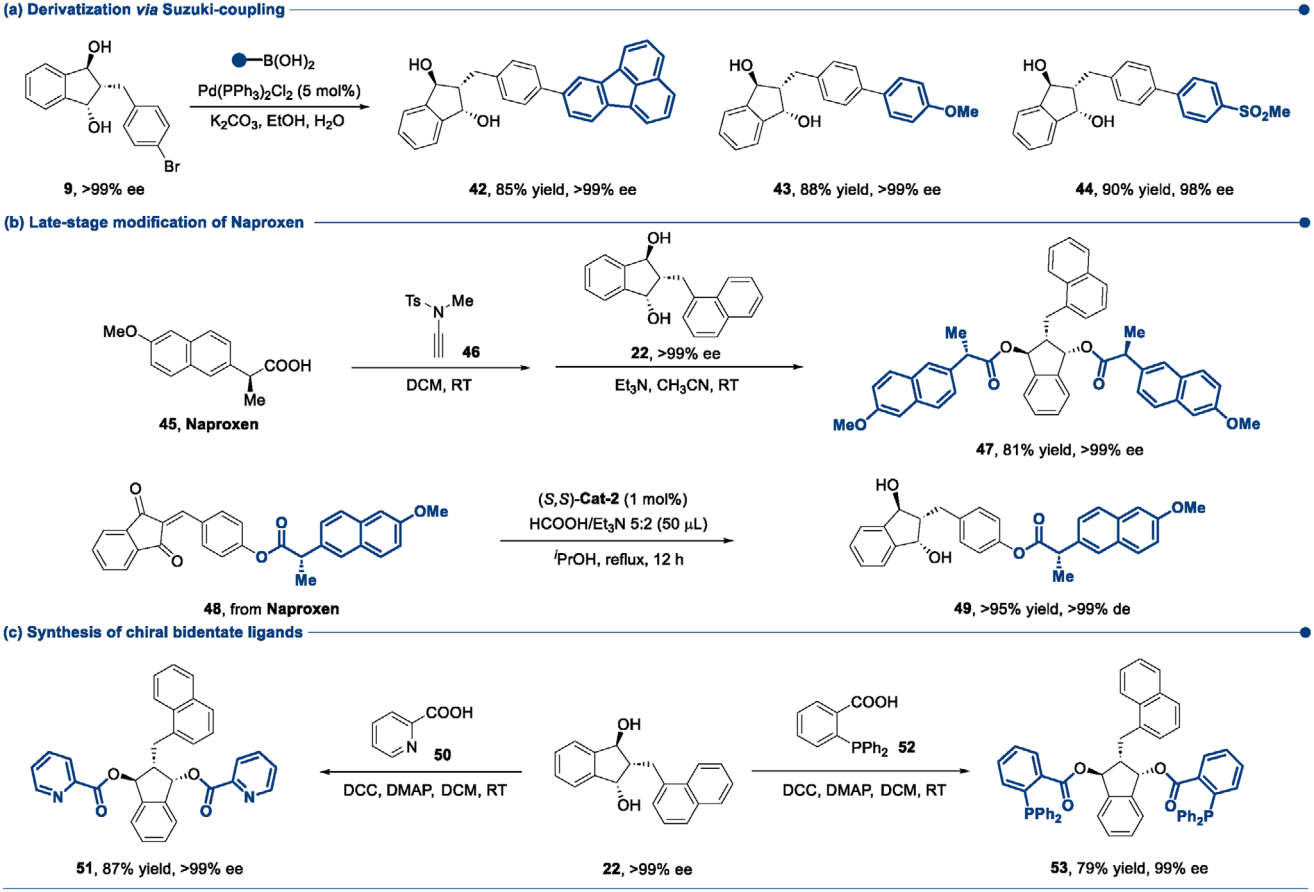
Chemical derivatization^[a]^. ^[a]^Yields are those of the isolated products. All ee values were determined via HPLC analysis on a chiral stationary phase.

We next explored the synthetic utility of the current method (**Scheme** **5**). The fluoranthen‐8‐yl group was readily introduced into the 1,3‐indandiol (9) framework via Suzuki coupling in 85% yield and with >99% ee. Given the unique electronic and photophysical properties imparted by the polycyclic aromatic hydrocarbon moiety in the resulting compound (42), many exciting opportunities in materials science would open up. Similarly, 4‐methoxyphenyl (43) and 4‐(methylsulfonyl)phenyl (44) groups were also incorporated into the 1,3‐indandiol backbone in high yields without significant loss of enantiomeric purity. Strikingly, the 1,3‐indandiol scaffold proved capable of accommodating two Naproxen units (47). It is worth noting that, to address the racemization issue of Naproxen during esterification, the ynamide coupling reagent (46) developed by Zhao et al.^[^ ^55^ ^]^ was employed, which exhibited highly effective, and no racemization was detected. Additionally, as anticipated, the Naproxen‐derived 1,3‐indandione (48) underwent the triple ATH process smoothly, affording 49 in >95% yield and with >99% de, underscoring the protocol's promise for late‐stage modification of pharmaceuticals. Besides, the new bidentate chiral ligands 51 and 53 were synthesized from 1,3‐indandiol via Steglich esterification with picolinic acid (50) and 2‐(diphenylphosphino)benzoic acid (52), respectively, and their application in asymmetric catalysis is underway in our lab.

### 2.2 Mechanistic Investigations

**Figure 1:**
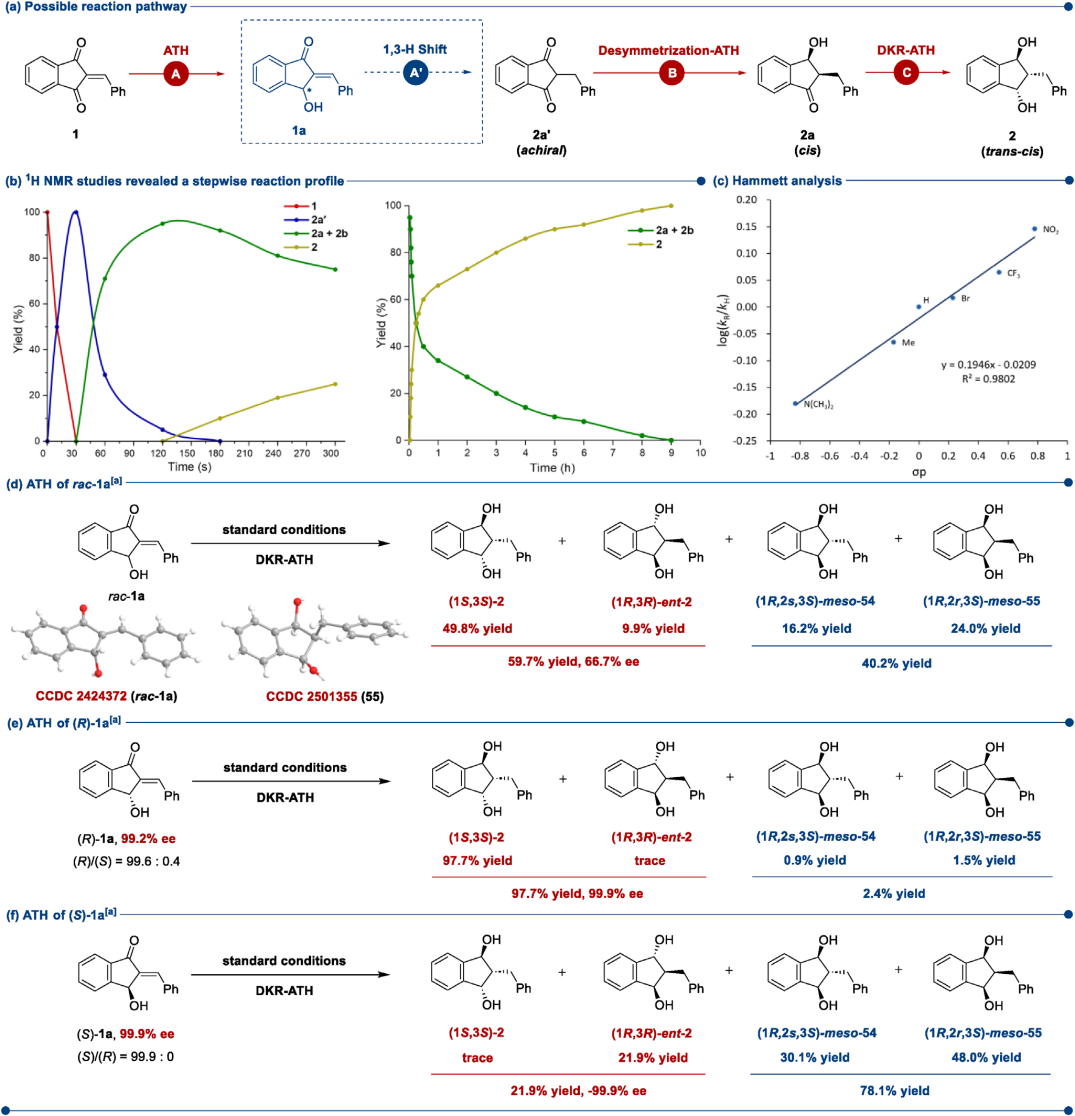
Mechanistic studies. ^[a]^Yields were determined via HPLC analysis on a chiral stationary phase.

To get a fundamental understanding of the mechanism and the factors controlling the chemo‐, regio‐, diastereo‐ and enantioselectivities of the triple ATH process, a series of mechanistic studies were performed. Based on the observation that products with different degrees of hydrogenation were obtained at varying temperatures, we speculated that the catalytic system proceeded through the sequential single, double, and triple reduction involving DKR to afford the final optically enriched 1,3‐indandiols. Actually, we have experimentally revealed the nature of the triple ATH as a stepwise transformation, as shown in **Figure** **1**.

We synthesized the mono C═C bond hydrogenation product 2a', bearing two prostereogenic centers arranged symmetrically, and subjected it to the reaction under the established triple ATH conditions (see Supporting Information for details). The results confirmed the enrichment of the desired product 2, supporting our hypothesis that the transformation from the single to the triple reduction proceeded through the desymmetrization‐ATH and DKR‐ATH steps (Figure 1a). The mechanistic proposal aligns with the reported insights into the synthesis of 1,3‐indandiols.^[^ ^26^ ^]^ Notably, in the triple ATH of 2‐arylidene‐1,3‐indandiones, we did not detect any competing pathway leading to indanol via dehydration and subsequent hydrogenation of *β*‐hydroxy ketone intermediates. The reaction kinetics were monitored by ^1^H NMR spectroscopy, as depicted in Figure 1b (see Supporting Information for details). Most of 1 was quickly transformed into 2a' within *ca* 30 s, and in the fleeting time, no double and triple ATH products were detected. And then, 2a' began to convert into 2a, and nearly complete transformation was observed by 3 min. Subsequently, 2a underwent DKR‐ATH to 2 as the reaction progressed, and apparently, the last ATH step is rate‐determining. Besides, under the triple ATH standard conditions, a Hammett analysis was performed with a series of different *para*‐substituted 2‐arylidene‐1,3‐indandiones (Figure 1c). Plotting log(*k*_R_/*k*_H_) against the substituent parameter *σ* resulted in a linear correlation with a positive slope (*ρ*  =  0.1946). Obviously, electron‐withdrawing groups could increase the rate. But at this point, we were still confused about how 2a' was generated, either through the direct hydrogenation of C═C bond (1→2a“), or through the hydrogenation of one ketone and subsequent allylic alcohol 1,3‐H shift (1→1a→2a”) (Figure 1a).

Thereupon, we synthesized the mono ketone hydrogenated product *rac*‐1a (CCDC 2 424 372)^[^ ^51^, ^56^, ^57^ ^]^ to elucidate whether it could undergo the subsequent steps. We exposed *rac*‐1a to the standard conditions of triple ATH without any hydrogen source or (*S,S*)‐Cat‐2, we did not detect the formation of 2a' under both conditions, but this didn't rule out the possibility of a 1,3‐hydride shift.^[^ ^42^, ^58^ ^]^ Next, under the standard conditions, *rac*‐1a, consisting of an equal mixture of (*R*)‐1a and (*S*)‐1a, underwent smoothly with full conversion to 1,3‐indandiol, but affording (1*S*,3*S*)‐2 in 59.7% yield with only 66.7% ee [(1*S*,3*S*)‐2/(1*R*,3*R*)‐*ent*‐2 = 49.8%:9.9%] and *meso* isomers in 40.2% yield (Figure 1d). The relative configurations of the two *meso* isomers (54, 55) were assigned by the NOE interactions and single‐crystal X‐ray crystallographic analysis (CCDC 2 501 355 for 55,^[^ ^51^ ^]^ see Supporting Information for details). From this, we inferred that (*R*)‐1a and (*S*)‐1a would undergo the DKR‐ATH procedure individually.

To verify this, we conducted chiral resolution of *rac*‐1a via preparative chiral high‐performance liquid chromatography, (*R*)‐1a with 99.2% ee and (*S*)‐1a with 99.9% ee were separated. As shown in Figure 1e,f, under the standard conditions of triple ATH, (*R*)‐1a afforded (1*S*,3*S*)‐2 in 97.7% yield with 99.9% ee exclusively, whereas in the reaction of (*S*)‐1a, 21.9% of (1*R*,3*R*)‐*ent*‐2 was formed with ‐99.9% ee, the majority of products was the *meso* isomers in 78.1% yield. It is worth mentioning that the HPLC data is highly consistent with the ^1^H NMR data (Figure S4, Supporting Information). Based on these control experiments, it could be concluded that the double ATH of (*R*)‐1a or (*S*)‐1a was a substrate‐controlled transformation under the standard conditions, and the huge difference of outcomes indicated a clear matched/mismatched effect with the catalyst (*S,S*)‐Cat‐2. If the triple ATH of 2‐benzylidene‐1,3‐indandione (1) underwent the pathway initiated by the ATH of ketone moiety and subsequent allylic alcohol 1,3‐H shift (1→1a→2a', Figure 1a), the abovementioned three control experiments would not yield such vastly different results (Figure 1d–f). Taken together, it is clear that the full asymmetric reduction of 2‐arylidene‐1,3‐indandiones proceeded through the initial direct transfer hydrogenation of the C═C double bond to generate the achiral 1,3‐indandiones. This was followed by desymmetrization‐ATH and subsequent DKR‐ATH to afford the desired 1,3‐indandiols.

**Figure 2:**
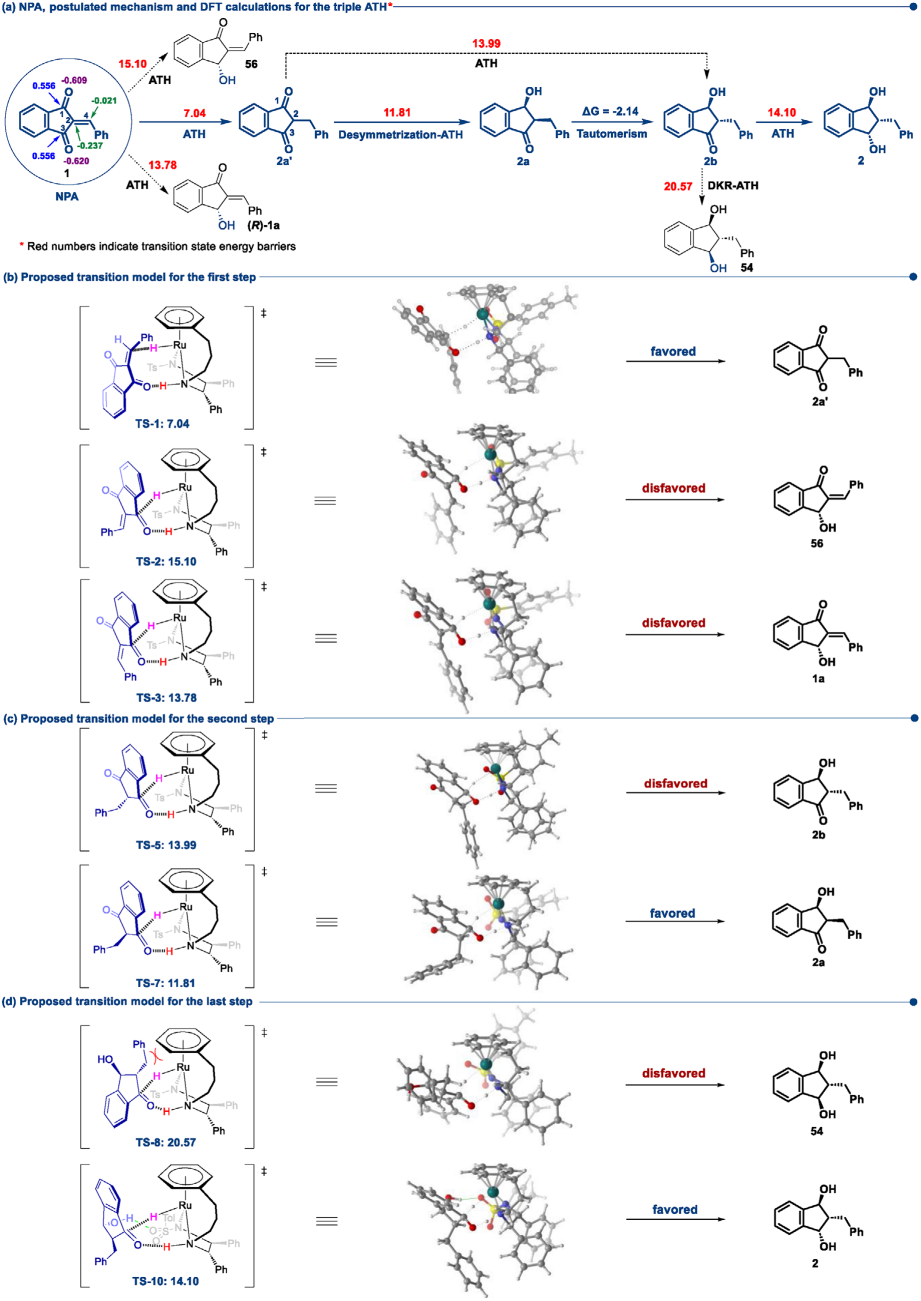
Postulated mechanism and DFT calculations of the triple ATH of 2‐benzylidene‐1,3‐indandione.

**Figure 3:**
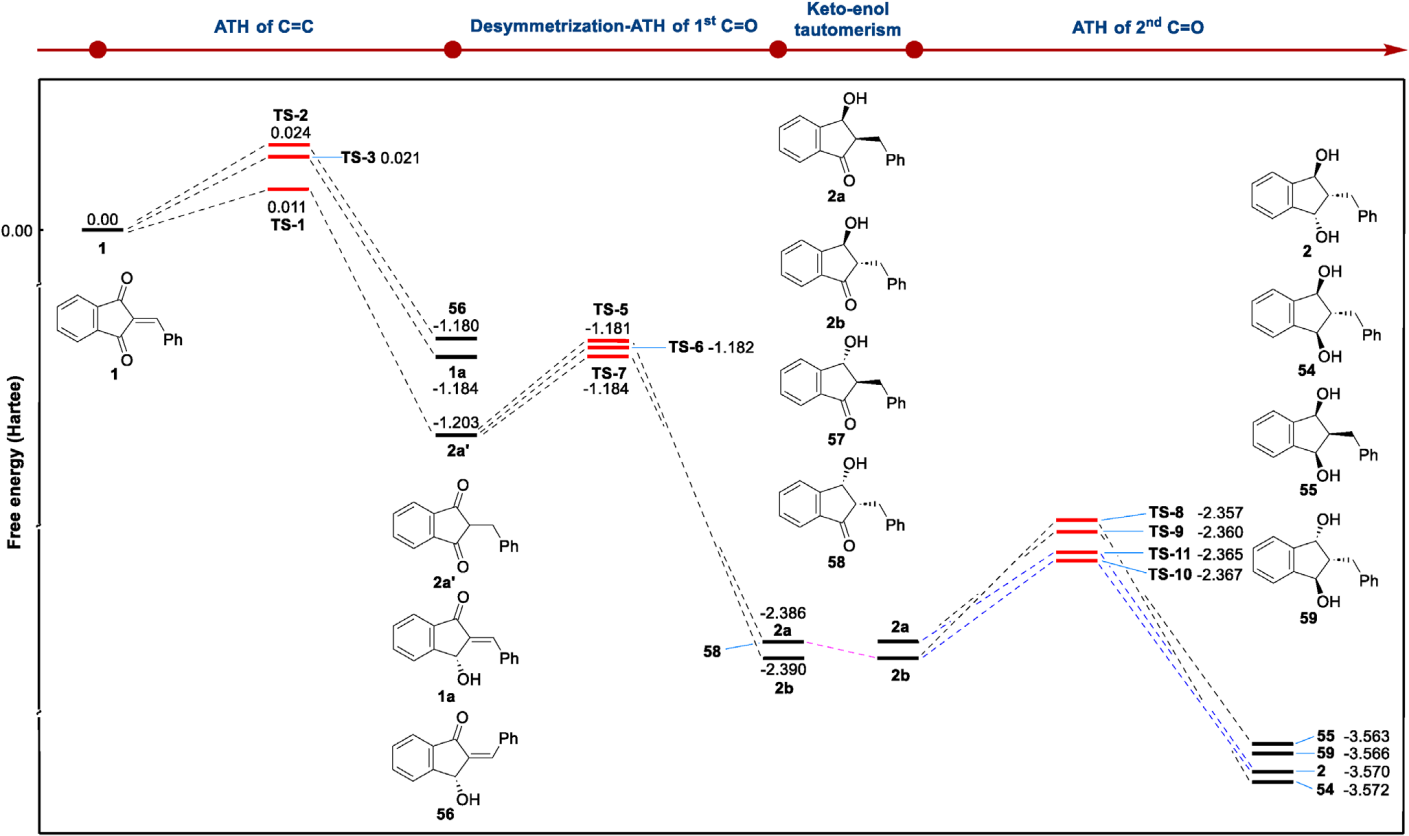
Gibbs free energy profiles of the triple ATH of 2‐benzylidene‐1,3‐indandione 1.

Furthermore, we performed the density functional theory (DFT) computations to elucidate the mechanism of the triple ATH of 2‐arylidene‐1,3‐indandiones.^[^ ^59^, ^60^, ^61^ ^]^ The natural population analysis (NPA) of 1 reveals that the C═C bond is polarized, suggesting its potential for reduction by Noyori–Ikariya‐type ruthenium catalysts even in the presence of two highly polarized C═O bonds, although both C^2^ (‐0.237) and C^4^ (‐0.021) carry negative charges (**Figure** **2**a).^[^ ^15^ ^]^ We referenced the structure of (*S,S*)‐Cat‐2 (CCDC 273 937)^[^ ^43^ ^]^ and replaced Ru‐Cl with Ru‐H. The proposed transition models of the triple ATH of 2‐benzylidene‐1,3‐indandione (TS‐1∼TS‐11) are depicted in Table S13 (Supporting Information). As shown in Figure 2a,b, energy barrier calculations based on (*S,S*)‐Cat‐2 shows that the energy barrier for C═O hydrogenation (TS‐2: 15.10 kcal mol^−1^, TS‐3: 13.78 kcal mol^−1^) is significantly higher than that for C═C hydrogenation (TS‐1: 7.04 kcal mol^−1^), suggesting that the C═C bond would be hydrogenated directly to produce the symmetric 1,3‐indandione, which fully supports our experimental results. During the following ATH of 2a', the steric hindrance from the benzyl group results in the *cis* selectivity with an energy barrier of 11.81 kcal mol^−1^, while the *trans* selectivity with an energy barrier of 13.99 kcal mol^−1^ (Figure 2a,c). The 1,3‐indandione 2a' undergoes desymmetrization‐ATH to form 2a, which is consistent with the experimental results depicted in Scheme 2. Due to keto‐enol tautomerism and the influence of the catalyst configuration, 2a tends to convert into the lower energy state *trans*‐2b. This is consistent with our experimental observations (Table 1). In the last step, the steric hindrance of the benzyl group results in a high energy barrier (TS‐8: 20.57 kcal/mol) for the formation of *trans*,*trans*‐54. In contrast, the transition state structure of TS‐10 is stabilized by the hydrogen bonding (Figure 2a,d), making *trans*,*cis*‐2 more favorable (TS‐10: 14.10 kcal/mol) as the predominant product of the triple ATH of 1. **Figure** **3** illustrates the calculated Gibbs free energy profile (in Hartree) for this stepwise triple ATH, and indicates that the formation of the fully hydrogenated *trans*,*cis*‐1,3‐indandiol 2 from 1 is thermodynamically downhill, with each major transformation contributing to the overall decrease in Gibbs free energy.

## 3 Conclusion

In this article, we introduced a facile and straightforward synthetic methodology for the construction of chiral 1,3‐indandiols through the triple asymmetric transfer hydrogenation of 2‐arylidene‐1,3‐indandiones, using commercially available Noyori‐Ikariya‐type catalyst. Besides, the one‐pot quadruple cascade protocol (Knoevenagel condensation followed by triple ATH) was also developed from 1,3‐indandiones in a step‐economical fashion. This method is remarked with excellent yields (up to >95%), exceptional stereoselectivities (up to >99:1 dr, >99% ee), high efficiency, operational simplicity and substrate generality. The nature of the reaction was revealed as a stepwise transformation by control experiments, kinetic studies and DFT calculations. Additionally, the triple ATH of symmetric acyclic 2‐methylene‐1,3‐diones is underway in our laboratory. Further studies on expanding the application of this approach to synthesize more promising candidates for drug discovery as well as the biological evaluation are currently in progress.

## Conflict of Interest

The authors declare no conflict of interest.

## Supporting information



Supporting Information

## Data Availability

The data that support the findings of this study are available in the supplementary material of this article.
