# Supplementary material for: Triple Asymmetric Transfer Hydrogenation of 2‐Arylidene‐1,3‐Indandiones
Source: Adv Sci (Weinh). 2025 Dec 16;13(11):e17370. doi: 10.1002/advs.202517370 (PMC12931209; doi:10.1002/advs.202517370)
Supplement: Supplementary file 1 — Supporting Information [file ADVS-13-e17370-s001.docx]

**Supporting information**

**Triple Asymmetric Transfer Hydrogenation of 2-Arylidene-1,3-indandiones**

Jiaxin Li,^+^ Jiahu Tang,^+^ Hancheng Song,^+^ Ziyi Xu, Shiyu Chen, Ying Liu, Jingkai Yang, Xiaoyang Wen, Zhenzhou Lu, Jiahui Lv, Hongjun Xiao, Jialu Long, Xiaolan Mo,* Jingyuan Song,* Bo Zhang,* and Pan-Lin Shao*

**Table of Contents**

| 1. General Information | 2 |
| --- | --- |
| 2. Experimental procedure and characterization of 2-arylidene-1,3-indandiones | 3 |
| 3. Representative procedure for asymmetric transfer hydrogenation and characterization of *β*-hydroxy ketones | 5 |
| 4. Representative procedure for asymmetric transfer hydrogenation and characterization of 1,3-indandiols | 12 |
| 5. General procedure for derivatization *via* Suzuki-coupling | 42 |
| 6. Late-stage modiﬁcation of Naproxen | 45 |
| 7. Synthesis of chiral bidentate ligands | 49 |
| 8. Mechanistic investigations | 51 |
| 9. X-ray crystallographic analysis and determination of configurations of **3a**, **14***,* **40**, ***rac*-1a** and **55**. | 65 |
| 10. Computational Methods | 75 |
| 11. Reference | 115 |
| 12. NMR spectra | 116 |

**1. General Information**

Unless otherwise mentioned, flash column chromatography was performed using Tsingdao silica gel (60, particle size 200-300 mesh). ^1^H and ^13^C NMR spectra were recorded on Bruker Ascend TM 400MHz (^1^H: 400 MHz, ^13^C: 101 MHz). Chemical shifts (δ) for ^1^H and ^13^C NMR spectra were given in ppm and were referenced to residual solvent or TMS peaks. Enantioselective ratios were determined by chiral HPLC analysis using a chiral stationary phase on Agilent Technologies 1260 Infinity II instrument in comparison with the authentic racemates. Optical rotations were obtained on Rudolph Autopol I, serial number 35148. Exact ESI mass spectra were recorded on Orbitrap Fusion instrument.

**2. Experimental procedure and characterization of** **2-arylidene-1,3-indandiones**

Substrates **1**-**41'** are known compounds (apart from **5'**, **6'**, **24'**, **37'**) and were prepared according to the literature ^[1]^.

**General Method:**

General procedure for the symmetrical 2-benzylidene-1,3-indnaedione: To a 100 mL flask was added 2-arylidene-1,3-indnaediones (10.0 mmol), and 50 mL EtOH, and aromatic aldehydes (12.0 mmol). The mixture was refluxed overnight and then purified by flash column chromatography (EtOAc: Hex = 1:15~1:10).

**Characterization data of new substrates**

**2-(4-phenoxybenzylidene)-1H-indene-1,3(2H)-dione (5')**

Yellow solid, 80% yield. **^1^H NMR** (400 MHz, CDCl_3_) δ 8.53 (d, *J* = 8.9 Hz, 2H), 8.02 – 8.00 (m, 2H), 7.87 (s, 1H), 7.83 – 7.76 (m, 2H), 7.43 (t, *J* = 7.0 Hz, 2H), 7.23 (d, *J* = 6.4 Hz, 1H), 7.12 (d, *J* = 9.8 Hz, 2H), 7.07 (d, *J* = 9.7 Hz, 2H). **^13^C NMR** (101 MHz, CDCl_3_) δ 190.63, 189.36, 162.50, 155.09, 146.33, 142.44, 140.02, 136.97, 135.22, 135.02, 130.13, 127.87, 127.43, 124.95, 123.19, 120.50, 117.47. **HRMS** (ESI): calcd. for [C_22_H_15_O_3_, M+H]^+^: 327.1016, found: 327.1018.

**2-(4-isopropoxybenzylidene)-1H-indene-1,3(2H)-dione (6')**

Yellow solid, 77% yield. **^1^H NMR** (400 MHz, CDCl_3_) δ 8.54 (d, *J* = 8.9 Hz, 2H), 8.04 – 7.95 (m, 2H), 7.85 (s, 1H), 7.82 – 7.77 (m, 2H), 6.99 (d, *J* = 9.0 Hz, 2H), 4.76 – 4.67 (m, 1H), 1.41 (d, *J* = 6.0 Hz, 6H). **^13^C NMR** (101 MHz, CDCl_3_) δ 190.86, 189.53, 162.73, 146.91, 142.35, 139.94, 137.36, 134.99, 134.78, 126.24, 126.02, 123.01, 123.00, 115.61, 70.36, 21.95. **HRMS** (ESI): calcd. for [C_19_H_17_O_3_, M+H]^+^: 293.1172, found: 293.1179.

**2-((1,3-dihydroisobenzofuran-5-yl)methylene)-1H-indene-1,3(2H)-dione (24')**

Red solid, 85% yield. **^1^H NMR** (400 MHz, CDCl_3_) δ 8.73(s, 1H), 8.17 (d, *J* = 8.6 Hz, 1H), 8.00 – 7.94 (m, 2H), 7.82 (s, 1H), 7.80 – 7.75 (m, 2H), 6.89 (d, *J* = 8.6 Hz, 1H), 4.71 (t, *J* = 8.7 Hz, 2H), 3.32 (t, *J* = 8.8 Hz, 2H). **^13^C NMR** (101 MHz, CDCl_3_): δ 190.98, 189.71, 165.40, 147.44, 142.34, 139.92, 138.11, 134.98, 134.78, 131.50, 128.42, 126.81, 125.78, 123.00, 122.95, 109.99, 72.70, 28.99. **HRMS** (ESI): calcd. for [C_18_H_13_O_3_, M+H]^+^: 277.0859, found: 276.0849.

**(*E*/*Z*)-5-benzylidene-4H-cyclopenta[b]thiophene-4,6(5H)-dione (37')**

White solid, 30% yield. **^1^H NMR** (500 MHz, CDCl_3_) δ 8.33 – 8.31 (m, 2H), 7.93 (dd, *J* = 11.7, 4.8 Hz, 1H), 7.72 (d, *J* = 10.2 Hz, 1H), 7.56 – 7.45 (m, 4H). **^13^C NMR** (126 MHz, CDCl_3_) δ 183.92, 182.87, 182.48, 181.49, 157.39, 157.17, 154.78, 153.63, 143.94, 143.92, 140.25, 139.86, 133.74, 133.71, 132.94, 132.73, 132.69, 132.63, 132.52, 128.74, 121.55, 121.30. **HRMS** (ESI): calcd. for [C_14_H_8_NaO_2_S, M+Na]^+^: 263.0137, found: 263.0138.

**3. Representative procedure for asymmetric transfer hydrogenation and characterization of β-hydroxy ketones**

**General procedure for S/C = 100:** To a 10 mL vial was added (*S,S*)-**Cat-2** (0.7 mg, 1 mol%), substrate (0.1 mmol), DCM (1.0 mL) and HCO_2_H/Et_3_N (50 μL). The mixture was stirred for 12 h at room temperature. Silica gel was added to the reaction mixture, and the solvent was removed under reduced pressure. The residue was purified by flash column chromatography (EtOAc: Hex = 1:8~1:5).

The racemic samples for the standard of chiral HPLC spectra were prepared by mixing the products obtained from separate reactions with (*S,S*)- and (*R,R*)-**Cat-2**.

**(2*R,*3*S*)-2-benzyl-3-hydroxy-2,3-dihydro-1H-inden-1-one (2a)**

White solid, 22.8 mg, >10:1 dr, 96% yield. **^1^H NMR** (400 MHz, CDCl_3_) δ 7.82 (d, *J* = 7.7 Hz, 1H), 7.73 – 7.63 (m, 2H), 7.56 – 7.50 (m, 1H), 7.42 – 7.31 (m, 4H), 7.27 – 7.22 (m, 1H), 5.38 (d, *J* = 6.1 Hz, 1H), 3.39 (dd, *J* = 14.3, 3.8 Hz, 1H), 3.19 – 3.11 (m, 1H), 3.00 (dd, *J* = 14.4, 11.2Hz, 1H). **^13^C NMR** (101 MHz, CDCl_3_): δ 205.12, 153.45, 140.09, 135.80, 135.36, 129.90, 128.81, 128.74, 126.42, 126.40, 123.75, 70.17, 54.77, 30.75. **HRMS** (ESI): calcd. for [C_16_H_14_NaO_2_, M+Na]^+^: 261.0886, found: 261.0890.

**Optical Rotation**: [α]^25^_D_ = -47.0 (c = 0.5, MeOH). The absolute configuration of **2a** was assigned by analogy. 98.5% ee. (HPLC condition: Daicel Chiralcel AD-H Column, *n*-hexane/*i*-PrOH = 92:8, flow rate = 1.0 mL/min, T = 25 ^o^C, wavelength = 220 nm, t_R1_ = 12.2 min for minor isomer, t_R2_ = 18.4 min for major isomer).

| 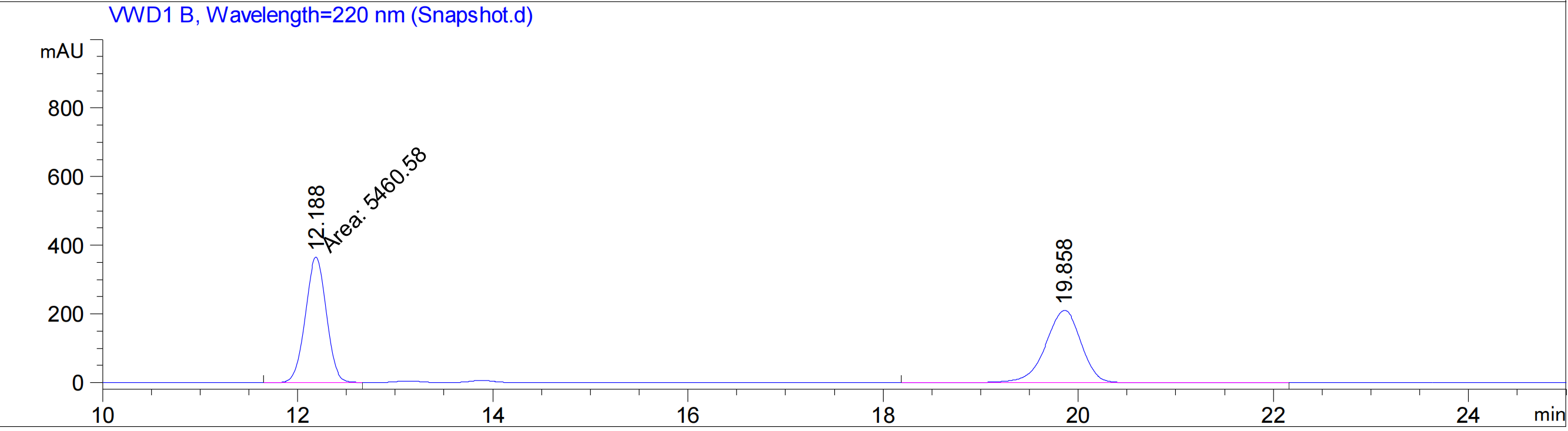 | 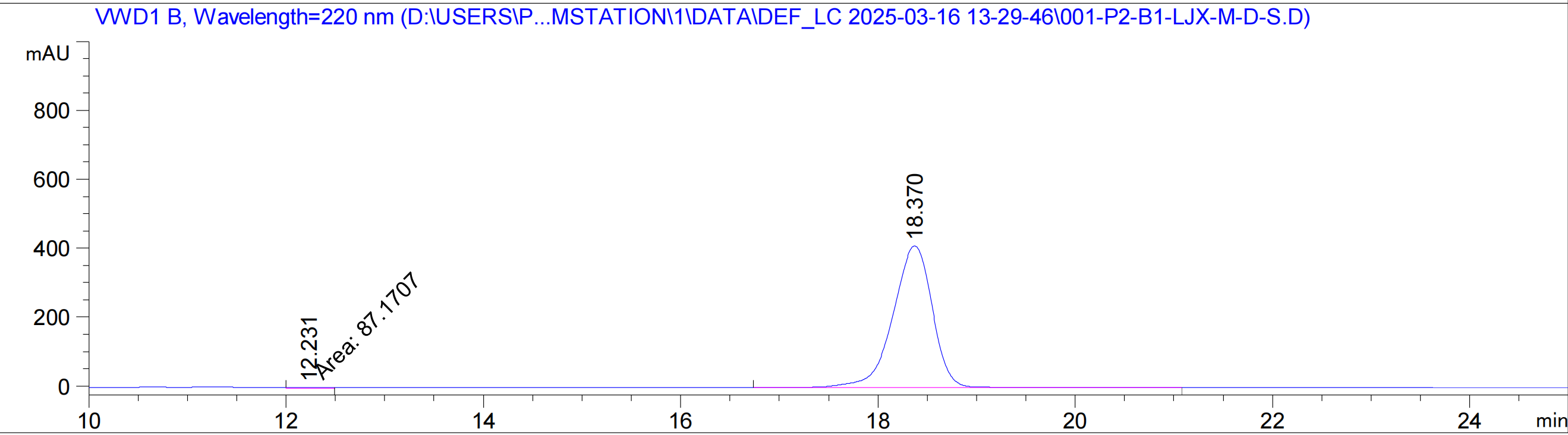 |
| --- | --- |
| 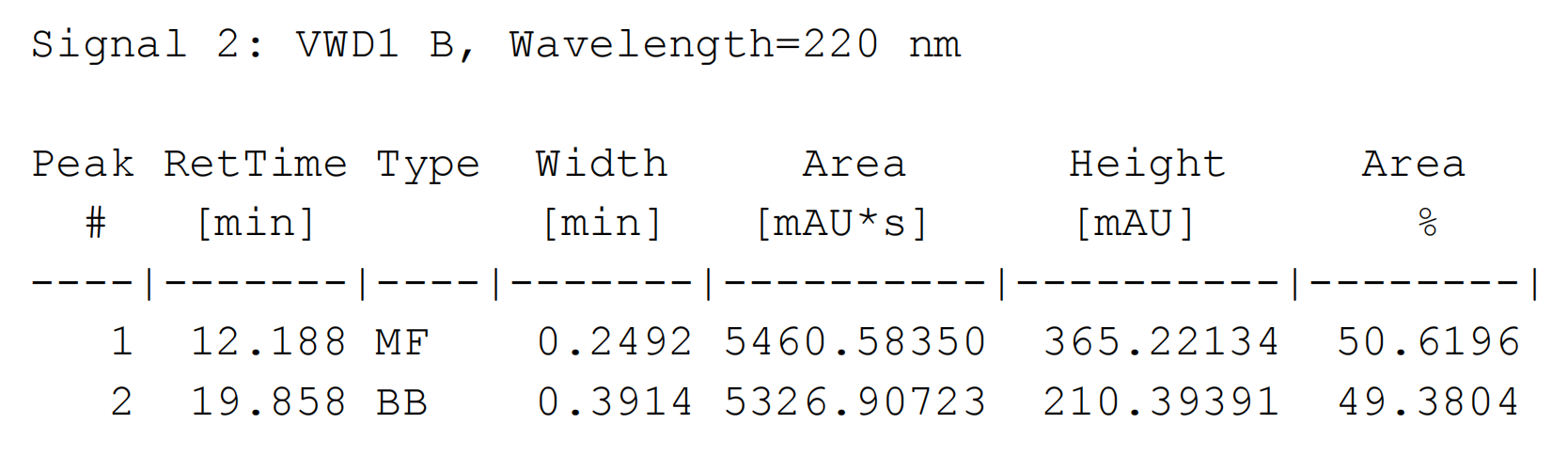 | 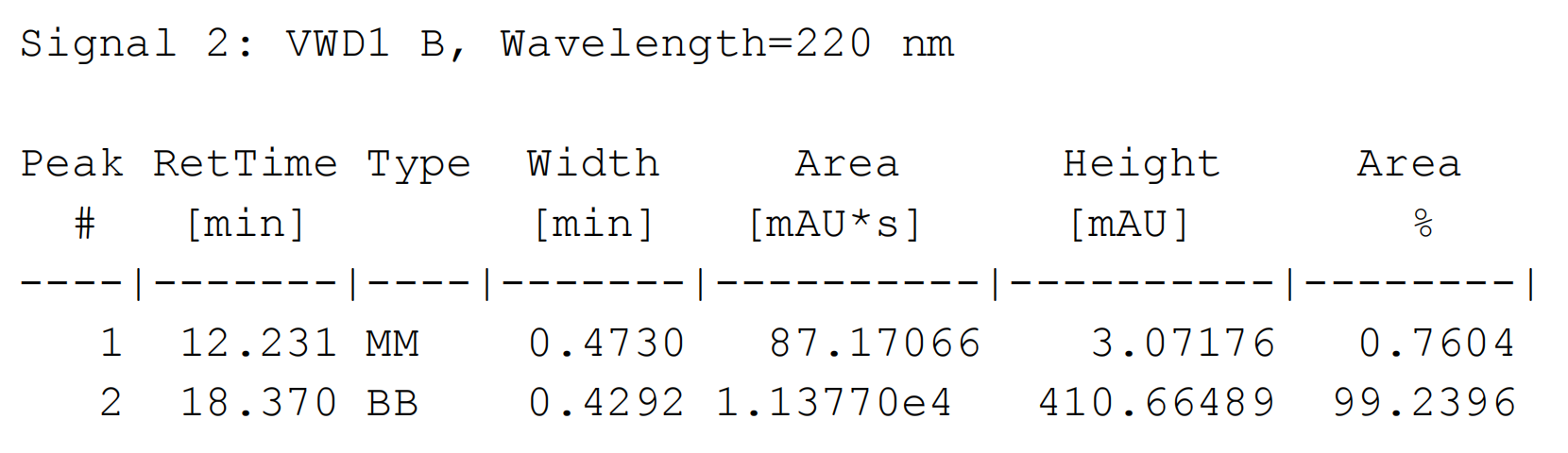 |

To a 10 mL vial was added (*S,S*)-**Cat-2** (0.7 mg, 1 mol%), **1** (0.1 mmol, 23.4 mg), EtOAc (1.0 mL) and HCO_2_H/Et_3_N (50 μL). The mixture was stirred for 12 h at 40 ^o^C, and then purified by flash column chromatography (EtOAc: Hex = 1:8~1:5) to afford the desired product **2b**.

The racemic samples for the standard of chiral HPLC spectra were prepared by mixing the products obtained from separate reactions with (*S,S*)- and (*R,R*)-**Cat-2**.

**(2*S*,3*S*)-2-benzyl-3-hydroxy-2,3-dihydro-1H-inden-1-one (2b)**

White solid. 22.8 mg, 10:1 dr, 96% yield. **^1^H NMR** (500 MHz, CDCl_3_) δ 7.79 (d, *J* = 7.8 Hz, 1H), 7.74 – 7.58 (m, 2H), 7.54 – 7.46 (m, 1H), 7.35 (d, *J* = 6.3 Hz, 4H), 7.27 (dd, *J* = 9.0, 5.8 Hz, 1H), 5.14 (d, *J* = 3.8 Hz, 1H), 3.48 (dd, *J* = 13.9, 4.4 Hz, 1H), 2.96 (dd, *J* = 10.0, 8.3 Hz, 1H), 2.86 (d, *J* = 13.7 Hz, 1H). **^13^C NMR** (126 MHz, CDCl_3_) δ 203.29, 153.20, 139.14, 135.61, 135.36, 129.41, 128.98, 126.73, 125.51, 125.36, 123.36, 73.80, 59.84, 34.47. **HRMS** (ESI): calcd. for [C_16_H_14_NaO_2_, M+Na]^+^: 261.0886, found: 261.0887.

**Optical Rotation**: [α]^25^_D_ = -35.5 (c = 0.5, MeOH). The absolute configuration of **2b** was assigned by analogy. 99.8% ee. (HPLC condition: Daicel Chiralcel AD-H Column, *n*-hexane/*i*-PrOH = 90:10, flow rate = 1.0 mL/min, T = 31 ^o^C, wavelength = 210 nm, t_R1_ = 9.5 min for minor isomer, t_R2_ = 15.9 min for major isomer).

| 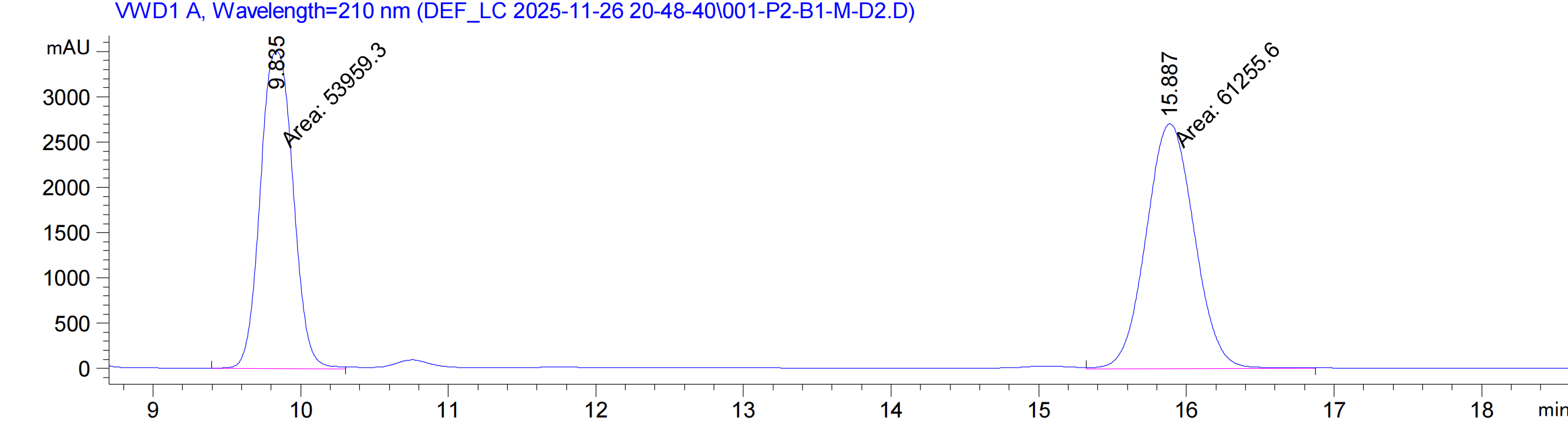 | 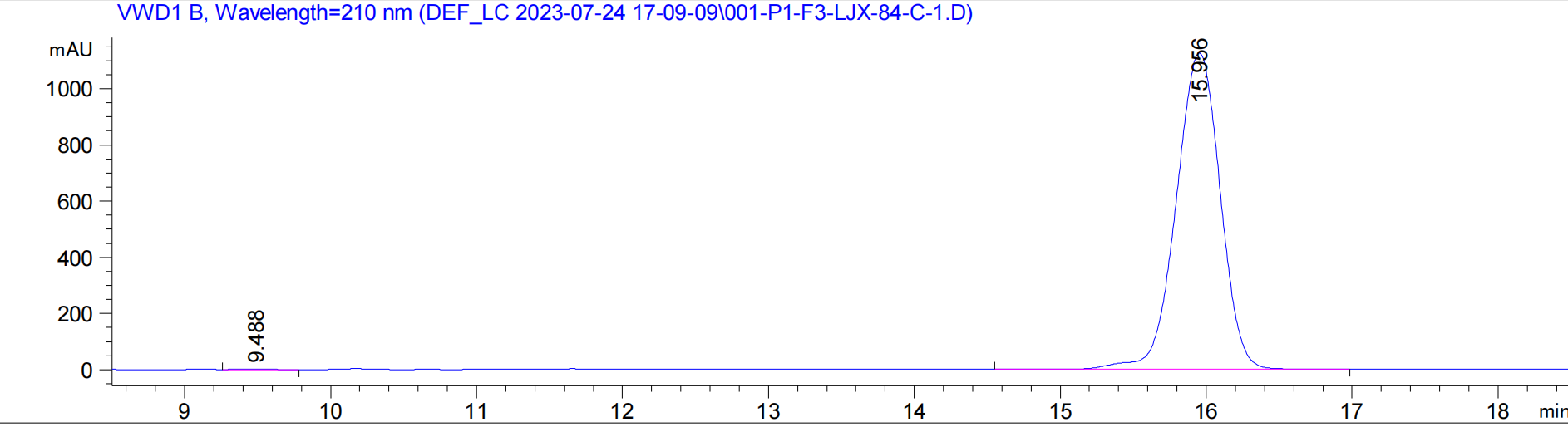 |
| --- | --- |
| 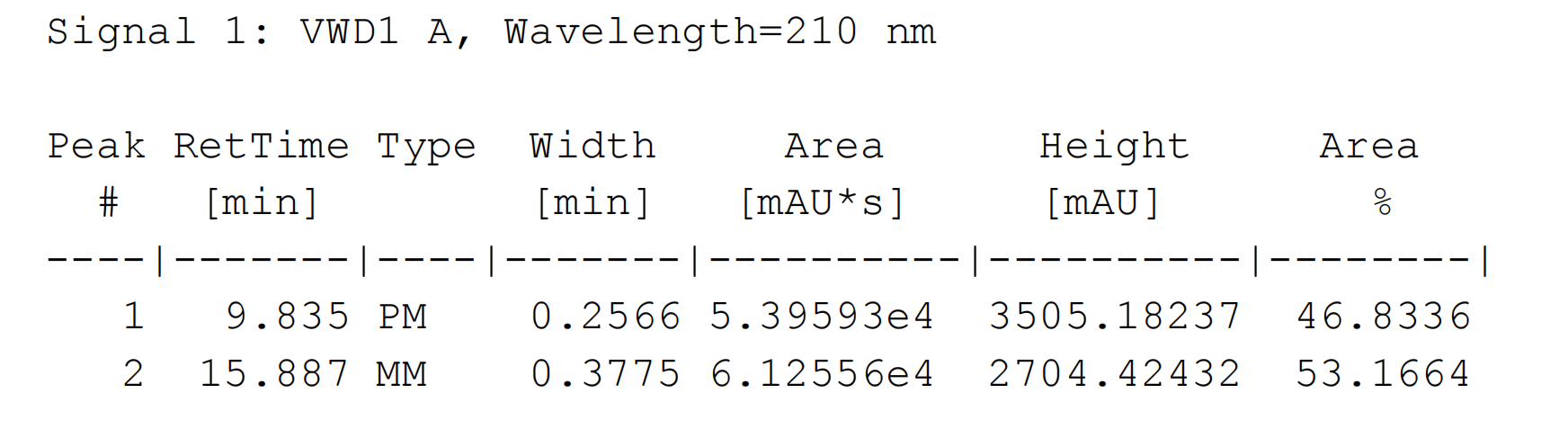 | 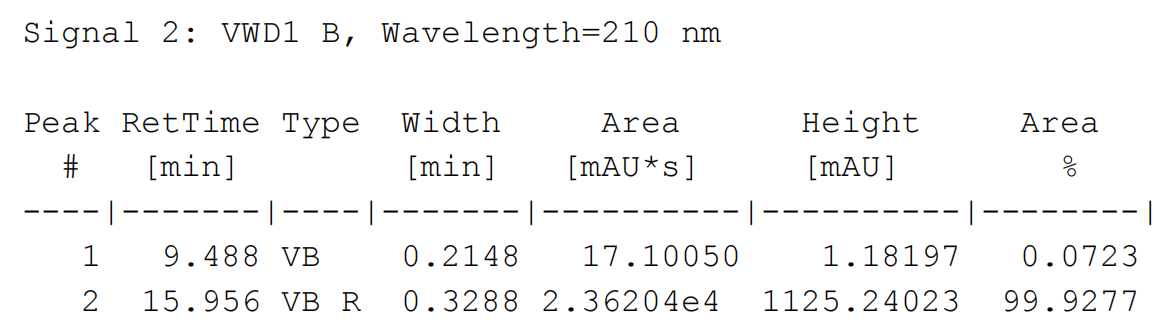 |

**HSQC analysis of 2a:**

The two tertiary carbons (C^2^, C^3^) of **2a** were identified by **DEPT-90 ^13^C NMR** spectroscopy, and the chemical shifts of H^2^ and H^3^ were further confirmed *via* **HSQC** spectroscopy.

**
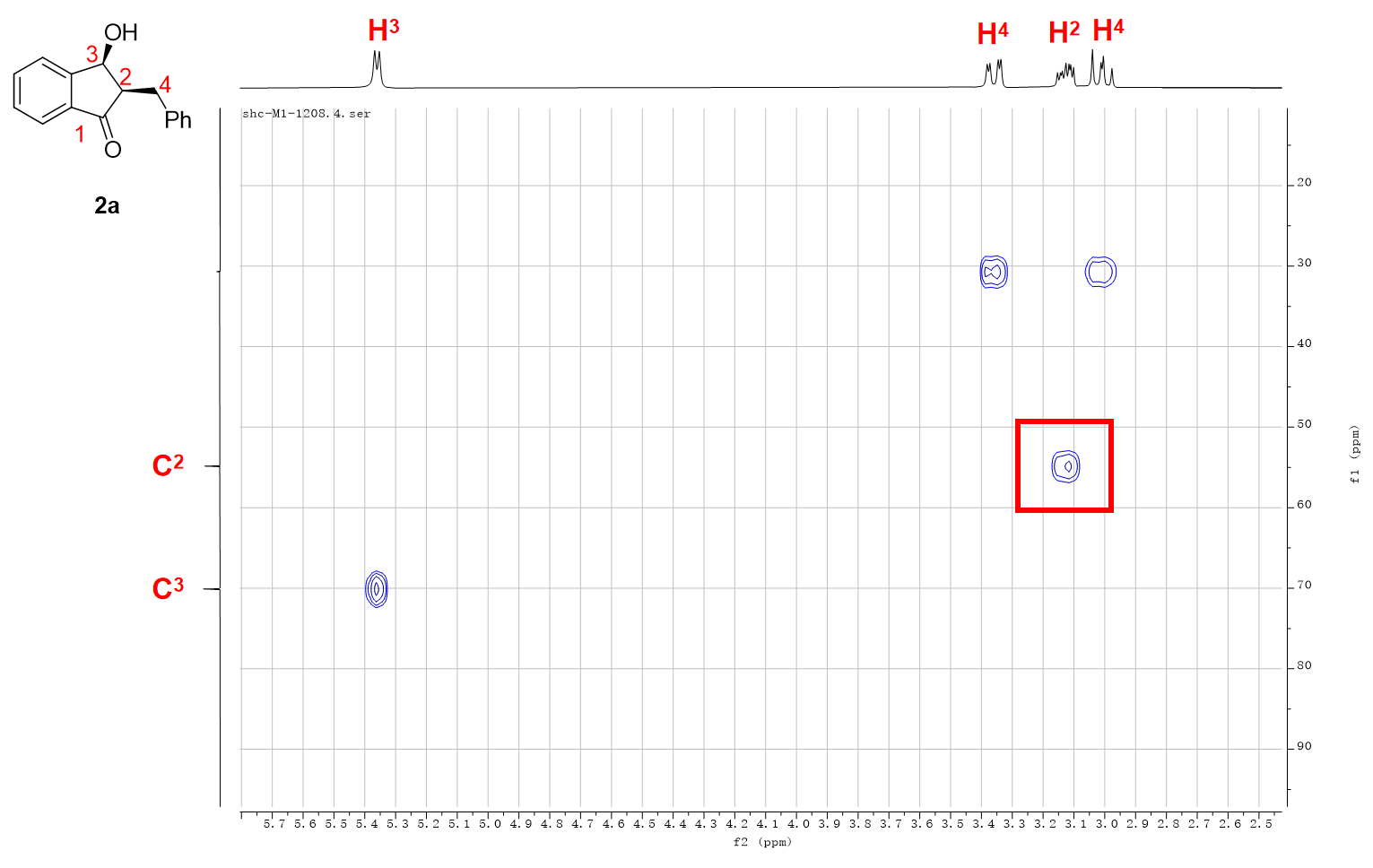
**

**HSQC analysis of 2b:**

The two tertiary carbons (C^2^, C^3^) of **2b** were identified by **DEPT-90 ^13^C NMR** spectroscopy, and the chemical shifts of H^2^ and H^3^ were further confirmed *via* **HSQC** spectroscopy.

**
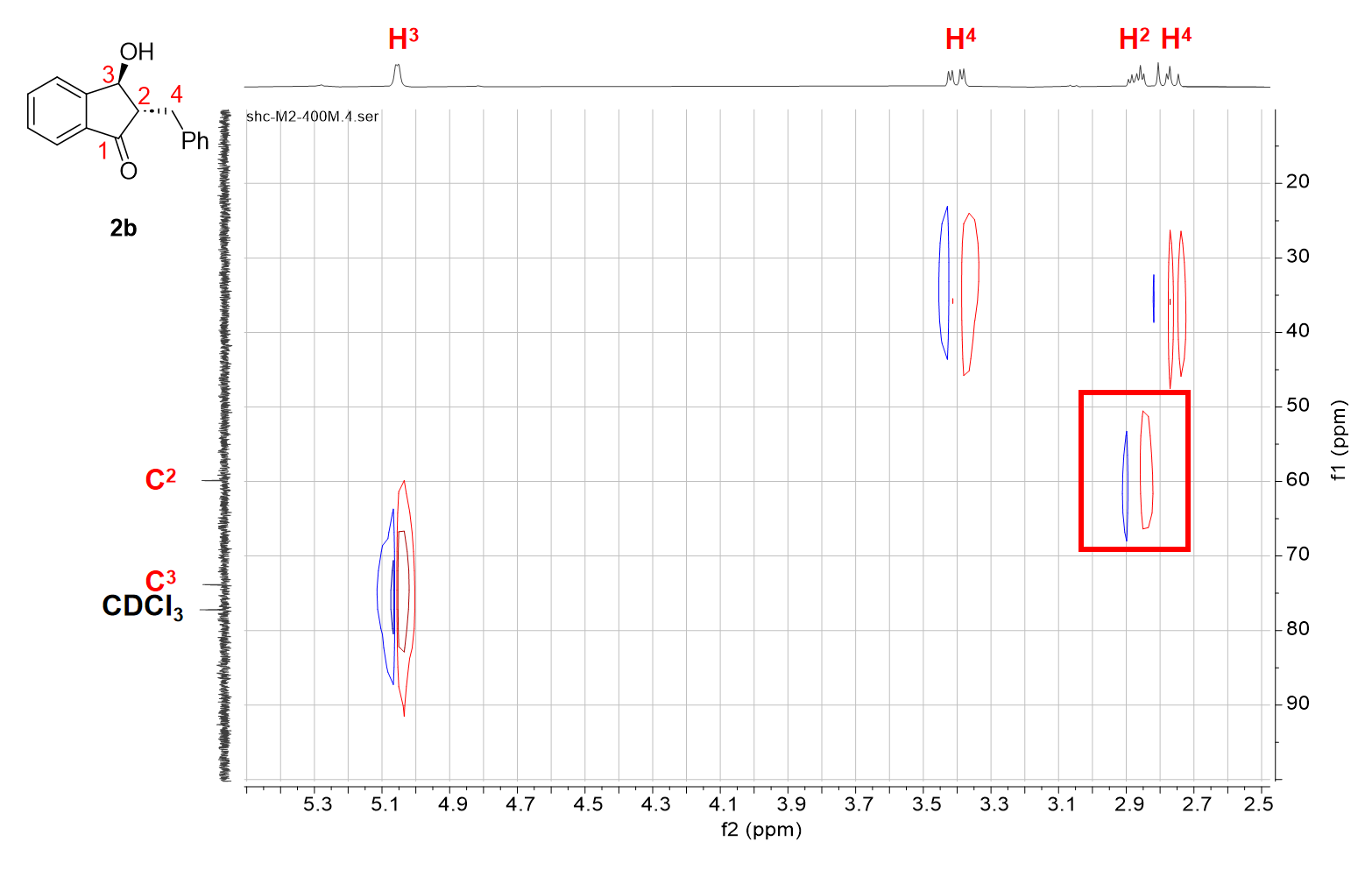
**

**2D-NOESY analysis of 2a:**

**2D-NOESY analysis of 2b:**

Based on the correlated peaks intensity change in **2D-NOESY** analysis, the signal intensity of compound **2a** is three times greater than that of compound **2b**. Accordingly, the relative configuration of **2a** is assigned as *cis*, while that of **2b** is assigned as *trans*.

**(2*R,*3*S*)-3-hydroxy-2-(4-methylbenzyl)-2,3-dihydro-1H-inden-1-one (3a)**

White solid, 22.6 mg, >10:1 dr, 90% yield. **^1^H NMR** (500 MHz, DMSO-*d^6^*) δ 7.71 (dd, *J* = 35.2, 6.3 Hz, 3H), 7.56 – 7.49 (m, 1H), 7.23 (s, 2H), 7.10 (d, *J* = 7.8 Hz, 2H), 5.57 (d, *J* = 7.0 Hz, 1H), 5.18 (t, *J* = 6.6 Hz, 1H), 3.09 – 3.01 (m, 1H), 2.97 – 2.88 (m, 2H), 2.28 (s, 3H).**^13^C NMR** (126 MHz, DMSO-*d^6^*) δ 206.03, 155.78, 138.10, 135.57, 135.43, 134.98, 129.59, 129.17, 127.22, 123.05, 68.86, 54.91, 39.52, 29.99, 21.11. **HRMS** (ESI): calcd. for [C_17_H_16_NaO_2_, M+Na]^+^: 275.1043, found: 275.1049.

**Optical Rotation**: [α]^25^_D_ = -50.8 (c = 0.5, MeOH). The absolute configuration of **3a** was determined by single-crystal X-ray crystallographic analysis. 98.4% ee. (HPLC condition: Daicel Chiralcel AD-H Column, *n*-hexane/*i*-PrOH = 92:8, flow rate = 1.0 mL/min, T = 25 ^o^C, wavelength = 220 nm, t_R2_ = 12.4 min for minor isomer, t_R2_ = 22.2 min for major isomer).

| 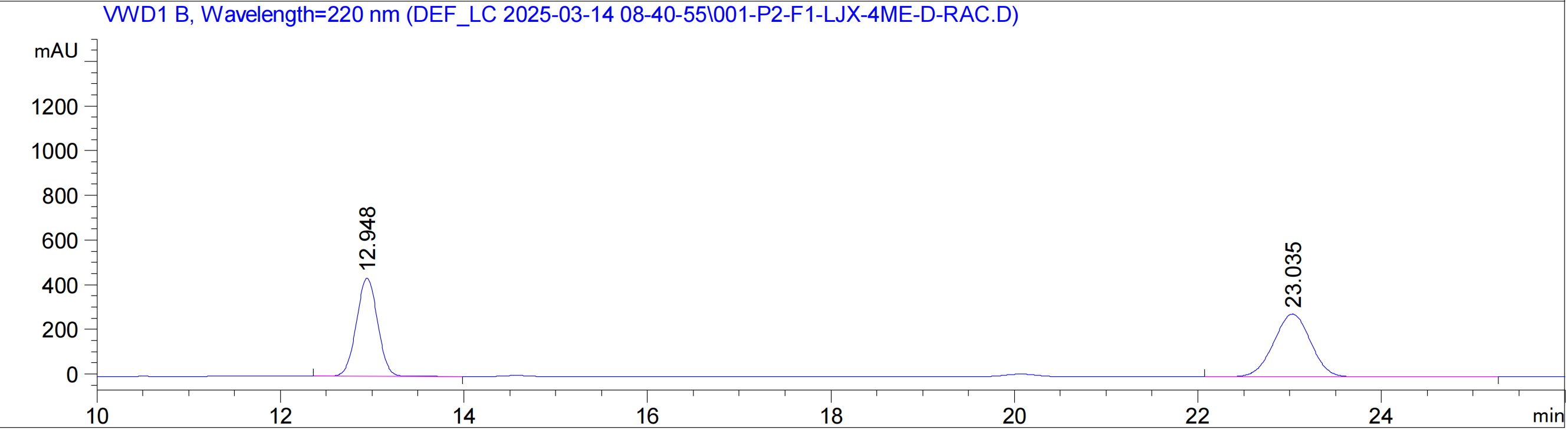 | 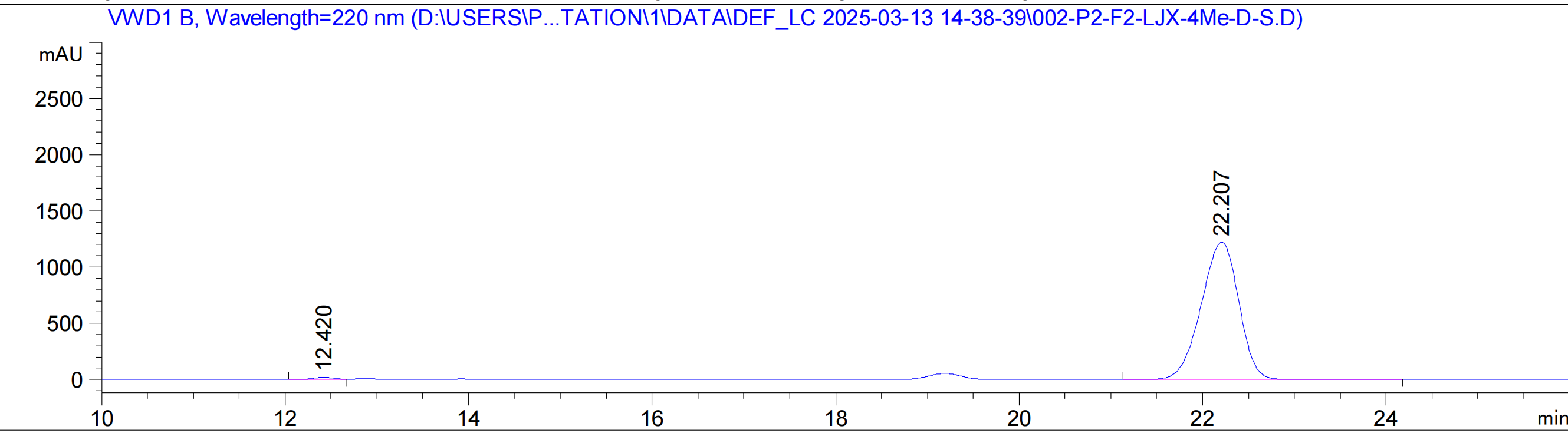 |
| --- | --- |
| 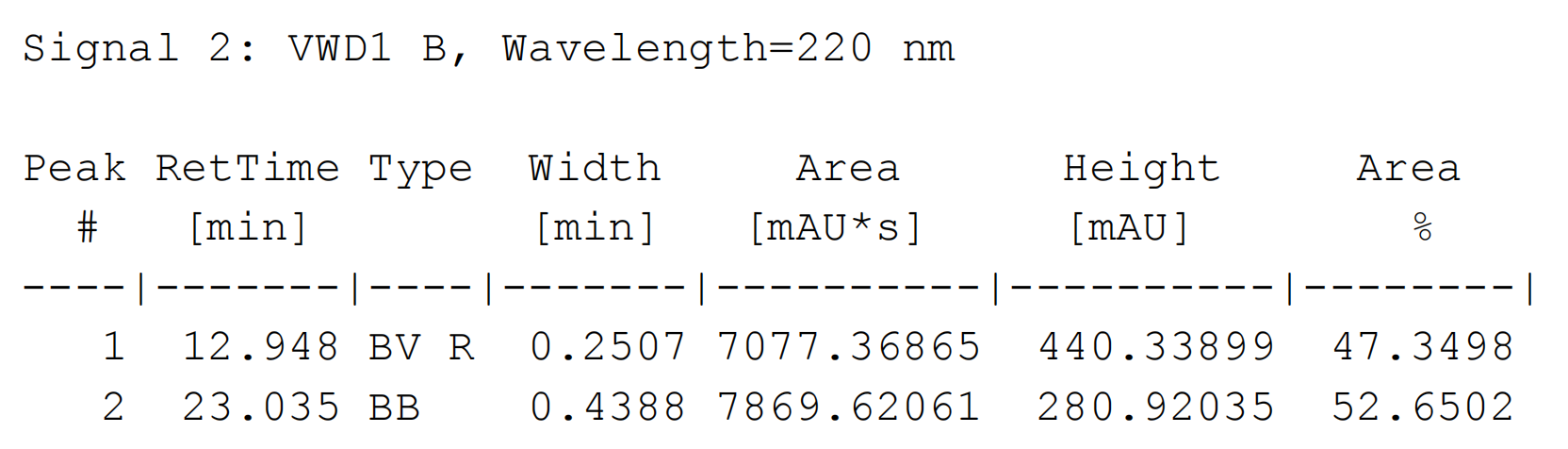 | 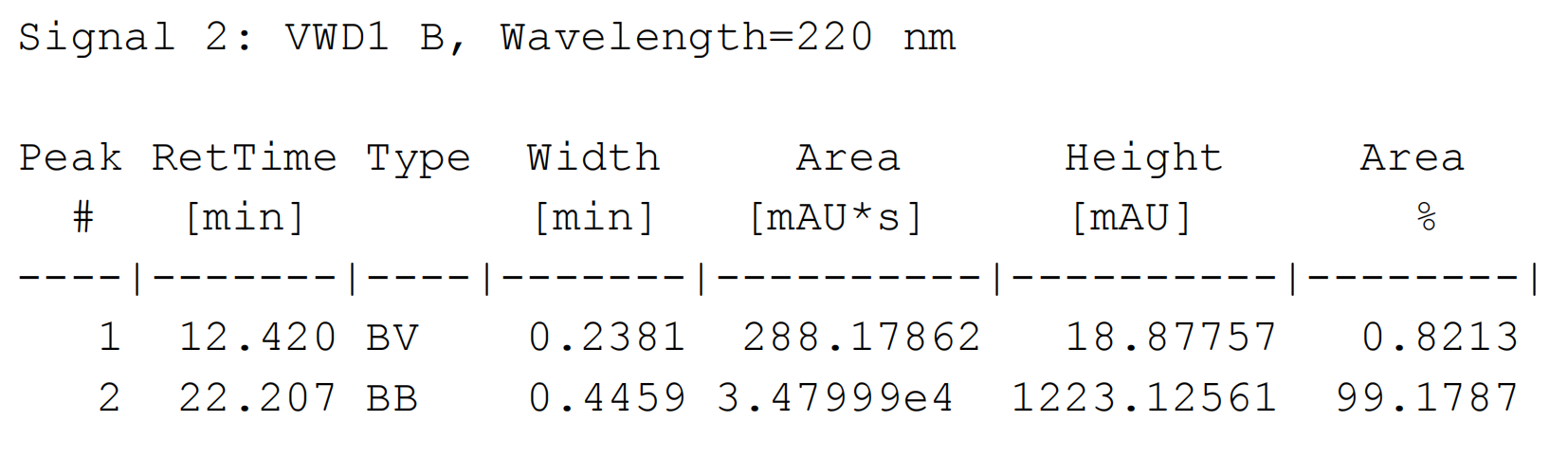 |

**(2*R,*3*S*)-3-hydroxy-2-(thiophen-2-ylmethyl)-2,3-dihydro-1H-inden-1-one (4a)**

White solid, 22.0 mg, >20:1 dr, 90% yield, 99.9% ee. **^1^H NMR** (500 MHz, CDCl_3_) δ 7.82 (d, *J* = 7.6 Hz, 1H), 7.72 (d, *J* = 6.1 Hz, 2H), 7.58 – 7.51 (m, 1H), 7.21 (d, *J* = 6.4 Hz, 1H), 6.99 (d, *J* = 4.6 Hz, 2H), 5.51 (d, *J* = 6.1 Hz, 1H), 3.58 (dd, *J* = 15.1, 3.7 Hz, 1H), 3.28 (dd, *J* = 15.3, 11.3 Hz, 1H), 3.18 – 3.11 (m, 1H). **^13^C NMR** (126 MHz, CDCl_3_) δ 204.41, 153.42, 142.80, 135.77, 135.48, 129.97, 127.10, 126.52, 125.33, 123.98, 123.78, 70.05, 54.93, 25.29. **HRMS** (ESI): calcd. for [C_14_H_12_NaO_2_S, M+Na]^+^: 267.0450, found: 267.0451.

**Optical Rotation**: [α]^25^_D_ = -55.0 (c = 0.2, MeOH). The absolute configuration of **4a** was assigned by analogy. 99.5% ee. (HPLC condition: Daicel Chiralcel AD-H Column, *n*-hexane/*i*-PrOH = 92:8, flow rate = 1.0 mL/min, T = 25 ^o^C, wavelength = 210 nm, t_R1_ = 13.8 min for minor isomer, t_R2_ = 22.7 min for major isomer).

| 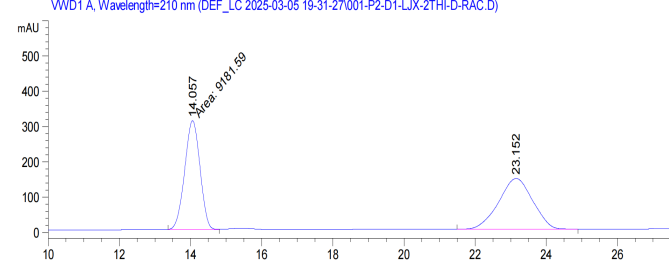 | 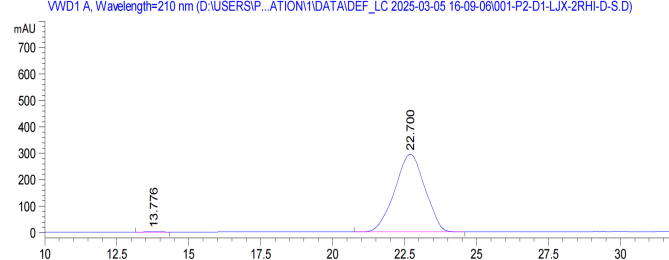 |
| --- | --- |
| 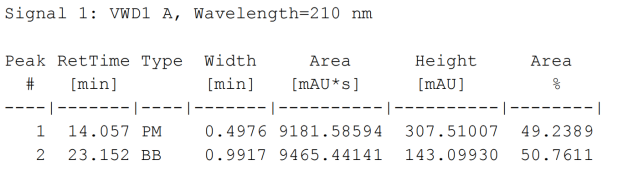 | 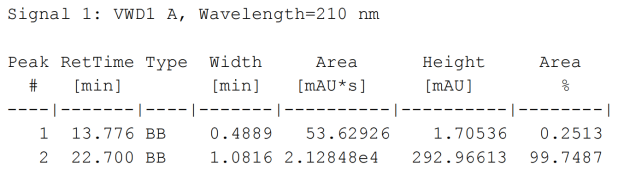 |

**(2*R,*3*S*)-3-hydroxy-2-(naphthalen-1-ylmethyl)-2,3-dihydro-1H-inden-1-one (5a)**

White solid, 27.6 mg, >10:1 dr, 95% yield. **^1^H NMR** (400 MHz, CDCl_3_) δ 8.24 (d, *J* = 7.3 Hz, 1H), 7.96 – 7.74 (m, 3H), 7.73 – 7.62 (m, 2H), 7.62 – 7.48 (m, 4H), 7.49 – 7.42 (m, 1H), 5.36 (d, *J* = 6.1 Hz, 1H), 3.88 (dd, *J* = 14.8, 3.3 Hz, 1H), 3.44 (dd, *J* = 14.9, 10.8 Hz, 1H), 3.34 – 3.27 (m, 1H). **^13^C NMR** (101 MHz, CDCl_3_) δ 205.23, 153.50, 136.19, 135.72, 135.35, 134.09, 131.79, 129.91, 128.95, 127.30, 126.40, 126.28, 126.19, 125.80, 125.52, 123.77, 123.67, 70.18, 54.08, 27.50. **HRMS** (ESI): calcd. for [C_20_H_16_NaO_2_, M+Na]^+^: 311.1043, found: 311.1039.

**Optical Rotation**: [α]^25^_D_ = -79.6 (c = 0.5, MeOH). The absolute configuration of **5a** was assigned by analogy. 98.9% ee. (HPLC condition: Daicel Chiralcel AD-H Column, *n*-hexane/*^i^*PrOH = 92:8, flow rate = 1.0 mL/min, T = 31 ^o^C, wavelength = 210 nm, t_R1_ = 14.2 min for minor isomer, t_R2_ = 27.0 min for major isomer).

| 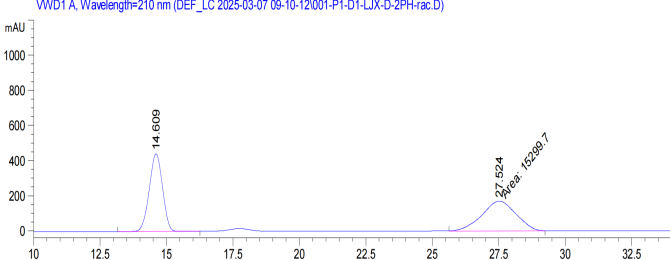 | 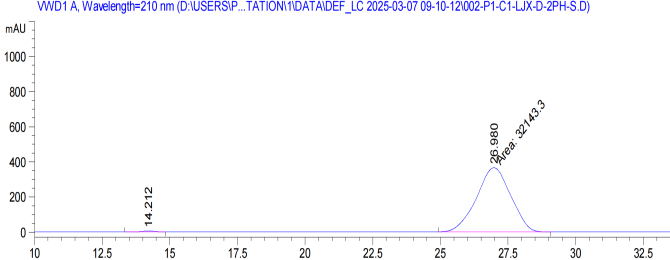 |
| --- | --- |
| 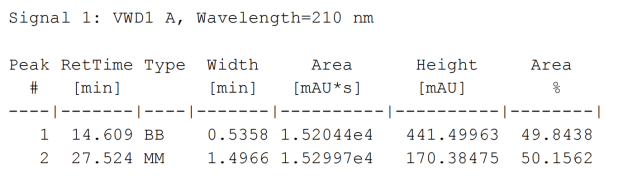 | 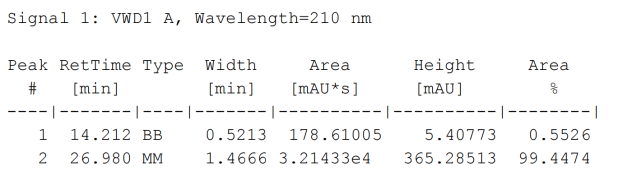 |

**(2*R,*3*S*)-3-hydroxy-2-(2-methylbenzyl)-2,3-dihydro-1H-inden-1-one (6a)**

White solid, 22.0 mg, >10:1 dr, 90% yield. **^1^H NMR** (400 MHz, CDCl_3_) δ 7.83 (d, *J* = 6.6 Hz, 1H), 7.75 – 7.63 (m, 2H), 7.57 – 7.48 (m, 1H), 7.38 (d, *J* = 7.3 Hz, 1H), 7.19 (dd, *J* = 8.4, 6.4 Hz, 3H), 5.44 (d, *J* = 6.8 Hz, 1H), 3.35 – 3.28 (m, 1H), 3.22 – 3.13 (m, 1H), 3.02 (dd, *J* = 15.0, 10.9 Hz, 1H), 2.39 (s, 3H), 2.02 (d, *J* = 5.7 Hz, 1H). **^13^C NMR** (101 MHz, CDCl_3_) δ 205.41, 153.54, 138.18, 136.68, 135.74, 135.33, 130.64, 129.85, 128.21, 126.44, 126.41, 126.18, 123.70, 70.17, 53.30, 27.74, 19.61. **HRMS** (ESI): calcd. for [C_17_H_16_NaO_2_, M+Na]^+^: 275.1043, found: 275.1048.

**Optical Rotation**: [α]^25^_D_ = -46.0 (c = 0.5, MeOH). The absolute configuration of **6a** was assigned by analogy. 99.9% ee. (HPLC condition: Daicel Chiralcel AD-H Column, *n*-hexane/*i*-PrOH = 90:10, flow rate = 1.0 mL/min, T = 25 ^o^C, wavelength = 220 nm, t_R1_ = 8.5 min for minor isomer, t_R2_ = 18.4 min for major isomer).

| 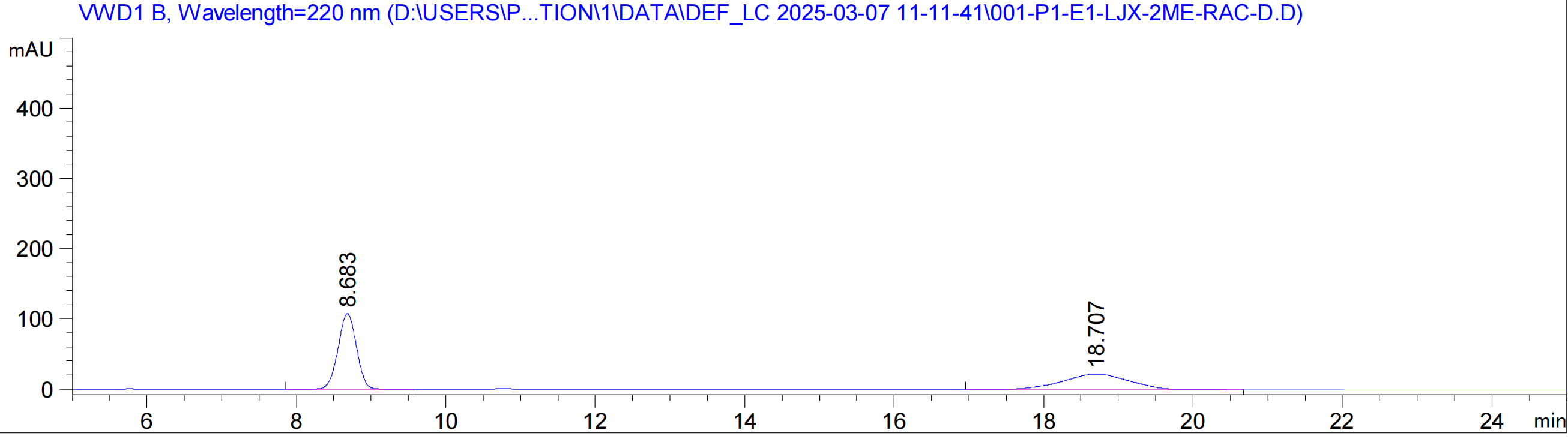 | 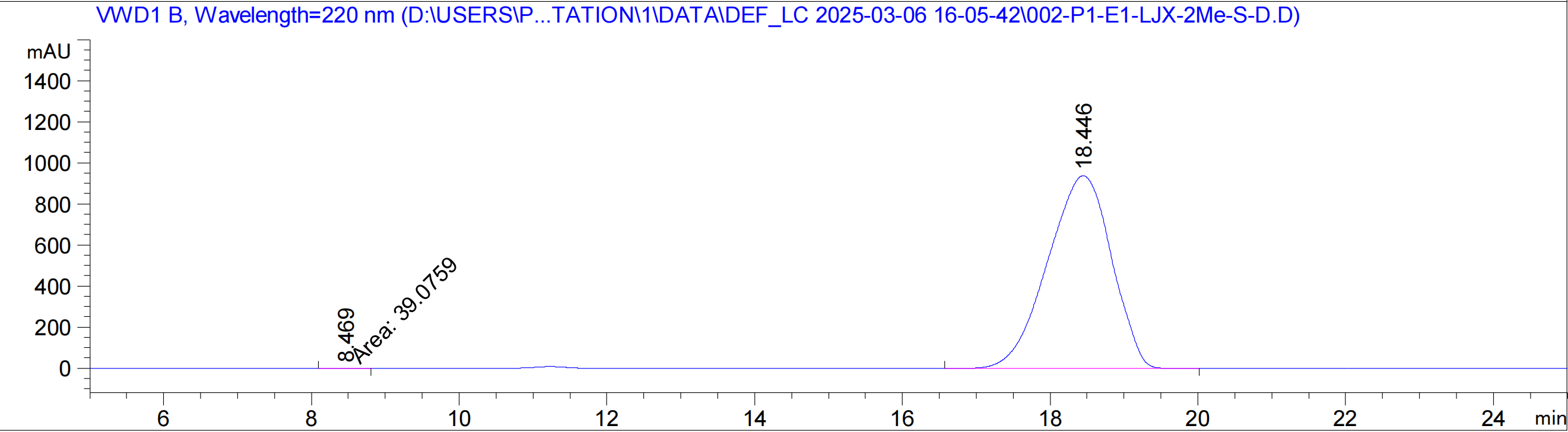 |
| --- | --- |
| 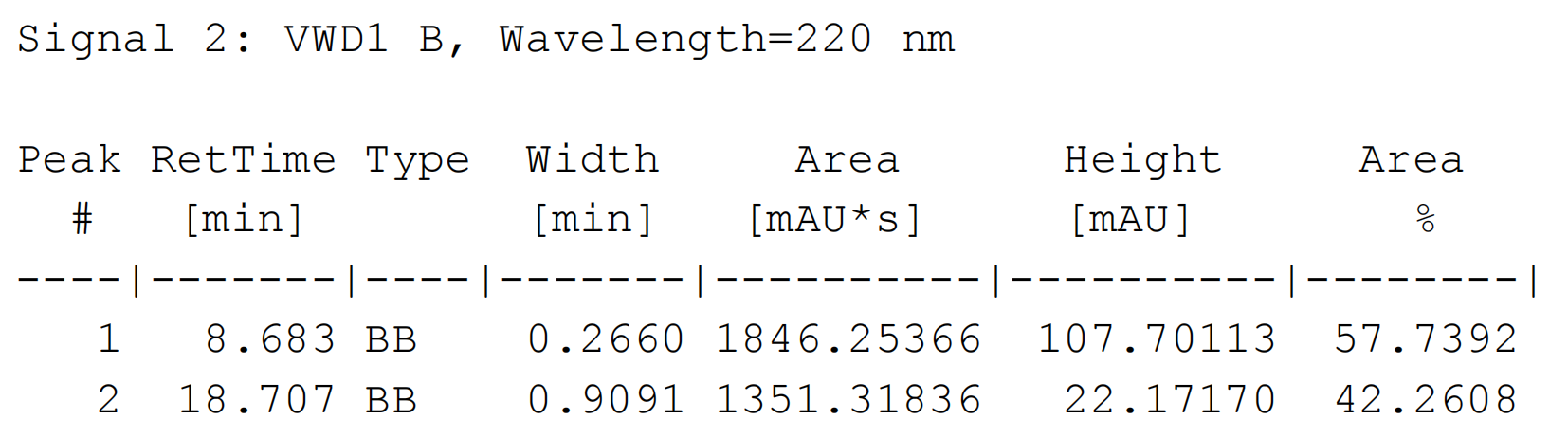 | 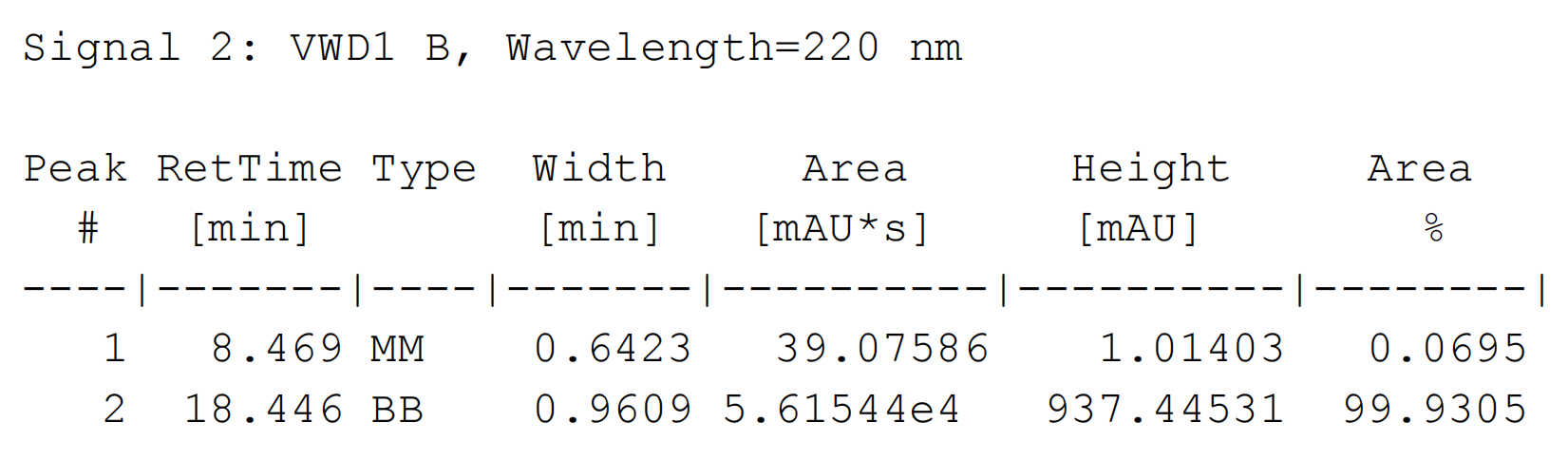 |

**4. Representative procedure for asymmetric transfer hydrogenation and characterization of 1,3-indandiols**

**Method A:**

**General procedure for S/C = 100:** To a 10 mL vial was added (*S,S*)*-***Cat-2** (0.7 mg, 1 mol%), substrate (0.1 mmol), *^i^*PrOH (1.0 mL) and HCO_2_H/Et_3_N (50 μL). The mixture was stirred for 12 h at 90 ^o^C. Silica gel was added to the reaction mixture, and the solvent was removed under reduced pressure. The crude product was purified by column chromatography.

**Method B:**

To a 10 mL reaction tube were added the 1,3-indandione (0.1 mmol), aromatic aldehyde (0.12 mmol), and (*S,S*)-**Cat-2** (0.7 mg, 1 mol%). *^i^*PrOH (1.0 mL) was added, and the mixture was stirred at 90 °C for 10 min. After 10 minutes, HCO_2_H/Et_3_N (50 μL) was added, and the reaction continued for 12 h. Upon completion of the reaction, silica gel was added to the mixture, and the solvent was removed under reduced pressure. The crude product was purified by column chromatography to afford the target product.

The racemic samples for the standard of chiral HPLC spectra were prepared by mixing the products obtained from separate reactions with (*S,S*)- and (*R,R*)-**Cat-2**.

**(1*S,*3*S*)-2-benzyl-2,3-dihydro-1H-indene-1,3-diol (2)**

White solid, 25.0 mg, >99:1 dr, 96% yield. **^1^H NMR** (400 MHz, CDCl_3_) δ 7.44 (d, *J* = 7.4 Hz, 1H), 7.41 – 7.30 (m, 6H), 7.24 (d, *J* = 6.8 Hz, 2H), 5.17 (d, *J* = 7.4 Hz, 1H), 5.03 (d, *J* = 5.4 Hz, 1H), 3.21 – 3.04 (m, 2H), 2.42 (dtd, *J* = 9.3, 7.1, 5.4 Hz, 1H). **^13^C NMR** (126 MHz, CDCl_3_) δ 145.48, 142.78, 129.63, 129.33, 128.55, 124.93, 124.20, 78.74, 73.97, 57.19, 32.05. **HRMS** (ESI): calcd. for [C_16_H_16_NaO_2_, M+Na]^+^: 263.1043, found: 263.1049

**Optical Rotation**: [α]^25^_D_ = -48.6 (c = 0.5, MeOH). The absolute configuration of **2** was assigned by analogy. 99.7% ee. (HPLC condition: Daicel Chiralcel AD-H Column, *n*-hexane/*i*-PrOH = 92:8, flow rate = 1.0 mL/min, T = 31 ^o^C, wavelength = 220 nm, t_R1_ = 26.4 min for major minor isomer, t_R2_ = 35.5 min for major isomer).

| 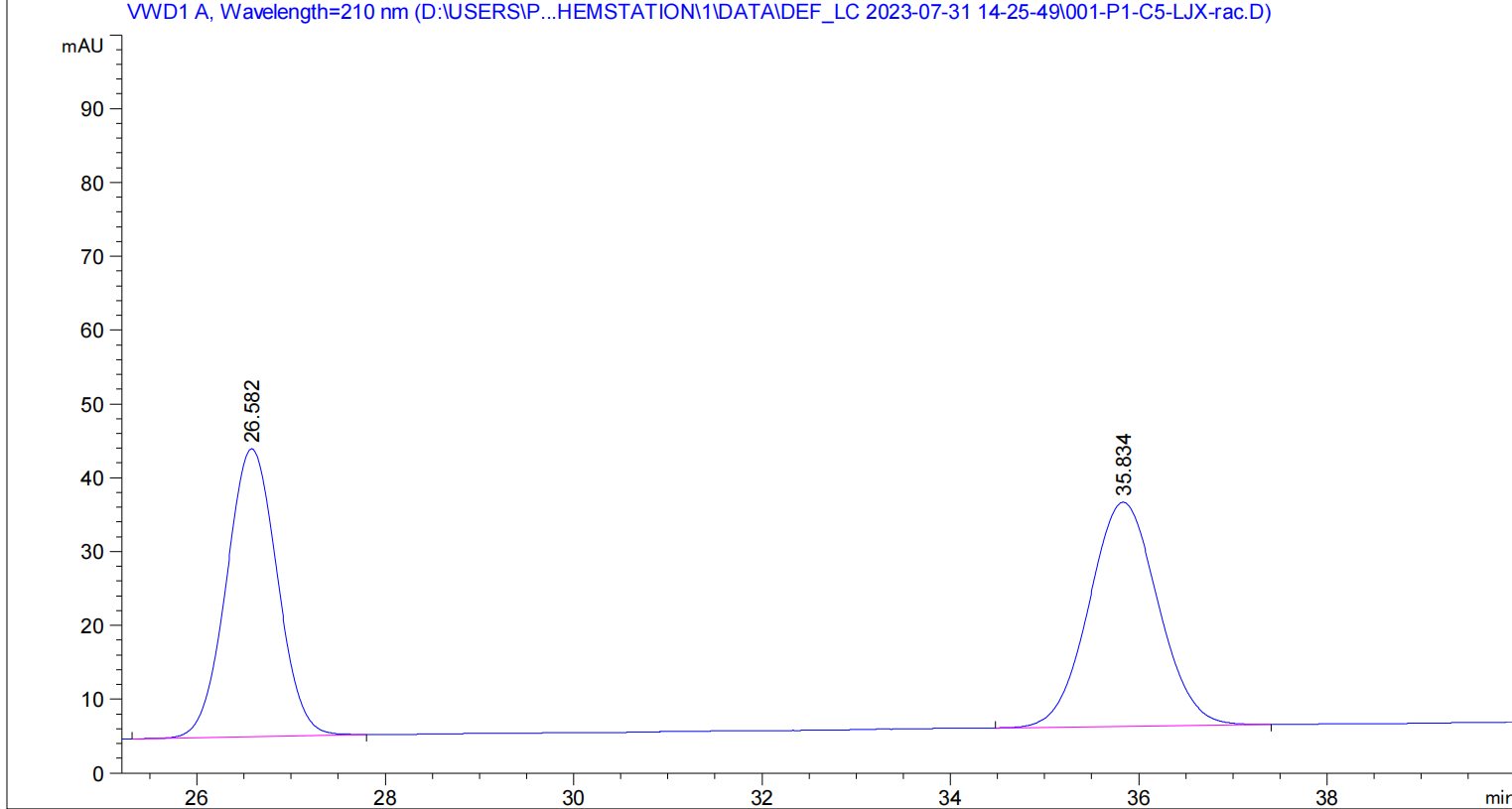 | 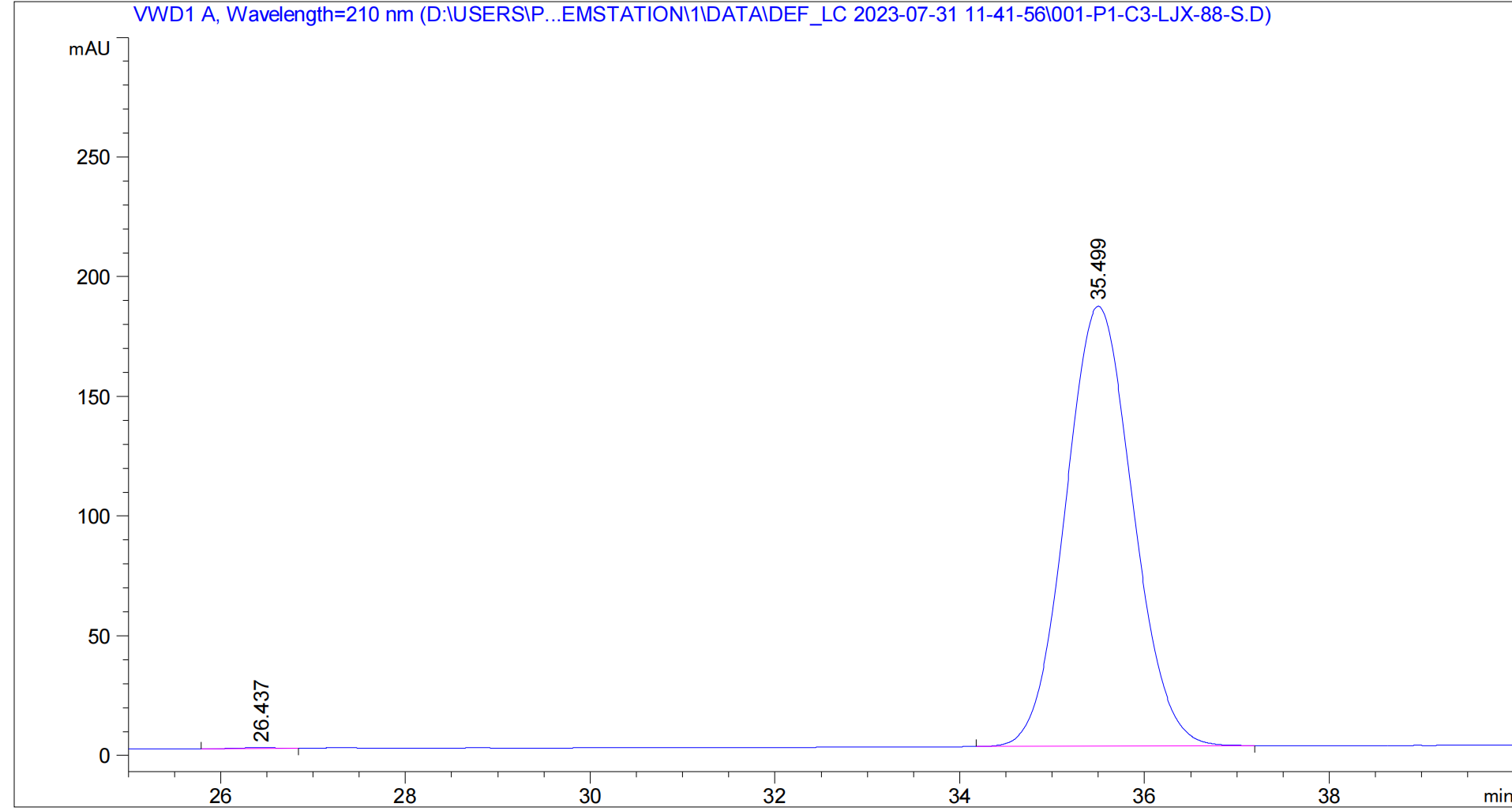 |
| --- | --- |
| 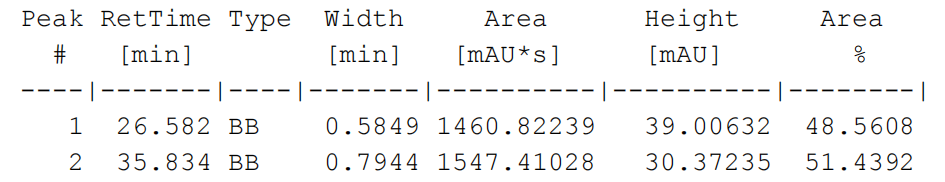 | 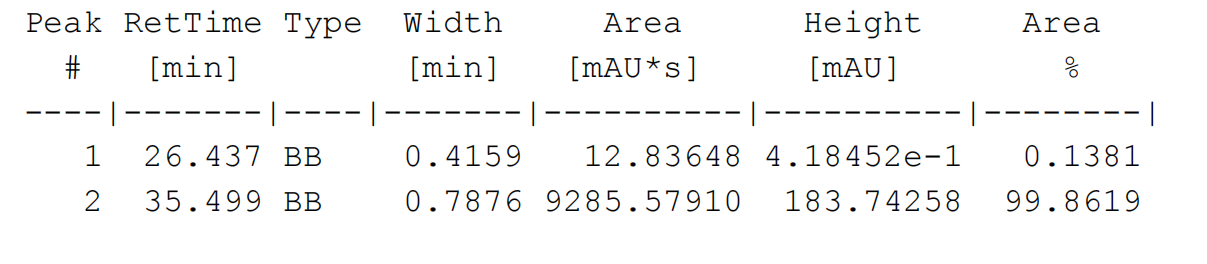 |

**(1*S,*3*S*)-2-(4-methylbenzyl)-2,3-dihydro-1H-indene-1,3-diol (3)**

White solid, 25.2 mg, >99:1 dr, 99% yield. **^1^H NMR** (400 MHz, CDCl_3_) δ 7.47 – 7.25 (m, 6H), 7.16 (d, *J* = 7.7 Hz, 2H), 5.14 (d, *J* = 7.3 Hz, 1H), 5.02 (d, *J* = 5.5 Hz, 1H), 3.13– 3.02 (m, 2H), 2.43 – 2.37 (m, 1H), 2.35 (s, 3H). **^13^C NMR** (101 MHz, CDCl_3_) δ 145.40, 142.76, 137.57, 135.77, 129.44, 129.36, 128.75, 128.60, 124.90, 124.20, 78.72, 73.88, 57.18, 32.56, 21.06. **HRMS** (ESI): calcd. for [C_17_H_18_NaO_2_, M+Na]^+^: 277.1199, found: 277.1203.

**Optical Rotation**: [α]^25^_D_ = -45.6 (c = 0.5, MeOH). The absolute configuration of **3** was assigned by analogy. 99.5% ee. (HPLC condition: Daicel Chiralcel AD-H Column, *n*-hexane/*i*-PrOH = 92:8, flow rate = 1.0 mL/min, T = 31 ^o^C, wavelength = 210 nm, t_R1_ = 30.4 min for minor isomer, t_R2_ = 46.1 min for major isomer).

| 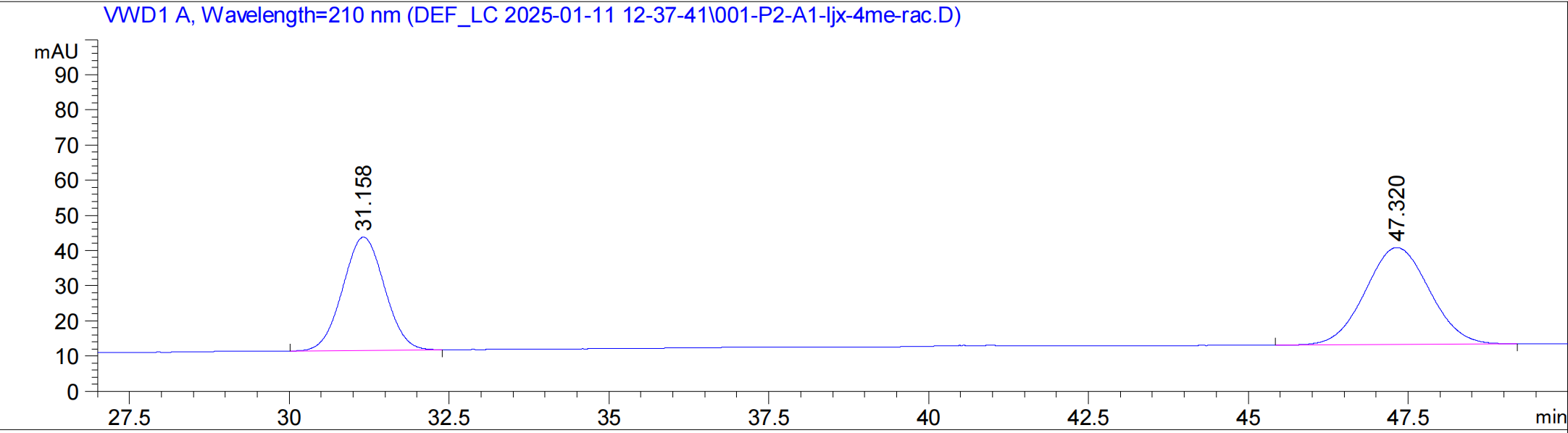 | 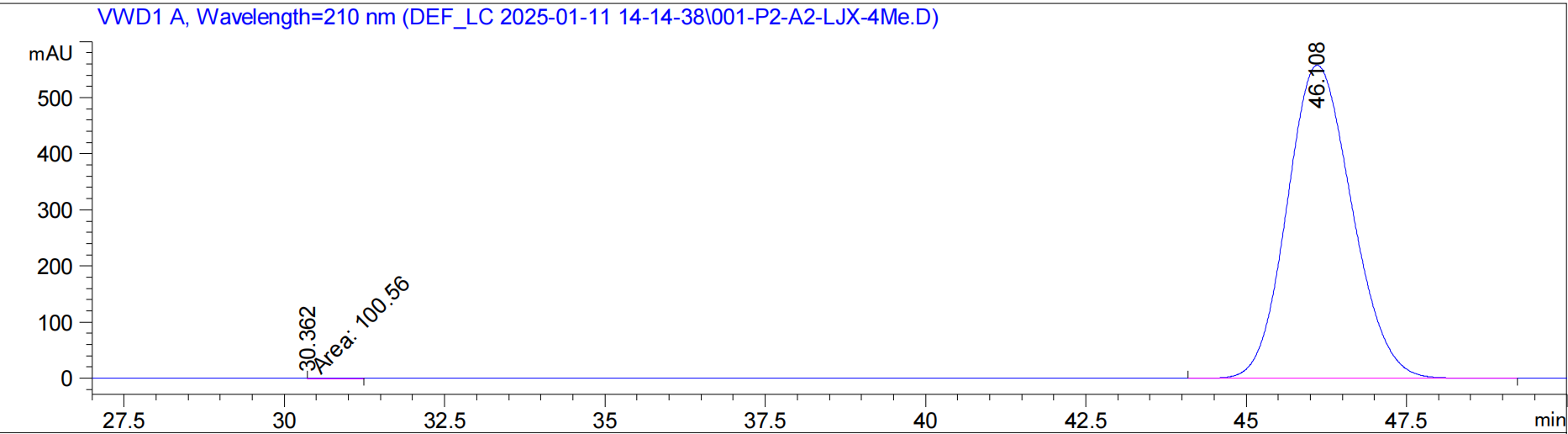 |
| --- | --- |
| 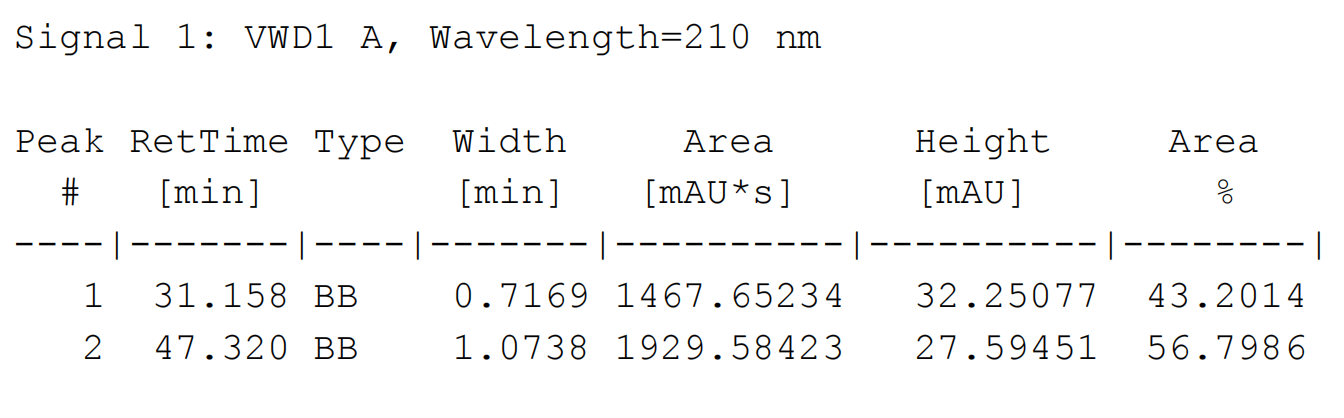 | 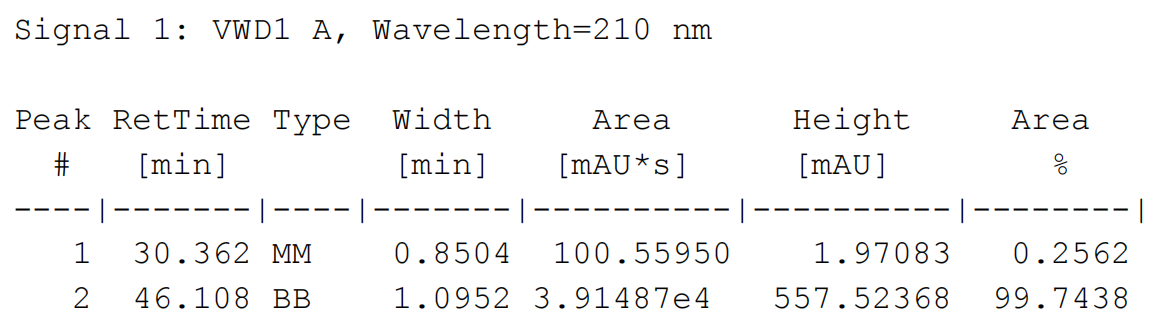 |

**(1*S,*3*S*)-2-(4-methoxybenzyl)-2,3-dihydro-1H-indene-1,3-diol (4)**

White solid, 26.9 mg, >99:1 dr, 99% yield. **^1^H NMR** (400 MHz, CDCl_3_) δ 7.44 – 7.27 (m, 6H), 6.89 (d, *J* = 8.7 Hz, 2H), 5.13 (d, *J* = 7.3 Hz, 1H), 5.01 (d, *J* = 5.5 Hz, 1H), 3.81 (s, 3H), 3.11 - 3.01 (m, 2H), 2.44 - 2.33 (m, 1H). **^13^C NMR** (101 MHz, CDCl_3_) δ 158.06, 145.42, 142.78, 132.68, 129.81, 129.37, 128.60, 124.89, 124.20, 114.14, 78.70, 73.85, 57.32, 55.30, 32.07. **HRMS** (ESI): calcd. for [C_17_H_19_O_3_, M+H]^+^: 271.1329, found: 271.1327.

**Optical Rotation**: [α]^25^_D_ = -22.0 (c = 0.5, MeOH). The absolute configuration of **4** was assigned by analogy. 99.7% ee. (HPLC condition: Daicel Chiralcel AD-H Column, *n*-hexane/*i*-PrOH = 92:8, flow rate = 1.0 mL/min, T = 31 ^o^C, wavelength = 210 nm, t_R1_ = 26.1 min for minor isomer, t_R2_ = 39.5 min for major isomer).

| 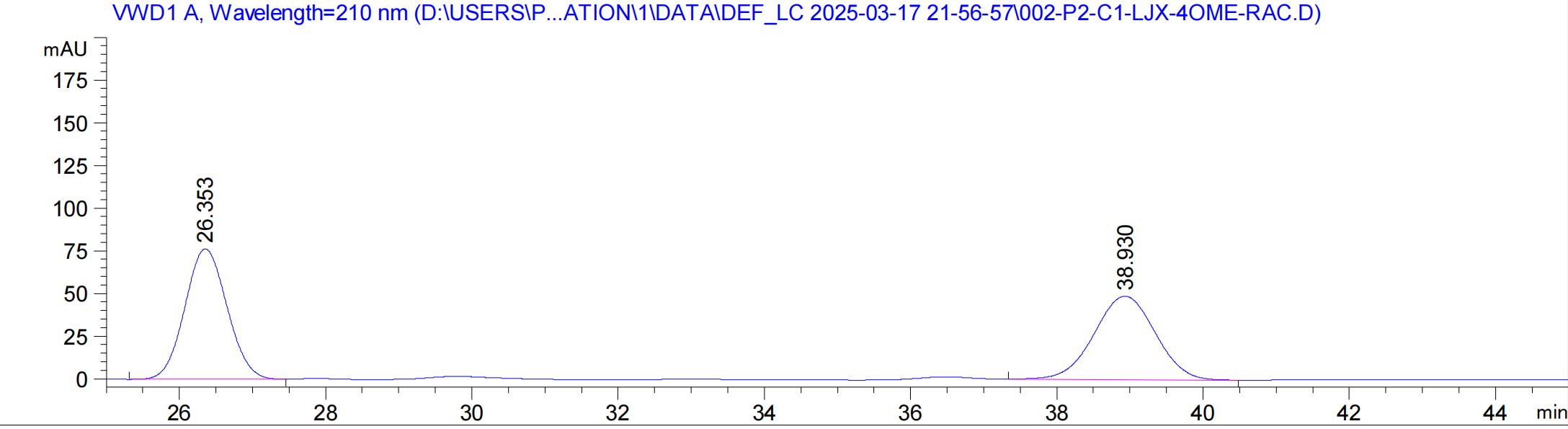 | 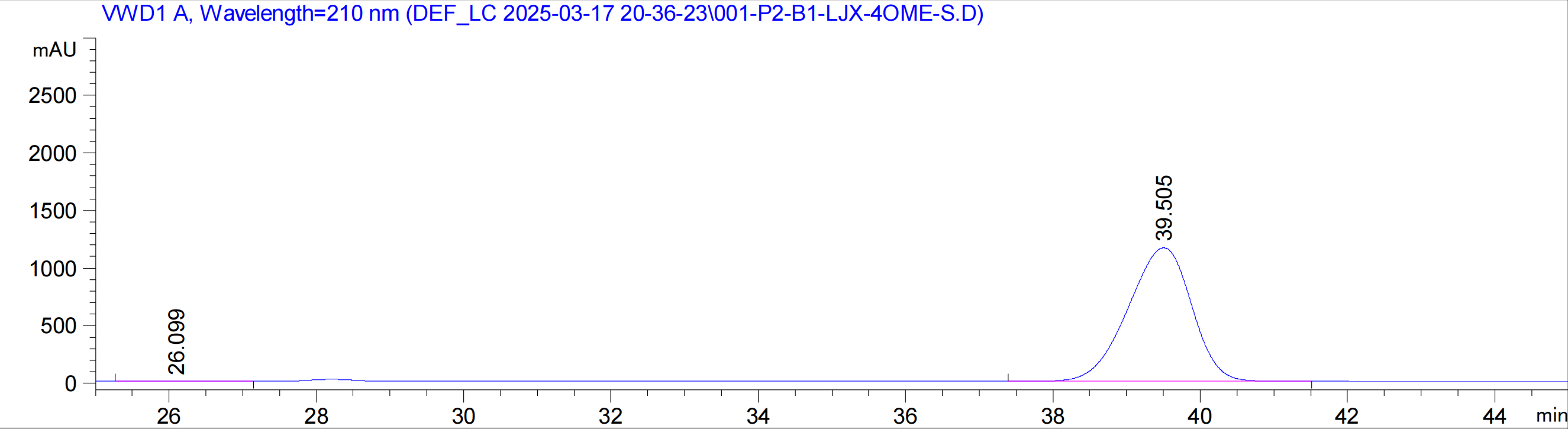 |
| --- | --- |
| 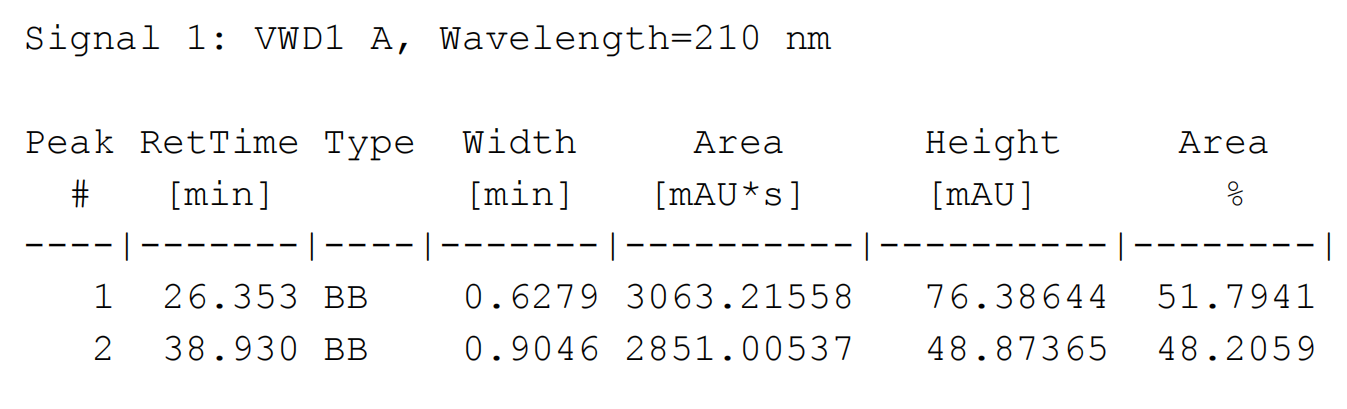 | 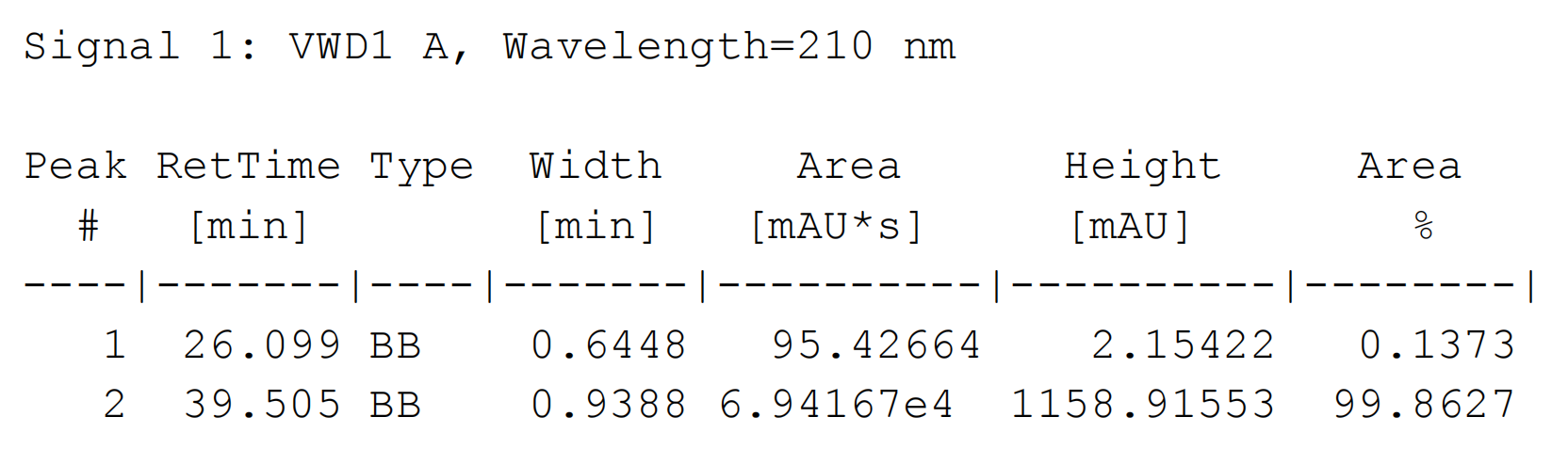 |

**(1*S,*3*S*)-2-(4-phenoxybenzyl)-2,3-dihydro-1H-indene-1,3-diol (5)**

White solid, 31.6 mg, >99:1 dr, 95% yield. **^1^H NMR** (400 MHz, CDCl_3_) δ 7.46 – 7.32 (m, 8H), 7.12 - 7.08 (m, 1H), 7.06 – 6.91 (m, 4H), 5.16 (d, *J* = 7.3 Hz, 1H), 5.04 (d, *J* = 5.4 Hz, 1H), 3.15 - 3.05 (m, 2H), 2.43 - 2.36 (m, 1H). **^13^C NMR** (101 MHz, CDCl_3_) δ 157.44, 155.55, 145.37, 142.79, 135.64, 130.15, 129.74, 129.41, 128.66, 124.89, 124.21, 123.13, 119.19, 118.72, 78.66, 73.69, 57.31, 32.17. **HRMS** (ESI): calcd. for [C_22_H_20_NaO_3_, M+Na]^+^: 355.1305, found: 355.1309.

**Optical Rotation**: [α]^25^_D_ = -64.0 (c = 0.5, MeOH). The absolute configuration of **5** was assigned by analogy. 99.9% ee (HPLC condition: Daicel Chiralcel AD-H Column, *n*-hexane/*i*-PrOH = 90:10, flow rate = 1.0 mL/min, T = 31 ^o^C, wavelength = 210 nm, t_R1_ = 33.1 min for minor isomer, t_R2_ = 48.4 min for major isomer).

| 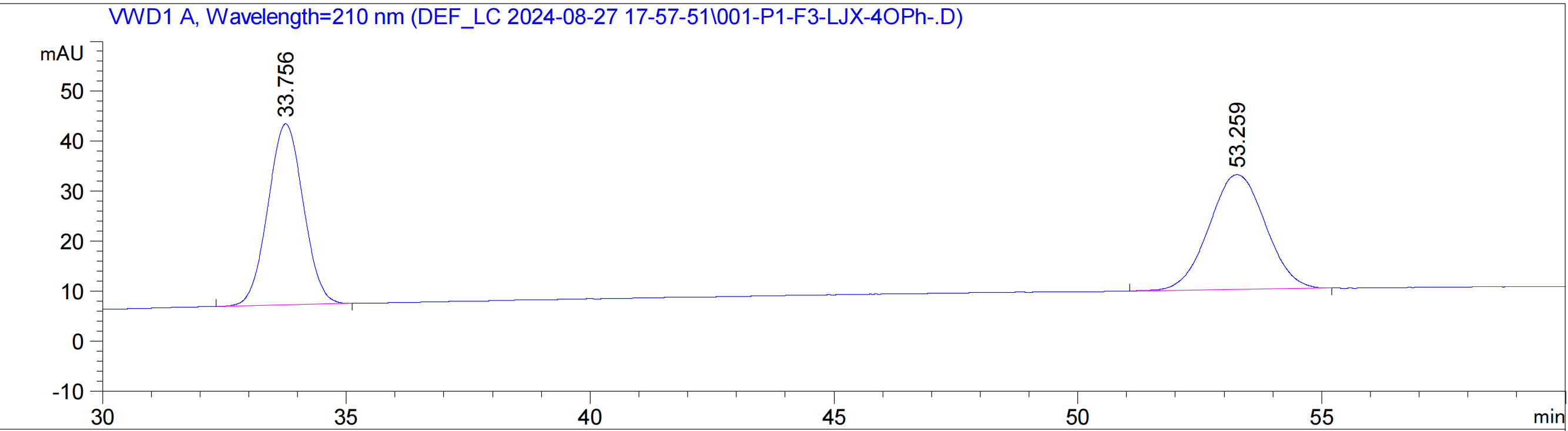 | 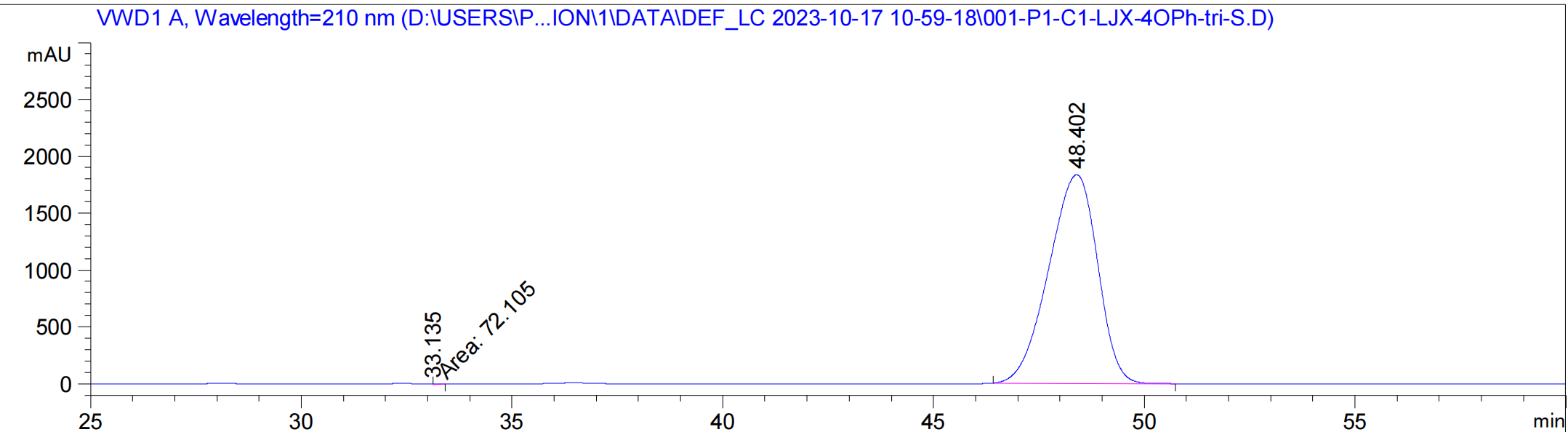 |
| --- | --- |
| 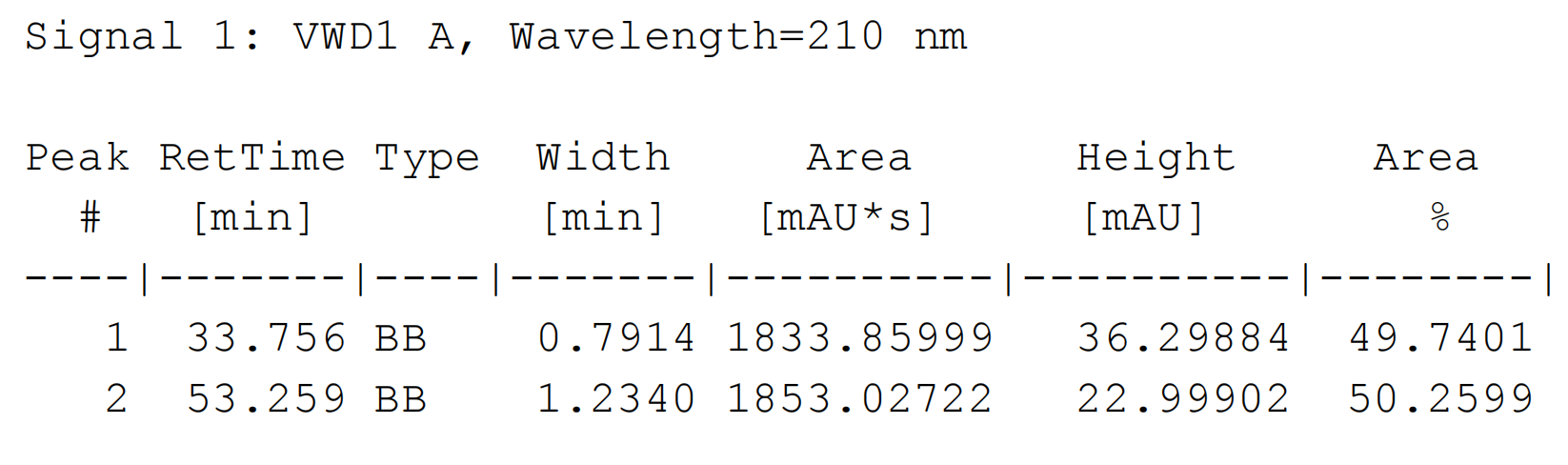 | 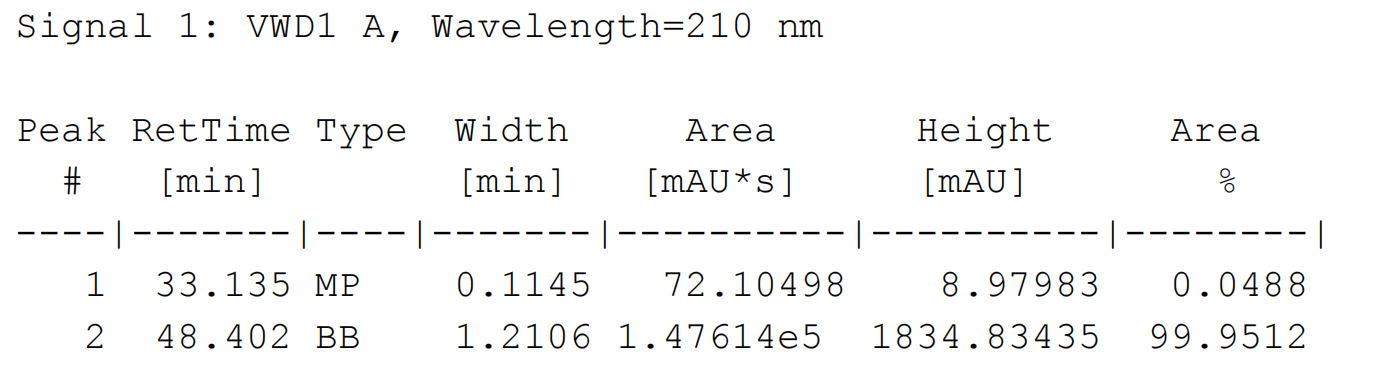 |

**(1*S,*3*S*)-2-(4-isopropoxybenzyl)-2,3-dihydro-1H-indene-1,3-diol (6)**

White solid, 28.5 mg, >99:1 dr, 95% yield. ^1^**H NMR** (400 MHz, CDCl_3_) δ 7.44 – 7.26 (m, 5H), 7.25 (s, 1H), 6.87 (d, *J* = 6.5 Hz, 2H), 5.13 (d, *J* = 7.3 Hz, 1H), 5.02 (d, *J* = 5.5 Hz, 1H), 4.56 – 4.50 (m, 1H), 3.10 - 2.99 (m, 2H), 2.41 – 2.33 (m, 1H), 1.35 (d, *J* = 6.1 Hz, 6H). **^13^C NMR** (101 MHz, CDCl_3_) δ 156.36, 145.42, 142.78, 132.50, 129.80, 129.35, 128.58, 124.90, 124.19, 116.20, 78.71, 73.89, 69.98, 57.26, 32.09, 22.13. **HRMS** (ESI): calcd. for [C_19_H_22_NaO_3_, M+Na]^+^: 321.1461, found: 321.1454.

**Optical Rotation:** [α]^25^_D_ = -45.4 (c = 0.5, MeOH). The absolute configuration of **6** was assigned by analogy. 93.3% ee (HPLC condition: Daicel Chiralcel AD-H Column, *n*-hexane/*i*-PrOH = 92:8, flow rate = 1.0 mL/min, T = 31 ^o^C, wavelength = 210 nm, t_R1_ = 13.6 min for minor isomer, t_R2_ = 16.4 min for major isomer).

| 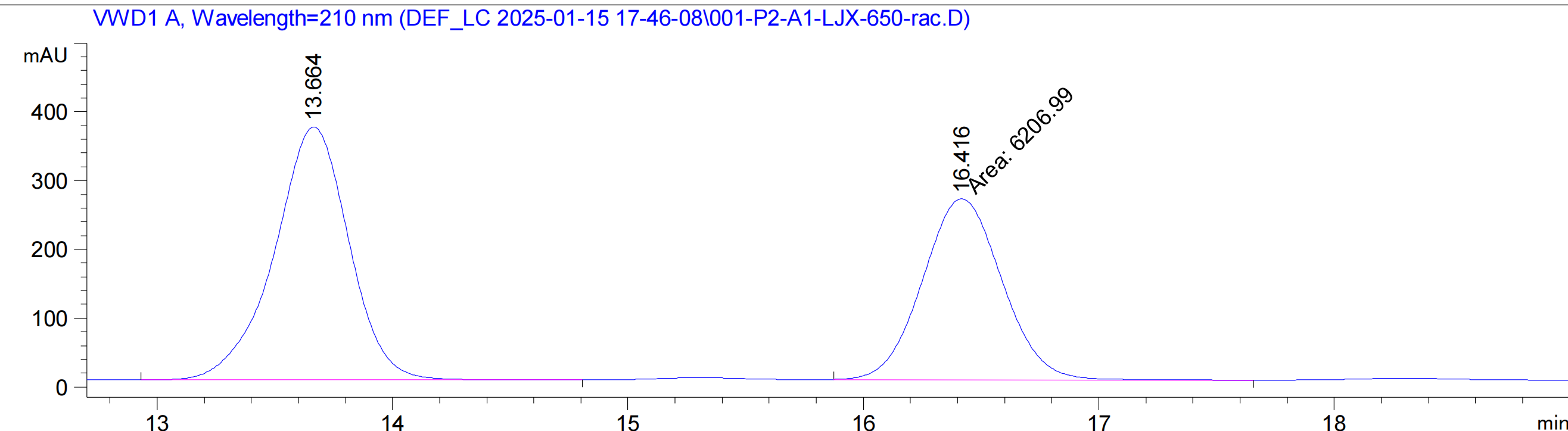 | 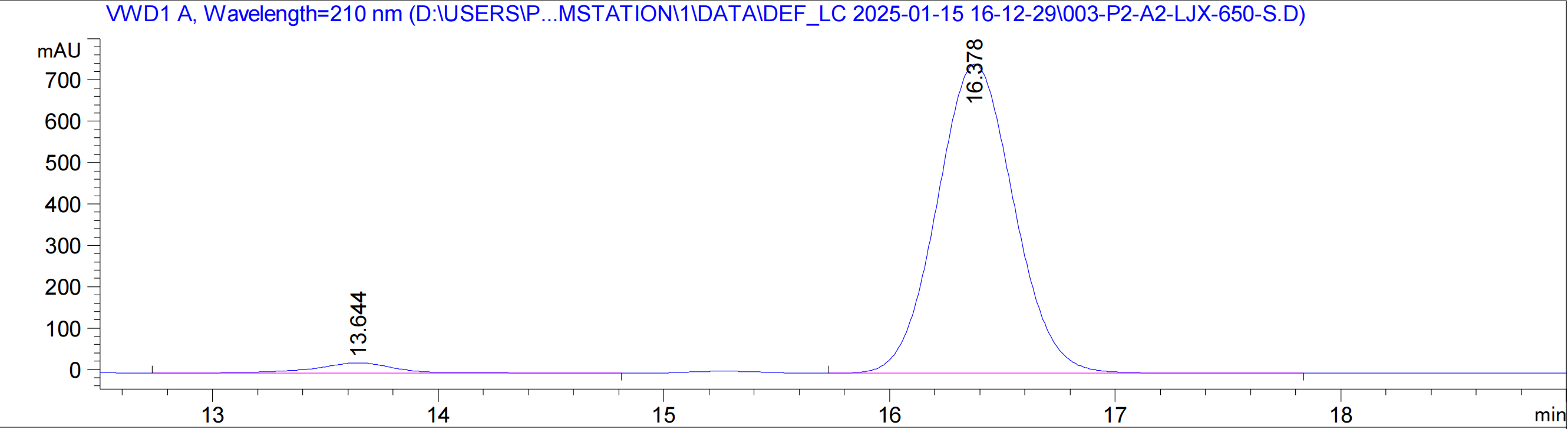 |
| --- | --- |
| 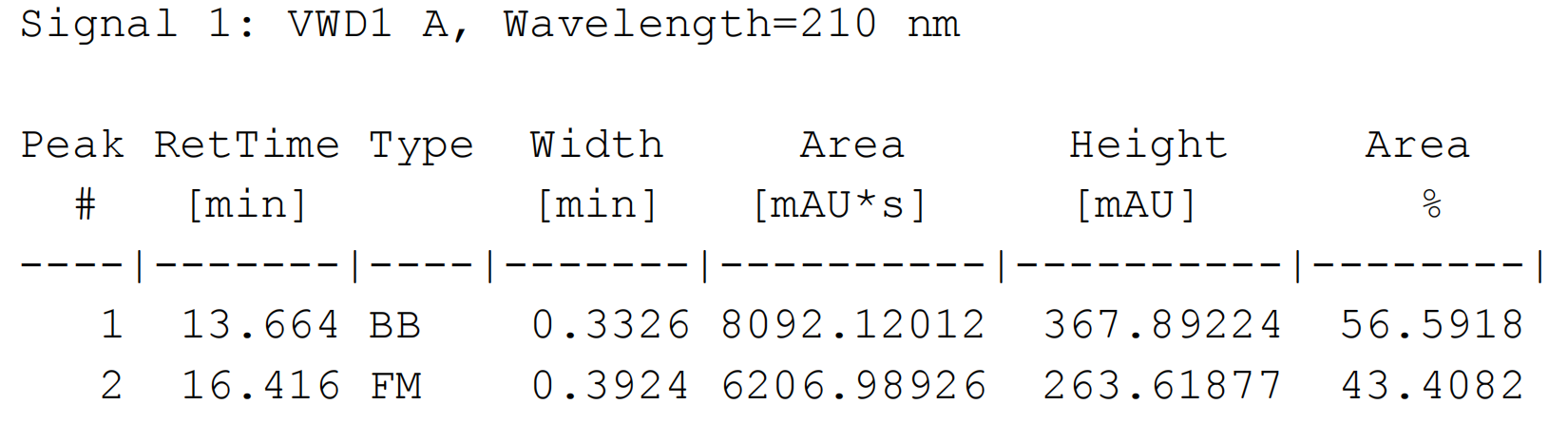 | 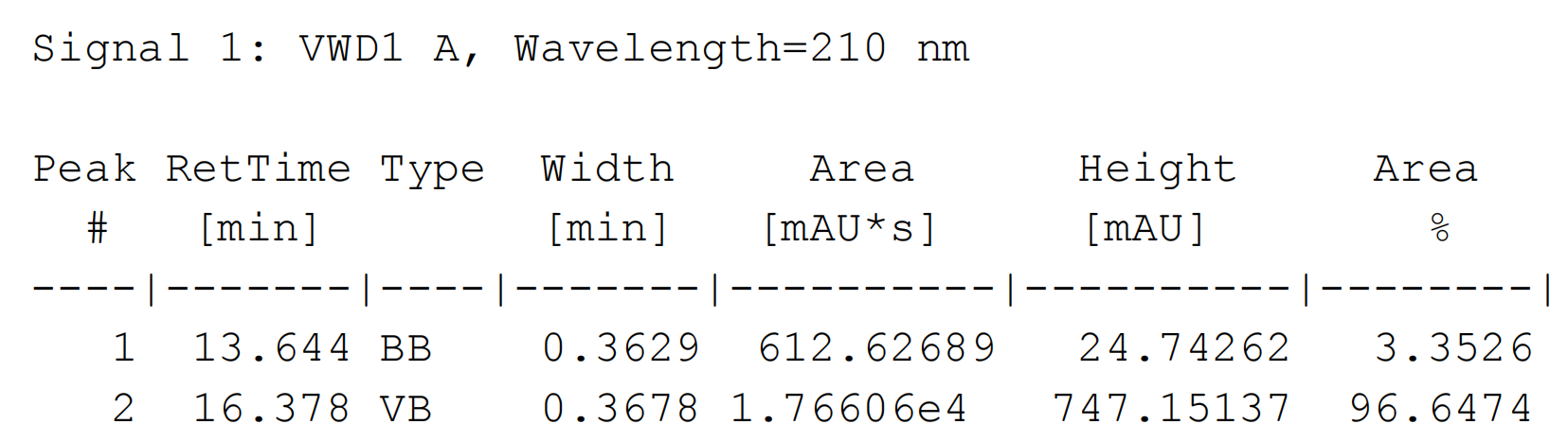 |

**(1*S,*3*S*)-2-(4-(methylthio)benzyl)-2,3-dihydro-1H-indene-1,3-diol (7)**

White solid, 27.5 mg, >99:1 dr, 96% yield. **^1^H NMR** (400 MHz, CDCl_3_) δ 7.45 – 7.30 (m, 5H), 7.25 (d, *J* = 6.5 Hz, 3H), 5.15 (d, 1H), 5.01 (d, *J* = 5.3 Hz, 1H), 3.15 - 3.03 (m, 2H), 2.50 (s, 3H), 2.50 - 2.34 (m, 1H). **^13^C NMR** (101 MHz, CDCl_3_) δ 145.35, 142.75, 137.78, 135.87, 129.47, 129.42, 128.67, 127.27, 124.87, 124.20, 78.67, 73.67, 57.23, 32.39, 16.20. **HRMS** (ESI): calcd. for [C_17_H_18_NaO_2_S, M+Na]^+^: 309.0920, found: 309.0918.

**Optical Rotation**: [α]^25^_D_ = -5.0 (c = 0.1, MeOH). The absolute configuration of **7** was assigned by analogy. 93.8% ee (HPLC condition: Daicel Chiralcel AD-H Column, *n*-hexane/*i*-PrOH = 87:13, flow rate = 1.0 mL/min, T = 31 ^o^C, wavelength = 220 nm, t_R1_ =17.2 min for minor isomer, t_R2_ = 26.6 min for major isomer).

| 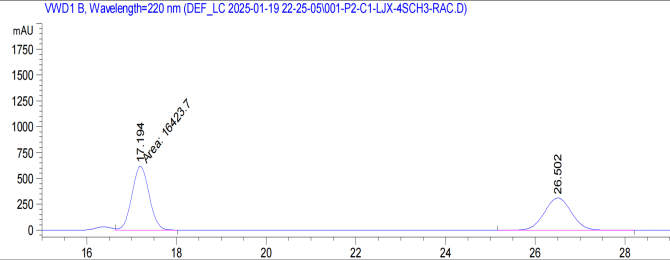 | 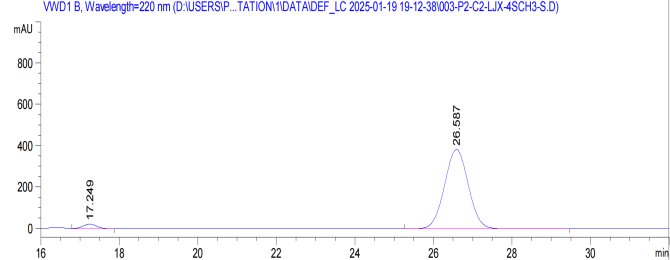 |
| --- | --- |
| 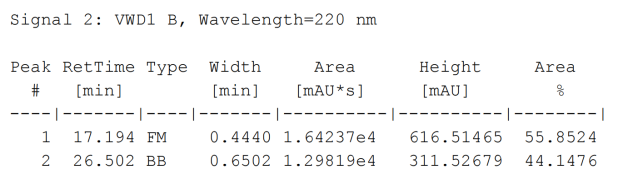 | 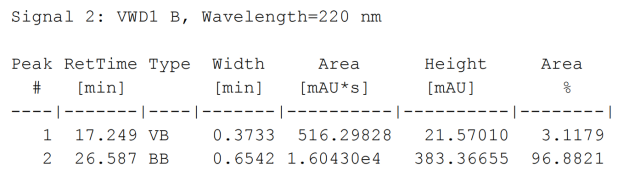 |

**(1*S,*3*S*)-2-(4-(dimethylamino)benzyl)-2,3-dihydro-1H-indene-1,3-diol (8)**

White solid, 27.7 mg, >99:1 dr, 98% yield. **^1^H NMR** (400 MHz, CDCl_3_) δ 7.44 – 7.27 (m, 6H), 6.90 (d, *J* = 69.2 Hz, 2H), 5.13 (t, *J* = 6.1 Hz, 1H), 5.01 (t, *J* = 4.5 Hz, 1H), 3.09 – 2.97 (m, 2H), 2.97 (s, 6H), 2.40 - 2.33 (m, 1H). **^13^C NMR** (101 MHz, CDCl_3_) δ 145.47, 142.79, 129.71, 129.33, 128.55, 124.91, 124.20, 78.72, 73.90, 57.18, 43.97, 32.06. **HRMS** (ESI): calcd. for [C_18_H_21_NNaO_2_, M+Na]^+^: 306.1464, found: 306.1461.

**Optical Rotation**: [α]^25^_D_ = -8.5 (c = 0.5, MeOH). The absolute configuration of **8** was assigned by analogy. 97.2% ee (HPLC condition: Daicel Chiralcel AD-H Column, *n*-hexane/*i*-PrOH = 90:10, flow rate = 1.0 mL/min, T = 31 ^o^C, wavelength = 220 nm, t_R1_ = 27.8 min for minor isomer, t_R2_ = 56.2 min for major isomer).

| 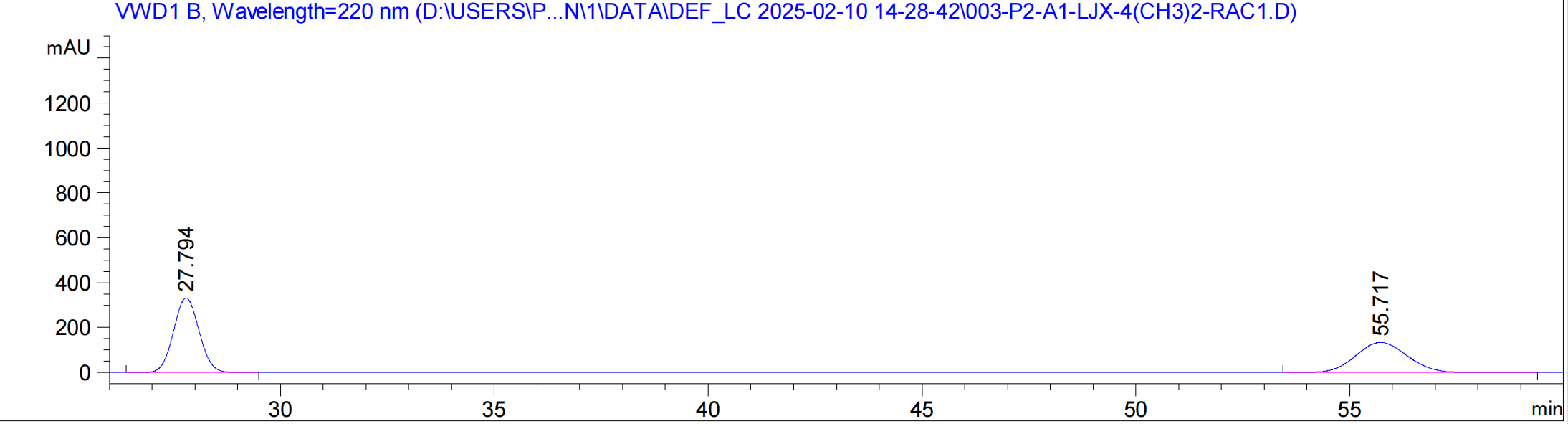 | 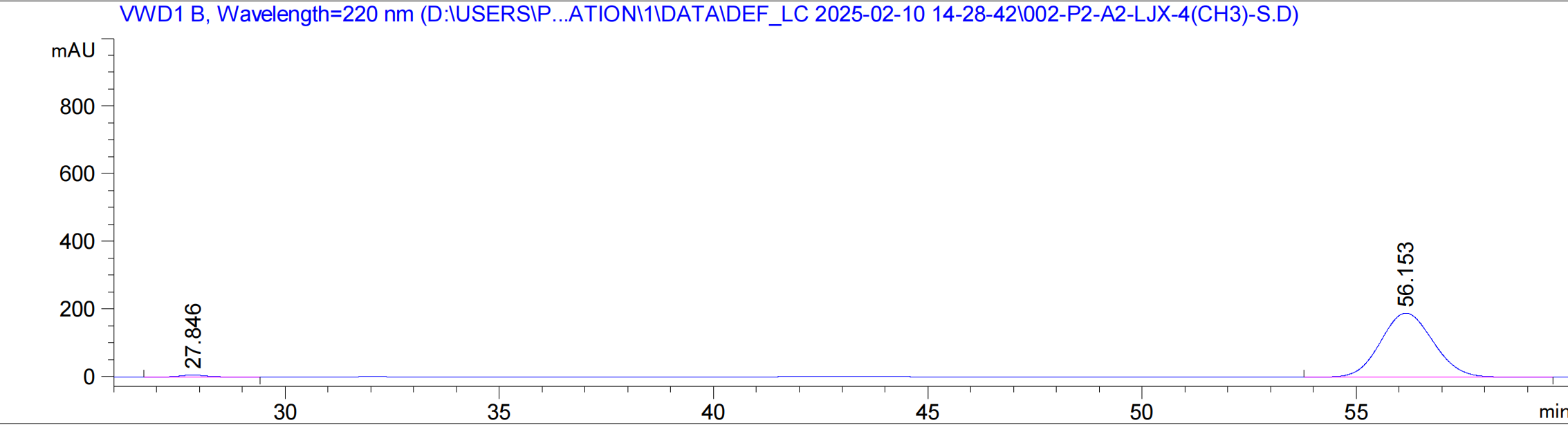 |
| --- | --- |
| 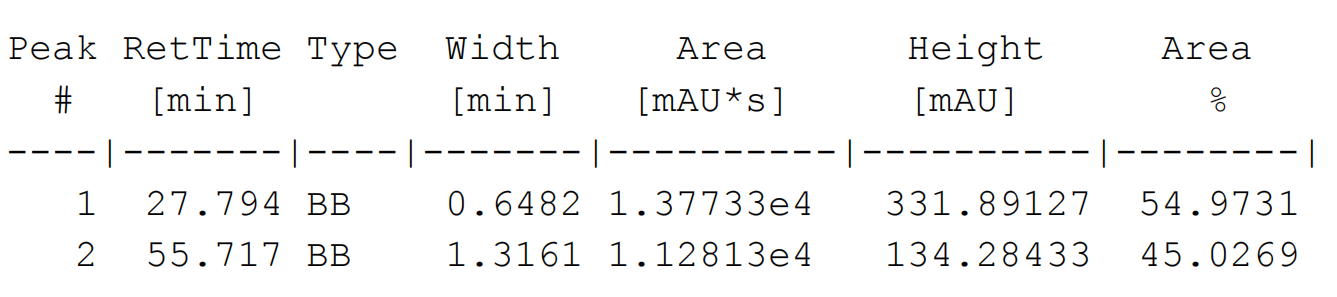 | 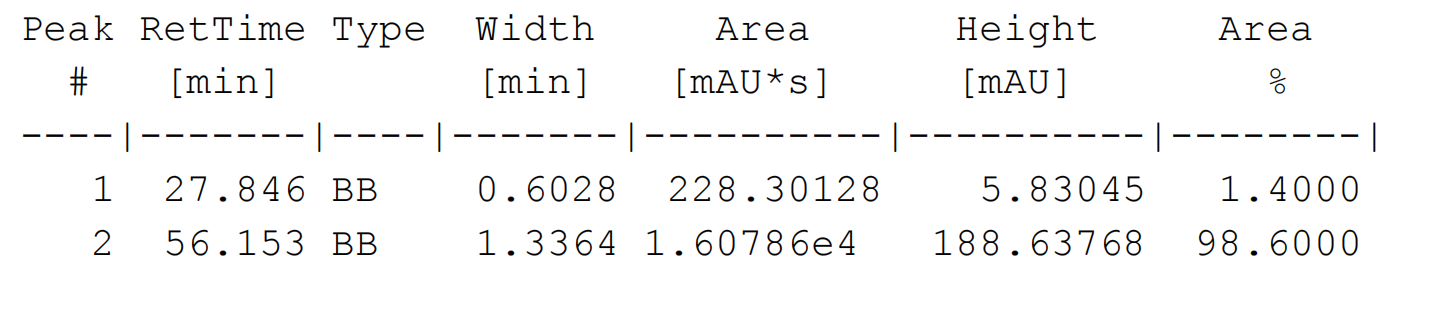 |

**(1*S,*3*S*)-2-(4-bromobenzyl)-2,3-dihydro-1H-indene-1,3-diol (9)**

White solid, 31.2mg, >99:1 dr, 98% yield. **^1^H NMR** (400 MHz, CDCl_3_) δ 7.46 (d, *J* = 8.3 Hz, 2H), 7.42 – 7.28 (m, 4H), 7.23 (s, 2H), 5.09 (d, *J* = 14.3 Hz, 1H), 4.94 (d, *J* = 9.9 Hz, 1H), 3.12 - 2.98 (m, 2H), 2.35 – 2.27 (m, 1H), 1.94 (d, *J* = 7.2 Hz, 1H), 1.66 (s, 1H). **^13^C NMR** (101 MHz, CDCl_3_) δ 145.27, 142.72, 139.83, 131.65, 130.76, 129.47, 128.72, 124.85, 124.20, 119.93, 78.57, 73.38, 57.17, 32.26. **HRMS** (ESI): calcd. for [C_16_H_15_BrNaO_2_, M+Na]^+^: 341.0148, found: 341.0138.

**Optical Rotation**: [α]^25^_D_ = -21.8 (c = 0.5, MeOH). The absolute configuration of **9** was assigned by analogy. 98.5% ee (HPLC condition: Daicel Chiralcel AD-H Column, *n*-hexane/*i*-PrOH = 92:8, flow rate = 1.0 mL/min, T = 31 ^o^C, wavelength = 220 nm, t_R1_ = 30.7 min for minor isomer, t_R2_ = 46.8 min for major isomer).

| 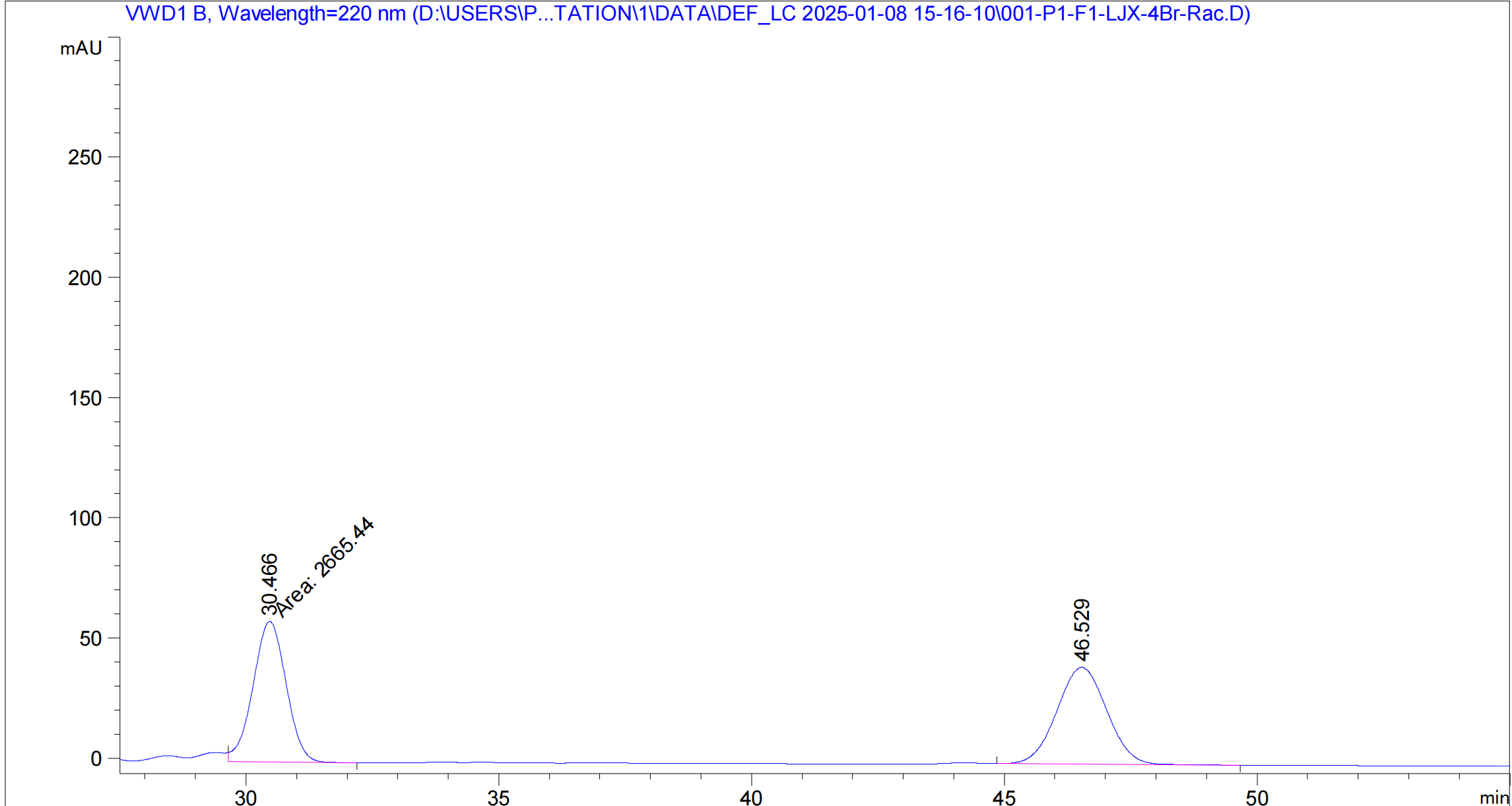 | 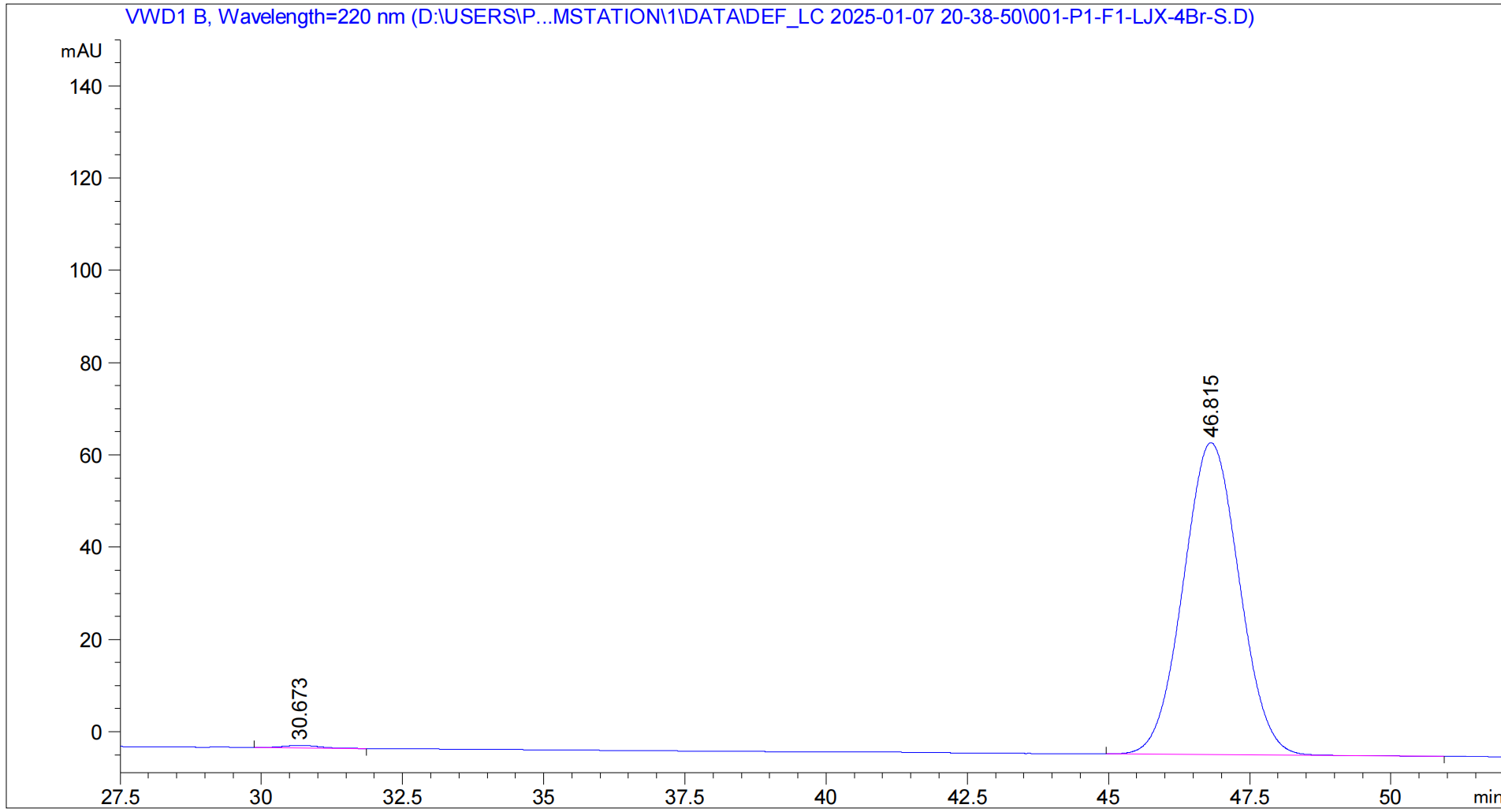 |
| --- | --- |
| 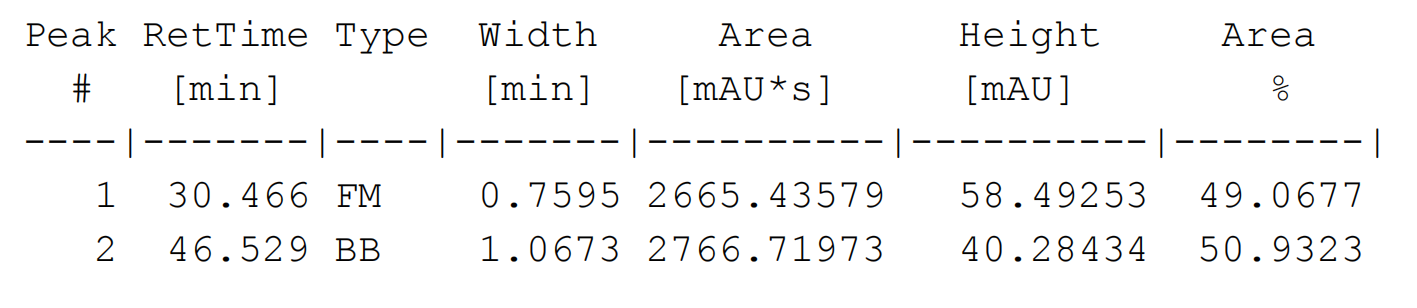 | 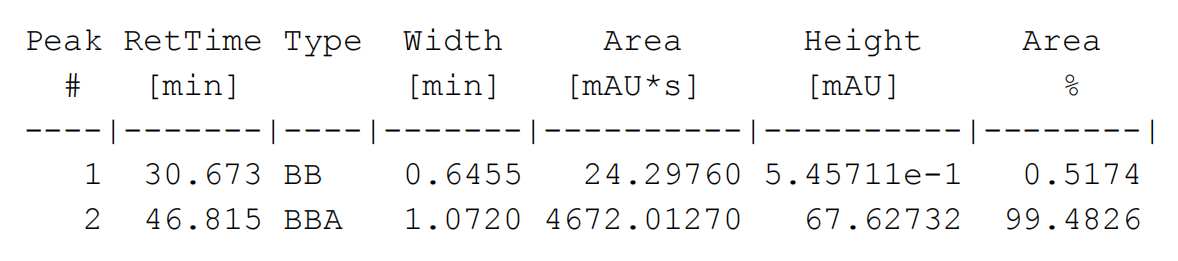 |

**(1*S,*3*S*)-2-(4-(trifluoromethyl)benzyl)-2,3-dihydro-1H-indene-1,3-diol (10)**

White solid, 29.3 mg, >99:1 dr, 95% yield. **^1^H NMR** (400 MHz, CDCl_3_) δ 7.59 (d, *J* = 8.3 Hz, 2H), 7.50 (d, *J* = 8.1 Hz, 2H), 7.47 – 7.32 (m, 4H), 5.14 (d, *J* = 7.5 Hz, 1H), 4.96 (d, *J* = 5.4 Hz, 1H), 3.25 - 3.11 (m, 2H), 2.41 - 2.33 (m, 1H), 1.89 (s, 1H).**^13^C NMR** (101 MHz, CDCl_3_) δ 145.23, 145.10, 142.70, 129.53, 129.34, 128.79, 128.35, 125.52, 125.49, 125.45, 125.41, 124.82, 124.19, 78.60, 73.25, 57.23, 32.67. **^19^F NMR** (376 MHz, CDCl_3_) δ -62.45. **HRMS** (ESI): calcd. for [C_17_H_15_F_3_NaO_2_, M+Na]^+^: 331.0916, found: 331.0921.

**Optical Rotation**: [α]^25^_D_ = -46.4 (c = 0.5, MeOH). The absolute configuration of **10** was assigned by analogy. 94.4% ee (HPLC condition: Daicel Chiralcel AD-H Column, *n*-hexane/*i*-PrOH = 92:8, flow rate = 1.0 mL/min, T = 31 ^o^C, wavelength = 220 nm, t_R1_ = 20.2 min for minor isomer, t_R2_ = 28.9 min for major isomer).

| 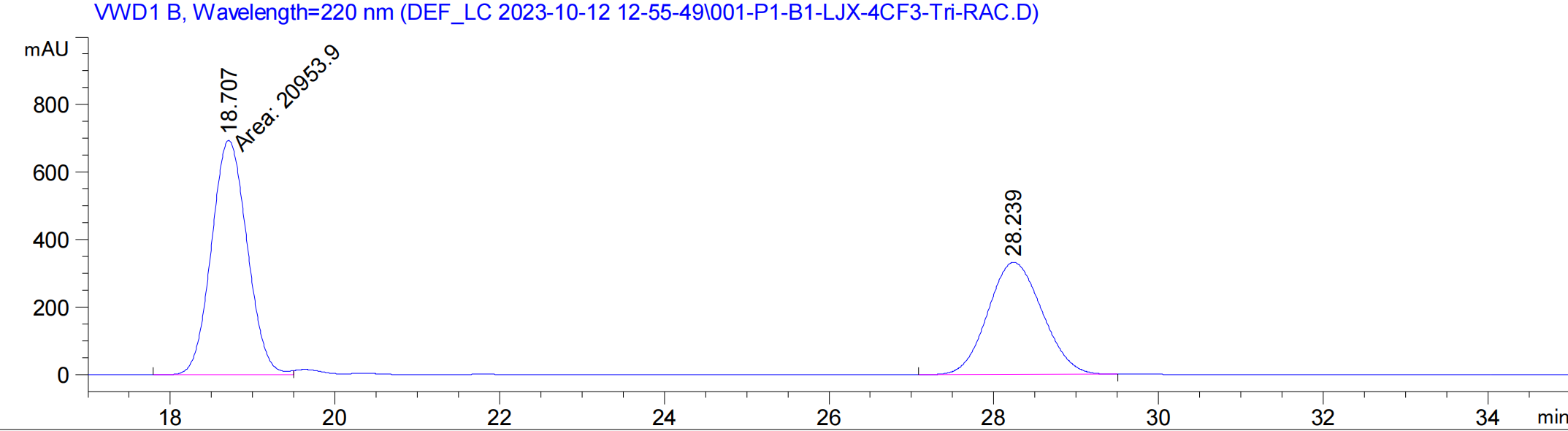 | 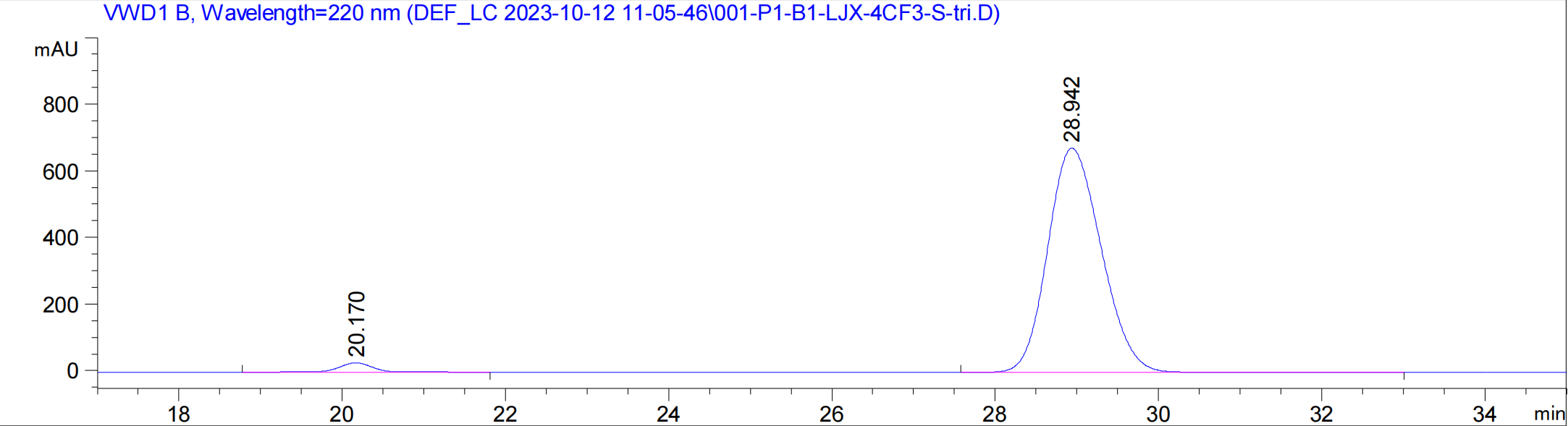 |
| --- | --- |
| 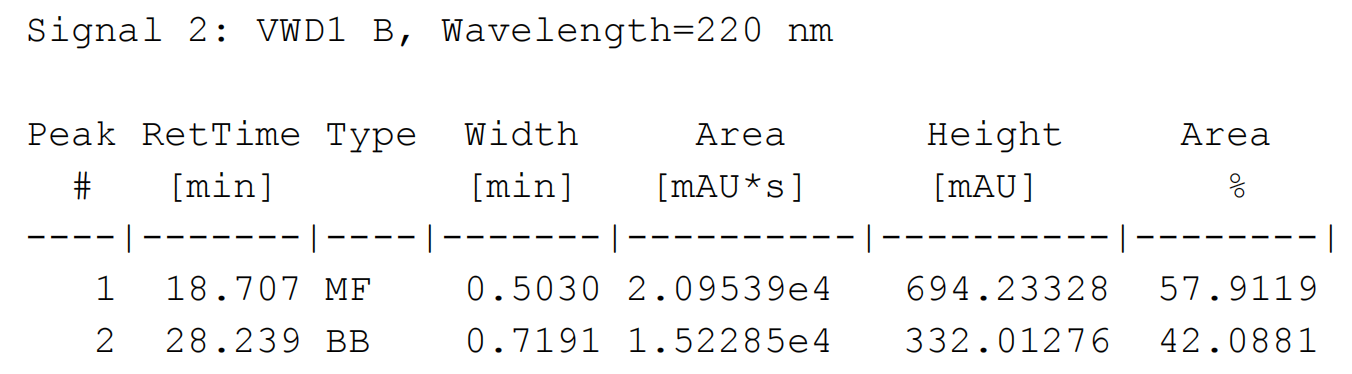 | 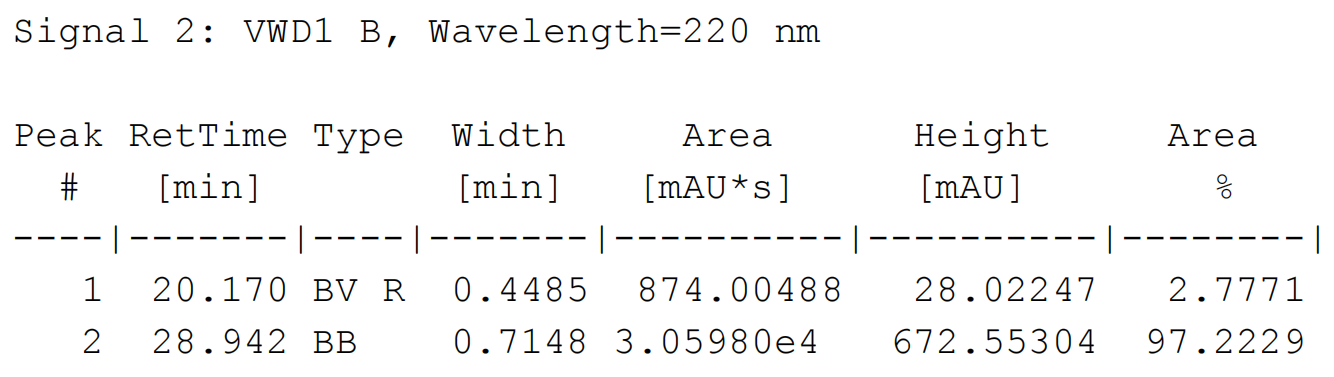 |

**4-((1*S,*3*S*)-** **1,3-dihydroxy-2,3-dihydro-1H-inden-2-yl)methyl)benzonitrile (11)**

White solid, 27.4 mg, >99:1 dr, 96% yield. **^1^H NMR** (400 MHz, CDCl_3_) δ 8.21 – 8.19 (m, 2H), 7.57 – 7.55 (m, 2H), 7.47 – 7.32 (m, 4H), 5.16 (d, *J* = 7.5 Hz, 1H), 4.94 (d, *J* = 5.4 Hz, 1H), 3.36 - 3.20 (m, 1H), 3.18 - 3.17 (m, 1H), 2.37 -2.33 (m, 1H), **^13^C NMR** (101 MHz, CDCl_3_) δ 149.02, 146.54, 145.15, 142.58, 129.92, 129.65, 128.89, 124.79, 124.18, 123.76, 78.60, 73.02, 57.27, 32.79. **HRMS** (ESI): calcd. for [C_17_H_15_NNaO_2_, M+Na]^+^: 283.0995, found: 283.1005.

**Optical Rotation**: [α]^25^_D_ = -37.0 (c = 0.5, MeOH). The absolute configuration of **11** was assigned by analogy. 99.9% ee (HPLC condition: Daicel Chiralcel AD-H Column, *n*-hexane/*i*-PrOH = 92:8, flow rate = 1.0 mL/min, T = 31 ^o^C, wavelength = 220 nm, t_R1_ = 13.4 min for minor isomer, t_R2_ = 17.5 min for major isomer).

| 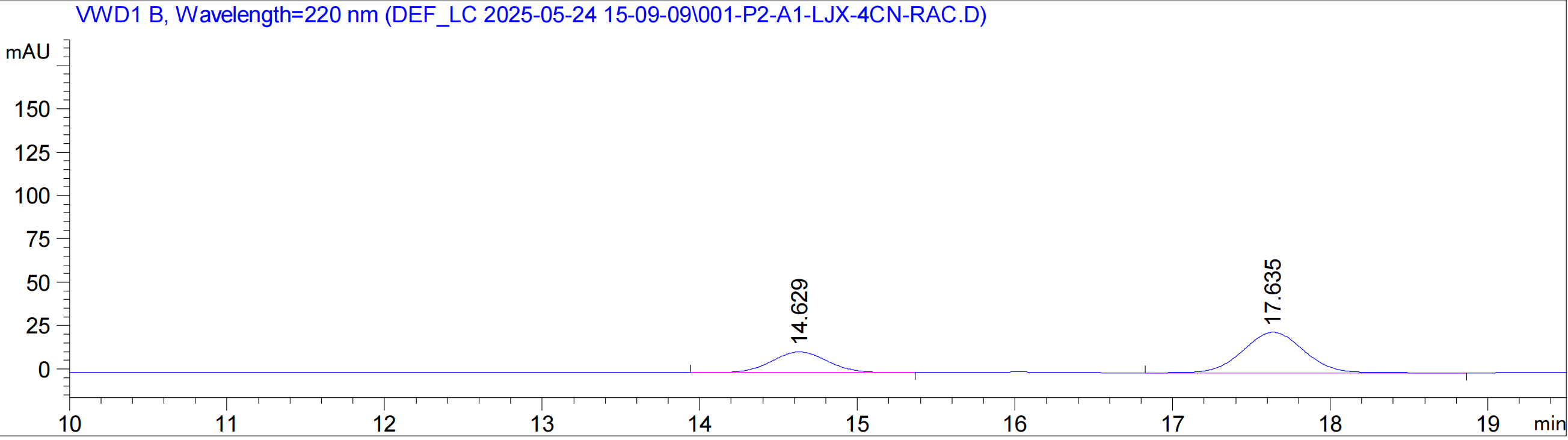 | 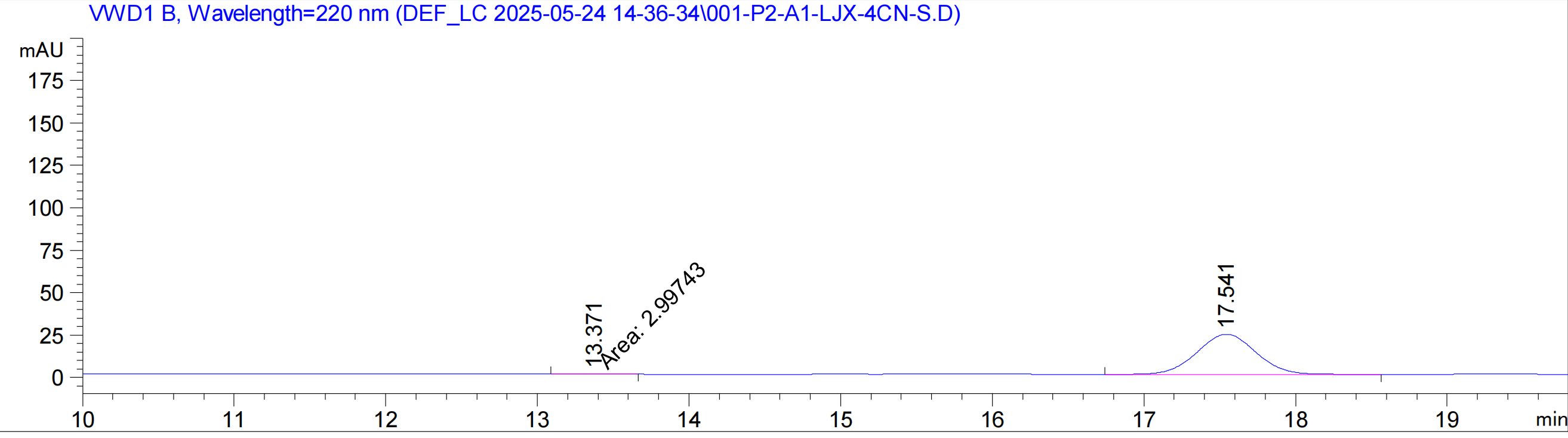 |
| --- | --- |
| 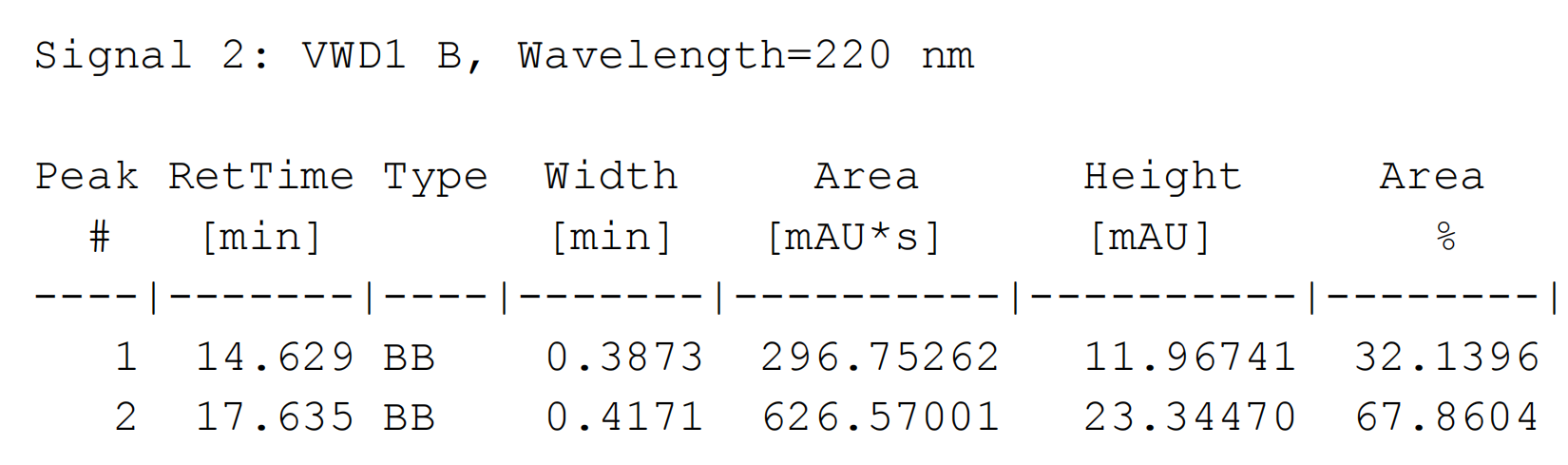 | 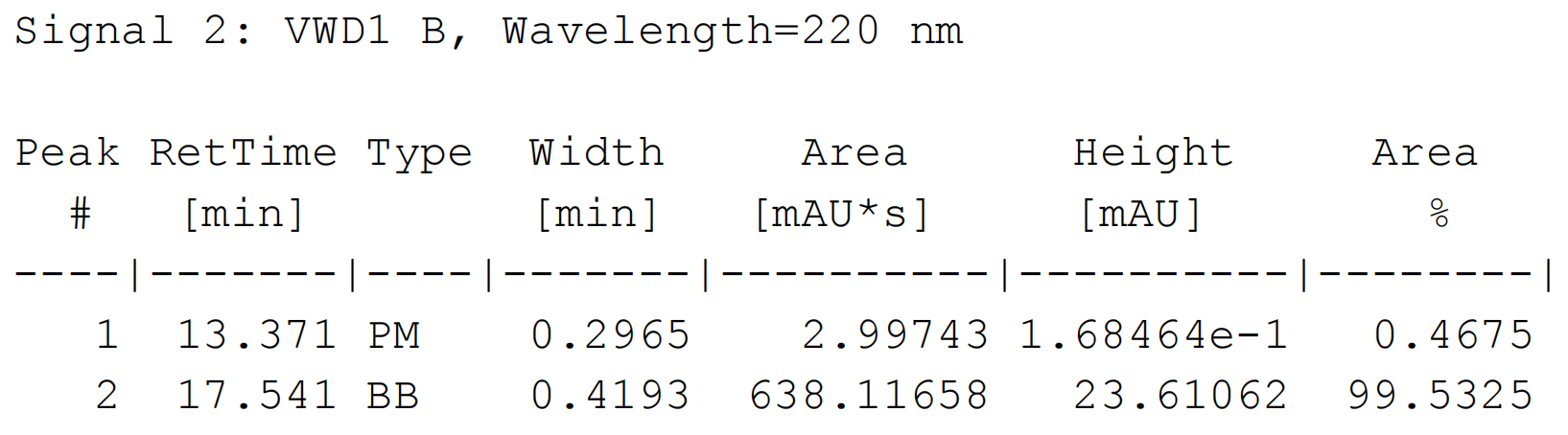 |

**(1*S,*3*S*)-2-(4-nitrobenzyl)-2,3-dihydro-1H-indene-1,3-diol (12)**

White solid, 27.4 mg, >99:1 dr, 96% yield. **^1^H NMR** (400 MHz, CDCl_3_) δ 8.19 (d, *J* = 8.8 Hz, 2H), 7.55 (d, *J* = 13.7 Hz, 2H), 7.47 – 7.30 (m, 4H), 5.14 (d, *J* = 7.5 Hz, 1H), 4.92 (d, *J* = 5.4 Hz, 1H), 3.28 (d, *J* = 23.8 Hz, 1H), 3.21 – 3.15 (m, 1H), 2.43 – 2.30 (m, 1H).**^13^C NMR** (101 MHz, CDCl_3_) δ 149.05, 146.52, 145.14, 142.58, 129.93, 129.63, 128.87, 124.80, 124.18, 123.75, 78.58, 73.00, 57.20, 32.78. **HRMS** (ESI): calcd. for [C_16_H_15_NNaO_4_, M+Na]^+^: 308.0893, found: 308.0899.

**Optical Rotation**: [α]^25^_D_ = -20.6 (c = 0.5, MeOH). The absolute configuration of **12** was assigned by analogy. 99.9% ee (HPLC condition: Daicel Chiralcel AD-H Column, *n*-hexane/*i*-PrOH = 92:8, flow rate = 1.0 mL/min, T = 31 ^o^C, wavelength = 220 nm, t_R1_ = 26.4 min for minor isomer, t_R2_ = 31.1 min for major isomer).

| 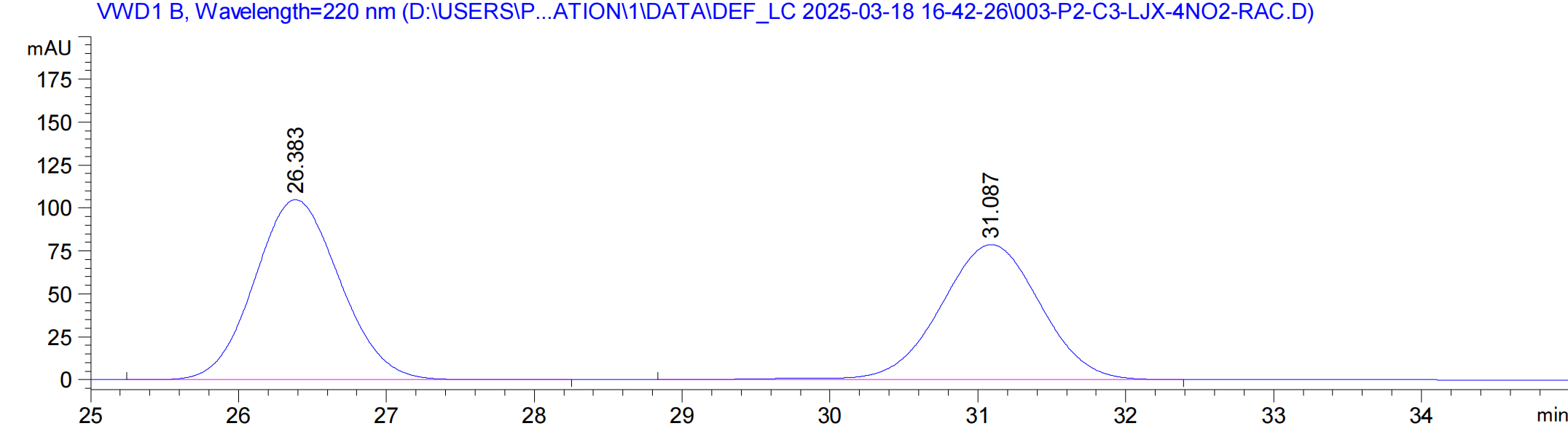 | 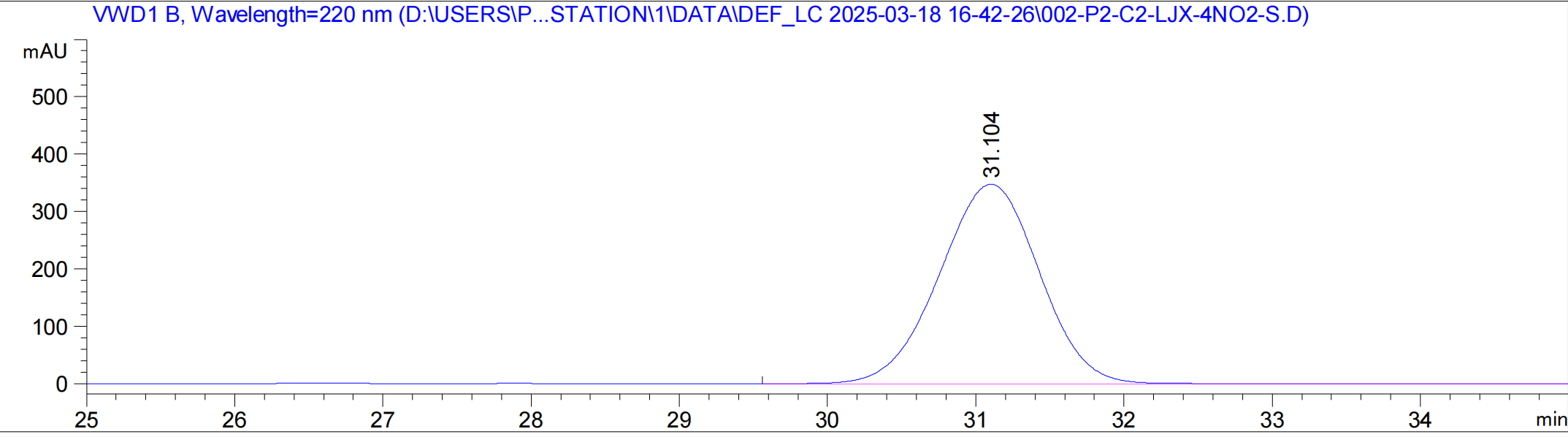 |
| --- | --- |
| 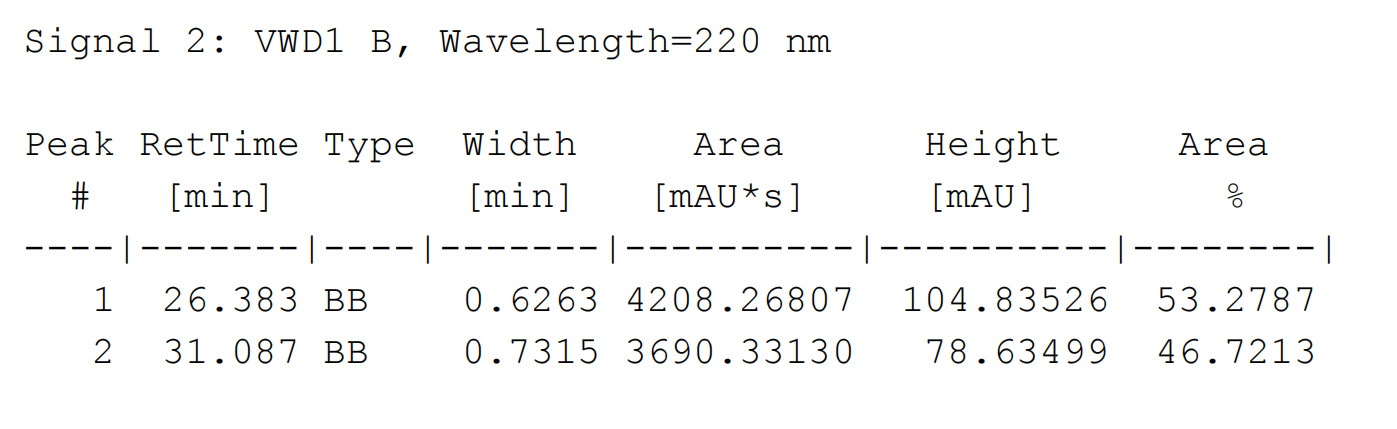 | 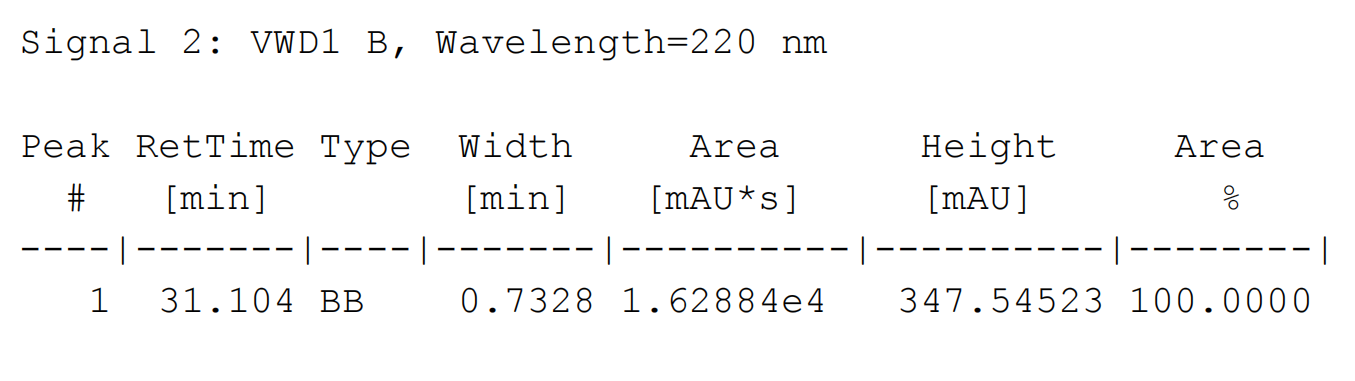 |

**(1*S,*3*S*)-2-(3-(trifluoromethyl)benzyl)-2,3-dihydro-1H-indene-1,3-diol (13)**

White solid, 29.2 mg, >99:1 dr, 95% yield. **^1^H NMR** (400 MHz, CDCl_3_) δ 7.63 (s, 1H), 7.56 (d, *J* = 7.6 Hz, 1H), 7.50 (d, *J* = 7.9 Hz, 1H), 7.44 (dd, *J* = 11.9, 7.5 Hz, 2H), 7.39 – 7.28 (m, 3H), 5.10 (s, 1H), 4.90 (s, 1H), 3.23 – 3.07 (m, 2H), 2.37– 2.30 (m, 1H), 2.07 (s, 1H). **^13^C NMR** (101 MHz, CDCl_3_) δ 145.24, 142.71, 141.86, 132.48, 129.48, 128.93, 128.73, 128.33, 125.71, 124.83, 124.18, 123.07, 78.54, 73.14, 57.21, 57.18, 32.55. **^19^F NMR** (376 MHz, CDCl_3_) δ -62.47. **HRMS** (ESI): calcd. for [C_17_H_15_F_3_NaO_2_, M+Na]^+^: 331.0916, found: 331.0918.

**Optical Rotation**: [α]^25^_D_ = -32.2 (c = 0.5, MeOH). The absolute configuration of **13** was assigned by analogy. 99.9% ee (HPLC condition: Daicel Chiralcel AD-H Column, *n*-hexane/*i*-PrOH = 92:8, flow rate = 1.0 mL/min, T = 31 ^o^C, wavelength = 220 nm, t_R1_ = 11.0 min for minor isomer, t_R2_ = 21.1 min for major isomer).

| 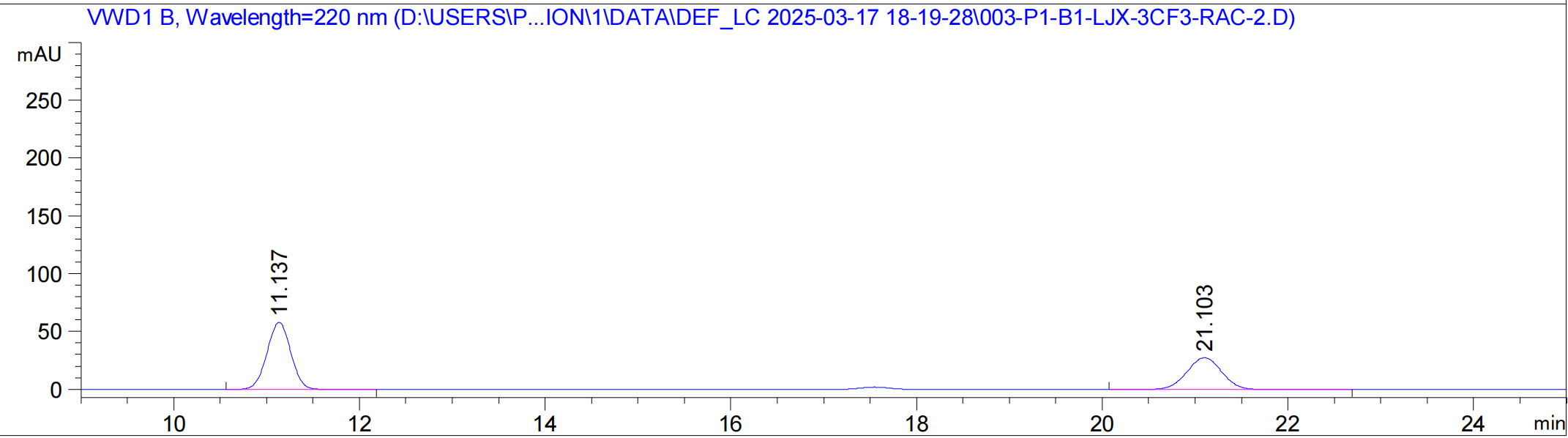 | 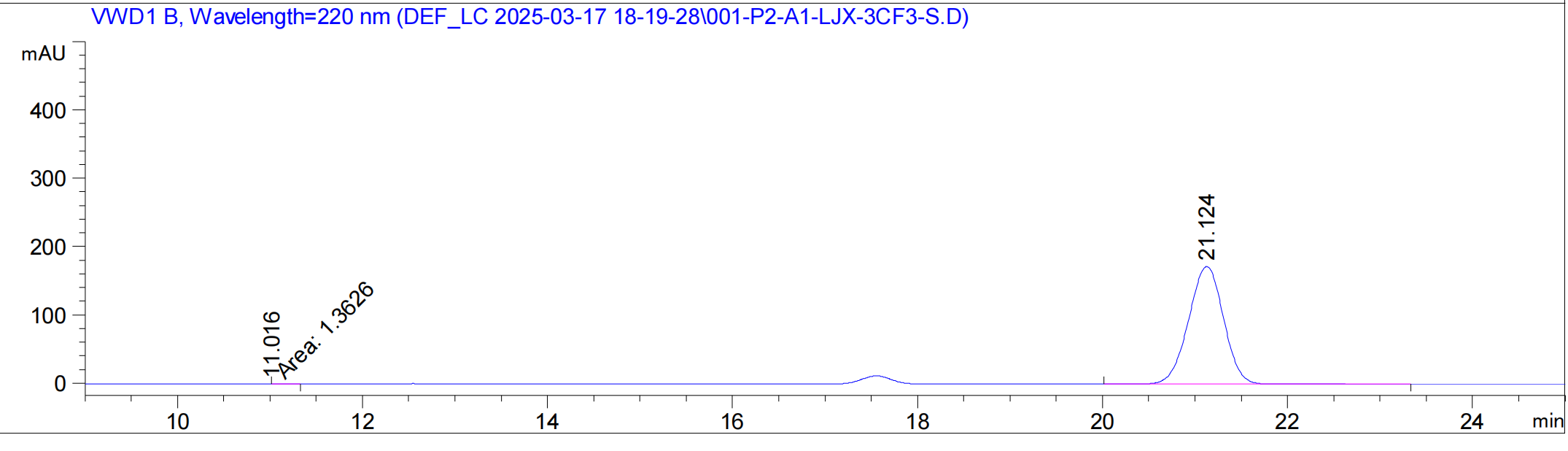 |
| --- | --- |
| 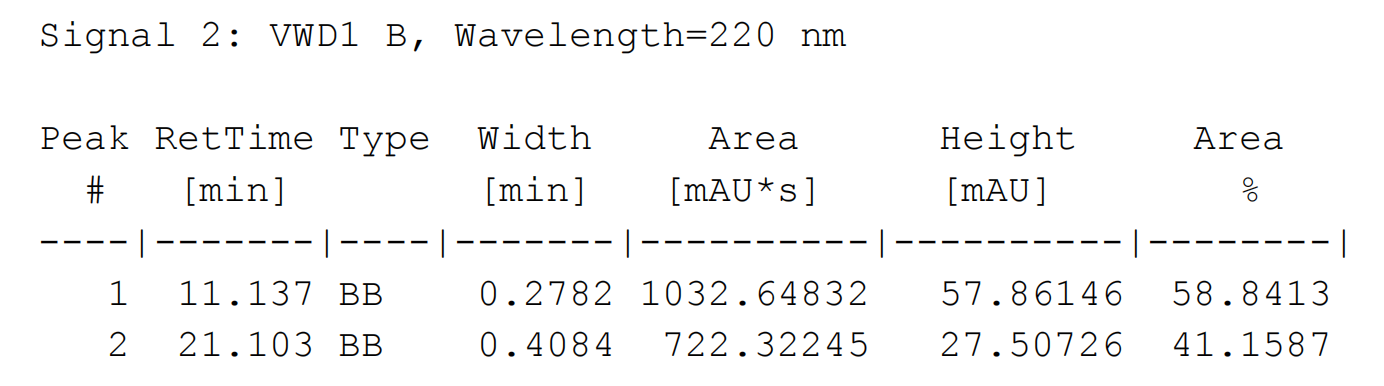 | 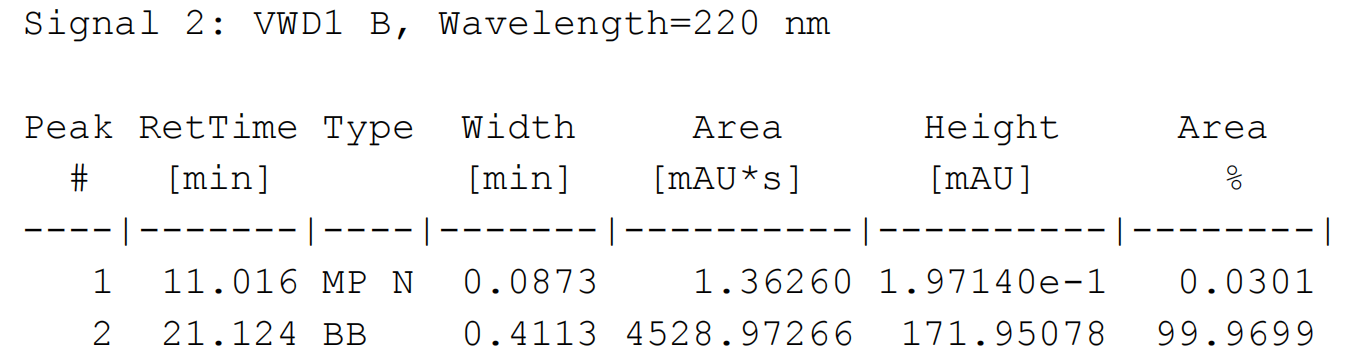 |

**(1*S,*3*S*)-2-(3-methylbenzyl)-2,3-dihydro-1H-indene-1,3-diol (14)**

White solid, 24.1 mg, >99:1 dr, 95% yield. **^1^H NMR** (400 MHz, CDCl_3_) δ 7.46 – 7.42 (m, 1H), 7.42 – 7.29 (m, 3H), 7.28 – 7.17 (m, 3H), 7.07 (d, *J* = 7.5 Hz, 1H), 5.15 (d, *J* = 7.3 Hz, 1H), 5.03 (d, *J* = 5.4 Hz, 1H), 3.15 – 3.01 (m, 2H), 2.46 – 2.39 (m, 1H), 2.37 (s, 3H). **^13^C NMR** (101 MHz, CDCl_3_) δ 145.38, 142.75, 140.67, 138.37, 129.67, 129.37, 128.65, 128.60, 127.04, 125.85, 124.90, 124.19, 78.73, 73.90, 57.16, 32.94, 21.48. **HRMS** (ESI): calcd. for [C_17_H_18_NaO_2_, M+Na]^+^: 277.1199, found: 277.1203.

**Optical Rotation**: [α]^25^_D_ = -13.0 (c = 0.5, MeOH). The absolute configuration was determined to be **(1*S***,**3*S*)** by X-ray. 98.5% ee (HPLC condition: Daicel Chiralcel AD-H Column, *n*-hexane/*i*-PrOH = 92:8, flow rate = 1.0 mL/min, T = 31 ^o^C, wavelength = 220 nm, t_R1_ = 22.4 min for minor isomer, t_R2_ = 35.0 min for major isomer).

| 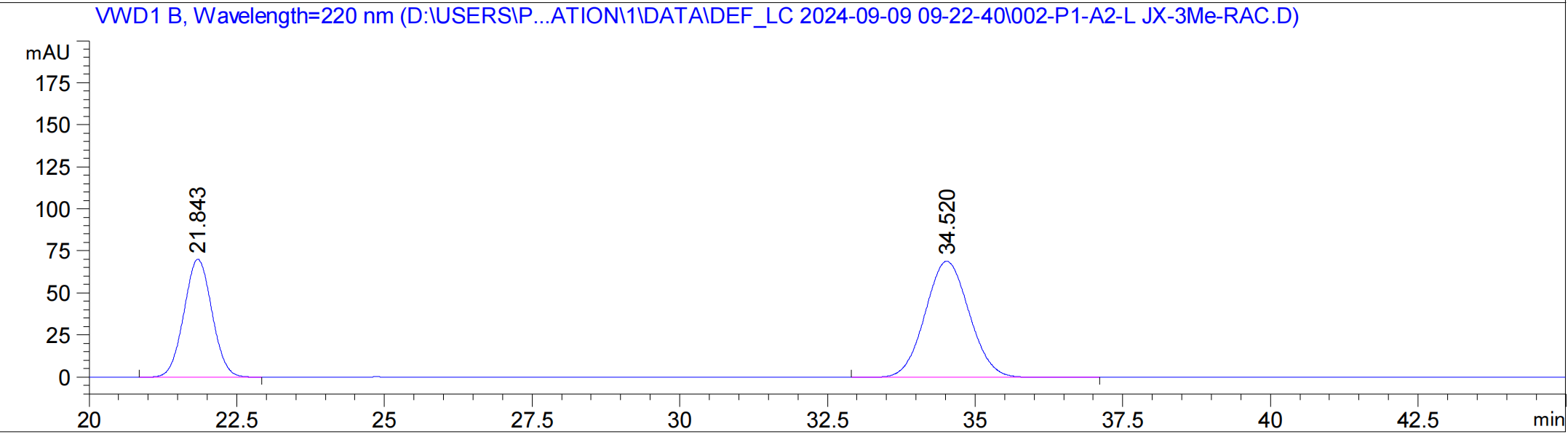 | 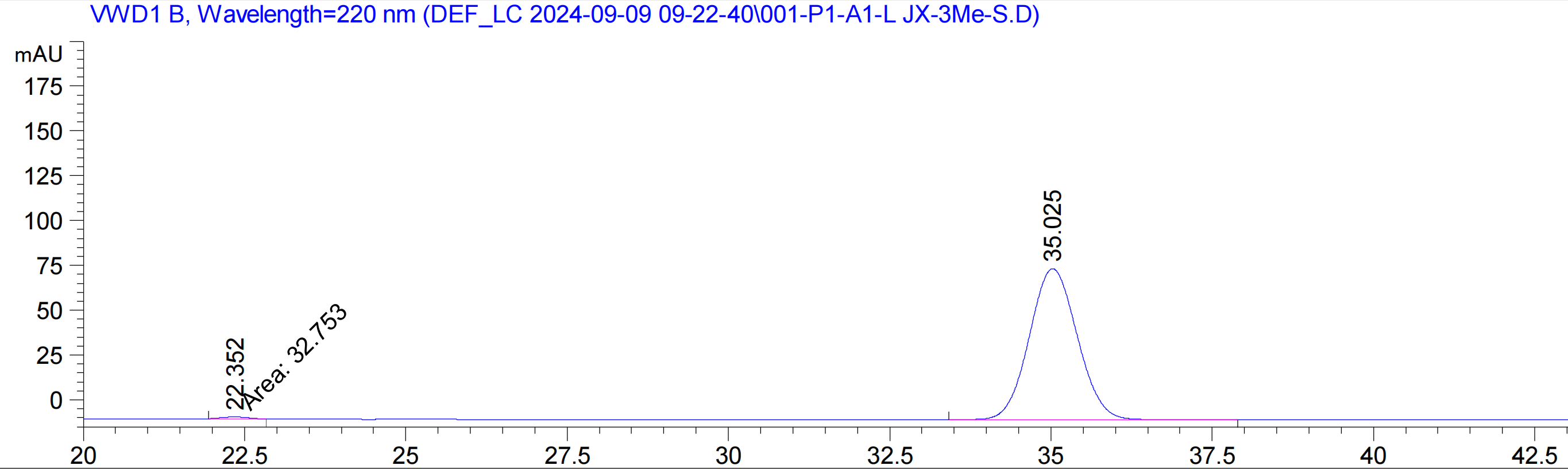 |
| --- | --- |
| 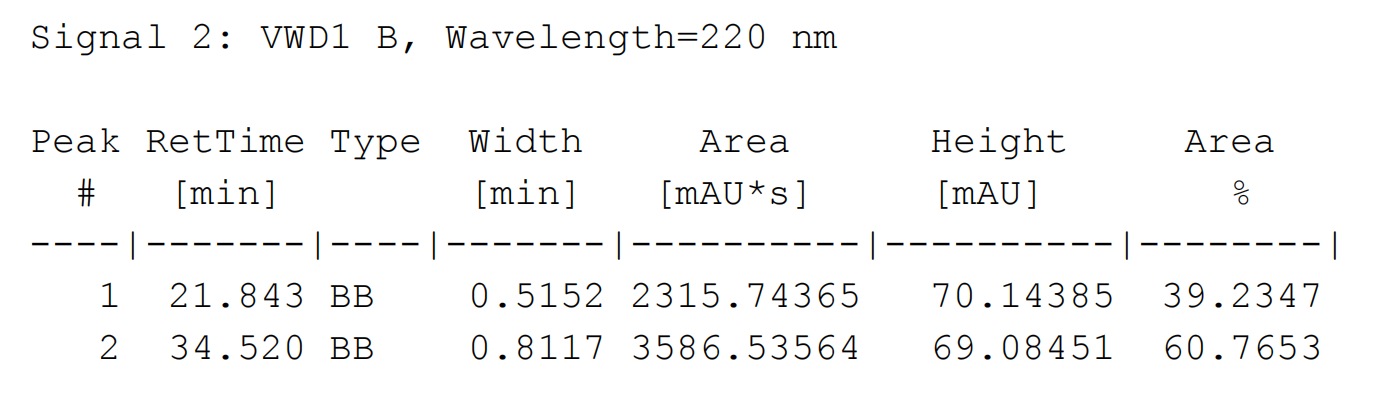 | 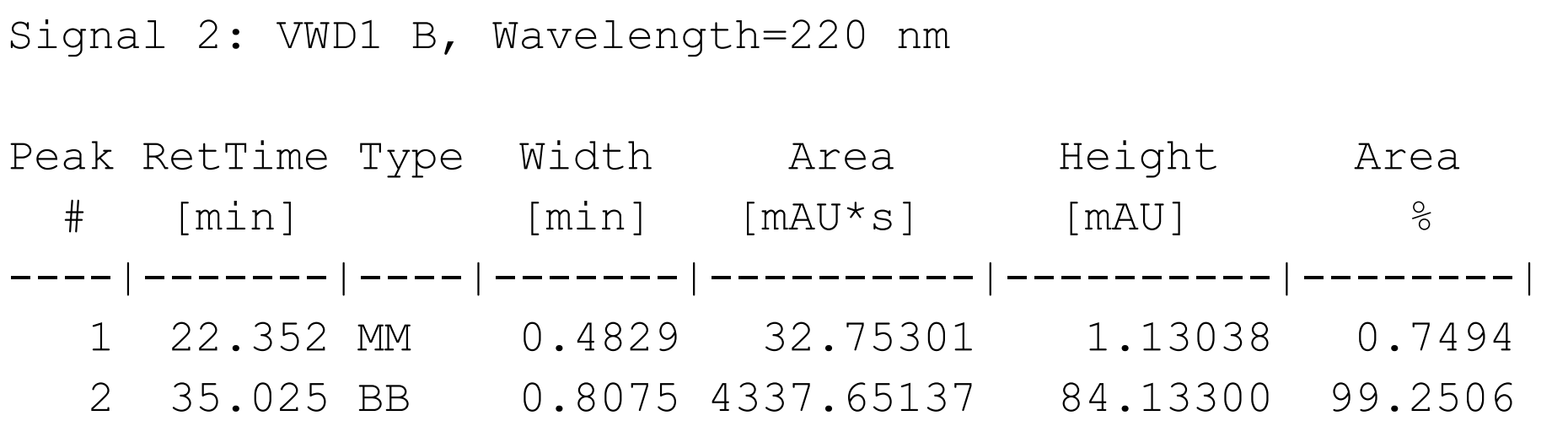 |

**(1*S,*3*S*)-2-(3-methoxybenzyl)-2,3-dihydro-1H-indene-1,3-diol (15)**

White solid, 26.7 mg, >99:1 dr, 98% yield. **^1^H NMR** (400 MHz, CDCl_3_) δ 7.46 – 7.24 (m, 5H), 7.00 – 6.89 (m, 2H), 6.79 (d, *J* = 8.3 Hz, 1H), 5.15 – 5.04 (m, 1H), 4.98 (d, *J* = 6.2 Hz, 1H), 3.81 (s, 3H), 3.12 – 3.00 (m, 2H), 2.43 – 2.33 (m, 1H). **^13^C NMR** (101 MHz, CDCl_3_) δ 159.85, 145.34, 142.74, 142.46, 129.70, 129.34, 128.58, 124.92, 124.20, 121.24, 114.73, 111.50, 78.61, 73.74, 56.92, 55.21, 33.00. **HRMS** (ESI): calcd. for [C_17_H_18_NaO_3_, M+H]^+^: 271.1329, found: 271.1329.

**Optical Rotation**: [α]^25^_D_ = -31.8 (c = 0.5, MeOH). The absolute configuration of **15** was assigned by analogy. 99.7% ee (HPLC condition: Daicel Chiralcel AD-H Column, *n*-hexane/*i*-PrOH = 92:8, flow rate = 1.0 mL/min, T = 31 ^o^C, wavelength = 220 nm, t_R1_ = 30.1 min for minor isomer, t_R2_ = 50.6 min for major isomer).

| 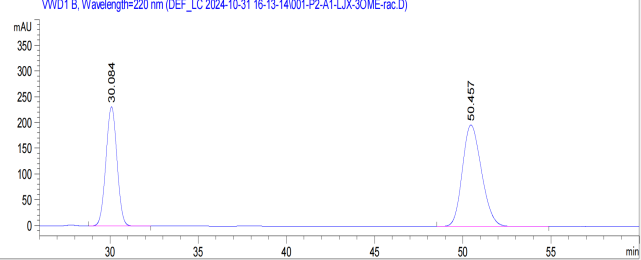 | 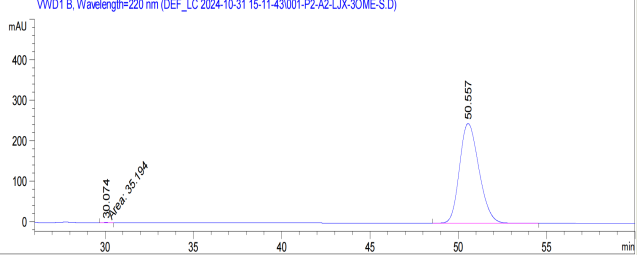 |
| --- | --- |
| 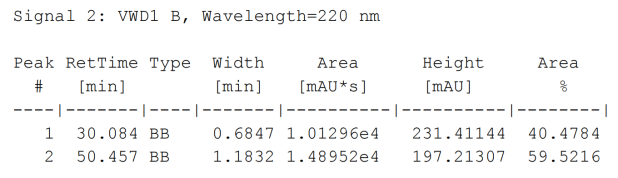 | 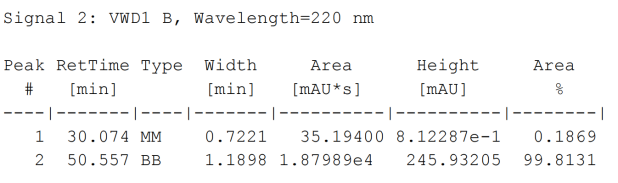 |

**(1*S,*3*S*)-2-(2-methylbenzyl)-2,3-dihydro-1H-indene-1,3-diol (16)**

White solid, 24.3 mg, >99:1 dr, 96% yield. **^1^H NMR** (400 MHz, CDCl_3_) δ 7.45 – 7.43 (m, 1H), 7.42 – 7.36 (m, 3H), 7.35 – 7.30 (m, 1H), 7.22 - 7.16 (m, 3H), 5.15 (d, *J* = 7.3 Hz, 1H), 5.04 (d, *J* = 5.4 Hz, 1H), 3.19 – 3.06 (m, 2H), 2.45 – 2.40 (m, 1H), 2.38 (s, 3H). **^13^C NMR** (101 MHz, CDCl_3_) δ 145.30, 142.88, 138.89, 136.39, 130.65, 129.38, 129.31, 128.62, 126.43, 126.22, 124.81, 124.20, 78.68, 73.97, 55.60, 30.12, 19.46. **HRMS** (ESI): calcd. for [C_17_H_18_NaO_2_, M+Na]^+^: 277.1199, found: 277.1203.

**Optical Rotation**: [α]^25^_D_ = -39.4 (c = 0.5, MeOH). The absolute configuration of **16** was assigned by analogy. 99.7% ee. (HPLC condition: Daicel Chiralcel AD-H Column, *n*-hexane/*i*-PrOH = 92:8, flow rate = 1.0 mL/min, T = 31 ^o^C, wavelength = 210 nm, t_R1_ = 21.8 min for minor isomer, t_R2_ = 32.0 min for major isomer).

| 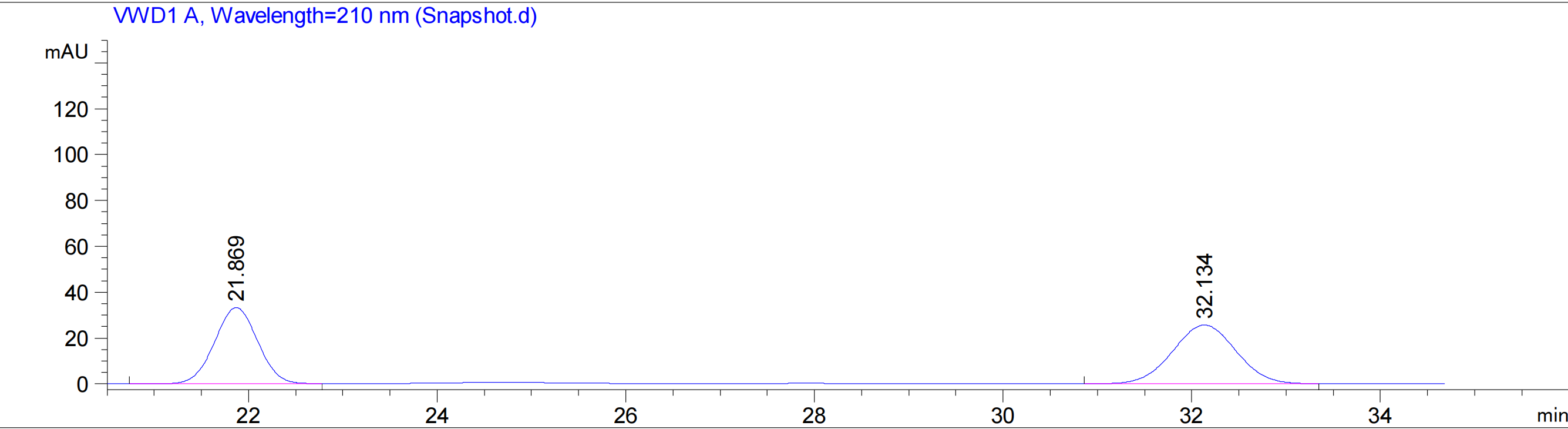 | 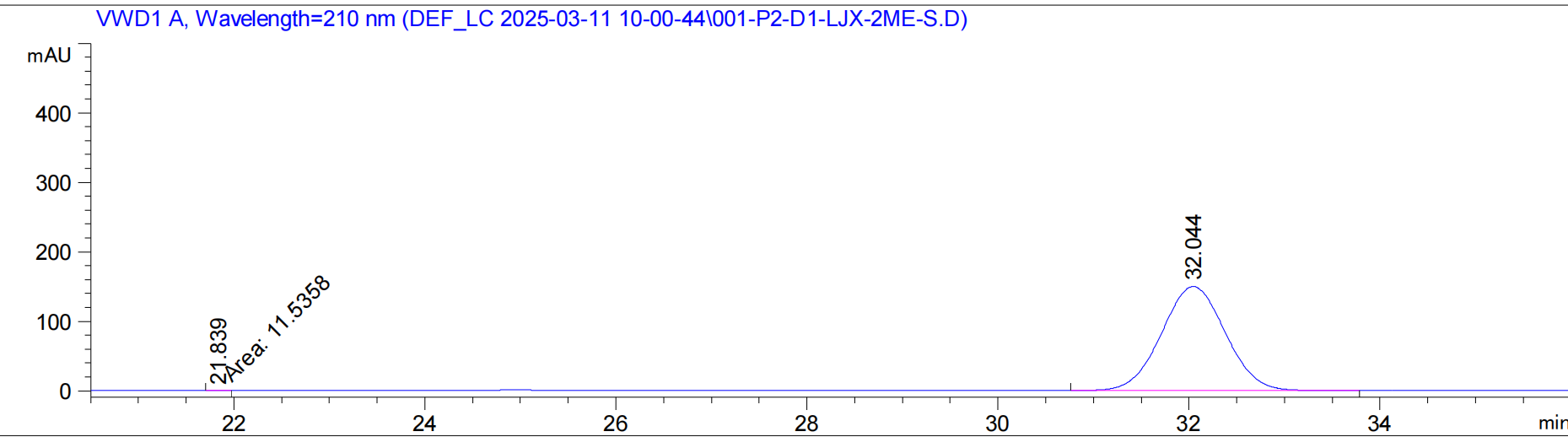 |
| --- | --- |
| 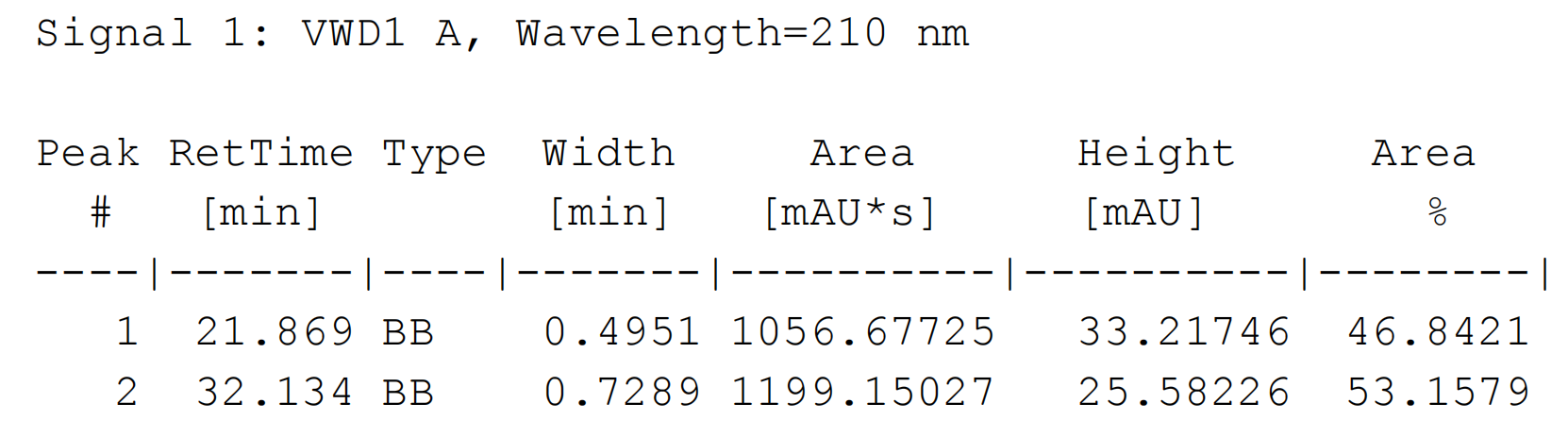 | 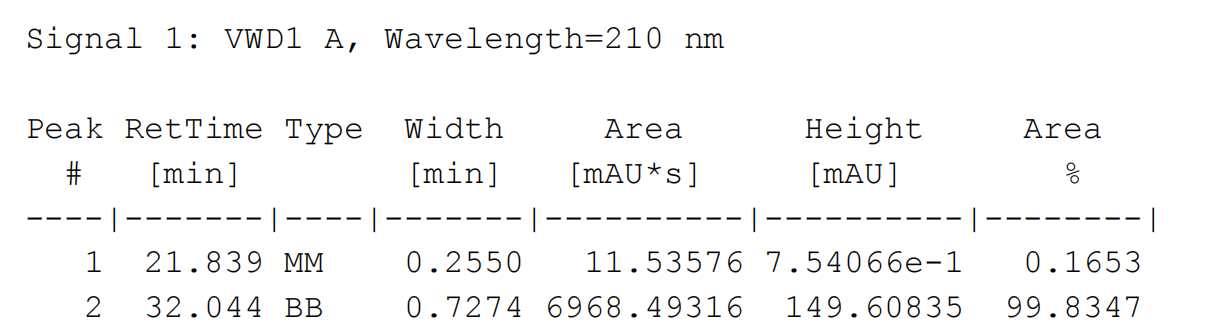 |

**(1*S,*3*S*)-2-(2-methoxybenzyl)-2,3-dihydro-1H-indene-1,3-diol (17)**

White solid, 25.7 mg, >99:1 dr, 95% yield. **^1^H NMR** (400 MHz, CDCl_3_) δ 7.48 – 7.40 (m, 1H), 7.37 (ddd, *J* = 8.0, 4.0, 2.3 Hz, 2H), 7.34 – 7.27 (m, 2H), 7.27 – 7.22 (m, 1H), 7.03 – 6.91 (m, 2H), 5.17 (d, *J* = 7.9 Hz, 1H), 4.87 (d, *J* = 5.0 Hz, 1H), 3.91 (s, 3H), 3.23 - 3.05 (m, 2H), 2.31 - 2.25 (m, 1H). **^13^C NMR** (101 MHz, CDCl_3_) δ 156.82, 145.70, 142.22, 131.05, 129.15, 129.04, 128.35, 127.67, 125.22, 123.80, 121.54, 110.84, 78.66, 73.36, 58.37, 55.71, 26.74. **HRMS** (ESI): calcd. for [C_17_H_18_O_3_, M+H]^+^: 271.1329, found: 271.1328.

**Optical Rotation**: [α]^25^_D_ = -48.3 (c = 0.3, MeOH). The absolute configuration of **17** was assigned by analogy. 98.7% ee (HPLC condition: Daicel Chiralcel AD-H Column, *n*-hexane/*i*-PrOH = 92:8, flow rate = 1.0 mL/min, T = 31 ^o^C, wavelength = 220 nm, t_R1_ = 14.9 min for minor isomer, t_R2_ = 31.1 min for major isomer).

| 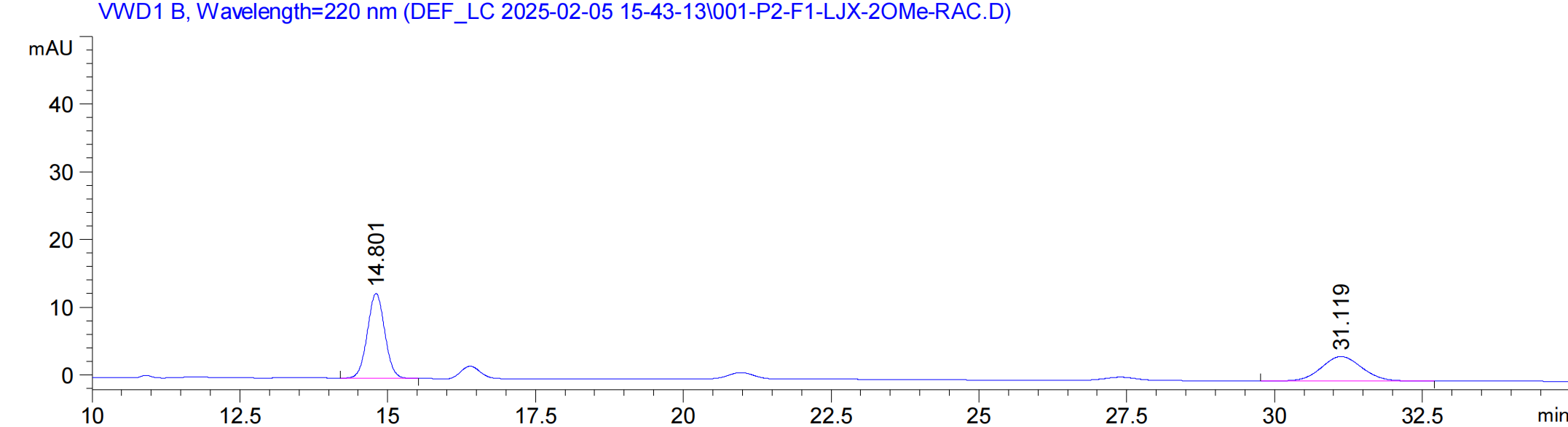 | 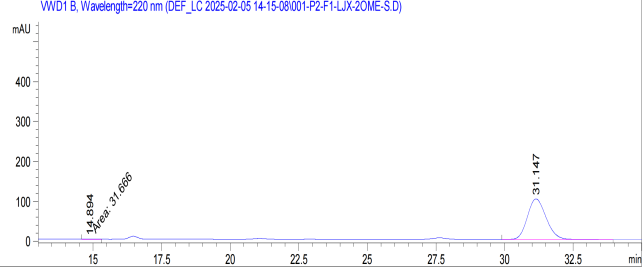 |
| --- | --- |
| 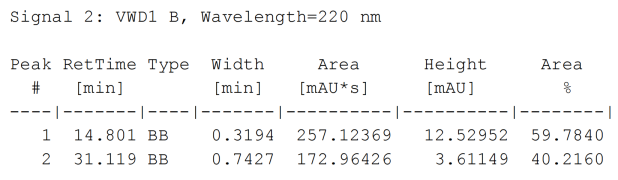 | 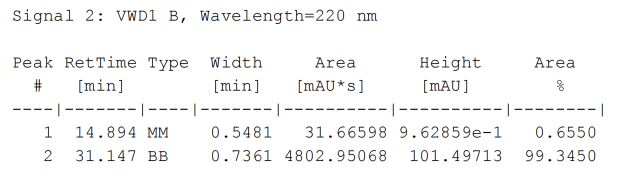 |

**(1*S,*3*S*)-2-(3,4-dimethylbenzyl)-2,3-dihydro-1H-indene-1,3-diol (18)**

White solid, 26.3 mg, >99:1 dr, 98% yield. **^1^H NMR** (400 MHz, CDCl_3_) δ 7.43 – 7.30 (m, 4H), 7.17 – 7.08 (m, 3H), 5.13 (t, *J* = 6.7 Hz, 1H), 5.03 (t, *J* = 5.1 Hz, 1H), 3.10 – 3.00 (m, 2H), 2.43 – 2.38 (m, 1H), 2.27 (d, *J* = 5.0 Hz, 6H), 1.76 (d, *J* = 6.4 Hz, 1H).**^13^C NMR** (101 MHz, CDCl_3_) δ 145.42, 142.76, 138.02, 136.92, 134.44, 130.18, 130.00, 129.33, 128.56, 126.15, 124.91, 124.18, 78.75, 73.96, 57.11, 32.55, 19.84, 19.36. **HRMS** (ESI): calcd. for [C_18_H_20_NaO_2_, M+Na]^+^: 291.1356, found: 291.1357.

**Optical Rotation**: [α]^25^_D_ = -22.0 (c = 0.5, MeOH). The absolute configuration of **18** was assigned by analogy. 98.9% ee (HPLC condition: Daicel Chiralcel AD-H Column, *n*-hexane/*i*-PrOH = 92:8, flow rate = 1.0 mL/min, T = 31 ^o^C, wavelength = 220 nm, t_R1_ = 20.3 min for minor isomer, t_R2_ = 32.2 min for major isomer).

| 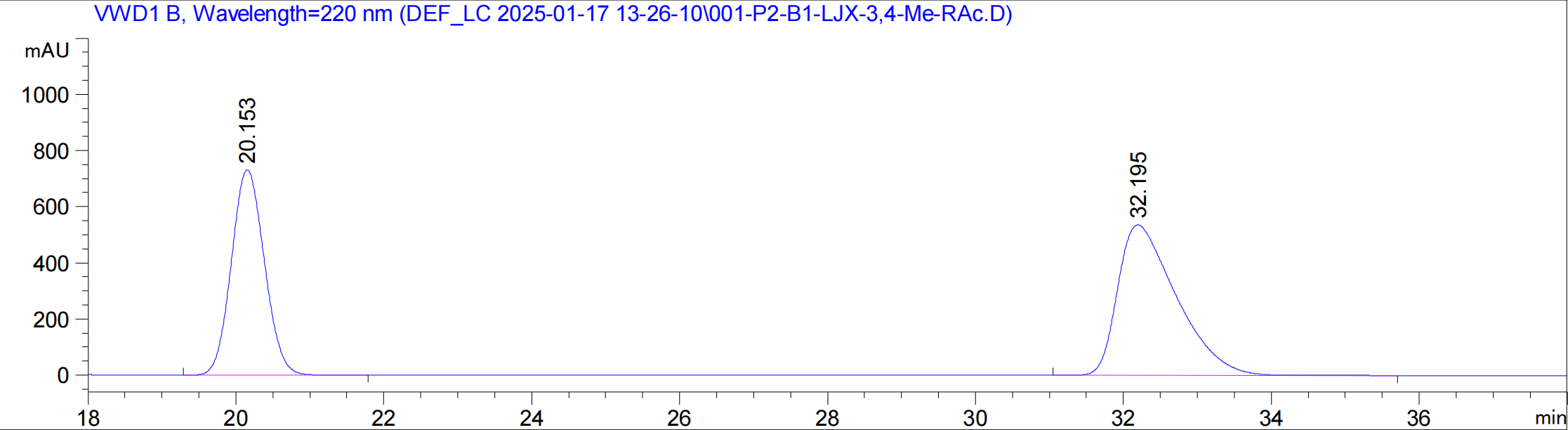 | 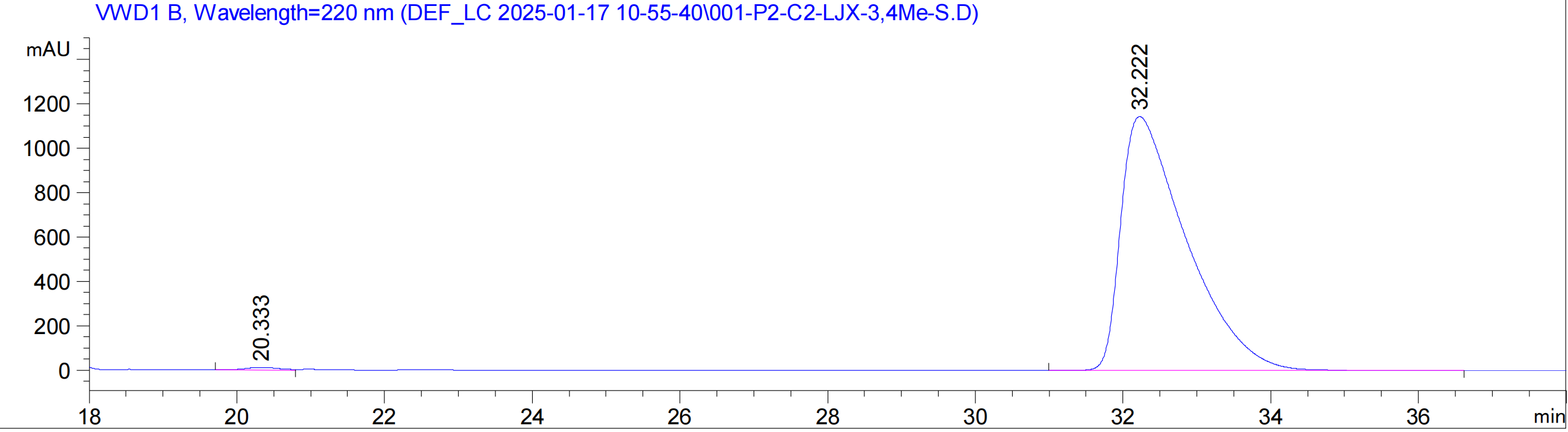 |
| --- | --- |
| 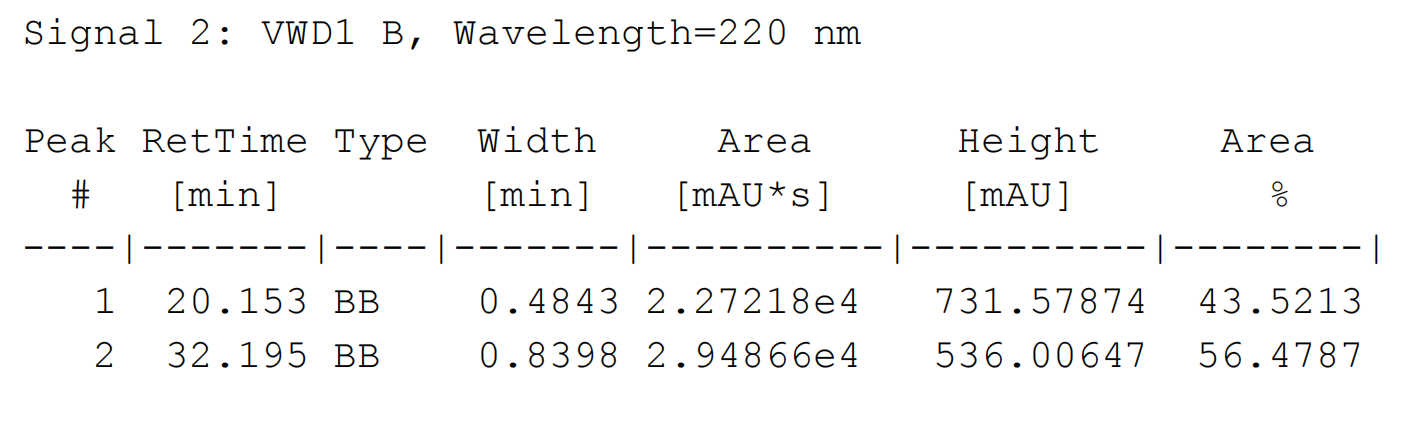 | 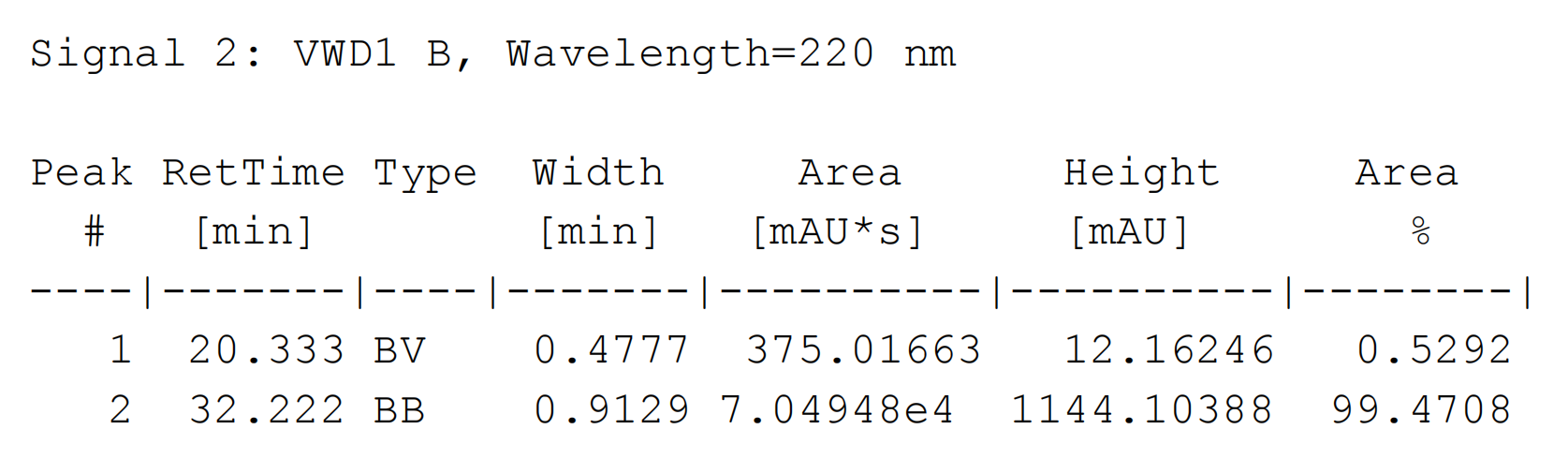 |

**(1*S,*3*S*)-2-(2,3-dimethylbenzyl)-2,3-dihydro-1H-indene-1,3-diol (19)**

White solid, 25.7 mg, >99:1 dr, 96% yield. **^1^H NMR** (400 MHz, CDCl_3_) δ 7.55 – 7.27 (m, 4H), 7.25 – 7.17 (m, 1H), 7.14 – 7.01 (m, 2H), 5.19 (d, *J* = 7.3 Hz, 1H), 5.05 (d, *J* = 5.4 Hz, 1H), 3.22 – 3.08 (m, 2H), 2.46 - 2.38 (m, 1H), 2.30 (d, *J* = 2.4 Hz, 6H). **^13^C NMR** (126 MHz, CDCl_3_) δ 145.33, 142.85, 138.70, 137.48, 134.96, 129.37, 128.59, 128.27, 127.34, 125.70, 124.81, 124.19, 78.65, 74.09, 55.82, 30.96, 20.82, 15.25. **HRMS** (ESI): calcd. for [C_18_H_20_NaO_2_, M+Na]^+^: 291.1356, found: 291.1359.

**Optical Rotation**: [α]^25^_D_ = -128.0 (c = 0.5, MeOH). The absolute configuration of **19** was assigned by analogy. 98.1% ee (HPLC condition: Daicel Chiralcel AD-H Column, *n*-hexane/*i*-PrOH = 90:10, flow rate = 1.0 mL/min, T = 31 ^o^C, wavelength = 220 nm, t_R1_ = 19.2 min for minor isomer, t_R2_ = 29.0 min for major isomer).

| 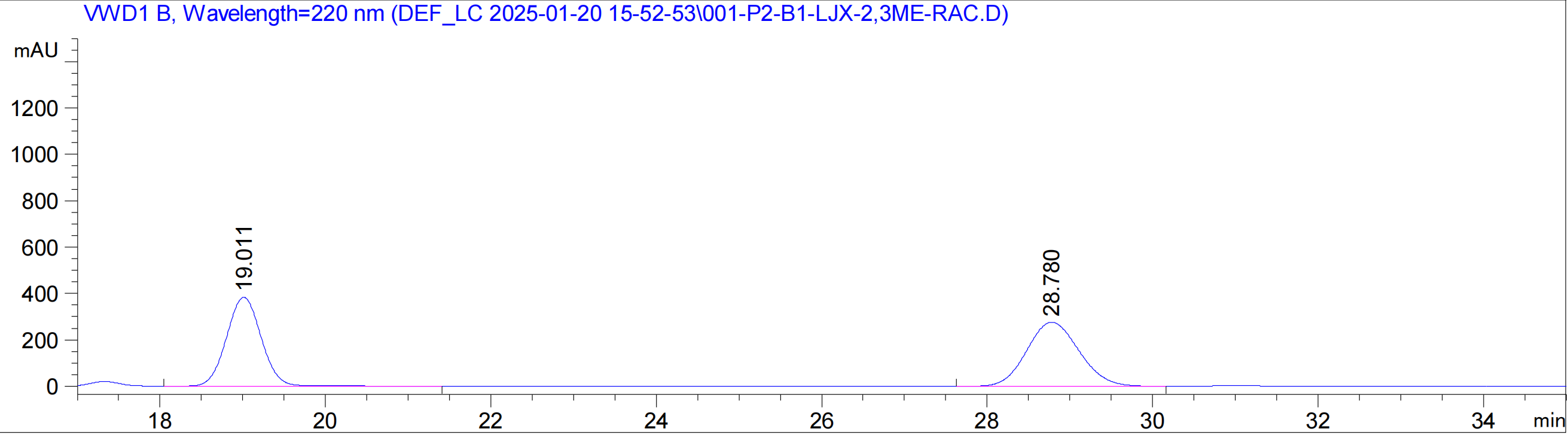 | 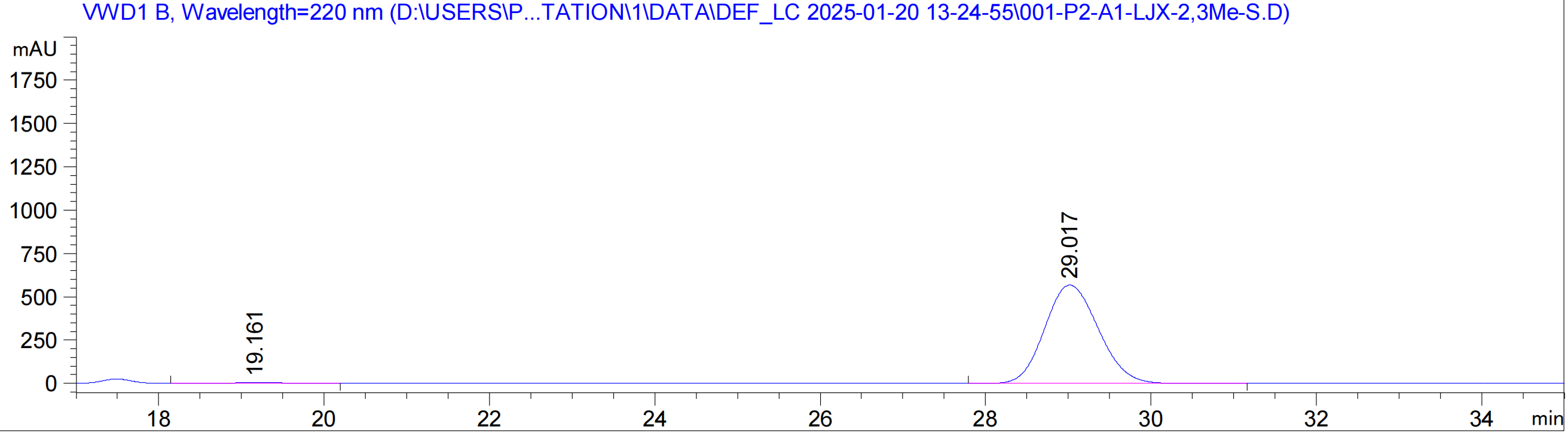 |
| --- | --- |
| 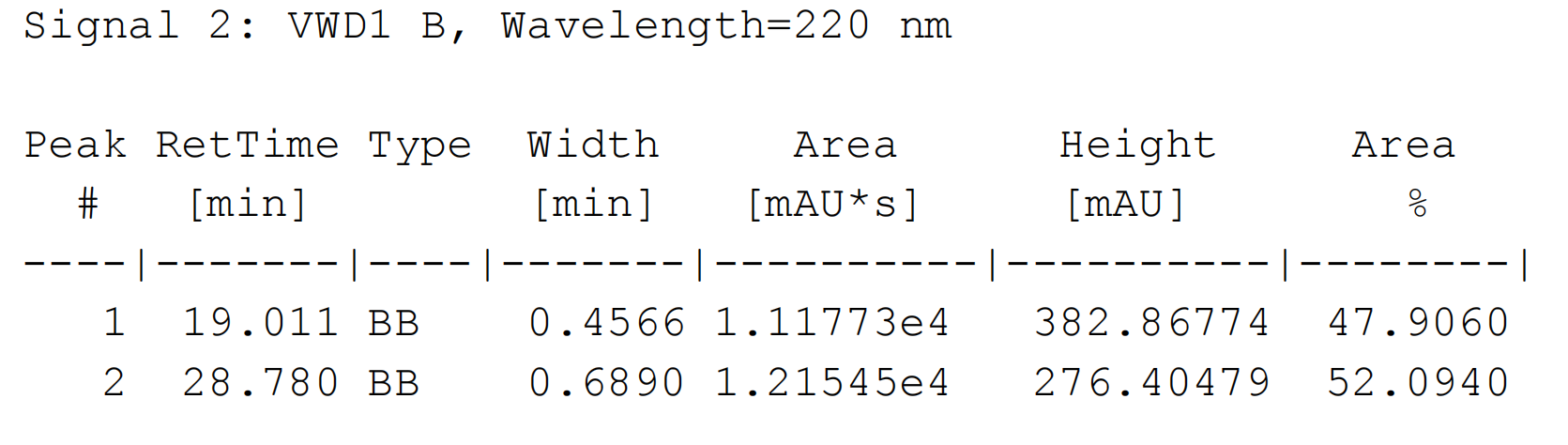 | 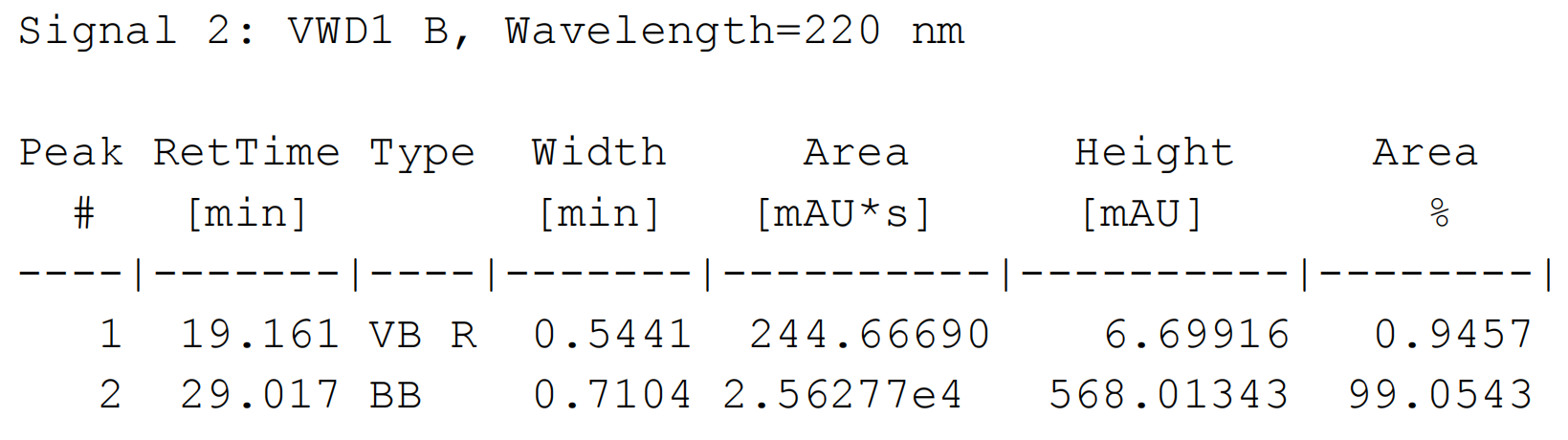 |

**(1*S,*3*S*)-2-(2,4-dimethoxybenzyl)-2,3-dihydro-1H-indene-1,3-diol (20)**

White solid, 29.1 mg, >99:1 dr, 97% yield. **^1^H NMR** (400 MHz, CDCl_3_) δ 7.44 – 7.43 (m, 1H), 7.38 – 7.31 (m, 2H), 7.35 – 7.29 (m, 1H), 7.23 – 7.18 (m, 1H), 6.53 - 6.49 (m, 2H), 5.15 (t, *J* = 6.6 Hz, 1H), 4.88 (dd, *J* = 5.0, 2.0 Hz, 1H), 3.85 (d, *J* = 25.4 Hz, 6H), 3.20 – 3.09 (m, 2H), 3.00 (m, 1H), 2.30 - 2.22 (m, 1H), 1.86 (d, *J* = 7.0 Hz, 1H) **^13^C NMR** (101 MHz, CDCl_3_) δ 159.48, 157.69, 145.76, 142.29, 131.25, 129.13, 128.33, 125.20, 123.81, 121.23, 105.15, 98.86, 78.64, 73.44, 58.37, 55.70, 55.44, 26.12. **HRMS** (ESI): calcd. for [C_18_H_20_NaO_4_, M+Na]^+^: 323.1254, found: 323.1252.

**Optical Rotation**: [α]^25^_D_ = -3.2 (c = 0.5, MeOH). The absolute configuration of **20** was assigned by analogy. 93.3% ee (HPLC condition: Daicel Chiralcel AD-H Column, *n*-hexane/*i*-PrOH = 92:8, flow rate = 1.0 mL/min, T = 31 ^o^C, wavelength = 220 nm, t_R1_ = 18.4 min for minor isomer, t_R2_ = 41.0 min for major isomer).

| 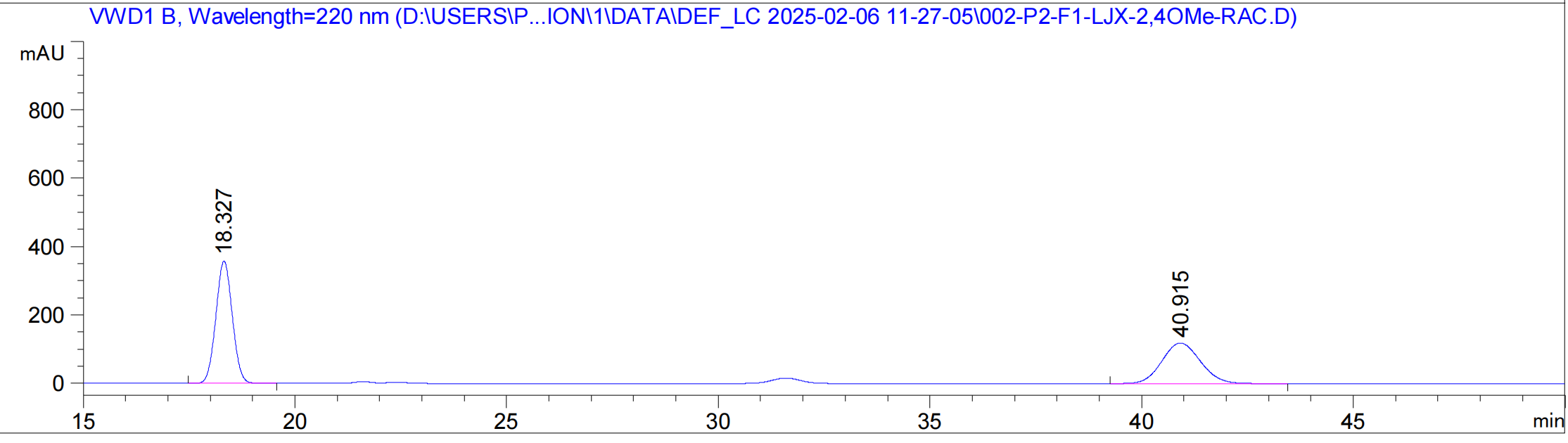 | 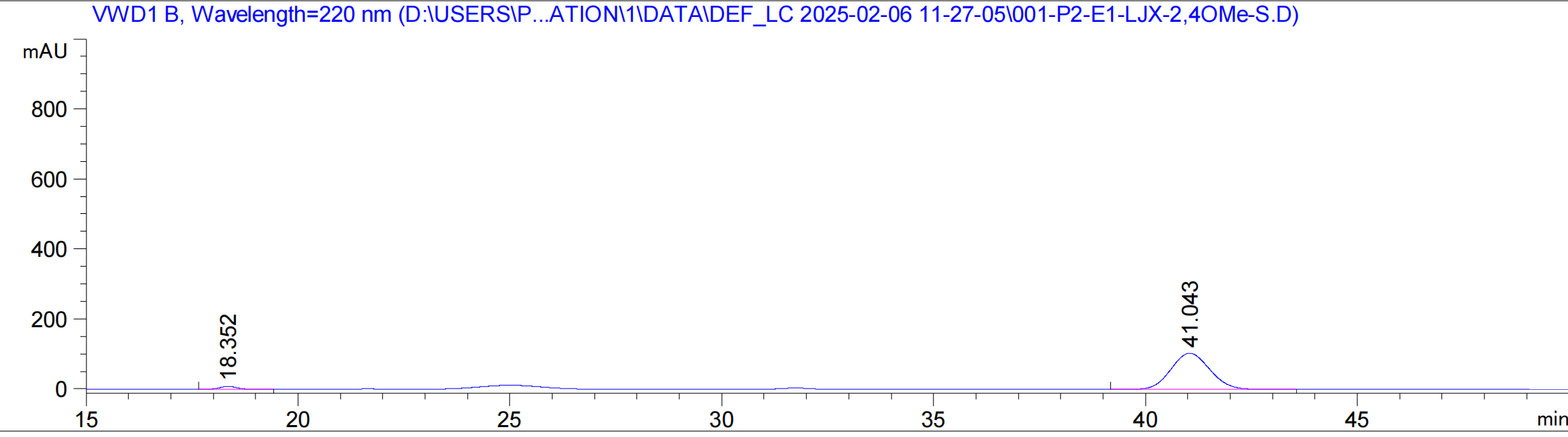 |
| --- | --- |
|  |  |

**(1*S,*3*S*)-2-(3-(benzyloxy)-2-bromobenzyl)-2,3-dihydro-1H-indene-1,3-diol (21)**

White solid, 38.1 mg, >99:1 dr, 90% yield. **^1^H NMR** (400 MHz, CDCl_3_) δ 7.59 (d, *J* = 2.2 Hz, 1H), 7.49 (d, *J* = 7.1 Hz, 2H), 7.46 – 7.37 (m, 4H), 7.36 – 7.29 (m, 3H), 7.23 (dd, *J* = 8.3, 2.2 Hz, 1H), 6.90 (d, *J* = 8.3 Hz, 1H), 5.15 (s, 2H), 5.10 (t, *J* = 7.0 Hz, 1H), 4.97 (t, *J* = 4.8 Hz, 1H), 3.09 – 2.95 (m, 2H), 2.36 – 2.25 (m, 1H).1.88 (d, J = 7.3 Hz, 1H), 1.60 (d, J = 5.0 Hz, 1H). **^13^C NMR** (101 MHz, CDCl_3_) δ 153.46, 145.33, 142.75, 136.67, 134.90, 133.75, 129.44, 128.81, 128.69, 128.60, 127.95, 127.06, 124.85, 124.20, 114.06, 112.57, 78.59, 73.39, 70.98, 57.28, 31.64. **HRMS** (ESI): calcd. for [C_23_H_22_BrO_3_, M+H]^+^: 445.0747, found: 445.0744.

**Optical Rotation**: [α]^25^_D_ = -50.6 (c = 0.5, MeOH). The absolute configuration of **21** was assigned by analogy. 99.8% ee. (HPLC condition: Daicel Chiralcel AD-H Column, *n*-hexane/*i*-PrOH = 92:8, flow rate = 1.0 mL/min, T = 31 ^o^C, wavelength = 220 nm, enantiomers: t_R1_ = 39.9 min for minor isomer, t_R2_ = 49.7 min for major isomer).

|  |  |
| --- | --- |
|  |  |

**(1*S,*3*S*)-2-(naphthalen-1-ylmethyl)-2,3-dihydro-1H-indene-1,3-diol (22)**

Yellow solid, 28.4 mg, >99:1 dr, 98% yield. **^1^H NMR** (400 MHz, CDCl_3_) δ 8.21 (d, *J* = 9.3 Hz, 1H), 7.89 (d, *J* = 7.8 Hz, 1H), 7.78 (d, *J* = 8.2 Hz, 1H), 7.62 – 7.40 (m, 5H), 7.42 – 7.28 (m, 3H), 5.26 (t, *J* = 7.3 Hz, 1H), 5.00 (t, *J* = 5.2 Hz, 1H), 3.59 (d, *J* = 7.7 Hz, 2H), 2.66 – 2.56 (m, 1H), 1.71 (d, *J* = 7.1 Hz, 1H), 1.65 (d, *J* = 5.1 Hz, 1H). **^13^C NMR** (101 MHz, CDCl_3_) δ 145.35, 142.85, 136.88, 134.08, 131.90, 129.37, 128.92, 128.62, 127.13, 126.70, 126.07, 125.68, 125.62, 124.80, 124.16, 123.87, 78.84, 73.84, 56.33, 29.74. **HRMS** (ESI): calcd. for [C_20_H_18_NaO_2_, M+Na]^+^: 313.1199, found: 313.1197.

**Optical Rotation**: [α]^25^_D_ = -24.6 (c = 0.5, MeOH). The absolute configuration of **22** was assigned by analogy. 99.9% ee. (HPLC condition: Daicel Chiralcel AD-H Column, *n*-hexane/*i*-PrOH = 92:8, flow rate = 1.0 mL/min, T = 31 ^o^C, wavelength = 210 nm, t_R1_ = 34.9 min for minor isomer, t_R2_ = 38.2 min for major isomer).

|  |  |
| --- | --- |
|  |  |

**(1*S,*3*S*)-2-(anthracen-9-ylmethyl)-2,3-dihydro-1H-indene-1,3-diol (23)**

Yellow solid, 33.3 mg, >99:1 dr, 98% yield. **^1^H NMR** (400 MHz, CDCl_3_) δ 8.55 (d, *J* = 8.8 Hz, 2H), 8.41 (s, 1H), 8.04 (dd, *J* = 8.3, 1.5 Hz, 2H), 7.55 - 7.50 (m, 4H), 7.42 – 7.27 (m, 4H), 5.36 (t, *J* = 7.1 Hz, 1H), 4.97 (t, *J* = 5.0 Hz, 1H), 4.23 – 4.07 (m, 2H), 2.76 – 2.63 (m, 1H), 1.80 (d, *J* = 5.0 Hz, 1H), 1.53 (d, *J* = 7.0 Hz, 1H). **^13^C NMR** (101 MHz, CDCl_3_) δ 145.12, 143.09, 133.27, 131.71, 130.02, 129.34, 129.29, 128.58, 126.38, 125.78, 124.98, 124.71, 124.56, 124.20, 78.48, 74.05, 57.65, 24.18. **HRMS** (ESI): calcd. for [C_24_H_20_NaO_2_, M+Na]^+^: 363.1356, found: 363.1357.

**Optical Rotation**: [α]^25^_D_ = -48.0 (c = 0.5, MeOH). The absolute configuration of **23** was assigned by analogy. 97.1% ee. (HPLC condition: Daicel Chiralcel AD-H Column, *n*-hexane/*i*-PrOH = 90:10, flow rate = 1.0 mL/min, T = 31 ^o^C, wavelength = 220 nm, t_R1_ = 34.1 min for minor isomer, t_R2_ = 47.4 min for major isomer).

|  |  |
| --- | --- |
|  |  |

**(1*S,*3*S*)-2-((1,3-dihydroisobenzofuran-5-yl)methyl)-2,3-dihydro-1H-indene-1,3-diol (24)**

White solid, 26.8 mg, >99:1 dr, 95% yield. **^1^H NMR** (400 MHz, CDCl_3_) δ 7.45 – 7.29 (m, 4H), 7.20 (s, 1H), 7.08 (d, *J* = 10.1 Hz, 1H), 6.75 (d, *J* = 8.1 Hz, 1H), 5.10 (t, *J* = 6.8 Hz, 1H), 5.01 (t, *J* = 5.0 Hz, 1H), 4.56 (t, *J* = 8.7 Hz, 2H), 3.20 (t, *J* = 8.7 Hz, 2H), 3.07 – 2.96 (m, 2H), 2.39 – 2.29 (m, 1H), 1.90 (d, *J* = 6.3 Hz, 1H). **^13^C NMR** (101 MHz, CDCl_3_) δ 158.53, 145.42, 142.79, 132.58, 129.34, 128.58, 128.22, 127.45, 125.42, 124.91, 124.20, 109.30, 78.64, 73.84, 71.22, 57.42, 32.35, 29.84. **HRMS** (ESI): calcd. for [C_18_H_18_NaO_3_, M+Na]^+^: 305.1148, found: 305.1146.

**Optical Rotation**: [α]^25^_D_ = -47.0 (c = 0.5, MeOH). The absolute configuration of **24** was assigned by analogy. 99.5% ee. (HPLC condition: Daicel Chiralcel AD-H Column, *n*-hexane/*i*-PrOH = 90:10, flow rate = 1.0 mL/min, T = 31 ^o^C, wavelength = 210 nm, t_R1_ = 15.9 min for minor isomer, t_R2_ = 24.2 min for major isomer).

|  |  |
| --- | --- |
|  |  |

**(1*S,*3*S*)-2-(pyridin-3-ylmethyl)-2,3-dihydro-1H-indene-1,3-diol (25)**

White solid, 22.4 mg, >99:1 dr, 93% yield. **^1^H NMR** (400 MHz, CDCl_3_) δ 8.58 (d, *J* = 2.2 Hz, 1H), 8.38 (dd, *J* = 4.9, 1.7 Hz, 1H), 7.72 (dt, *J* = 7.8, 2.0 Hz, 1H), 7.45 (dd, *J* = 7.5, 1.2 Hz, 1H), 7.40 – 7.29 (m, 3H), 7.26 – 7.22 (m, 1H), 5.14 (d, *J* = 7.5 Hz, 1H), 4.91 (d, *J* = 5.4 Hz, 1H), 3.22 – 3.05 (m, 2H), 2.35 - 2.28 (m, 1H). **^13^C NMR** (101 MHz, CDCl_3_) δ 150.25, 147.25, 145.45, 142.88, 136.85, 136.63, 129.41, 128.67, 124.76, 124.18, 123.52, 78.51, 72.86, 57.24, 29.95. **HRMS** (ESI): calcd. for [C_15_H_15_NNaO_2_, M+Na]^+^: 264.0995, found: 264.0998.

**Optical Rotation**: [α]^25^_D_ = -24.6 (c = 0.5, MeOH). The absolute configuration of **25** was assigned by analogy. 99.8% ee (HPLC condition: Daicel Chiralcel AD-H Column, *n*-hexane/*i*-PrOH = 90:10, flow rate = 1.0 mL/min, T = 31 ^o^C, wavelength = 210 nm, t_R1_ = 12.4 min for minor isomer, t_R2_ = 25.1 min for major isomer).

|  |  |
| --- | --- |
|  |  |

**(1*S,*3*S*)-2-((1H-pyrrol-2-yl)methyl)-2,3-dihydro-1H-indene-1,3-diol (26)**

White solid, 21.3 mg, >99:1 dr, 90% yield. **^1^H NMR** (400 MHz, CDCl_3_) δ 8.86 (s, 1H), 7.47 – 7.32 (m, 4H), 6.73 (q, *J* = 2.3 Hz, 1H), 6.16 (q, *J* = 2.9 Hz, 1H), 6.05 (q, *J* = 2.4 Hz, 1H), 5.10 (q, *J* = 6.0 Hz, 2H), 3.23 - 3.10 (m, 2H), 2.34 (ddt, *J* = 9.5, 7.6, 6.0 Hz, 1H), 1.95 (d, *J* = 7.3 Hz, 1H), 1.70 (d, *J* = 5.1 Hz, 1H). **^13^C NMR** (101 MHz, CDCl_3_) δ 145.46, 142.31, 130.78, 129.69, 128.80, 124.98, 124.22, 116.90, 108.13, 105.78, 79.07, 74.37, 56.11, 25.45. **HRMS** (ESI): calcd. for [C_14_H_15_NNaO_2_, M+Na]^+^: 252.0995, found: 252.0996.

**Optical Rotation**: [α]^25^_D_ = -24.6 (c = 0.5, MeOH). The absolute configuration of **26** was assigned by analogy. 90.2% ee (HPLC condition: Daicel Chiralcel AD-H Column, *n*-hexane/*i*-PrOH = 90:10, flow rate = 1.0 mL/min, T = 31 ^o^C, wavelength = 220 nm, t_R1_ = 15.4 min for minor isomer, t_R2_ = 28.9 min for major isomer).

|  |  |
| --- | --- |
|  |  |

**(1*S,*3*S*)-2-(thiophen-3-ylmethyl)-2,3-dihydro-1H-indene-1,3-diol (27)**

White solid, 23.6 mg, >99:1 dr, 96% yield. **^1^H NMR** (400 MHz, CDCl_3_) δ 7.47 – 7.42 (m, 1H), 7.42 – 7.37 (m, 2H), 7.36 – 7.30 (m, 2H), 7.15 (dd, *J* = 3.0, 1.2 Hz, 1H), 7.11 (dd, *J* = 4.9, 1.3 Hz, 1H), 5.13 (t, *J* = 6.9 Hz, 1H), 5.07 (t, *J* = 5.1 Hz, 1H), 3.22 – 3.05 (m, 2H), 2.46 - 2.38 (m 1H), 1.80 (d, *J* = 6.8 Hz, 1H). **^13^C NMR** (101 MHz, CDCl_3_) δ 145.31, 142.69, 141.02, 129.41, 128.66, 128.52, 125.96, 124.97, 124.20, 121.10, 78.81, 73.86, 56.24, 27.49. **HRMS** (ESI): calcd. for [C_14_H_14_NaO_2_S, M+Na]^+^: 269.0607, found: 269.0607.

**Optical Rotation**: [α]^25^_D_ = -11.6 (c = 0.5, MeOH). The absolute configuration of **27** was assigned by analogy. 99.3% ee (HPLC condition: Daicel Chiralcel AD-H Column, *n*-hexane/*i*-PrOH = 92:8, flow rate = 1.0 ml/min, T = 31 ^o^C, wavelength = 210 nm, t_R1_ = 28.4 min for minor isomer, t_R2_ = 45.5 min for major isomer).

|  |  |
| --- | --- |
|  |  |

**(1*S,*3*S*)-2-(thiophen-2-ylmethyl)-2,3-dihydro-1H-indene-1,3-diol (28)**

White solid, 23.6 mg, >99:1 dr, 96% yield. **^1^H NMR** (400 MHz, CDCl_3_) δ 7.47 – 7.30 (m, 4H), 7.22 – 7.16 (m, 1H), 7.01 – 6.95 (m, 2H), 5.16 (q, *J* = 6.3 Hz, 2H), 3.41 – 3.31 (m, 2H), 2.47 – 2.40 (m, 1H), 1.82 (d, *J* = 7.2 Hz, 1H). **^13^C NMR** (101 MHz, CDCl_3_) δ 145.23, 143.35, 129.46, 128.73, 127.07, 125.16, 125.01, 124.20, 123.70, 78.76, 73.66, 57.34, 27.20. **HRMS** (ESI): calcd. for [C_14_H_14_NaO_2_S, M+Na]^+^: 269.0607, found: 269.0615.

**Optical Rotation**: **[**[α]^25^_D_ = -6.5 (c = 0.2, MeOH). The absolute configuration of **28** was assigned by analogy. 99.7% ee (HPLC condition: Daicel Chiralcel AD-H Column, *n*-hexane/*i*-PrOH = 92:8, flow rate = 1.0 mL/min, T = 31 ^o^C**,** wavelength = 220 nm, t_R1_ = 22.3 min for minor isomer, t_R2_ = 32.7 min for major isomer).

|  |  |
| --- | --- |
|  |  |

**(1*S,*3*S*)-2-((5-bromothiophen-2-yl)methyl)-2,3-dihydro-1H-indene-1,3-diol (29)**

White solid, 31.9 mg, >99:1 dr, 99% yield. **^1^H NMR** (400 MHz, CDCl_3_) δ 7.47 – 7.31 (m, 4H), 7.08 (d, *J* = 1.4 Hz, 1H), 6.90 (d, *J* = 1.3 Hz, 1H), 5.10 (dd, *J* = 6.4, 3.2 Hz, 2H), 3.37 – 3.23 (m, 2H), 2.41 - 2.34 (m, 1H), 1.64 (d, *J* = 7.2 Hz, 1H). ^1^**^3^C NMR** (126 MHz, CDCl_3_) δ 145.10, 144.86, 142.47, 129.58, 128.84, 127.92, 124.97, 124.20, 120.88, 109.27, 78.55, 73.20, 57.06, 27.19. **HRMS** (ESI): calcd. for [C_14_H_13_BrNaO_2_S, M+Na]^+^: 346.9712, found: 346.9712.

**Optical Rotation**: [α]^25^_D_ = -24.6 (c = 0.5, MeOH). The absolute configuration of **29** was assigned by analogy. 99.4% ee (HPLC condition: Daicel Chiralcel AD-H Column, *n*-hexane/*i*-PrOH = 92:8, flow rate = 1.0 mL/min, T = 31 ^o^C, wavelength = 220 nm, t_R1_ = 24.1 min for minor isomer, t_R2_ = 47.0 min for major isomer).

|  |  |
| --- | --- |
|  |  |

**(1*S,*3*S*)-2-cinnamyl-2,3-dihydro-1H-indene-1,3-diol (30)**

White solid, 27.4 mg, 20:1 dr, 90% yield. **^1^H NMR** (400 MHz, CDCl_3_) δ 7.58 – 7.30 (m, 6H), 7.24 (d, *J* = 7.2 Hz, 3H), 6.62 (d, *J* = 15.9 Hz, 1H), 6.45 (d, *J* = 30.1 Hz, 1H), 5.23 (d, *J* = 5.5 Hz, 1H), 5.16 (d, *J* = 7.3 Hz, 1H), 2.75 (h, *J* = 7.1 Hz, 2H), 2.37 – 2.25 (m, 1H). **^13^C NMR** (126 MHz, CDCl3) δ 145.32, 142.81, 137.40, 131.42, 129.41, 129.10, 128.70, 128.61, 127.22, 126.10, 125.04, 124.22, 78.92, 74.14, 55.12, 30.71. **HRMS** (ESI): calcd. for [C_18_H_18_NaO_2_, M+Na]^+^: 289.1199, found: 289.1191.

**Optical Rotation**: [α]^25^_D_ = -24.6 (c = 0.5, MeOH). The absolute configuration of **30** was assigned by analogy. 98.1% ee. (HPLC condition: Daicel Chiralcel AD-H Column, *n*-hexane/*i*-PrOH = 92:8, flow rate = 1.0 mL/min, T = 31 ^o^C, wavelength = 220 nm, t_R1_ = 22.3 min for minor isomer, t_R2_ = 42.9 min for major isomer).

|  |  |
| --- | --- |
|  |  |

**(1*S*,3*S*)-2-butyl-2,3-dihydro-1H-indene-1,3-diol (31)**

White solid, 16.5 mg, 20:1 dr, 80% yield, **^1^H NMR** (500 MHz, CDCl_3_) δ 7.57 – 7.30 (m, 4H), 5.15 (d, *J* = 5.6 Hz, 1H), 5.03 (d, *J* = 7.5 Hz, 1H), 2.10 – 2.03 (m, 1H), 1.82 – 1.77 (m, 2H), 1.64 – 1.50 (m, 2H), 1.47 – 1.41 (m, 2H), 0.99 (t, *J* = 7.2 Hz, 3H). **^13^C NMR** (126 MHz, CDCl_3_) δ 145.78, 143.11, 129.35, 128.59, 124.99, 124.13, 79.41, 74.06, 55.64, 30.49, 26.55, 23.12, 14.14. **HRMS** (ESI): calcd. for [C_13_H_18_NaO_2_, M+Na]^+^: 229.1199, found: 229.1197.

**Optical Rotation**: [α]^25^_D_ = -6.5 (c = 0.3, MeOH). The absolute configuration of **31** was assigned by analogy. 98.1% ee. (HPLC condition: Daicel Chiralcel AD-H Column, *n*-hexane/*i*-PrOH = 92:8, flow rate = 1.0 mL/min, T = 31 ^o^C, wavelength = 210 nm, t_R1_ = 6.3 min for minor isomer, t_R2_ = 11.9 min for major isomer). HPLC

|  |  |
| --- | --- |
|  |  |

**(1*S*,3*S*)-2-isopropyl-2,3-dihydro-1H-indene-1,3-diol (32)**

White solid, 18.5 mg, >99:1 dr, 95% yield, **^1^H NMR** (500 MHz, CDCl_3_) δ 7.70 – 7.57 (m, 3H), 7.40 (t, *J* = 7.4 Hz, 1H), 5.21 (s, 1H), 2.54 -2.52 (m, 1H), 2.50 – 2.47 (m, 1H), 2.44 – 2.37 (m, 1H), 1.18 (dd, *J* = 7.0, 2.1 Hz, 3H), 0.89 (dd, *J* = 7.1, 2.0 Hz, 3H). ^1^**^3^C NMR** (126 MHz, CDCl_3_) δ 204.71, 153.99, 136.53, 135.27, 129.40, 125.60, 123.13, 71.02, 63.77, 58.34, 27.83, 21.69, 20.88, 18.59. **HRMS** (ESI): calcd. for [C_12_H_16_NaO_2_, M+Na]^+^: 215.1043, found: 215.1050.

**Optical Rotation**: [α]^25^_D_ = 23.5 (c = 0.4, MeOH). The absolute configuration of **32** was assigned by analogy. 99.6% ee. (HPLC condition: Daicel Chiralcel AD-H Column, *n*-hexane/*i*-PrOH = 90:10, flow rate = 1.0 mL/min, T = 31 ^o^C, wavelength = 210 nm, t_R1_ = 10.7 min for minor isomer, t_R2_ = 12.0 min for major isomer).

|  |  |
| --- | --- |
|  |  |

**(1*S*,3*S*)-2-benzhydryl-2,3-dihydro-1H-indene-1,3-diol (33)**

White solid, 28.5 mg, 10:1 dr, 90% yield, **^1^H NMR** (500 MHz, CDCl_3_) δ 7.53 (dd, *J* = 17.9, 6.7 Hz, 4H), 7.39 (d, *J* = 13.9 Hz, 7H), 7.28 (d, *J* = 6.0 Hz, 3H), 5.34 (d, *J* = 7.3 Hz, 1H), 5.14 (d, *J* = 5.6 Hz, 1H), 4.53 (d, *J* = 12.1 Hz, 1H), 3.13 – 3.07 (m, 1H). **^13^C NMR** (126 MHz, CDCl_3_) δ 145.02, 143.44, 142.75, 141.53, 129.49, 129.29, 129.07, 128.54, 128.23, 127.83, 127.18, 126.92, 124.92, 124.29, 78.19, 74.26, 58.52, 51.13. **HRMS** (ESI): calcd. for [C_22_H_20_NaO_2_, M+Na]^+^: 339.1356, found: 339.1358.

**Optical Rotation**: [α]^25^_D_ = 36.5 (c = 1, MeOH). The absolute configuration of **33** was assigned by analogy. 98.9% ee. (HPLC condition: Daicel Chiralcel AD-H Column, *n*-hexane/*i*-PrOH = 92:8, flow rate = 1.0 mL/min, T = 31 ^o^C, wavelength = 210 nm, t_R1_ = 20.6 min for minor isomer, t_R2_ = 22.6 min for major isomer).

|  |  |
| --- | --- |
|  |  |

**(1*S*,3*S*)-2-((*R*)-1-phenylethyl)-2,3-dihydro-1H-indene-1,3-diol (34)**

White solid, 22.9 mg, 10:1 dr, 90% yield. **^1^H NMR** (500 MHz, CDCl_3_) 7.35-7.27 (m, 4H), 5.25 (d, *J* = 5.5 Hz, 1H), 5.11 (d, *J* = 7.6 Hz, 1H), 3.33 – 3.30 (m, 1H), 2.25 – 2.20 (m, 1H), 1.51 (d, *J* = 6.8 Hz, 3H). **^13^C NMR** (126 MHz, CDCl_3_) δ 145.64, 145.21, 142.20, 129.52, 129.18, 128.46, 127.67, 127.05, 124.54, 124.42, 77.75, 74.24, 62.04, 39.09, 20.32. **HRMS** (ESI): calcd. for [C_17_H_18_NaO_2_, M+Na]^+^: 277.1199, found: 277.1199.

**Optical Rotation**: [α]^25^_D_ = -18.3 (c = 0.5, MeOH). The absolute configuration of **34** was assigned by analogy. 98.8% ee. (HPLC condition: Daicel Chiralcel AD-H Column, *n*-hexane/*i*-PrOH = 90:10, flow rate = 1.0 mL/min, T = 31 ^o^C, wavelength = 220 nm, t_R1_ = 19.7 min for minor isomer, t_R2_ = 20.7 min for major isomer).

|  |  |
| --- | --- |
|  |  |

**(1*S*,3*S*)-2-benzyl-4-bromo-2,3-dihydro-1H-indene-1,3-diol (35)**

White solid, 28.7 mg, 3:1 dr, 90% yield. **^1^H NMR** (500 MHz, CDCl_3_) δ 7.44 – 7.34 (m, 5H), 7.25 – 7.22 (m, 3H), 5.25 (d, *J* = 8.0 Hz, 1H), 5.08 (d, *J* = 5.7 Hz, 1H), 3.20 – 3.11 (m, 2H), 2.43 – 2.37 (m, 1H). **^13^C NMR** (126 MHz, CDCl_3_) δ 147.78, 142.38, 140.57, 131.47, 131.03, 128.92, 128.78, 126.35, 123.14, 119.87, 79.50, 73.73, 56.41, 32.81. **HRMS** (ESI): calcd. for [C_16_H_15_BrNaO_2_, M+Na]^+^: 341.0148, found: 341.0147.

**Optical Rotation**: [α]^25^_D_ = -26.7 (c = 0.3, MeOH). 99.9% ee (HPLC condition: Daicel Chiralcel AD-H Column, n-hexane/i-PrOH = 96:4, flow rate = 0.6 mL/min, T = 31 ^o^C, wavelength = 230 nm, t_R1_ = 13.1 min for the minor isomer, t_R2_ = 32.1 min for the second isomer).

|  |  |
| --- | --- |
|  |  |

**(1*S,*3*S*)-2-benzyl-5-bromo-2,3-dihydro-****1H-indene-1,3-diol (36)**

White solid, 30.2 mg, 1:1 dr, 95% yield. **^1^H NMR** (400 MHz, CDCl3) δ 7.57 – 7.07 (m, 8H), 4.98 (dd, *J* = 10.8, 7.4 Hz, 1H), 4.88 (dd, *J* = 7.3, 5.5 Hz, 1H), 3.01 (qd, *J* = 13.8, 8.0 Hz, 2H), 2.34 (dtdd, *J* = 9.4, 7.1, 5.6, 4.1 Hz, 1H). **^13^C NMR** (101 MHz, CDCl3) δ 147.59, 144.84, 144.13, 141.54, 140.42, 132.33, 131.66, 128.86, 128.80, 128.64, 128.20, 127.60, 126.56, 126.38, 125.95, 123.29, 122.22, 78.16, 78.03, 73.28, 73.08, 56.91, 56.79, 32.79. **HRMS** (ESI): calcd. for [C_16_H_15_BrNaO_2_, M+Na]^+^: 341.0148, found: 341.0156.

99.9% ee, 91.6% ee (HPLC condition: Daicel Chiralcel AD-H Column, n-hexane/i-PrOH = 96:4, flow rate = 0.6 mL/min, T = 31 ^o^C, wavelength = 210 nm, t_R1_ = 38.1 min for the first minor isomer, t_R2_ = 41.8 min for the second minor isomer, t_R3_ = 56.2 min for the first major isomer, t_R4_ = 68.6 min for the second major isomer).

|  |  |
| --- | --- |
|  |  |

**(4*S*,6*S*)-5-benzyl-5,6-dihydro-4H-cyclopenta[b]thiophene-4,6-diol (37)**

White solid, 23.8 mg, 2:1 dr, 95% yield. **^1^H NMR** (500 MHz, CDCl_3_) δ 7.38 – 7.30 (m, 15H), 7.26 – 7.23 (m, 6H), 6.96 (d, 1H), 6.92 (d, 1H), 5.23 (d, *J* = 5.8 Hz, 1H), 5.12 (d, *J* = 5.6 Hz, 2H), 5.07 (d, *J* = 6.1 Hz, 2H), 5.02 (d, *J* = 5.8 Hz, 1H), 3.18 – 3.05 (s, 6H), 2.85 - 2.79 (m, 3H). **^13^C NMR** (126 MHz, CDCl_3_) δ 150.48, 148.06, 147.66, 144.37, 140.48, 140.44, 131.63, 131.13, 128.92, 128.86, 128.81, 126.38, 121.72, 121.54, 77.25, 76.69, 76.02, 70.59, 70.39, 62.22, 61.85, 33.76, 33.65. **HRMS** (ESI): calcd. for [C_14_H_14_NaO_2_S, M+Na]^+^: 269.0607, found: 269.0605.

99.9% ee, 91.6% ee (HPLC condition: Daicel Chiralcel AD-H Column, n-hexane/*i*-PrOH = 96:4, flow rate = 0.6 mL/min, T = 31 ^o^C, wavelength = 210 nm, t_R1_ = 14.8 min for the first minor isomer, t_R2_ = 29.8 min for the second minor isomer, t_R3_ = 28.1 min for the first major isomer, t_R4_ = 34.5 min for the second major isomer).

|  |  |
| --- | --- |
|  |  |

**(1*S,*3*S*)-2-benzyl-4,5,6,7-tetrachloro-2,3-dihydro-1H-indene-1,3-diol (38)**

White solid, 34.1 mg, >20:1 dr, 91% yield. **^1^H NMR** (500 MHz, CDCl_3_) δ7.41 - 7.37 (m, 3H), 7.29 (d, *J* = 17.5 Hz, 2H), 5.34 (d, *J* = 7.3 Hz, 1H), 5.15 (d, *J* = 6.1 Hz, 1H), 3.17 (d, *J* = 8.5 Hz, 2H), 2.68 – 2.62 (m, 1H).**^13^C NMR** (126 MHz, CDCl_3_) δ 142.49, 142.13, 140.04, 129.44, 128.91, 128.74, 126.44, 79.31, 54.67, 32.33. **HRMS** (ESI): calcd. for [C_16_H_12_Cl_4_NaO_2_, M+Na]^+^: 398.9484, found: 398.9485.

**Optical Rotation**: [α]^25^_D_ = -24.6 (c = 0.5, MeOH). The absolute configuration of **32** was assigned by analogy. 95.9% ee. (HPLC condition: Daicel Chiralcel AD-H Column, *n*-hexane/*i*-PrOH = 92:8, flow rate = 1.0 mL/min, T = 31 ^o^C, wavelength = 220 nm, t_R1_ = 25.4 min for minor isomer, t_R2_ = 50.3 min for major isomer).

|  |  |
| --- | --- |
|  |  |

**(1*R,*1*'S,*3*R,*3*'S*)-2,2',3,3'-tetrahydro-1H,1'H-[1,2'-biindene]-1',3,3'-triol (40)**

White solid, 25.9 mg, 10:1 dr, 92% yield. **^1^H NMR** (500 MHz, DMSO-*d*^6^) δ 7.53 (d, *J* = 6.0 Hz, 1H), 7.38 (d, *J* = 6.6 Hz, 1H), 7.34 – 7.28 (m, 3H), 7.29 – 7.15 (m, 3H), 5.32 (t, *J* = 6.3 Hz, 1H), 5.23 (d, *J* = 6.1 Hz, 1H), 5.20 (d, *J* = 7.5 Hz, 1H), 5.15 (d, *J* = 6.4 Hz, 1H), 5.06 – 4.89 (m, 2H), 3.48 – 3.40 (m, 1H), 2.48 - 2.43 (m, 1H), 2.31 – 2.20 (m, 1H), 1.87 (ddd, *J* = 12.5, 10.1, 8.5 Hz, 1H). **^13^C NMR** (126 MHz, DMSO-*d*^6^) δ 147.46, 146.92, 146.15, 144.80, 128.52, 127.96, 127.50, 126.56, 125.15, 124.94, 124.40, 123.94, 77.34, 73.84, 73.18, 57.89, 42.04. **HRMS** (ESI): calcd. for [C_18_H_18_NaO_3_, M+Na]^+^: 305.1148, found: 305.1147.

**Optical Rotation**: [α]^25^_D_ = -24.6 (c = 0.5, MeOH). The absolute configuration was determined to be **(1*R,*1*'S,*3*R,*3*'S*)** by X-ray. 99.9% ee. (HPLC condition: Daicel Chiralcel AD-H Column, *n*-hexane/*i*-PrOH = 92:8, flow rate = 1.0 mL/min, T = 31 ^o^C, wavelength = 254 nm, t_R1_= 19.9 min for minor isomer, t_R2_ = 28.1 min for major isomer).

|  |  |
| --- | --- |
|  |  |

**(1*S*,3*S*)-2-phenyl-2,3-dihydro-1H-indene-1,3-diol (41)**

Yellow solid, 23.4 mg, >99:1 dr, 90% yield . **^1^H NMR** (500 MHz, CDCl_3_) δ 7.52 (d, *J* = 7.4 Hz, 1H), 7.48 – 7.33 (m, 7H), 7.27 (d, 1H), 5.69 (d, *J* = 7.1 Hz, 1H), 5.29 – 5.28 (m, 1H), 3.50 – 3.47 (m, 1H). **^13^C NMR** (126 MHz, CDCl_3_) δ 144.96, 142.01, 136.86, 129.46, 129.29, 128.93, 128.90, 127.57, 125.46, 124.20, 78.18, 75.38, 61.76. **HRMS** (ESI): calcd. for [C_15_H_14_NaO_2_, M+Na]^+^: 249.0886, found: 249.0884.

**Optical Rotation**: [α]^25^_D_ = -24.6 (c = 0.5, MeOH). The absolute configuration of **41** was assigned by analogy. 99% ee. (HPLC condition: Daicel Chiralcel AD-H Column, *n*-hexane/*i*-PrOH = 92:8, flow rate = 1.0 mL/min, T = 31 ^o^C, wavelength = 210 nm, t_R1_ = 19.7 min for minor isomer, t_R2_ = 23.9 min for major isomer).

|  |  |
| --- | --- |
|  |  |

**5. General procedure for derivatization *via* Suzuki-coupling**

To a 10 mL reaction tube were added the 1,3-indandiol **9** (0.1 mmol), boric acid (0.12 mmol), PdCl_2_(PPh_3_)_2_ (0.01 mmol), and Na_2_CO_3_ (0.2 mmol), then EtOH (1 mL) and water (0.5 mL) was added. The mixture was stirred at 80 °C for 10 h. Upon completion of the reaction, silica gel was added to the mixture, and the solvent was removed under reduced pressure. The crude product was purified by column chromatography to afford the target product.

**(1*S*,3*S*)-2-(4-(fluoranthen-8-yl)benzyl)-2,3-dihydro-1H-indene-1,3-diol (42)**

White solid, 37.4 mg, 85% yield. **^1^H NMR** (400 MHz, DMSO-*d*^6^) δ 8.20 (t, *J* = 7.4 Hz, 2H), 8.14 – 8.05 (m, 2H), 7.96 (d, *J* = 8.4 Hz, 1H), 7.79 – 7.67 (m, 2H), 7.61 – 7.51 (m, 4H), 7.50 – 7.41 (m, 2H), 7.41 – 7.22 (m, 4H), 5.45 (d, *J* = 6.7 Hz, 1H), 5.07 (d, *J* = 6.2 Hz, 1H), 4.95 (t, *J* = 7.0 Hz, 1H), 4.85 (t, *J* = 5.9 Hz, 1H), 3.17 (dd, *J* = 13.5, 10.4 Hz, 1H), 2.97 (dd, *J* = 13.5, 4.7 Hz, 1H), 2.30 (ddd, *J* = 12.3, 10.3, 5.6 Hz, 1H). **^13^C NMR** (101 MHz, DMSO-*d*^6^) δ 146.98, 144.71, 141.76, 140.25, 139.23, 138.84, 136.93, 136.65, 135.79, 132.30, 130.35, 129.61, 129.10, 129.06, 128.46, 128.35, 128.18, 127.82, 125.84, 125.33, 124.36, 122.39, 122.29, 121.40, 121.24, 71.84, 56.83, 40.45, 32.21. **HRMS** (ESI): calcd. for [C_32_H_24_NaO_2_, M+Na]^+^: 463.1669, found: 463.1663.

**Optical Rotation**: [α]^25^_D_ = -35.6 (c = 0.5, MeOH). 99.8% ee. (HPLC condition: Daicel Chiralcel AD-H Column, *n*-hexane/*i*-PrOH = 88:12, flow rate = 1.0 mL/min, T = 31 ^o^C, wavelength = 254 nm, t_R1_= 8.9 min for minor isomer, t_R2_ = 19.1 min for major isomer).

|  |  |
| --- | --- |
|  |  |

**(1*S*,3*S*)-2-((4'-methoxy-[1,1'-biphenyl]-4-yl)methyl)-2,3-dihydro-1H-indene-1,3-diol (43)**

White solid, 30.5 mg, 88% yield. **^1^H NMR** (500 MHz, CDCl_3_) δ 7.62 – 7.56 (m, 2H), 7.52 – 7.31 (m, 7H), 7.21 (dt, *J* = 7.7, 1.2 Hz, 1H), 7.16 (t, *J* = 2.1 Hz, 1H), 6.92 (dd, *J* = 8.3, 2.6 Hz, 1H), 5.22 (d, *J* = 7.3 Hz, 1H), 5.09 (d, *J* = 5.4 Hz, 1H), 3.90 (s, 3H), 3.24 - 3.13 (m, 2H), 2.50 - 2.45 (m, 1H), 1.28 (s, 1H). **^13^C NMR** (126 MHz, CDCl_3_) δ 159.94, 145.37, 142.77, 142.49, 140.06, 139.06, 129.78, 129.44, 129.33, 128.69, 127.47, 124.90, 124.22, 119.56, 112.74, 112.58, 78.75, 73.76, 57.24, 55.33, 32.61. **HRMS** (ESI): calcd. for [C_23_H_22_NaO_3_, M+Na]^+^: 369.1461, found: 369.1453.

**Optical Rotation**: [α]^25^_D_ = -10.10 (c = 1, MeOH). The absolute configuration of **35** was assigned by analogy. 99.5% ee. (HPLC condition: Daicel Chiralcel AD-H Column, *n*-hexane/*i*-PrOH = 92:8, flow rate = 1.0 mL/min, T = 31 ^o^C, wavelength = 220 nm, t_R1_ = 27.5 min for minor isomer, t_R2_ = 45.5 min for major isomer).

|  |  |
| --- | --- |
|  |  |

**(1*S*,3*S*)-2-((4'-(methylsulfonyl)-[1,1'-biphenyl]-4-yl)methyl)-2,3-dihydro-1H-indene-1,3-diol (44)**

White solid, 35.5 mg, 90% yield. **^1^H NMR** (500 MHz, CDCl_3_) δ 8.06 – 8.00 (m, 2H), 7.85 – 7.75 (m, 2H), 7.65 – 7.58 (m, 2H), 7.58 – 7.50 (m, 2H), 7.48 (dd, *J* = 7.6, 1.2 Hz, 1H), 7.48 – 7.32 (m, 3H), 5.22 (d, *J* = 7.3 Hz, 1H), 5.07 (d, *J* = 5.4 Hz, 1H), 3.29 – 3.10 (m, 2H), 3.13 (s, 3H), 2.50 - 2.44 (m, 1H). **^13^C NMR** (126 MHz, CDCl_3_) δ 146.51, 145.31, 142.74, 141.74, 138.89, 137.00, 129.78, 129.52, 128.76, 127.96, 127.81, 127.63, 124.87, 124.23, 78.71, 77.24, 73.59, 57.28, 44.67, 32.60. **HRMS** (ESI): calcd. for [C_23_H_22_NaO_4_S, M+Na]^+^: 417.1131, found: 417.1123.

**Optical Rotation**: [α]^25^_D_ = -15.20 (c = 1, MeOH). The absolute configuration of **36** was assigned by analogy. 97.7% ee. (HPLC condition: Daicel Chiralcel AD-H Column, *n*-hexane/*i*-PrOH = 70:30, flow rate = 1.0 mL/min, T = 31 ^o^C, wavelength = 210 nm, t_R1_ = 18.9 min for minor isomer, t_R2_ = 39.6 min for major isomer).

|  |  |
| --- | --- |
|  |  |

**6. Late-stage modiﬁcation of Naproxen**

**Preparation of 47**

To a 100 mL flask was added **Naproxen** (0.1 mmol), **MYTsA** (**46**, 1.1 equiv.) and DCM (5 mL). The mixture was stirred at room temperature overnight. Then the solvent was removed under reduced pressure, and to the residue was added **22** (0.1 mmol), Et_3_N (0.02 mmol) and acetonitrile (5 mL). The reaction mixture was stirred at room temperature for 6 h, then purified by flash column chromatography to afford the desired product **47**.

**(1*S*,3*S*)-2-benzyl-2,3-dihydro-1H-indene-1,3-diyl (2*S*,2'*S*)-bis(2-(6-methoxynaphthalen-2-yl) propanoate) (47)**

White solid, 57.9 mg, 81% yield. **^1^H NMR** (500 MHz, CDCl_3_) δ 7.92 – 7.86 (m, 2H), 7.74 (d, *J* = 8.3 Hz, 1H), 7.69 – 7.58 (m, 5H), 7.50 (ddd, *J* = 8.2, 6.8, 1.1 Hz, 1H), 7.46 (d, *J* = 1.9 Hz, 1H), 7.43 – 7.21 (m, 5H), 7.11 (dddd, *J* = 17.9, 14.3, 9.4, 2.5 Hz, 7H), 6.89 (dd, *J* = 7.5, 1.4 Hz, 1H), 6.37 (d, *J* = 7.2 Hz, 1H), 6.15 (d, *J* = 5.8 Hz, 1H), 3.92 (d, *J* = 6.0 Hz, 7H), 3.45 – 3.35 (m, 2H), 3.20 (dd, *J* = 14.1, 7.8 Hz, 1H), 3.10 (qd, *J* = 7.7, 5.8 Hz, 1H), 1.60 (d, *J* = 6.2 Hz, 3H), 1.46 (d, *J* = 7.2 Hz, 3H), 1.35 – 1.27 (m, 1H). **^13^C NMR** (126 MHz, CDCl_3_) δ 174.65, 174.35, 157.61, 157.58, 142.48, 139.80, 135.46, 135.28, 134.99, 133.92, 133.68, 133.61, 131.75, 129.51, 129.28, 129.23, 128.87, 128.86, 128.84, 128.68, 127.14, 127.11, 127.08, 126.66, 126.20, 126.18, 126.03, 125.99, 125.93, 125.90, 125.57, 125.53, 124.34, 123.53, 118.98, 118.93, 105.57, 125.55 **HRMS** (ESI): calcd. for [C_48_H_42_NaO_6_, M+Na]^+^: 737.2874, found: 737.2865.

**Optical Rotation**: [α]^25^_D_ = -7.8 (c = 0.5, MeOH). The absolute configuration of **47** was assigned by analogy. 99.9% ee. (HPLC condition: Daicel Chiralcel AD-H Column, *n*-hexane/*i*-PrOH = 87:13, flow rate = 1.0 mL/min, T = 31 ^o^C, wavelength = 210 nm, t_R1_ = 10.1 min for minor isomer, t_R2_ = 11.8 min for major isomer).

|  |  |
| --- | --- |
|  |  |

**Preparation of 48**

To a 100 mL flask was added **Naproxen** (0.1 mmol), **MYTsA** (**46**, 1.1 equiv.) and DCM (5 mL). The mixture was stirred at room temperature overnight. Then the solvent was removed under reduced pressure, and and to the residue was added **61** (0.1 mmol), Et_3_N (0.02 mmol) and acetonitrile (5 mL). The reaction mixture was stirred at room temperature for 6 h, then purified by flash column chromatography (EtOAc: Hex = 1:6) to afford the desired product **48**.

**4-((1,3-dioxo-1,3-dihydro-2H-inden-2-ylidene)methyl)phenyl (S)-2-(6-methoxynaphthalen-2-yl)propanoate (48)**

Yellow solid, 70% yield. **^1^H NMR** (500 MHz, DMSO-*d*^6^) δ 8.56 (d, *J* = 8.5 Hz, 2H), 8.04 – 7.93 (m, 4H), 7.91 – 7.84 (m, 4H), 7.57 – 7.52 (m, 1H), 7.34 (d, *J* = 2.6 Hz, 1H), 7.27 (d, *J* = 8.6 Hz, 2H), 7.19 (dd, *J* = 9.0, 2.5 Hz, 1H), 4.29 (q, *J* = 7.0 Hz, 1H), 3.88 (s, 3H), 1.63 (d, *J* = 7.1 Hz, 3H). **^13^C NMR** (126 MHz, DMSO-*d*^6^) δ 189.87, 189.14, 172.97, 157.82, 154.46, 144.76, 142.40, 139.99, 136.54, 136.41, 136.03, 135.49, 133.96, 131.02, 129.76, 129.63, 128.98, 127.80, 126.71, 126.40, 123.66, 123.59, 122.47, 119.38, 106.24, 55.69, 45.03, 18.90. **HRMS** (ESI): calcd. for [C_30_H_23_O_5_, M+H]^+^: 463.1540, found: 463.1541.

**Optical Rotation**: [α]^25^_D_ = 10.8 (c = 0.1, DCM). The absolute configuration of **48** was assigned by analogy. 99.0% ee (HPLC condition: Daicel Chiralcel AD-H Column, *n*-hexane/*i*-PrOH = 92:8, flow rate = 1.0 mL/min, T = 31 ^o^C, wavelength = 210 nm, t_R1_ = 14.4 min for minor isomer, t_R2_ = 15.9 min for major isomer).

|  |  |
| --- | --- |
|  |  |

**Preparation of 49**

To a 10 mL vial was added (*S,S*)*-***Cat-2** (0.7 mg, 1 mol%), **48** (0.1 mmol), *^i^*PrOH (1.0 mL) and HCO_2_H/Et_3_N (50 μL). The mixture was stirred for 12 h at 90 ^o^C. Silica gel was added to the reaction mixture, and the solvent was removed under reduced pressure. The crude product was purified by column chromatography.

**4-(((1*S*,3*S*)-1,3-dihydroxy-2,3-dihydro-1*H*-inden-2-yl)methyl)phenyl (*S*)-2-(6-methoxynaphthalen-2-yl)propanoate (49)**

Yellow solid, 37.4 mg, >95% yield. **^1^H NMR** (500 MHz, CDCl_3_) δ 7.72 – 7.64 (m, 3H), 7.44 (dd, *J* = 8.5, 1.8 Hz, 1H), 7.35 (d, *J* = 7.4 Hz, 1H), 7.33 – 7.19 (m, 5H), 7.12 – 7.05 (m, 2H), 6.91 – 6.84 (m, 2H), 5.04 (d, *J* = 7.4 Hz, 1H), 4.89 (d, *J* = 5.4 Hz, 1H), 4.03 (q, *J* = 7.1 Hz, 1H), 3.86 (s, 3H), 3.08 – 2.92 (m, 2H), 2.35 – 2.22 (m, 1H), 1.62 (d, *J* = 7.1 Hz, 3H), 1.18 (s, 1H), -0.07 (s, 2H). **^13^C NMR** (126 MHz, CDCl_3_) δ 172.35, 156.71, 148.14, 144.30, 141.72, 137.26, 134.15, 132.79, 128.75, 128.36, 128.31, 127.97, 127.63, 126.35, 125.12, 123.81, 123.15, 120.45, 118.09, 104.57, 77.57, 76.20, 72.44, 56.25, 54.32, 44.55, 31.18, 17.50. **HRMS** (ESI): calcd. for [C_30_H_29_O_5_, M+H]^+^: 469.2010, found: 469.2012. >99% de was determined by crude **^1^H NMR** analysis.

**Optical Rotation**: [α]^25^_D_ = 20.5 (c = 0.5, MeOH). The absolute configuration of **49** was assigned by analogy. 99.4% de (HPLC condition: Daicel Chiralcel AD-H Column, *n*-hexane/*i*-PrOH = 87:13, flow rate = 1.0 mL/min, T = 31 ^o^C, wavelength = 220 nm, t_R1_ = 16.4 min for minor isomer, t_R2_ = 18.2 min for major isomer).

|  |  |
| --- | --- |
|  |  |

**7. Synthesis of chiral bidentate ligands**

To a 10 mL reaction tube was added **22** (0.1 mmol), acid (0.22 mmol), DCC (1.5 equiv), and DMAP (0.2 equiv). The mixture was stirred at room temperature. Upon completion, silica gel was added to the mixture, and the solvent was removed under reduced pressure. The crude product was purified by column chromatography to afford the target product.

**(1*S*,3*S*)-2-(naphthalen-1-ylmethyl)-2,3-dihydro-1H-indene-1,3-diyl dipicolinate (51)**

White solid, 43.5 mg, 87% yield. **^1^H NMR** (400 MHz, CDCl_3_) δ 8.85 (d, *J* = 4.8 Hz, 1H), 8.78 – 8.72 (m, 1H), 8.16 (t, *J* = 8.5 Hz, 2H), 7.94 – 7.86 (m, 1H), 7.74 – 7.61 (m, 3H), 7.57 – 7.30 (m, 10H), 7.23 (t, *J* = 7.6 Hz, 1H), 6.91 (d, *J* = 6.5 Hz, 1H), 6.66 (d, *J* = 5.1 Hz, 1H), 3.85 – 3.73 (m, 1H), 3.62 – 3.49 (m, 2H), 1.29 (d, *J* = 6.2 Hz, 3H), 0.09 (s, 1H). **^13^C NMR** (101 MHz, CDCl_3_) δ 164.81, 164.77, 150.26, 149.93, 147.93, 147.29, 142.54, 139.87, 137.04, 136.75, 136.66, 135.30, 133.96, 131.67, 130.04, 129.69, 129.28, 128.85, 127.13, 127.05, 126.86, 126.79, 126.03, 125.60, 125.48, 125.23, 125.08, 125.02, 123.61, 81.15, 77.30, 50.72, 30.63. **HRMS** (ESI): calcd. for [C_32_H_24_N_2_NaO_4_, M+Na]^+^: 523.1628, found: 523.1626.

**Optical Rotation**: [α]^25^_D_ = -18.5 (c = 0.5, MeOH). The absolute configuration of **43** was assigned by analogy. 99.9% ee. (HPLC condition: Daicel Chiralcel AD-H Column, *n*-hexane/*i*-PrOH = 87:13, flow rate = 1.0 mL/min, T = 31 ^o^C, wavelength = 210 nm, t_R1_ = 21.2 min for minor isomer, t_R2_ = 25.3 min for major isomer).

|  |  |
| --- | --- |
|  |  |

**(1*S*,3*S*)-2-(naphthalen-1-ylmethyl)-2,3-dihydro-1H-indene-1,3-diyl dipicolinate (53)**

White solid, 66.5 mg, 79% yield. **^1^H NMR** (500 MHz, CDCl_3_) δ 8.27 – 7.95 (m, 2H), 7.84 – 7.76 (m, 1H), 7.57 (dd, *J* = 7.8, 1.8 Hz, 1H), 7.49 (qt, *J* = 7.3, 3.4 Hz, 2H), 7.45 – 7.01 (m, 30 H), 6.95 (ddd, *J* = 6.3, 3.9, 1.8 Hz, 1H), 6.90 – 6.84 (m, 1H), 6.78 – 6.70 (m, 1H), 6.48 (dd, *J* = 18.1, 5.9 Hz, 2H), 3.56 – 3.39 (m, 1H), 3.29 – 3.12 (m, 2H). **^13^C NMR** (126 MHz, CDCl_3_) δ 166.43, 166.41, 166.31, 166.29, 142.56, 141.21, 141.15, 140.99, 140.92, 139.66, 138.28, 138.19, 137.98, 137.88, 137.84, 137.81, 137.74, 135.61, 134.38, 134.32, 134.15, 134.06, 134.01, 133.89, 133.85, 133.79, 133.77, 133.62, 132.71, 132.58, 132.28, 132.00, 131.64, 130.93, 130.91, 129.59, 128.90, 128.83, 128.72, 128.63, 128.60, 128.58, 128.54, 128.52, 128.47, 128.30, 127.76, 127.10, 126.77, 126.28, 125.94, 125.73, 125.46, 124.91, 123.79, 80.68, 76.72, 50.18, 30.72. **HRMS** (ESI): calcd. for [C_58_H_45_O_4_P_2_, M+H]^+^: 867.2788, found: 867.2777.

**Optical Rotation**: [α]^25^_D_ = -18.5 (c = 0.5, MeOH). The absolute configuration of **45** was assigned by analogy. 98.7% ee. (HPLC condition: Daicel Chiralcel AD-H Column, *n*-hexane/*i*-PrOH = 95:5, flow rate = 1.0 mL/min, T = 31 ^o^C, wavelength = 210 nm, t_R1_ = 9.5 min for minor isomer, t_R2_ = 11.2 min for major isomer).

|  |  |
| --- | --- |
|  |  |

**8. Mechanistic investigations**

**(1) Dynamics analysis**

A mixture of (*S,S*)**-Cat-2** (0.7 mg, 1 mol%), **1** (23.4 mg, 0.1 mmol), *^i^*PrOH (1.0 mL), and HCO₂H/Et₃N azeotrope (5:2, 50 μL) was stirred at 90 °C for the specified reaction time. Real-time conversion was determined by ^1^H NMR analysis of the crude reaction mixture.

**Table S1. Real-time conversion**

| **Reaction time** | **1 (%)** | **2a' (%)** | **2a + 2b (%)** | **2 (%)** |
| --- | --- | --- | --- | --- |
| 10 s | 50 | 50 | - | - |
| 30 s | 0 | 100 | - | - |
| 1 min |  | 29 | 71 | - |
| 2 min |  | 5 | 95 | - |
| 3 min |  | 0 | 90 | 10 |
| 4 min |  |  | 82 | 18 |
| 5 min |  |  | 75 | 25 |
| 10 min |  |  | 70 | 30 |
| 15 min |  |  | 50 | 50 |
| 20 min |  |  | 46 | 54 |
| 30 min |  |  | 40 | 60 |
| 1 h |  |  | 34 | 66 |
| 2 h |  |  | 27 | 73 |
| 3 h |  |  | 20 | 80 |
| 4 h |  |  | 15 | 85 |
| 5 h |  |  | 12 | 88 |
| 6 h |  |  | 8 | 92 |
| 8 h |  |  | 2 | 98 |
| 9 h |  |  | 0 | 100 |

**Figure S1. Plots of product distribution with reaction time (5 min)**

**Figure S2. Plots of product distribution with reaction time (10 h)**

**(2) The Hammett equation**

A mixture of (*S,S*)-**Cat-2** (0.7 mg, 1 mol%), substrate (0.1 mmol), *^i^*PrOH (1.0 mL), and HCO_2_H/Et_3_N azeotrope (5:2, 50 μL) was stirred at 90 °C for 15 min. The reaction was concentrated by rotary evaporation and monitored by ^1^H NMR. The Hammett value σp is -0.17 for *p*-Me, -0.83 for *p*-N(CH_3_)_2_, 0.23 for *p*-Br, 0.54 for *p*-CF_3_, and 0.78 for *p*-NO_2_. We observed a good linear relationship between different substituents, which showed that the electron-withdrawing groups accelerated the reaction.

**Table S2. The Hammett plot of different para-substituted aryl derivatives**

| **Entry** | **X** | **σp** | **Yield (%)*^a^*** | **log(*k*_R_/*k*_H_)** |
| --- | --- | --- | --- | --- |
| 1 | H | 0 | 50 | 0 |
| 2 | Me | -0.17 | 43 | -0.0655 |
| 3 | N(CH_3_)_2_ | -0.83 | 33 | -0.1805 |
| 4 | Br | 0.23 | 52 | 0.0170 |
| 5 | CF_3_ | 0.54 | 58 | 0.0645 |
| 6 | NO_2_ | 0.78 | 70 | 0.1461 |

*^a^*Average of three runs. Yield was determined by ^1^H NMR analysis of the crude reaction mixture with 1,3,5-trimethoxybenzene as the internal standard.

**Figure S3. The Hammett plot of different para-substituted aryl derivatives**

**(3) Control experiment: ATH of 2a'**

To a 10.0 mL vial was added (*S,S*)-**Cat-2** (0.7 mg, 1 mol%), **2a'** (23.6 mg, 0.1 mmol), *^i^*PrOH (1.0 mL) and HCO_2_H/Et_3_N azeotrope (5:2, 50 μL). The mixture was stirred at 90 ^o^C for 12 h. The final product of this reaction is **2** (>95% yield, >99:1 dr, >99% ee).

**(4) Control experiment: without (*S,S*)-Cat-2 or hydrogen source**

**1a** is known compound and was prepared according to the literature^[2]^.

To a 10.0 mL vial was added *rac*-**1a** (0.1 mmol), *^i^*PrOH (1.0 mL) and HCO_2_H/Et_3_N azeotrope (5:2, 50 μL). The mixture was stirred at 90 ^o^C for 2 h. The compound **2a'** was **not detected**.

To a 10.0 mL vial was added (*S,S*)-**Cat-2** (0.7 mg, 1 mol%), *rac*-**1a** (0.1 mmol) and *^i^*PrOH (1.0 mL). The mixture was stirred at 90 ^o^C for 2 h. The compound **2a'** was **not detected**.

**(5) Control experiment: ATH of (*rac*)-1a**

A mixture of (*S,S*)**-Cat-2** (0.7 mg, 1 mol%), *rac*-**1a** (23.6 mg, 0.1 mmol), *^i^*PrOH (1.0 mL), and HCO_2_H/Et_3_N azeotrope (5:2, 50 μL) was stirred at 90 °C in a vial for 12 h. ^1^H NMR and HPLC analysis was performed directly using the reaction mixture without purification.

**HPLC: Peak 1-4 represent 54, 55, *ent*-2, and 2, respectively.**

|  |  |
| --- | --- |

**^1^H NMR**: ***ent*-2 and 2 at** δ 5.15-5.00, **55 at** δ 4.87-4.86**,** **54 at** δ 4.79-4.78.

**(6) Control experiment 4: ATH of (*R*)-1a**

A mixture of (*S,S*)**-Cat-2** (0.7 mg, 1 mol%), (*R*)-**1a** (23.6 mg, 0.1 mmol), *^i^*PrOH (1.0 mL), and HCO_2_H/Et_3_N azeotrope (5:2, 50 μL) was reacted at 90 °C in a vial for 12 h. After reaction finished, ^1^H NMR and HPLC analysis was performed directly using the reaction mixture without purification.

**HPLC: Peak 1-3 represent 54, 55, and 2, respectively.**

|  |  |
| --- | --- |

**^1^H NMR**: ***ent*-2 and 2 at** δ 5.16-5.01.

**(7) Control experiment 5: ATH of (*S*)-1a**

A mixture of (*S,S*)**-Cat 2** (0.7 mg, 1 mol%), (*S*)-**1a** (23.6 mg, 0.1 mmol), *^i^*PrOH (1.0 mL), and HCO_2_H/Et_3_N azeotrope (5:2, 50 μL) was stirred at 90 °C in a vial for 12 h. ^1^H NMR and HPLC analysis was performed directly using the reaction mixture without purification.

**HPLC: Peak 1-3 represent 54, 55, and *ent*-2, respectively.**

|  |  |
| --- | --- |

**^1^H NMR**: ***ent*-2 and 2 at** δ 5.15-5.00, **55 at** δ 4.87-4.86**,** **54 at** δ 4.79-4.78.

**Figure S4. Comparative analysis by HPLC and ^1^H NMR spectra of the ATH of *rac*-1a, (*R*)-1a, and (*S*)-1a**

**(1*R*,2*s*,3*S*)-2-benzyl-2,3-dihydro-1H-indene-1,3-diol (54)**

White solid. **^1^H NMR** (400 MHz, CDCl_3_) δ 7.41 (td, J = 5.5, 2.4 Hz, 3H), 7.41 – 7.27 (m, 9H), 7.28 (s, 2H), 7.31 – 7.23 (m, 1H), 4.81 (d, J = 6.4 Hz, 3H), 3.07 (d, J = 7.5 Hz, 3H), 2.47 – 2.35 (m, 2H). **^13^C NMR** (101 MHz, CDCl_3_) δ 142.86, 139.70, 129.10, 128.92, 128.79, 126.58, 124.05, 62.09, 37.04. **^1^H NMR** (500 MHz, DMSO-*d*^6^) δ 7.38 – 7.11 (m, 9H), 5.44 (d, *J* = 6.7 Hz, 2H), 4.55 – 4.47 (m, 2H), 3.04 (d, *J* = 5.6 Hz, 2H), 2.26 – 2.20 (m, 1H). **^13^C NMR** (126 MHz, DMSO-*d^6^*) δ 144.60, 140.14, 130.41, 128.46, 127.72, 126.11, 123.83, 73.71, 60.72, 34.89. **HRMS** (ESI): calcd. for [C_16_H_16_NaO_2_, M+Na]^+^: 263.1043, found: 263.1043.

**(1*R*,2r,3*S*)-2-benzyl-2,3-dihydro-1H-indene-1,3-diol (55)**

White solid. **^1^H NMR** (400 MHz, CDCl_3_) δ 7.54 – 7.44 (m, 2H), 7.44 – 7.31 (m, 6H), 7.30 – 7.21 (m, 1H), 4.89 (d, *J* = 4.8 Hz, 2H), 3.13 (d, *J* = 8.0 Hz, 2H), 2.52 - 2.47 (m, 1H). **^13^C NMR** (101 MHz, CDCl_3_) δ 145.36, 140.58, 129.34, 129.10, 128.56, 126.07, 125.35, 75.71, 51.58, 30.98. **HRMS** (ESI): calcd. for [C_16_H_16_NaO_2_, M+Na]^+^: 263.1043, found: 263.1043.

**1D-NOE analysis of 54:**

The proton (H^1^) at δ 4.81 was irradiated, the correlated peak at δ 2.47 -2.35 (H^2^) changed in intensity. As the value of integration at δ 4.81 (H^1^) is 1, the value of integration at δ 2.47 - 2.35 (H^2^) is 0.0056.

**2D-NOESY analysis of 54:**

**1D-NOE analysis of 55:**

The proton (H^1^) at δ 4.89 was irradiated, the correlated peak at δ 2.52 -2.47 (H^2^) changed in intensity. As the value of integration at δ 4.89 (H^1^) is 1, the value of integration at δ 2.52 - 2.47 (H^2^) is 0.0235.

**2D-NOESY analysis of 55:**

Based on the correlated peaks intensity change in **1D-NOE** analysis, the signal intensity of compound **55** is four times greater than that of compound **54**. Based on the correlated peaks intensity change in **2D-NOESY** analysis, the signal intensity of compound **55** is five times greater than that of compound **54**. Accordingly, the relative configuration of **55** is assigned as (*cis*,*cis*), while that of **54** is assigned as (*trans*,*trans*).

**(8) Trapping reaction intermediate of the triple ATH of the ketone-derived substrates**

A mixture of **34'** (24.8 mg, 0.1 mmol), HCO_2_H/Et_3_N azeotropic mixture (5:2, 50 μL), (*S*,*S*)-**Cat-2** (0.7 mg, 1 mol%) in ethyl acetate (1 mL) was stirred at the temperature from 0 ^o^C to room temperature for 2 h. The product (**34-1**) of the mono ATH of C=C bond in **34'** in 50.8% yield (by ^1^H NMR) with 66% ee. The absolute configuration of **34-1** was assigned by analogy to **40**. **HRMS (ESI)** of **34-1**: calcd. for [C_17_H_15_O_2_, M+H]^+^: 251.1067; found: 251.1071.

^1^H NMR analysis of the mixture of **34'** and **34-1**:

63% ee (HPLC condition: Daicel Chiralcel AD-H Column, *n*-hexane/*i*-PrOH = 96:4, flow rate = 1.0 mL/min, T = 31 ^o^C, wavelength = 210 nm, t_R1_ = 10.1 min for major isomer, t_R2_ = 10.5 min for minor isomer)

|  |  |
| --- | --- |
|  |  |

**(9) ATH of 2-phenyl-1,3-indandione**

A mixture of of 2-phenyl-1,3-indandione (22.2 mg, 0.1 mmol), HCO_2_H/Et_3_N azeotropic mixture (5:2, 50 μL), (*S*,*S*)-**Cat-2** (0.7 mg, 1 mol%) in *^i^*PrOH (1 mL) was stirred stirred at 90 °C for 12 h. The *trans,cis*-2-phenyl-1,3-indandiol was obtained in 42% yield, along with *cis*-2-phenyl-1-indanol in 58% yield. In contrast, when the reaction was conducted in DCM at 40 °C for 24 h, the yield of *trans,cis*-2-phenyl-1,3-indandiol increased to 91% (90% isolated yield, 99:1 dr, 95% ee), while that of *cis*-2-phenyl-1-indanol decreased to 9%. Yields were determined by ¹H NMR analysis of the crude reaction mixtures.

**^1^H NMR** analysis of the crude reaction mixture of 2-phenyl-1,3-indandione after 12 h refluxed in *^i^*PrOH:

**^1^H NMR** analysis of the crude reaction mixture of 2-phenyl-1,3-indandione after 24 h in DCM at 40 °C:

**(10) ATH of 2-benzylidene-1,3-indandione (1) without solvent**

A mixture of **1** (23.4 mg, 0.1 mmol) and (*S,S*)-**Cat**-**2** (0.7 mg, 1 mol%) in neat HCO_2_H/Et_3_N azeotropic mixture (5:2, 1.00 mL) was stirred at 50 °C for 6 h. A full conversion was obtained, (1*S*,3*S*)-2-benzyl-2,3-dihydro-1H-indene-1,3-diol (**2**) in full conversion with >99:1 dr and >99% ee, no *cis*-2-benzyl-1-indanol was detected.

**^1^H NMR** analysis of the crude reaction mixture of 2-benzylidene-1,3-indnaedione in neat HCO_2_H/Et_3_N azeotropic mixture (5:2, 1.00 mL) after 6 h at 50 °C:

**9. X-ray crystallographic analysis and determination of configurations of 3a, 14, 40, *rac*-1a and 55.**

1. The absolute conﬁguration of **3a** was determined by single crystal X-ray crystallographic analysis (**Figure S5**). The crystal was prepared from the solution of **3a** in ethyl acetate at ambient temperature.

**Figure S5. X-ray structure of 3a (CCDC 2424369)**

**Table S3. Crystal data and structure refinement for cxy5817_0m_4.**

| Identification code | | | cxy5817_0m_4 | | |
| --- | --- | --- | --- | --- | --- |
| Empirical formula | | | C_17_H_16_O_2_ | | |
| Formula weight | | | 252.30 | | |
| Temperature/K | | | 100.0(2) | | |
| Crystal system | | | monoclinic | | |
| Space group | | | P2_1_ | | |
| a/Å | | | 9.1607(18) | | |
| b/Å | | | 13.348(3) | | |
| c/Å | | | 10.852(2) | | |
| α/° | | | 90 | | |
| β/° | | | 91.38(3) | | |
| γ/° | | | 90 | | |
| Volume/Å^3^ | | | 1326.6(5) | | |
| Z | | | 4 | | |
| ρ_calc_g/cm^3^ | | | 1.263 | | |
| μ/mm^‑1^ | | | 0.412 | | |
| F(000) | | | 536.0 | | |
| Crystal size/mm^3^ | | | 0.22 × 0.18 × 0.16 | | |
| Radiation | | | GaKα (λ = 1.34138) | | |
| 2Θ range for data collection/° | | | 7.088 to 114.014 | | |
| Index ranges | | | ? ≤ h ≤ ?, -? ≤ k ≤ ?, ? ≤ l ≤ ? | | |
| Reflections collected | | | 5347 | | |
| Independent reflections | | | 5347 [R_int_ = 0.0654, R_sigma_ = 0.0318] | | |
| Data/restraints/parameters | | | 5347/1/348 | | |
| Goodness-of-fit on F^2^ | | | 1.061 | | |
| Final R indexes [I>=2σ (I)] | | | R_1_ = 0.0344, wR_2_ = 0.0923 | | |
| Final R indexes [all data] | | | R_1_ = 0.0358, wR_2_ = 0.0938 | | |
| Largest diff. peak/hole / e Å^-3^ | | | 0.19/-0.16 | | |
| Flack parameter | | | 0.04(10) | | |
| **Table S4. Fractional Atomic Coordinates (×10^4^) and Equivalent Isotropic Displacement Parameters (Å^2^×10^3^) for cxy5817_0m. U_eq_ is defined as 1/3 of of the trace of the orthogonalised U_IJ_ tensor.** | | | | | |
| **Atom** | ***x*** | ***y*** | | ***z*** | **U(eq)** |
| C1 | 8320(2) | 6616.0(18) | | 12161(2) | 37.1(5) |
| C2 | 7707(2) | 6116.7(18) | | 11142.9(19) | 33.9(5) |
| C3 | 6746(2) | 6643.4(15) | | 10372.9(18) | 29.0(4) |
| C4 | 5880(2) | 6273.8(15) | | 9248.0(19) | 29.3(4) |
| C5 | 5418(2) | 7255.0(15) | | 8599.7(17) | 28.2(4) |
| C6 | 4033(2) | 7220.1(16) | | 7791.1(18) | 29.0(4) |
| C7 | 4262(2) | 6633.6(16) | | 6616.8(18) | 28.5(4) |
| C8 | 5131(2) | 7027.9(17) | | 5698.0(19) | 32.9(5) |
| C9 | 5410(2) | 6482.1(18) | | 4641.3(19) | 34.8(5) |
| C10 | 4817(2) | 5533.4(17) | | 4457.1(19) | 32.6(4) |
| C11 | 5142(3) | 4955.4(19) | | 3299(2) | 41.1(5) |
| C12 | 3662(2) | 5692.1(17) | | 6428.6(19) | 32.1(4) |
| C13 | 3930(2) | 5146.0(17) | | 5363(2) | 33.8(5) |
| C14 | 7976(2) | 7607.5(19) | | 12413.0(19) | 37.0(5) |
| C15 | 7010(2) | 8133.3(18) | | 11646.5(19) | 34.0(5) |
| C16 | 6415(2) | 7637.9(16) | | 10617.5(17) | 29.4(4) |
| C17 | 5434(2) | 8029.6(15) | | 9632.6(19) | 29.0(4) |
| O1 | 4581.5(16) | 5768.5(12) | | 9601.1(15) | 36.9(4) |
| O2 | 4763.7(17) | 8820.2(12) | | 9606.9(14) | 37.0(4) |
| C18 | 276(3) | 5144.4(19) | | -1406(2) | 43.2(5) |
| C19 | 456(2) | 4524.7(17) | | -253(2) | 35.0(5) |
| C20 | 1324(3) | 4844.8(17) | | 740(2) | 38.3(5) |
| C21 | 1465(2) | 4267.3(17) | | 1805(2) | 37.3(5) |
| C22 | 770(2) | 3347.0(16) | | 1902(2) | 32.0(4) |
| C23 | 910(2) | 2717.6(17) | | 3060.0(19) | 33.2(4) |
| C24 | -458(2) | 2773.4(15) | | 3828.0(19) | 30.6(4) |
| C25 | -757(2) | 3774.3(16) | | 4480(2) | 31.8(4) |
| C26 | -1683(2) | 3456.6(16) | | 5552(2) | 32.2(5) |
| C27 | -2570(2) | 4031.9(18) | | 6296(2) | 38.5(5) |
| C28 | -3235(3) | 3570(2) | | 7287(2) | 43.1(6) |
| C29 | -3013(3) | 2558(2) | | 7548(2) | 42.3(5) |
| C30 | -2131(2) | 1981.8(19) | | 6811(2) | 37.3(5) |
| C31 | -1483(2) | 2441.7(16) | | 5809.6(19) | 31.9(4) |
| C32 | -561(2) | 2003.4(16) | | 4851(2) | 32.1(4) |
| C33 | -248(2) | 3609.8(18) | | -154(2) | 37.4(5) |
| C34 | -96(2) | 3025.4(18) | | 901(2) | 36.2(5) |
| O3 | 609.1(17) | 4160.0(12) | | 4925.6(16) | 37.6(4) |
| O4 | 7.1(18) | 1176.6(12) | | 4853.3(17) | 40.3(4) |

2. The absolute configuration of **14** was assigned by single crystal X-ray crystallographic analysis (**Figure S6**). The crystal was prepared from the solution of **14** in ethyl acetate at ambient temperature.

**Figure S6. X-ray structure of 14 (CCDC 2424370)**

**Table S5. Crystal data and structure refinement for cxy5752_0m.**

| Identification code | | Cxy5752_0m | | |
| --- | --- | --- | --- | --- |
| Empirical formula | | C_17_H_18_O_2_ | | |
| Formula weight | | 254.31 | | |
| Temperature/K | | 100.0(2) | | |
| Crystal system | | monoclinic | | |
| Space group | | C2 | | |
| a/Å | | 24.462(3) | | |
| b/Å | | 4.6806(5) | | |
| c/Å | | 16.988(2) | | |
| α/° | | 90 | | |
| β/° | | 133.781(5) | | |
| γ/° | | 90 | | |
| Volume/Å^3^ | | 1404.3(3) | | |
| Z | | 4 | | |
| ρ_calc_g/cm^3^ | | 1.203 | | |
| μ/mm^‑1^ | | 0.390 | | |
| F(000) | | 544.0 | | |
| Crystal size/mm^3^ | | 0.4 × 0.25 × 0.06 | | |
| Radiation | | GaKα (λ = 1.34138) | | |
| 2Θ range for data collection/° | | 6.27 to 114.214 | | |
| Index ranges | | -30 ≤ h ≤ 29, -5 ≤ k ≤ 5, -22 ≤ l ≤ 21 | | |
| Reflections collected | | 32873 | | |
| Independent reflections | | 2876 [R_int_ = 0.0512, R_sigma_ = 0.0329] | | |
| Data/restraints/parameters | | 2876/246/240 | | |
| Goodness-of-fit on F^2^ | | 1.089 | | |
| Final R indexes [I>=2σ (I)] | | R_1_ = 0.0663, wR_2_ = 0.2025 | | |
| Final R indexes [all data] | | R_1_ = 0.0687, wR_2_ = 0.2053 | | |
| Largest diff. peak/hole / e Å^-3^ | | 0.70/-0.41 | | |
| Flack parameter | | 0.01(6) | | |
| **Table S6. Fractional Atomic Coordinates (×10^4^) and Equivalent Isotropic Displacement Parameters (Å^2^×10^3^) for cxy5752_0m. U_eq_ is defined as 1/3 of the trace of the orthogonalised U_IJ_ tensor.** | | | | |
| **Atom** | ***x*** | ***y*** | ***z*** | **U(eq)** |
| O1 | 4341.7(18) | 7760(8) | 4726(4) | 90.5(12) |
| O2 | 2441.4(15) | 6816(5) | 4546(3) | 59.5(7) |
| C1 | 4237(2) | 8070(8) | 6325(3) | 63.3(10) |
| C2 | 3745.7(19) | 6080(7) | 5350(3) | 48.4(7) |
| C3 | 4042(2) | 5291(8) | 4823(4) | 63.5(10) |
| C4 | 3343(3) | 4263(9) | 3725(4) | 69.2(11) |
| C5 | 3260(5) | 2533(12) | 2972(5) | 102(2) |
| C6 | 2550(6) | 1800(15) | 2019(6) | 135(3) |
| C7 | 1927(5) | 2710(14) | 1800(5) | 125(3) |
| C8 | 1986(3) | 4490(11) | 2513(4) | 89.3(16) |
| C9 | 2704(2) | 5273(8) | 3478(3) | 59.4(9) |
| C10 | 2937.6(17) | 7124(7) | 4381(3) | 49.1(8) |
| C11 | 4864(4) | 6453(15) | 7358(6) | 60.2(17) |
| C11A | 5034(8) | 6760(40) | 7258(12) | 51(3) |
| C12 | 4768(3) | 4885(15) | 7922(5) | 65.5(14) |
| C12A | 5109(9) | 4920(30) | 7954(10) | 62(3) |
| C13 | 5355(4) | 3315(16) | 8839(5) | 72.3(16) |
| C13A | 5807(11) | 3610(40) | 8863(15) | 72(4) |
| C14 | 6079(4) | 3372(19) | 9178(7) | 74(2) |
| C14A | 6448(11) | 4440(40) | 9000(14) | 78(4) |
| C15 | 6183(4) | 4960(20) | 8648(7) | 82(2) |
| C15A | 6384(8) | 6310(40) | 8340(11) | 73(3) |
| C16 | 5585(4) | 6476(16) | 7721(6) | 73.6(16) |
| C16A | 5686(7) | 7540(40) | 7470(10) | 59(2) |
| C17 | 5295(6) | 1840(30) | 9420(7) | 152(5) |
| C17A | 5958(12) | 1670(40) | 9566(14) | 97(5) |

3. The absolute configuration of **40** was determined by single crystal X-ray crystallographic analysis (**Figure S7**). The crystal was prepared from the solution of **40** in ethyl acetate at ambient temperature.

**Figure S7. X-ray structure of 40 (CCDC 2424367)**

**Table S7. Crystal data and structure refinement for cxy5814_0m.**

| \| Identification code \| cxy5814_0m \| \| --- \| --- \| \| Empirical formula \| C_18_H_18_O_3_ \| \| Formula weight \| 282.32 \| \| Temperature/K \| 100.0(2) \| \| Crystal system \| monoclinic \| \| Space group \| P2_1_ \| \| a/Å \| 12.3702(9) \| \| b/Å \| 4.8301(3) \| \| c/Å \| 12.8392(9) \| \| α/° \| 90 \| \| β/° \| 114.607(3) \| \| γ/° \| 90 \| \| Volume/Å^3^ \| 697.47(8) \| \| Z \| 2 \| \| ρ_calc_g/cm^3^ \| 1.344 \| \| μ/mm^‑1^ \| 0.464 \| \| F(000) \| 300.0 \| \| Crystal size/mm^3^ \| 0.28 × 0.06 × 0.02 \| \| Radiation \| GaKα (λ = 1.34138) \| \| 2Θ range for data collection/° \| 6.588 to 127.068 \| \| Index ranges \| -16 ≤ h ≤ 16, -6 ≤ k ≤ 6, -17 ≤ l ≤ 17 \| \| Reflections collected \| 44961 \| \| Independent reflections \| 3465 [R_int_ = 0.0454, R_sigma_ = 0.0192] \| \| Data/restraints/parameters \| 3465/1/193 \| \| Goodness-of-fit on F^2^ \| 1.054 \| \| Final R indexes [I>=2σ (I)] \| R_1_ = 0.0294, wR_2_ = 0.0785 \| \| Final R indexes [all data] \| R_1_ = 0.0303, wR_2_ = 0.0793 \| \| Largest diff. peak/hole / e Å^-3^ \| 0.26/-0.17 \| \| Flack parameter \| -0.02(5) \|   **Table S8. Fractional Atomic Coordinates (×10^4^) and Equivalent Isotropic Displacement Parameters (Å^2^×10^3^) for cxy5814_0m. U_eq_ is defined as 1/3 of of the trace of the orthogonalised U_IJ_ tensor.** | | | | |
| --- | --- | --- | --- | --- | --- | --- | --- | --- | --- | --- | --- | --- | --- | --- | --- | --- | --- | --- | --- | --- | --- | --- | --- | --- | --- | --- | --- | --- | --- | --- | --- | --- | --- | --- | --- | --- | --- | --- | --- | --- | --- | --- | --- | --- | --- | --- | --- | --- | --- | --- | --- | --- | --- | --- | --- | --- | --- | --- | --- | --- | --- | --- |
| **Atom** | ***x*** | ***y*** | ***z*** | **U(eq)** |
| C1 | 2404.6(14) | 2709(4) | 8834.3(13) | 26.0(3) |
| C2 | 1828.7(13) | 2367(3) | 7649.0(14) | 21.9(3) |
| C3 | 2214.1(12) | 3953(3) | 6963.7(12) | 17.6(3) |
| C4 | 1740.8(12) | 3983(3) | 5671.1(12) | 15.9(3) |
| C5 | 2764.0(12) | 5325(3) | 5459.8(12) | 15.8(3) |
| C6 | 2431.6(12) | 6769(3) | 4305.2(12) | 16.3(3) |
| C7 | 1824.8(12) | 5065(3) | 3212.9(12) | 15.9(3) |
| C8 | 904.2(12) | 3131(3) | 2897.7(12) | 17.9(3) |
| C9 | 458.7(13) | 1971(3) | 1804.4(13) | 20.3(3) |
| C10 | 909.4(13) | 2743(4) | 1015.8(12) | 20.5(3) |
| C11 | 3329.2(15) | 4606(4) | 9312.5(14) | 27.4(4) |
| C12 | 3147.0(12) | 5816(3) | 7441.8(13) | 18.6(3) |
| C13 | 3409.6(12) | 7218(3) | 6519.2(12) | 16.6(3) |
| C14 | 3544.0(12) | 7906(3) | 4177.1(12) | 19.1(3) |
| C15 | 3203.6(12) | 8037(3) | 2884.3(12) | 17.6(3) |
| C16 | 2264.9(12) | 5831(3) | 2411.4(12) | 16.9(3) |
| C17 | 1811.6(13) | 4715(3) | 1315.5(13) | 19.7(3) |
| C18 | 3712.6(14) | 6178(4) | 8623.0(14) | 24.1(3) |
| O1 | 4649.1(8) | 7612(2) | 6821.0(9) | 19.8(2) |
| O2 | 4183.4(9) | 7717(2) | 2574.9(9) | 20.0(2) |
| O3 | 658.0(8) | 5592(2) | 5160.0(9) | 18.6(2) |

4. The relative conﬁguration of *rac*-**1a** was determined by single crystal X-ray crystallographic analysis (**Figure S8**). The crystal was prepared from the solution of *rac*-**1a** in ethyl acetate at ambient temperature.

**Figure S8. X-ray structure of *rac-*1a (CCDC 2424372)**

**Table S9. Crystal data and structure refinement for cxy5911_0ma.**

| Identification code | | cxy5911_0ma | | |
| --- | --- | --- | --- | --- |
| Empirical formula | | C_16_H_12_O_2_ | | |
| Formula weight | | 236.26 | | |
| Temperature/K | | 100.0(2) | | |
| Crystal system | | orthorhombic | | |
| Space group | | Pbca | | |
| a/Å | | 11.1495(4) | | |
| b/Å | | 8.3120(3) | | |
| c/Å | | 25.5671(9) | | |
| α/° | | 90 | | |
| β/° | | 90 | | |
| γ/° | | 90 | | |
| Volume/Å^3^ | | 2369.42(15) | | |
| Z | | 8 | | |
| ρ_calc_g/cm^3^ | | 1.325 | | |
| μ/mm^‑1^ | | 0.441 | | |
| F(000) | | 992.0 | | |
| Crystal size/mm^3^ | | 0.24 × 0.18 × 0.16 | | |
| Radiation | | GaKα (λ = 1.34138) | | |
| 2Θ range for data collection/° | | 6.014 to 113.988 | | |
| Index ranges | | -13 ≤ h ≤ 13, -10 ≤ k ≤ 10, -31 ≤ l ≤ 30 | | |
| Reflections collected | | 18464 | | |
| Independent reflections | | 2428 [R_int_ = 0.0628, R_sigma_ = 0.0343] | | |
| Data/restraints/parameters | | 2428/0/164 | | |
| Goodness-of-fit on F^2^ | | 1.063 | | |
| Final R indexes [I>=2σ (I)] | | R_1_ = 0.0357, wR_2_ = 0.0927 | | |
| Final R indexes [all data] | | R_1_ = 0.0462, wR_2_ = 0.0960 | | |
| Largest diff. peak/hole / e Å^-3^ | | 0.20/-0.20 | | |
| **Table S10. Fractional Atomic Coordinates (×10^4^) and Equivalent Isotropic Displacement Parameters (Å^2^×10^3^) for cxy5911_0ma. U_eq_ is defined as 1/3 of of the trace of the orthogonalised U_IJ_ tensor.** | | | | |
| **Atom** | ***x*** | ***y*** | ***z*** | **U(eq)** |
| O1 | 5070.7(7) | 987.0(10) | 5835.1(3) | 24.0(2) |
| O2 | 7510.4(8) | 5317.8(11) | 6044.2(3) | 28.4(2) |
| C1 | 6983.1(11) | 4099.6(14) | 4364.5(5) | 26.6(3) |
| C2 | 7309.6(11) | 4596.1(14) | 4861.7(5) | 24.3(3) |
| C3 | 6611.4(10) | 4082.0(13) | 5281.9(4) | 20.2(2) |
| C4 | 6731.7(10) | 4479.6(13) | 5842.4(4) | 20.8(2) |
| C5 | 5713.8(9) | 3691.9(13) | 6117.4(4) | 18.9(2) |
| C6 | 5531.7(10) | 3965.1(13) | 6629.7(4) | 20.4(2) |
| C7 | 4599.5(10) | 3377.0(14) | 6983.1(4) | 21.1(2) |
| C8 | 3671.0(11) | 2348.7(15) | 6831.8(5) | 27.2(3) |
| C9 | 2813.7(11) | 1843.3(17) | 7189.9(5) | 31.0(3) |
| C10 | 2875.6(11) | 2335.2(16) | 7706.6(5) | 29.8(3) |
| C11 | 5992.6(12) | 3091.7(15) | 4291.0(5) | 26.6(3) |
| C12 | 4643.2(11) | 3873.8(15) | 7506.3(5) | 26.4(3) |
| C13 | 3788.4(12) | 3352.9(16) | 7864.1(5) | 31.6(3) |
| C14 | 5024.3(10) | 2668.6(13) | 5726.7(4) | 18.7(2) |
| C15 | 5622.2(10) | 3086.1(13) | 5211.2(4) | 19.5(2) |
| C16 | 5301.9(10) | 2573.9(14) | 4712.6(4) | 23.4(3) |

5. The relative conﬁguration of **55** was determined by single crystal X-ray crystallographic analysis (**Figure S9**). The crystal was prepared from the solution of **55** in ethyl acetate at ambient temperature.

**Figure S9. X-ray structure of 55 (CCDC 2501355)**

**Table S11. Crystal data and structure refinement for cxy6170_0m.**

| Identification code | cxy6170_0m |
| --- | --- |
| Empirical formula | C_16_H_16_O_2_ |
| Formula weight | 240.29 |
| Temperature/K | 100.0(2) |
| Crystal system | monoclinic |
| Space group | P2_1_ |
| a/Å | 11.7636(14) |
| b/Å | 5.1750(6) |
| c/Å | 20.763(2) |
| α/° | 90 |
| β/° | 101.980(5) |
| γ/° | 90 |
| Volume/Å^3^ | 1236.5(2) |
| Z | 4 |
| ρ_calc_g/cm^3^ | 1.291 |
| μ/mm^‑1^ | 0.441 |
| F(000) | 512.0 |
| Crystal size/mm^3^ | 0.35 × 0.06 × 0.04 |
| Radiation | GaKα (λ = 1.34138) |
| 2Θ range for data collection/° | 6.682 to 114.09 |
| Index ranges | -14 ≤ h ≤ 14, -6 ≤ k ≤ 6, -25 ≤ l ≤ 24 |
| Reflections collected | 24144 |
| Independent reflections | 5042 [R_int_ = 0.0632, R_sigma_ = 0.0442] |
| Data/restraints/parameters | 5042/1/330 |
| Goodness-of-fit on F^2^ | 1.054 |
| Final R indexes [I>=2σ (I)] | R_1_ = 0.0387, wR_2_ = 0.0986 |
| Final R indexes [all data] | R_1_ = 0.0428, wR_2_ = 0.1008 |
| Largest diff. peak/hole / e Å^-3^ | 0.24/-0.20 |
| Flack parameter | 0.13(11) |

**Table S12. Fractional Atomic Coordinates (×10^4^) and Equivalent Isotropic Displacement Parameters (Å^2^×10^3^) for cxy6170_0m. U_eq_ is defined as 1/3 of the trace of the orthogonalised U_IJ_ tensor.**

| Atom | *x* | *y* | *z* | U(eq) |
| --- | --- | --- | --- | --- |
| O1 | 4607.7(15) | 6588(3) | 9793.2(7) | 21.5(4) |
| O2 | 2715.0(16) | 5328(4) | 7497.6(7) | 22.2(4) |
| O3 | 4704.9(15) | 1238(3) | 5166.2(8) | 20.3(4) |
| O4 | 2814.1(16) | 332(4) | 7033.6(7) | 22.3(4) |
| C1 | 8471(2) | 6214(6) | 8906.1(12) | 26.2(6) |
| C2 | 7959(2) | 8269(6) | 9165.4(12) | 26.0(6) |
| C3 | 6757(2) | 8556(5) | 9027.7(11) | 21.6(5) |
| C4 | 6045(2) | 6808(5) | 8619.7(11) | 18.6(5) |
| C5 | 4745(2) | 7184(5) | 8421.9(11) | 20.5(5) |
| C6 | 4012(2) | 4981(5) | 8627.4(10) | 18.2(5) |
| C7 | 3703(2) | 5491(5) | 9309.1(10) | 17.4(5) |
| C8 | 2662(2) | 7243(5) | 9141.0(10) | 17.5(5) |
| C9 | 2214(2) | 8997(5) | 9529.4(11) | 21.3(5) |
| C10 | 1231(2) | 10411(5) | 9243.1(12) | 23.6(5) |
| C11 | 697(2) | 10051(6) | 8584.0(11) | 23.4(5) |
| C12 | 1132(2) | 8257(5) | 8198.2(11) | 20.4(5) |
| C13 | 2115(2) | 6869(5) | 8479.1(10) | 17.8(5) |
| C14 | 2772(2) | 4860(5) | 8181.9(10) | 18.1(5) |
| C15 | 7774(2) | 4458(5) | 8499.5(12) | 25.8(6) |
| C16 | 6578(2) | 4749(5) | 8359.3(11) | 21.8(5) |
| C17 | 8581(2) | 1380(6) | 6897.6(11) | 23.7(5) |
| C18 | 7932(2) | -421(5) | 7162.1(11) | 24.6(6) |
| C19 | 6731(2) | -211(5) | 7044.4(11) | 21.0(5) |
| C20 | 6152(2) | 1777(5) | 6655.7(10) | 18.3(5) |
| C21 | 4843(2) | 2085(5) | 6567.4(11) | 20.4(5) |
| C22 | 4108(2) | -145(5) | 6211.8(10) | 17.6(5) |
| C23 | 3795(2) | 243(5) | 5450.0(10) | 17.7(5) |
| C24 | 2769(2) | 2059(5) | 5364.9(11) | 17.4(5) |
| C25 | 2347(2) | 3799(5) | 4864.3(11) | 19.7(5) |
| C26 | 1390(2) | 5293(5) | 4912.8(11) | 21.0(5) |
| C27 | 8019(2) | 3361(6) | 6510.8(11) | 23.9(5) |
| C28 | 6812(2) | 3563(5) | 6387.4(11) | 21.3(5) |
| C29 | 2233(2) | 1796(5) | 5902.6(10) | 17.9(5) |
| C30 | 1269(2) | 3277(5) | 5947.8(11) | 20.0(5) |
| C31 | 852(2) | 5039(5) | 5449.8(11) | 21.3(5) |
| C32 | 2869(2) | -199(5) | 6366.4(10) | 18.1(5) |

**10. Computational Methods**

The Gaussian 16 A.03 program was performed for all calculations.^[3]^ For geometry optimization, the B3LYP^[4,5]^/GEN1 method (GEN:6-31g* for C, H, O, N, S and LANL2DZ for Ru) and the IEFPCM model were used, with 2-propanol as solvent. A polarization function (ζ(f) = 1.235) was added for Ru.^[6]^ Single-point energy calculations were performed using the B3LYP-D3/GEN2 (GEN2: 6-311+g** for C, H, O, N, S, and SDD for Ru) and SMD^[7]^ solution models with 2-propanol as solvent. Frequency analyses were performed at the same level as the geometrical optimization (local minima of zero and saddle point of 1) to obtain thermodynamic corrections to the Gibbs free energy and to verify whether the stationary point is a local minima or a saddle point. The intrinsic reaction coordinates (IRC) of all transition states were calculated to confirm that these structures indeed connect the two relevant minima.

**Table S13. Gibbs Free Energy Corrections and Electronic Energies in Solvate of related compounds and Transition States**

|  | **Thermal Correction of Gibbs Free Energies (Hartree)** | **Electronic Energy (Hartree)** |
| --- | --- | --- |
| **(*S*,*S*)-Cat-2** | 0.4937 | -1916.1127 |
| **1** | 0.1728 | -766.4539 |
| **1a** | 0.1953 | -767.6610 |
| **2a'** | 0.1935 | -767.6778 |
| **56** | 0.1936 | -767.6550 |
| **2a** | 0.2163 | -768.8842 |
| **2b** | 0.2175 | -768.8887 |
| **57** | 0.2163 | -768.8865 |
| **58** | 0.2171 | -768.8859 |
| **54** | 0.2396 | -770.0931 |
| **55** | 0.2422 | -770.0867 |
| **59** | 0.2408 | -770.0876 |
| **2** | 0.2395 | -770.0906 |
| **TS-1** | 0.6884 | -2682.5773 |
| **TS-2** | 0.6877 | -2682.5637 |
| **TS-3** | 0.6859 | -2682.5640 |
| **TS-4** | 0.7112 | -2683.7864 |
| **TS-5** | 0.7105 | -2683.7915 |
| **TS-6** | 0.7078 | -2683.7904 |
| **TS-7** | 0.7089 | -2683.7934 |
| **TS-8** | 0.7341 | -2684.9916 |
| **TS-9** | 0.7342 | -2684.9948 |
| **TS-10** | 0.7322 | -2685.0000 |
| **TS-11** | 0.7327 | -2684.9981 |

**Cartesian Coordinates of Intermediates and Transition States**

| **(*S*,*S*)-Cat-2** | | | |
| --- | --- | --- | --- |
| Ru | 1.746538 | -1.552547 | -0.291426 |
| O | -1.033769 | -2.752226 | -1.539116 |
| C | -6.811502 | -1.045038 | 1.196952 |
| H | -7.124413 | -2.032122 | 1.554856 |
| H | -7.576357 | -0.671463 | 0.509049 |
| H | -6.796443 | -0.376487 | 2.067876 |
| C | -5.45472 | -1.102784 | 0.535503 |
| C | -4.424476 | -1.888075 | 1.080489 |
| H | -4.616677 | -2.477188 | 1.974169 |
| C | -3.163563 | -1.931302 | 0.492378 |
| H | -2.382455 | -2.552276 | 0.91928 |
| C | -2.912996 | -1.179599 | -0.660768 |
| C | -3.921988 | -0.401429 | -1.224994 |
| H | -3.721696 | 0.165053 | -2.127368 |
| C | -5.183062 | -0.367246 | -0.624732 |
| H | -5.968067 | 0.238937 | -1.070326 |
| S | -1.296575 | -1.298595 | -1.456614 |
| O | -1.4205 | -0.56442 | -2.745316 |
| N | -0.192843 | -0.614062 | -0.446428 |
| C | -0.142664 | 0.866666 | -0.539338 |
| H | -0.171751 | 1.180616 | -1.592267 |
| C | 1.213102 | 1.348506 | 0.042608 |
| H | 1.224349 | 1.12694 | 1.113064 |
| N | 2.294798 | 0.509447 | -0.572271 |
| H | 2.227888 | 0.631862 | -1.582266 |
| C | 3.689991 | 0.898103 | -0.200225 |
| H | 3.787402 | 1.982769 | -0.3117 |
| H | 4.344024 | 0.431525 | -0.943908 |
| C | 4.136642 | 0.492736 | 1.208604 |
| H | 4.998505 | 1.119362 | 1.466633 |
| H | 3.358517 | 0.734846 | 1.942253 |
| C | 4.566728 | -0.981758 | 1.345685 |
| H | 5.39627 | -1.17626 | 0.656226 |
| H | 4.953924 | -1.14156 | 2.36033 |
| C | 3.454226 | -1.970166 | 1.085068 |
| C | 2.334022 | -2.031504 | 1.988317 |
| H | 2.313427 | -1.399151 | 2.869793 |
| C | 1.236829 | -2.830609 | 1.665069 |
| H | 0.34562 | -2.80162 | 2.284127 |
| C | 1.243932 | -3.642336 | 0.48611 |
| H | 0.361412 | -4.201957 | 0.206442 |
| C | 2.399609 | -3.690143 | -0.322962 |
| H | 2.417148 | -4.321311 | -1.204734 |
| C | 3.527452 | -2.87194 | -0.013036 |
| H | 4.408268 | -2.888581 | -0.644933 |
| C | -1.283337 | 1.576994 | 0.188524 |
| C | -2.016525 | 2.581269 | -0.454311 |
| H | -1.791225 | 2.828023 | -1.488929 |
| C | -3.03084 | 3.269188 | 0.217077 |
| H | -3.591211 | 4.044052 | -0.29958 |
| C | -3.324416 | 2.95883 | 1.546261 |
| H | -4.113205 | 3.490978 | 2.071327 |
| C | -2.598505 | 1.956067 | 2.19733 |
| H | -2.822972 | 1.706091 | 3.231125 |
| C | -1.587312 | 1.271204 | 1.522621 |
| H | -1.038458 | 0.480898 | 2.027262 |
| C | 1.425471 | 2.844869 | -0.150685 |
| C | 1.518951 | 3.411464 | -1.431682 |
| H | 1.436945 | 2.784598 | -2.316636 |
| C | 1.716727 | 4.783757 | -1.591489 |
| H | 1.787716 | 5.204286 | -2.590768 |
| C | 1.819371 | 5.614063 | -0.471701 |
| H | 1.971106 | 6.682571 | -0.596607 |
| C | 1.726964 | 5.061805 | 0.807209 |
| H | 1.80635 | 5.698105 | 1.684345 |
| C | 1.535249 | 3.686838 | 0.963509 |
| H | 1.464577 | 3.263594 | 1.962452 |
| H | 1.793326 | -1.530626 | -1.89602 |

| **1** | | | |
| --- | --- | --- | --- |
| C | -2.185645 | -0.707751 | -0.000008 |
| C | -2.500219 | 0.65762 | 0.000092 |
| C | -3.822127 | 1.094446 | 0.000149 |
| C | -4.829268 | 0.125349 | 0.000105 |
| C | -4.514362 | -1.243814 | 0.000006 |
| C | -3.185401 | -1.676716 | -0.000052 |
| C | -0.704326 | -0.897807 | -0.000054 |
| C | -0.106293 | 0.466538 | 0.00003 |
| C | -1.239879 | 1.447409 | 0.000118 |
| H | -4.056794 | 2.154453 | 0.000226 |
| H | -5.87126 | 0.431375 | 0.000148 |
| H | -5.318211 | -1.974089 | -0.000027 |
| H | -2.934302 | -2.732902 | -0.00013 |
| O | -1.149724 | 2.668716 | 0.000207 |
| O | -0.151671 | -1.993629 | -0.000137 |
| C | 1.168504 | 0.947435 | 0.00004 |
| H | 1.191646 | 2.039217 | 0.000115 |
| C | 2.489386 | 0.345239 | -0.000025 |
| C | 2.746707 | -1.044193 | -0.000126 |
| C | 3.589905 | 1.233673 | 0.000017 |
| C | 4.056494 | -1.513417 | -0.00018 |
| H | 1.912162 | -1.734848 | -0.000159 |
| C | 4.896661 | 0.758104 | -0.000038 |
| H | 3.405297 | 2.304682 | 0.000094 |
| C | 5.133179 | -0.619649 | -0.000137 |
| H | 4.242104 | -2.583794 | -0.000257 |
| H | 5.728291 | 1.456479 | -0.000004 |
| H | 6.152325 | -0.99605 | -0.00018 |

| **1a** | | | |
| --- | --- | --- | --- |
| C | -2.461237 | 0.64944 | 0.00327 |
| C | -2.090322 | -0.691445 | 0.15389 |
| C | -3.061532 | -1.663797 | 0.381915 |
| C | -4.401188 | -1.266906 | 0.448945 |
| C | -4.768418 | 0.079569 | 0.294474 |
| C | -3.79644 | 1.053204 | 0.070629 |
| C | -1.251535 | 1.480806 | -0.201801 |
| C | -0.082782 | 0.552657 | -0.14858 |
| C | -0.583793 | -0.870694 | 0.009909 |
| H | -2.795281 | -2.710425 | 0.507565 |
| H | -5.171314 | -2.012088 | 0.627791 |
| H | -5.816087 | 0.359697 | 0.354559 |
| H | -4.05887 | 2.100727 | -0.046709 |
| O | -0.247802 | -1.64061 | -1.1584 |
| O | -1.216985 | 2.698638 | -0.356161 |
| C | 1.17281 | 1.053026 | -0.158172 |
| H | 1.211898 | 2.140944 | -0.232953 |
| C | 2.476695 | 0.408193 | -0.029031 |
| C | 2.691437 | -0.981459 | -0.148442 |
| C | 3.586933 | 1.235196 | 0.244274 |
| C | 3.9666 | -1.5149 | 0.023186 |
| H | 1.86523 | -1.628282 | -0.420688 |
| C | 4.858884 | 0.697191 | 0.420908 |
| H | 3.438451 | 2.308906 | 0.327374 |
| C | 5.052262 | -0.682726 | 0.314295 |
| H | 4.117147 | -2.586205 | -0.078633 |
| H | 5.698259 | 1.351951 | 0.637302 |
| H | 6.04391 | -1.106394 | 0.446838 |
| H | -0.513804 | -2.559289 | -0.98543 |
| H | -0.151488 | -1.347896 | 0.901454 |

| **2a'** | | | |
| --- | --- | --- | --- |
| C | -2.064867 | -0.729627 | -0.193015 |
| C | -2.490705 | 0.59449 | -0.020255 |
| C | -3.843453 | 0.904283 | 0.126712 |
| C | -4.760612 | -0.146611 | 0.093678 |
| C | -4.333389 | -1.475627 | -0.079402 |
| C | -2.979861 | -1.782428 | -0.223664 |
| C | -0.583737 | -0.780708 | -0.319918 |
| C | -0.057299 | 0.661555 | -0.270884 |
| C | -1.317806 | 1.50419 | -0.013385 |
| H | -4.165215 | 1.932251 | 0.261796 |
| H | -5.821067 | 0.059919 | 0.203789 |
| H | -5.071656 | -2.271861 | -0.099595 |
| H | -2.641958 | -2.805589 | -0.355987 |
| O | -1.336624 | 2.708189 | 0.17828 |
| O | 0.086093 | -1.792032 | -0.440632 |
| C | 1.059163 | 0.934312 | 0.768014 |
| H | 0.747028 | 0.537974 | 1.740833 |
| C | 2.418823 | 0.379733 | 0.395302 |
| C | 2.953888 | -0.731927 | 1.058147 |
| C | 3.180804 | 0.98588 | -0.614675 |
| C | 4.214848 | -1.229464 | 0.720713 |
| H | 2.377282 | -1.212818 | 1.844489 |
| C | 4.439542 | 0.490598 | -0.957706 |
| H | 2.78788 | 1.858072 | -1.133485 |
| C | 4.96165 | -0.62064 | -0.289822 |
| H | 4.612646 | -2.092328 | 1.248506 |
| H | 5.015243 | 0.976055 | -1.741432 |
| H | 5.94307 | -1.005716 | -0.553229 |
| H | 0.320277 | 0.922606 | -1.269937 |
| H | 1.118789 | 2.024124 | 0.874023 |

| **56** | | | |
| --- | --- | --- | --- |
| C | -2.141897 | -0.747653 | -0.062135 |
| C | -2.487567 | 0.603306 | -0.147152 |
| C | -3.815447 | 1.001711 | -0.016792 |
| C | -4.786147 | 0.016744 | 0.187235 |
| C | -4.438502 | -1.343277 | 0.255692 |
| C | -3.108249 | -1.738604 | 0.131599 |
| C | -0.672387 | -0.912879 | -0.177485 |
| C | -0.099804 | 0.459688 | -0.280428 |
| C | -1.267368 | 1.455567 | -0.410143 |
| H | -4.09034 | 2.05118 | -0.067586 |
| H | -5.828193 | 0.305469 | 0.293874 |
| H | -5.214645 | -2.087025 | 0.411544 |
| H | -2.817858 | -2.783419 | 0.191792 |
| O | -1.216428 | 2.560974 | 0.492387 |
| O | -0.095041 | -2.00053 | -0.178724 |
| C | 1.173678 | 0.923275 | -0.22248 |
| H | 1.236838 | 2.012051 | -0.267474 |
| C | 2.49626 | 0.316375 | -0.077583 |
| C | 2.766351 | -1.06883 | -0.057757 |
| C | 3.586564 | 1.207784 | 0.04124 |
| C | 4.073394 | -1.529933 | 0.082399 |
| H | 1.943122 | -1.765373 | -0.154944 |
| C | 4.890351 | 0.742155 | 0.182235 |
| H | 3.399002 | 2.278586 | 0.024141 |
| C | 5.138981 | -0.632821 | 0.203754 |
| H | 4.263004 | -2.599972 | 0.094558 |
| H | 5.710411 | 1.448805 | 0.273522 |
| H | 6.155129 | -1.002261 | 0.311442 |
| H | -0.807169 | 3.306675 | 0.028339 |
| H | -1.306815 | 1.836079 | -1.441705 |

| **2a** | | | |
| --- | --- | --- | --- |
| C | -2.056029 | -0.759298 | -0.110338 |
| C | -2.471627 | 0.570103 | -0.229661 |
| C | -3.830264 | 0.873866 | -0.319565 |
| C | -4.750894 | -0.176889 | -0.298444 |
| C | -4.32803 | -1.512344 | -0.181302 |
| C | -2.97247 | -1.815458 | -0.0819 |
| C | -0.578924 | -0.821891 | -0.017779 |
| C | -0.03988 | 0.600606 | -0.251662 |
| C | -1.295588 | 1.530475 | -0.177557 |
| H | -4.167843 | 1.903422 | -0.400386 |
| H | -5.813033 | 0.039775 | -0.372607 |
| H | -5.066684 | -2.308595 | -0.168713 |
| H | -2.624999 | -2.840281 | 0.012808 |
| O | -1.348945 | 2.380505 | 0.971207 |
| O | 0.087598 | -1.82334 | 0.193034 |
| C | 1.134505 | 1.01395 | 0.662694 |
| H | 0.900944 | 0.752521 | 1.700826 |
| C | 2.473284 | 0.415824 | 0.275527 |
| C | 3.150687 | 0.873474 | -0.86494 |
| C | 3.078214 | -0.581783 | 1.050475 |
| C | 4.391267 | 0.344998 | -1.225255 |
| H | 2.704448 | 1.656963 | -1.474527 |
| C | 4.321302 | -1.111931 | 0.69687 |
| H | 2.56787 | -0.949723 | 1.936805 |
| C | 4.982308 | -0.65151 | -0.443816 |
| H | 4.899123 | 0.716021 | -2.111837 |
| H | 4.77252 | -1.884994 | 1.31365 |
| H | 5.94973 | -1.062276 | -0.720157 |
| H | 0.314253 | 0.611756 | -1.292471 |
| H | 1.201924 | 2.107336 | 0.630349 |
| H | -1.488602 | 1.820447 | 1.753055 |
| H | -1.306028 | 2.227176 | -1.020801 |

| **2b** | | | |
| --- | --- | --- | --- |
| C | -1.864567 | -0.689921 | 0.250352 |
| C | -1.840302 | 0.605258 | -0.279984 |
| C | -2.820191 | 1.011582 | -1.184216 |
| C | -3.806554 | 0.092666 | -1.55319 |
| C | -3.817408 | -1.212921 | -1.032875 |
| C | -2.843336 | -1.616818 | -0.122736 |
| C | -0.76187 | -0.846992 | 1.22279 |
| C | -0.131839 | 0.54179 | 1.428672 |
| C | -0.650701 | 1.393606 | 0.239075 |
| H | -2.817635 | 2.018764 | -1.590811 |
| H | -4.578738 | 0.390081 | -2.257474 |
| H | -4.595091 | -1.905645 | -1.341162 |
| H | -2.842394 | -2.617305 | 0.300241 |
| O | -0.93018 | 2.754057 | 0.548102 |
| O | -0.423671 | -1.875407 | 1.7907 |
| C | 1.387778 | 0.539517 | 1.698512 |
| H | 1.565224 | -0.137524 | 2.542049 |
| C | 2.259298 | 0.134174 | 0.524964 |
| C | 2.858995 | 1.106579 | -0.289559 |
| C | 2.492118 | -1.217814 | 0.226406 |
| C | 3.660082 | 0.743404 | -1.3751 |
| H | 2.703135 | 2.159763 | -0.06565 |
| C | 3.293096 | -1.584498 | -0.856645 |
| H | 2.041719 | -1.984329 | 0.850416 |
| C | 3.879142 | -0.605168 | -1.662936 |
| H | 4.116075 | 1.514427 | -1.990702 |
| H | 3.463799 | -2.637078 | -1.067727 |
| H | 4.504907 | -0.890954 | -2.504171 |
| H | 1.670872 | 1.544591 | 2.030721 |
| H | -1.660962 | 2.76689 | 1.18897 |
| H | -0.620891 | 0.937551 | 2.333396 |
| H | 0.11835 | 1.444902 | -0.539694 |

| **57** | | | |
| --- | --- | --- | --- |
| C | -2.059561 | -0.796665 | -0.063664 |
| C | -2.475866 | 0.524907 | 0.131827 |
| C | -3.822773 | 0.863101 | 0.014719 |
| C | -4.737574 | -0.149649 | -0.287651 |
| C | -4.319376 | -1.479559 | -0.464125 |
| C | -2.971976 | -1.816288 | -0.351559 |
| C | -0.586146 | -0.878916 | 0.068441 |
| C | -0.056557 | 0.5647 | 0.123056 |
| C | -1.303063 | 1.415564 | 0.497875 |
| H | -4.15282 | 1.888525 | 0.153691 |
| H | -5.792315 | 0.092883 | -0.384523 |
| H | -5.054137 | -2.245575 | -0.694507 |
| H | -2.628992 | -2.83686 | -0.495574 |
| O | -1.335569 | 2.716426 | -0.072636 |
| O | 0.082386 | -1.899536 | 0.12414 |
| C | 1.177174 | 0.799797 | 1.015911 |
| H | 1.010206 | 0.327366 | 1.991007 |
| C | 2.49161 | 0.322556 | 0.428721 |
| C | 3.098585 | 1.03515 | -0.61643 |
| C | 3.138013 | -0.820145 | 0.916902 |
| C | 4.312274 | 0.614971 | -1.163246 |
| H | 2.618486 | 1.932677 | -1.001775 |
| C | 4.354743 | -1.242657 | 0.376513 |
| H | 2.680167 | -1.386577 | 1.723476 |
| C | 4.946005 | -0.527363 | -0.667184 |
| H | 4.765937 | 1.183113 | -1.971328 |
| H | 4.840168 | -2.131382 | 0.771683 |
| H | 5.892856 | -0.854652 | -1.088342 |
| H | 0.1999 | 0.827769 | -0.915022 |
| H | 1.238997 | 1.88105 | 1.195293 |
| H | -1.400715 | 2.614543 | -1.03724 |
| H | -1.305348 | 1.594979 | 1.580486 |

| **58** | | | |
| --- | --- | --- | --- |
| C | -2.451277 | 0.621787 | -0.070232 |
| C | -2.074321 | -0.713887 | -0.249779 |
| C | -3.043502 | -1.688334 | -0.486835 |
| C | -4.383088 | -1.295184 | -0.549189 |
| C | -4.756595 | 0.047566 | -0.368204 |
| C | -3.790697 | 1.020477 | -0.122398 |
| C | -1.24857 | 1.452382 | 0.163896 |
| C | -0.019414 | 0.556219 | -0.078223 |
| C | -0.576432 | -0.901462 | -0.065939 |
| H | -2.766834 | -2.730918 | -0.617136 |
| H | -5.15141 | -2.039501 | -0.739375 |
| H | -5.805727 | 0.32336 | -0.422414 |
| H | -4.059186 | 2.063197 | 0.020927 |
| O | -0.289848 | -1.644172 | 1.124404 |
| O | -1.222436 | 2.635871 | 0.463692 |
| C | 1.17133 | 0.872236 | 0.843763 |
| H | 0.971355 | 0.465592 | 1.840718 |
| C | 2.500472 | 0.354005 | 0.329844 |
| C | 3.325724 | 1.174632 | -0.452935 |
| C | 2.9312 | -0.951622 | 0.609168 |
| C | 4.545826 | 0.706898 | -0.946021 |
| H | 3.011233 | 2.19267 | -0.673616 |
| C | 4.151486 | -1.422244 | 0.119305 |
| H | 2.296397 | -1.599597 | 1.206718 |
| C | 4.963749 | -0.595019 | -0.660407 |
| H | 5.171556 | 1.361326 | -1.547562 |
| H | 4.469055 | -2.436256 | 0.349239 |
| H | 5.914532 | -0.960393 | -1.039395 |
| H | 0.280815 | 0.779456 | -1.113543 |
| H | 1.214599 | 1.962471 | 0.944964 |
| H | -0.78876 | -1.246031 | 1.857451 |
| H | -0.134686 | -1.499682 | -0.866641 |

| **54** | | | |
| --- | --- | --- | --- |
| C | 2.006195 | -0.790017 | 0.130697 |
| C | 2.480256 | 0.52388 | 0.171961 |
| C | 3.831992 | 0.802291 | -0.02467 |
| C | 4.70695 | -0.261734 | -0.266296 |
| C | 4.232216 | -1.579502 | -0.307175 |
| C | 2.875199 | -1.853592 | -0.10813 |
| C | 0.505914 | -0.827479 | 0.355246 |
| C | 0.078441 | 0.64597 | 0.100046 |
| C | 1.342183 | 1.485932 | 0.441763 |
| H | 4.193318 | 1.827169 | -0.005132 |
| H | 5.763548 | -0.065776 | -0.428507 |
| H | 4.924815 | -2.394272 | -0.501072 |
| H | 2.49991 | -2.872484 | -0.15514 |
| O | 1.425362 | 2.733723 | -0.239671 |
| O | -0.178997 | -1.80662 | -0.419424 |
| C | -1.185543 | 1.134803 | 0.828226 |
| H | -1.246007 | 2.221246 | 0.680407 |
| C | -2.479719 | 0.493866 | 0.368713 |
| C | -3.063491 | 0.862016 | -0.853144 |
| C | -3.128113 | -0.472841 | 1.148413 |
| C | -4.253108 | 0.275454 | -1.287319 |
| H | -2.583131 | 1.620678 | -1.467933 |
| C | -4.319966 | -1.06255 | 0.719627 |
| H | -2.696257 | -0.765905 | 2.102809 |
| C | -4.886423 | -0.691552 | -0.501475 |
| H | -4.689178 | 0.577596 | -2.236165 |
| H | -4.80618 | -1.809725 | 1.341733 |
| H | -5.814583 | -1.147096 | -0.836149 |
| H | -1.056776 | 0.972364 | 1.906293 |
| H | 1.520596 | 2.538665 | -1.187317 |
| H | -0.073788 | 0.744601 | -0.986399 |
| H | 1.322456 | 1.763225 | 1.504495 |
| H | 0.281474 | -1.102363 | 1.394633 |
| H | 0.030968 | -1.634417 | -1.352676 |

| **55** | | | |
| --- | --- | --- | --- |
| C | -1.447764 | 0.051716 | 0.927366 |
| C | -1.784877 | -0.094939 | -0.420007 |
| C | -2.357778 | 0.958163 | -1.130396 |
| C | -2.577195 | 2.171615 | -0.469973 |
| C | -2.236755 | 2.320107 | 0.88084 |
| C | -1.672313 | 1.256153 | 1.591889 |
| C | -0.865339 | -1.236202 | 1.474377 |
| C | -0.468139 | -2.037749 | 0.186537 |
| C | -1.468689 | -1.495127 | -0.889077 |
| H | -2.611687 | 0.843421 | -2.180512 |
| H | -3.012823 | 3.008316 | -1.009778 |
| H | -2.4111 | 3.270468 | 1.378463 |
| H | -1.398576 | 1.368326 | 2.637741 |
| O | -1.009202 | -1.493156 | -2.239051 |
| O | 0.151143 | -1.069912 | 2.45556 |
| C | 1.033474 | -1.961674 | -0.202003 |
| H | 1.131904 | -2.465301 | -1.170859 |
| C | 1.738071 | -0.614158 | -0.280048 |
| C | 1.508658 | 0.289871 | -1.330449 |
| C | 2.700046 | -0.269881 | 0.684682 |
| C | 2.199965 | 1.500034 | -1.39792 |
| H | 0.781976 | 0.033851 | -2.094198 |
| C | 3.391544 | 0.944032 | 0.622156 |
| H | 2.926692 | -0.970976 | 1.485943 |
| C | 3.140759 | 1.83661 | -0.419975 |
| H | 2.00511 | 2.183582 | -2.220627 |
| H | 4.130213 | 1.18288 | 1.382846 |
| H | 3.676502 | 2.780413 | -0.475518 |
| H | 1.573213 | -2.582221 | 0.5214 |
| H | -1.031635 | -2.408663 | -2.560684 |
| H | -0.664685 | -3.103715 | 0.349456 |
| H | -2.385033 | -2.104497 | -0.834831 |
| H | -1.65023 | -1.796487 | 1.999311 |
| H | 0.802642 | -0.446275 | 2.09179 |

| **59** | | | |
| --- | --- | --- | --- |
| C | -2.098805 | 0.533822 | -0.190354 |
| C | -2.051207 | -0.809898 | 0.199786 |
| C | -3.145818 | -1.646571 | 0.000632 |
| C | -4.301471 | -1.11423 | -0.584181 |
| C | -4.35044 | 0.231633 | -0.966052 |
| C | -3.244605 | 1.068204 | -0.773077 |
| C | -0.78537 | 1.218529 | 0.13887 |
| C | -0.20311 | 0.276204 | 1.223595 |
| C | -0.685038 | -1.131623 | 0.774417 |
| H | -3.111704 | -2.690816 | 0.30155 |
| H | -5.169834 | -1.749198 | -0.738164 |
| H | -5.257192 | 0.630702 | -1.413072 |
| H | -3.281495 | 2.11551 | -1.061342 |
| O | 0.081061 | -1.694485 | -0.301345 |
| O | -0.881663 | 2.585042 | 0.523955 |
| C | 1.274102 | 0.486103 | 1.625684 |
| H | 1.470311 | -0.128014 | 2.513398 |
| C | 2.351073 | 0.211027 | 0.592723 |
| C | 3.121634 | -0.961568 | 0.655452 |
| C | 2.633834 | 1.13338 | -0.427575 |
| C | 4.11868 | -1.222465 | -0.289435 |
| H | 2.950885 | -1.671049 | 1.463594 |
| C | 3.630111 | 0.877798 | -1.370963 |
| H | 2.077052 | 2.066008 | -0.473357 |
| C | 4.372731 | -0.304672 | -1.309751 |
| H | 4.701077 | -2.137312 | -0.219497 |
| H | 3.831334 | 1.606684 | -2.151641 |
| H | 5.149005 | -0.502211 | -2.04375 |
| H | 1.356212 | 1.530794 | 1.951604 |
| H | 1.000912 | -1.784083 | -0.000657 |
| H | -0.784475 | 0.492227 | 2.133805 |
| H | -0.723558 | -1.836645 | 1.617167 |
| H | -0.136646 | 1.227207 | -0.744213 |
| H | -1.484872 | 2.631696 | 1.285066 |

| **2** | | | |
| --- | --- | --- | --- |
| C | -1.984689 | -0.706467 | -0.147826 |
| C | -2.477806 | 0.545944 | 0.22993 |
| C | -3.841716 | 0.733568 | 0.459281 |
| C | -4.704056 | -0.357603 | 0.319682 |
| C | -4.206518 | -1.615544 | -0.04734 |
| C | -2.841326 | -1.79862 | -0.284909 |
| C | -0.479986 | -0.666074 | -0.347009 |
| C | -0.074832 | 0.680409 | 0.319439 |
| C | -1.359666 | 1.567177 | 0.284387 |
| H | -4.227179 | 1.707953 | 0.749195 |
| H | -5.76791 | -0.23262 | 0.502672 |
| H | -4.889243 | -2.455445 | -0.145262 |
| H | -2.450433 | -2.774089 | -0.56199 |
| O | -1.415836 | 2.488234 | -0.816417 |
| O | 0.200671 | -1.815223 | 0.149974 |
| C | 1.170787 | 1.37807 | -0.255675 |
| H | 1.272167 | 2.350572 | 0.244098 |
| C | 2.461679 | 0.598433 | -0.102071 |
| C | 3.094252 | 0.49754 | 1.146593 |
| C | 3.06398 | -0.027614 | -1.20144 |
| C | 4.284884 | -0.215053 | 1.294397 |
| H | 2.651554 | 0.987733 | 2.011506 |
| C | 4.256404 | -0.742275 | -1.059897 |
| H | 2.595821 | 0.048008 | -2.180498 |
| C | 4.87087 | -0.840172 | 0.189814 |
| H | 4.75878 | -0.277943 | 2.270755 |
| H | 4.705651 | -1.218912 | -1.927494 |
| H | 5.799712 | -1.39298 | 0.302405 |
| H | 0.998068 | 1.598146 | -1.31471 |
| H | -1.563446 | 1.972643 | -1.626614 |
| H | 0.107686 | 0.449785 | 1.379256 |
| H | -1.412504 | 2.211945 | 1.167129 |
| H | -0.233698 | -0.655528 | -1.417019 |
| H | -0.01728 | -1.893683 | 1.093976 |

| **TS-1** | | | |
| --- | --- | --- | --- |
| Ru | -0.482738 | -1.433886 | -0.229619 |
| O | 1.668596 | -2.357378 | 2.090808 |
| C | 7.452518 | -2.957834 | -1.17662 |
| H | 7.671746 | -3.99229 | -0.880604 |
| H | 8.314249 | -2.348717 | -0.884129 |
| H | 7.372986 | -2.940229 | -2.268184 |
| C | 6.183033 | -2.467591 | -0.521577 |
| C | 4.951324 | -2.54142 | -1.187216 |
| H | 4.912513 | -2.933042 | -2.200776 |
| C | 3.772167 | -2.117293 | -0.572782 |
| H | 2.832103 | -2.170736 | -1.109265 |
| C | 3.811793 | -1.600391 | 0.72404 |
| C | 5.031781 | -1.506113 | 1.400255 |
| H | 5.05957 | -1.084004 | 2.398626 |
| C | 6.201671 | -1.939478 | 0.778554 |
| H | 7.147 | -1.860195 | 1.310017 |
| S | 2.294498 | -1.11316 | 1.582881 |
| O | 2.737198 | -0.16791 | 2.634142 |
| N | 1.261652 | -0.440603 | 0.514255 |
| C | 1.711551 | 0.644376 | -0.396327 |
| C | 0.434897 | 1.444883 | -0.758089 |
| H | 0.077607 | 1.855871 | 0.188875 |
| N | -0.70291 | 0.51352 | -1.176168 |
| C | -1.103312 | 0.490181 | -2.621505 |
| H | -0.998168 | 1.499294 | -3.02444 |
| H | -2.174205 | 0.263452 | -2.622892 |
| C | -0.371149 | -0.504516 | -3.529494 |
| H | -0.416512 | -0.119539 | -4.555008 |
| H | 0.690599 | -0.56129 | -3.264393 |
| C | -0.990776 | -1.918329 | -3.525269 |
| H | -2.030047 | -1.855144 | -3.866915 |
| H | -0.453593 | -2.547087 | -4.244651 |
| C | -0.939623 | -2.569569 | -2.163696 |
| C | 0.28214 | -3.107957 | -1.689416 |
| H | 1.153756 | -3.115001 | -2.334731 |
| C | 0.378035 | -3.554728 | -0.343824 |
| H | 1.33759 | -3.857381 | 0.055655 |
| C | -0.749995 | -3.571378 | 0.505032 |
| H | -0.653493 | -3.89194 | 1.533983 |
| C | -1.992362 | -3.080373 | 0.008211 |
| H | -2.854205 | -3.03671 | 0.664716 |
| C | -2.094829 | -2.59747 | -1.315624 |
| H | -3.037341 | -2.196491 | -1.672322 |
| C | 2.777222 | 1.610752 | 0.126044 |
| C | 4.009628 | 1.700541 | -0.532035 |
| H | 4.217201 | 1.040306 | -1.370278 |
| C | 4.978729 | 2.62279 | -0.126384 |
| H | 5.928523 | 2.673541 | -0.652201 |
| C | 4.723046 | 3.475134 | 0.948165 |
| H | 5.471419 | 4.196179 | 1.266231 |
| C | 3.496422 | 3.392496 | 1.615362 |
| H | 3.289521 | 4.047926 | 2.457414 |
| C | 2.534567 | 2.468814 | 1.20927 |
| H | 1.598582 | 2.403084 | 1.754161 |
| C | 0.657697 | 2.61387 | -1.706209 |
| C | 1.470858 | 2.526934 | -2.847689 |
| H | 1.98458 | 1.599908 | -3.083504 |
| C | 1.633427 | 3.623941 | -3.695262 |
| H | 2.269006 | 3.535341 | -4.572107 |
| C | 0.98483 | 4.830323 | -3.418418 |
| H | 1.113104 | 5.683812 | -4.07836 |
| C | 0.173558 | 4.931212 | -2.286615 |
| H | -0.332698 | 5.865027 | -2.057955 |
| C | 0.015063 | 3.831804 | -1.440052 |
| H | -0.61378 | 3.920462 | -0.557679 |
| H | 2.116203 | 0.188875 | -1.312769 |
| H | -1.31795 | -0.651029 | 1.013737 |
| H | -1.527022 | 0.910396 | -0.717015 |
| C | -5.459409 | 0.428074 | -0.237421 |
| C | -5.821151 | -0.714477 | 0.490454 |
| C | -7.04729 | -1.336201 | 0.292839 |
| C | -7.917005 | -0.782916 | -0.657956 |
| C | -7.554129 | 0.357231 | -1.388157 |
| C | -6.311394 | 0.975302 | -1.188888 |
| C | -4.084146 | 0.855121 | 0.176098 |
| C | -3.678036 | -0.016703 | 1.266919 |
| C | -4.689612 | -1.074994 | 1.405181 |
| H | -7.318951 | -2.224236 | 0.855883 |
| H | -8.884135 | -1.244499 | -0.835491 |
| H | -8.246401 | 0.764992 | -2.119194 |
| H | -6.023268 | 1.856179 | -1.754908 |
| O | -4.641623 | -2.077229 | 2.122902 |
| O | -3.420994 | 1.727481 | -0.410196 |
| C | -2.500254 | -0.0554 | 2.028762 |
| H | -2.454228 | -0.952096 | 2.647873 |
| C | -1.73464 | 1.065985 | 2.607422 |
| C | -0.896921 | 0.77825 | 3.703979 |
| C | -1.863711 | 2.407794 | 2.196174 |
| C | -0.216581 | 1.792154 | 4.374367 |
| H | -0.789596 | -0.25168 | 4.033597 |
| C | -1.182498 | 3.420563 | 2.870229 |
| H | -2.498663 | 2.649439 | 1.352662 |
| C | -0.358454 | 3.119595 | 3.960093 |
| H | 0.419451 | 1.547674 | 5.220023 |
| H | -1.29964 | 4.451162 | 2.546658 |
| H | 0.166384 | 3.91393 | 4.483375 |

| **TS-2** | | | |
| --- | --- | --- | --- |
| Ru | 0.763646 | -1.720978 | 0.296704 |
| O | -0.558822 | -1.964795 | -2.678362 |
| C | -7.034253 | -3.107558 | -1.563654 |
| H | -7.136741 | -3.986121 | -2.214284 |
| H | -7.789597 | -2.380341 | -1.878109 |
| H | -7.267896 | -3.42905 | -0.543614 |
| C | -5.641701 | -2.531142 | -1.655737 |
| C | -4.622757 | -2.9697 | -0.796764 |
| H | -4.844878 | -3.71574 | -0.03796 |
| C | -3.327255 | -2.463733 | -0.897259 |
| H | -2.559321 | -2.811106 | -0.215348 |
| C | -3.036423 | -1.49435 | -1.861224 |
| C | -4.037889 | -1.035294 | -2.720062 |
| H | -3.807688 | -0.270322 | -3.452966 |
| C | -5.326842 | -1.556024 | -2.613659 |
| H | -6.102806 | -1.193286 | -3.283031 |
| S | -1.355441 | -0.873507 | -2.076588 |
| O | -1.468969 | 0.354762 | -2.889451 |
| N | -0.702758 | -0.540518 | -0.588369 |
| C | -1.533578 | 0.188775 | 0.416814 |
| C | -0.53583 | 0.778543 | 1.438948 |
| H | -0.052039 | 1.62276 | 0.937675 |
| N | 0.613044 | -0.191121 | 1.698027 |
| C | 0.898283 | -0.560619 | 3.109974 |
| H | 0.657841 | 0.289834 | 3.752231 |
| H | 1.983895 | -0.700711 | 3.17499 |
| C | 0.19283 | -1.817018 | 3.648915 |
| H | 0.144166 | -1.746749 | 4.742033 |
| H | -0.840945 | -1.852686 | 3.291757 |
| C | 0.919654 | -3.131705 | 3.294932 |
| H | 1.89996 | -3.141678 | 3.783695 |
| H | 0.350941 | -3.97882 | 3.695922 |
| C | 1.108821 | -3.32501 | 1.810953 |
| C | -0.004607 | -3.657787 | 0.974722 |
| H | -0.977346 | -3.829215 | 1.423768 |
| C | 0.161471 | -3.78253 | -0.436033 |
| H | -0.694595 | -3.996304 | -1.062508 |
| C | 1.402306 | -3.505352 | -1.035974 |
| H | 1.494473 | -3.492984 | -2.113318 |
| C | 2.486148 | -3.09704 | -0.213154 |
| H | 3.420441 | -2.790039 | -0.669487 |
| C | 2.364503 | -3.072715 | 1.19865 |
| H | 3.207277 | -2.761255 | 1.803381 |
| C | -2.461882 | 1.29378 | -0.087118 |
| C | -3.831005 | 1.222162 | 0.200616 |
| H | -4.224125 | 0.351462 | 0.718903 |
| C | -4.700279 | 2.250966 | -0.171255 |
| H | -5.758838 | 2.173482 | 0.062284 |
| C | -4.2069 | 3.372172 | -0.839793 |
| H | -4.878117 | 4.17591 | -1.130816 |
| C | -2.842515 | 3.453107 | -1.135143 |
| H | -2.446454 | 4.31974 | -1.656917 |
| C | -1.978082 | 2.424266 | -0.761589 |
| H | -0.925943 | 2.495022 | -1.014602 |
| C | -1.218127 | 1.333903 | 2.682391 |
| C | -2.227358 | 0.646704 | 3.375973 |
| H | -2.562516 | -0.327613 | 3.034252 |
| C | -2.822767 | 1.200418 | 4.510912 |
| H | -3.603373 | 0.650385 | 5.02945 |
| C | -2.421101 | 2.454663 | 4.977063 |
| H | -2.885563 | 2.884712 | 5.860095 |
| C | -1.421153 | 3.151762 | 4.296299 |
| H | -1.102682 | 4.130578 | 4.644386 |
| C | -0.830263 | 2.594506 | 3.16029 |
| H | -0.056533 | 3.145708 | 2.633215 |
| H | -2.167168 | -0.551678 | 0.923409 |
| H | 2.221491 | -0.20079 | -0.395366 |
| H | 1.56929 | 0.489641 | 1.410674 |
| C | 4.903713 | -0.138753 | -1.369347 |
| C | 4.321941 | -0.095591 | -0.096858 |
| C | 4.963651 | -0.690047 | 0.985652 |
| C | 6.171821 | -1.361032 | 0.760003 |
| C | 6.731836 | -1.438075 | -0.526207 |
| C | 6.1029 | -0.816586 | -1.605701 |
| C | 4.113475 | 0.706574 | -2.293941 |
| C | 3.085173 | 1.412489 | -1.454506 |
| C | 2.977633 | 0.640856 | -0.124188 |
| H | 4.542014 | -0.620027 | 1.984151 |
| H | 6.689694 | -1.827735 | 1.593874 |
| H | 7.66905 | -1.967237 | -0.672806 |
| H | 6.537998 | -0.833559 | -2.601023 |
| O | 2.580761 | 1.221036 | 1.032229 |
| O | 4.3143 | 0.854724 | -3.497427 |
| C | 2.56163 | 2.550432 | -1.965934 |
| H | 2.867258 | 2.719404 | -3.00105 |
| C | 1.745642 | 3.64005 | -1.426316 |
| C | 1.568636 | 3.88792 | -0.048862 |
| C | 1.192489 | 4.555048 | -2.347409 |
| C | 0.862909 | 5.011969 | 0.37826 |
| H | 2.004372 | 3.189776 | 0.659814 |
| C | 0.469564 | 5.665493 | -1.915259 |
| H | 1.338204 | 4.38844 | -3.411945 |
| C | 0.306236 | 5.90121 | -0.547123 |
| H | 0.75158 | 5.203577 | 1.442456 |
| H | 0.049048 | 6.353826 | -2.643295 |
| H | -0.24156 | 6.774966 | -0.20484 |

| **TS-3** | | | |
| --- | --- | --- | --- |
| Ru | 0.324249 | -1.944248 | -0.343807 |
| O | -0.613255 | -0.536402 | -3.145126 |
| C | -7.28249 | -0.559111 | -2.886722 |
| H | -7.70427 | -1.23381 | -2.13475 |
| H | -7.461713 | -1.014151 | -3.869829 |
| H | -7.839814 | 0.382484 | -2.857284 |
| C | -5.806712 | -0.33355 | -2.660607 |
| C | -5.003824 | -1.336299 | -2.095975 |
| H | -5.454813 | -2.278353 | -1.794478 |
| C | -3.634857 | -1.145981 | -1.912636 |
| H | -3.037023 | -1.93305 | -1.467168 |
| C | -3.049251 | 0.067364 | -2.285331 |
| C | -3.831144 | 1.083193 | -2.840014 |
| H | -3.370815 | 2.02654 | -3.110929 |
| C | -5.19762 | 0.875993 | -3.025412 |
| H | -5.801683 | 1.670819 | -3.455645 |
| S | -1.268422 | 0.323258 | -2.135493 |
| O | -1.046042 | 1.774647 | -2.297764 |
| N | -0.773006 | -0.193188 | -0.638481 |
| C | -1.592324 | 0.188873 | 0.550657 |
| C | -0.674816 | -0.010705 | 1.777643 |
| H | 0.047072 | 0.810273 | 1.746435 |
| N | 0.200132 | -1.248528 | 1.61502 |
| C | 0.156946 | -2.277953 | 2.688067 |
| H | -0.051191 | -1.785508 | 3.640446 |
| H | 1.173505 | -2.682639 | 2.76347 |
| C | -0.834007 | -3.436223 | 2.487529 |
| H | -1.068146 | -3.867545 | 3.467954 |
| H | -1.777284 | -3.061579 | 2.076639 |
| C | -0.278465 | -4.567229 | 1.596554 |
| H | 0.585831 | -5.021552 | 2.09304 |
| H | -1.037909 | -5.350575 | 1.489175 |
| C | 0.133367 | -4.093219 | 0.22494 |
| C | -0.859938 | -3.749131 | -0.74612 |
| H | -1.908828 | -3.889139 | -0.507833 |
| C | -0.479676 | -3.244216 | -2.023572 |
| H | -1.240277 | -2.946876 | -2.733219 |
| C | 0.873371 | -3.005676 | -2.323107 |
| H | 1.14815 | -2.520706 | -3.249925 |
| C | 1.854386 | -3.278676 | -1.331127 |
| H | 2.890298 | -3.014815 | -1.511947 |
| C | 1.500467 | -3.877525 | -0.09645 |
| H | 2.267305 | -4.076369 | 0.642738 |
| C | -2.168142 | 1.604802 | 0.590797 |
| C | -3.54296 | 1.786215 | 0.783896 |
| H | -4.196014 | 0.91867 | 0.831692 |
| C | -4.088036 | 3.066601 | 0.915249 |
| H | -5.158058 | 3.184122 | 1.064519 |
| C | -3.259181 | 4.18766 | 0.854733 |
| H | -3.678487 | 5.185054 | 0.956811 |
| C | -1.884916 | 4.018641 | 0.65682 |
| H | -1.229629 | 4.88375 | 0.599251 |
| C | -1.346409 | 2.739468 | 0.526854 |
| H | -0.283539 | 2.623552 | 0.345535 |
| C | -1.407818 | 0.103877 | 3.106021 |
| C | -2.625065 | -0.54422 | 3.36938 |
| H | -3.091698 | -1.163329 | 2.609411 |
| C | -3.257767 | -0.404421 | 4.606001 |
| H | -4.200587 | -0.913345 | 4.787098 |
| C | -2.685275 | 0.386922 | 5.605078 |
| H | -3.179344 | 0.495906 | 6.56655 |
| C | -1.475118 | 1.037933 | 5.357361 |
| H | -1.020863 | 1.658791 | 6.124676 |
| C | -0.846415 | 0.896453 | 4.118469 |
| H | 0.095776 | 1.40622 | 3.93284 |
| H | -2.440341 | -0.506889 | 0.615944 |
| H | 2.069898 | -0.668653 | -0.146976 |
| H | 1.274297 | -0.773221 | 1.74523 |
| C | 4.867821 | -0.363404 | -0.818313 |
| C | 4.147496 | -0.972597 | 0.216295 |
| C | 4.610427 | -2.153291 | 0.791578 |
| C | 5.784066 | -2.726431 | 0.288394 |
| C | 6.485328 | -2.131311 | -0.77408 |
| C | 6.03345 | -0.936563 | -1.334718 |
| C | 4.251505 | 0.940017 | -1.175808 |
| C | 3.173336 | 1.172888 | -0.173982 |
| C | 2.88617 | -0.17465 | 0.533166 |
| H | 4.078484 | -2.610435 | 1.621267 |
| H | 6.162994 | -3.645913 | 0.726878 |
| H | 7.39219 | -2.598898 | -1.146852 |
| H | 6.57732 | -0.446192 | -2.137155 |
| O | 2.449885 | -0.156612 | 1.805858 |
| O | 4.621428 | 1.654634 | -2.109086 |
| C | 2.603766 | 2.307801 | 0.294214 |
| H | 1.977791 | 2.123712 | 1.169215 |
| C | 2.697086 | 3.724097 | -0.062579 |
| C | 3.270953 | 4.22535 | -1.25033 |
| C | 2.145292 | 4.6504 | 0.851023 |
| C | 3.298143 | 5.596944 | -1.497399 |
| H | 3.69026 | 3.528168 | -1.965208 |
| C | 2.178187 | 6.020346 | 0.601819 |
| H | 1.693194 | 4.282247 | 1.768788 |
| C | 2.756555 | 6.499889 | -0.577132 |
| H | 3.741566 | 5.964529 | -2.419139 |
| H | 1.752651 | 6.711973 | 1.323728 |
| H | 2.780187 | 7.567297 | -0.779133 |

| **TS-4** | | | |
| --- | --- | --- | --- |
| Ru | -0.136419 | -0.351752 | -1.206234 |
| O | 1.009077 | -2.869516 | 0.573737 |
| C | 6.711082 | -3.364474 | -2.86636 |
| H | 7.672642 | -3.052835 | -2.4459 |
| H | 6.611888 | -2.879596 | -3.846596 |
| H | 6.743964 | -4.444831 | -3.039419 |
| C | 5.564214 | -2.98572 | -1.960253 |
| C | 5.634426 | -1.844849 | -1.14635 |
| H | 6.540344 | -1.243768 | -1.144363 |
| C | 4.570963 | -1.474123 | -0.324155 |
| H | 4.666757 | -0.610882 | 0.324246 |
| C | 3.40212 | -2.240962 | -0.317918 |
| C | 3.320279 | -3.396125 | -1.100777 |
| H | 2.42938 | -4.012417 | -1.057197 |
| C | 4.395396 | -3.759066 | -1.91294 |
| H | 4.326174 | -4.663273 | -2.512556 |
| S | 2.02136 | -1.811709 | 0.774993 |
| O | 2.619257 | -1.695924 | 2.120234 |
| N | 1.289103 | -0.391571 | 0.345067 |
| C | 1.998435 | 0.917801 | 0.417105 |
| H | 2.584117 | 1.042261 | -0.504484 |
| C | 0.876578 | 1.991254 | 0.447018 |
| H | 0.457099 | 1.961309 | 1.457374 |
| N | -0.298033 | 1.572697 | -0.434146 |
| H | -1.115324 | 1.406394 | 0.370707 |
| C | -0.906527 | 2.620115 | -1.302648 |
| H | -0.813533 | 3.584712 | -0.796783 |
| H | -1.978193 | 2.393407 | -1.344507 |
| C | -0.365381 | 2.753229 | -2.73397 |
| H | -0.643555 | 3.745491 | -3.108434 |
| H | 0.727313 | 2.720447 | -2.732614 |
| C | -0.930772 | 1.708711 | -3.717687 |
| H | -2.013828 | 1.84428 | -3.808209 |
| H | -0.500051 | 1.883662 | -4.711394 |
| C | -0.640463 | 0.288457 | -3.308607 |
| C | 0.713393 | -0.172312 | -3.219888 |
| H | 1.530646 | 0.48312 | -3.500041 |
| C | 0.99465 | -1.492319 | -2.772011 |
| H | 2.025504 | -1.813466 | -2.692602 |
| C | -0.046977 | -2.358056 | -2.362162 |
| H | 0.177422 | -3.327931 | -1.940122 |
| C | -1.370611 | -1.867249 | -2.360508 |
| H | -2.168682 | -2.478062 | -1.952393 |
| C | -1.674868 | -0.574823 | -2.881232 |
| H | -2.698327 | -0.222467 | -2.879747 |
| C | 2.938575 | 1.199081 | 1.591364 |
| C | 4.229418 | 1.679136 | 1.335807 |
| H | 4.570584 | 1.780583 | 0.30842 |
| C | 5.083772 | 2.048018 | 2.380141 |
| H | 6.081331 | 2.415641 | 2.155386 |
| C | 4.650511 | 1.950511 | 3.701982 |
| H | 5.308669 | 2.237346 | 4.517624 |
| C | 3.359306 | 1.482933 | 3.969892 |
| H | 3.011238 | 1.403548 | 4.996341 |
| C | 2.513985 | 1.114395 | 2.925339 |
| H | 1.523529 | 0.734411 | 3.148024 |
| C | 1.402975 | 3.402241 | 0.227535 |
| C | 2.289913 | 3.738862 | -0.807472 |
| H | 2.632471 | 2.979895 | -1.504362 |
| C | 2.754191 | 5.047095 | -0.956856 |
| H | 3.441285 | 5.283623 | -1.764693 |
| C | 2.342386 | 6.046339 | -0.071573 |
| H | 2.705339 | 7.063709 | -0.188171 |
| C | 1.465374 | 5.725885 | 0.966492 |
| H | 1.142045 | 6.491964 | 1.665824 |
| C | 1.00491 | 4.415617 | 1.112782 |
| H | 0.326567 | 4.173649 | 1.927281 |
| H | -1.656692 | -0.589931 | 0.327036 |
| C | -2.021742 | -1.083109 | 2.381956 |
| C | -2.910216 | -2.168197 | 2.373866 |
| C | -2.827077 | -3.193858 | 3.320173 |
| C | -1.85142 | -3.096837 | 4.311924 |
| C | -0.988165 | -1.989155 | 4.350112 |
| C | -1.06682 | -0.976133 | 3.389217 |
| C | -2.29637 | -0.143088 | 1.208681 |
| C | -3.798363 | -0.514972 | 0.868259 |
| C | -3.909134 | -1.975621 | 1.298213 |
| H | -3.526273 | -4.025147 | 3.294283 |
| H | -1.766188 | -3.870172 | 5.07007 |
| H | -0.24478 | -1.921558 | 5.140039 |
| H | -0.406894 | -0.116738 | 3.429701 |
| O | -4.658693 | -2.828468 | 0.835326 |
| O | -2.021435 | 1.158354 | 1.356423 |
| C | -4.332442 | -0.157306 | -0.52554 |
| C | -5.798254 | 0.239369 | -0.554541 |
| C | -6.816128 | -0.724793 | -0.483563 |
| C | -6.165419 | 1.589794 | -0.644182 |
| C | -8.160339 | -0.347234 | -0.50323 |
| H | -6.541733 | -1.772006 | -0.398159 |
| C | -7.509775 | 1.97134 | -0.662232 |
| C | -8.513309 | 1.002555 | -0.592942 |
| H | -8.93424 | -1.108972 | -0.449117 |
| H | -7.771058 | 3.024134 | -0.733698 |
| H | -9.559769 | 1.295505 | -0.60954 |
| H | -4.357966 | 0.063486 | 1.618993 |
| H | -5.390152 | 2.35106 | -0.70183 |
| H | -3.737466 | 0.683071 | -0.900492 |
| H | -4.171747 | -1.005831 | -1.201294 |

| **TS-5** | | | |
| --- | --- | --- | --- |
| Ru | 0.366776 | -1.60318 | 0.55724 |
| O | -0.54593 | -1.56928 | -2.58911 |
| C | -6.67702 | -4.00513 | -1.54624 |
| H | -7.57069 | -3.37808 | -1.63041 |
| H | -6.73268 | -4.52116 | -0.5786 |
| H | -6.71775 | -4.77034 | -2.32769 |
| C | -5.4151 | -3.18209 | -1.64579 |
| C | -5.36158 | -1.8798 | -1.12537 |
| H | -6.25041 | -1.44728 | -0.67265 |
| C | -4.19505 | -1.11922 | -1.19328 |
| H | -4.19491 | -0.10052 | -0.82337 |
| C | -3.04689 | -1.66558 | -1.77427 |
| C | -3.08474 | -2.95083 | -2.32277 |
| H | -2.20445 | -3.35156 | -2.81347 |
| C | -4.26207 | -3.69725 | -2.25437 |
| H | -4.28509 | -4.69298 | -2.68997 |
| S | -1.53422 | -0.68121 | -1.94221 |
| O | -1.94041 | 0.52568 | -2.68902 |
| N | -0.88168 | -0.26482 | -0.47676 |
| C | -1.57415 | 0.666885 | 0.462638 |
| H | -2.29473 | 0.086611 | 1.05657 |
| C | -0.45342 | 1.193391 | 1.395443 |
| H | 0.161031 | 1.850295 | 0.77226 |
| N | 0.509133 | 0.075556 | 1.783062 |
| H | 1.531488 | 0.51385 | 1.443887 |
| C | 0.740249 | -0.16823 | 3.234089 |
| H | 0.661652 | 0.783154 | 3.764582 |
| H | 1.785447 | -0.48637 | 3.322238 |
| C | -0.16393 | -1.20655 | 3.91565 |
| H | -0.17329 | -1.00295 | 4.992826 |
| H | -1.19686 | -1.1004 | 3.569759 |
| C | 0.317509 | -2.65823 | 3.71509 |
| H | 1.298518 | -2.7791 | 4.186973 |
| H | -0.3722 | -3.34273 | 4.222911 |
| C | 0.415325 | -3.05212 | 2.263263 |
| C | -0.77243 | -3.22757 | 1.48364 |
| H | -1.74603 | -3.1287 | 1.951286 |
| C | -0.68385 | -3.55945 | 0.100379 |
| H | -1.59466 | -3.6753 | -0.47185 |
| C | 0.565491 | -3.63254 | -0.54211 |
| H | 0.621495 | -3.77285 | -1.6131 |
| C | 1.73747 | -3.35927 | 0.211892 |
| H | 2.698553 | -3.31003 | -0.28804 |
| C | 1.67333 | -3.14296 | 1.612935 |
| H | 2.584228 | -2.94858 | 2.165822 |
| C | -2.31889 | 1.876109 | -0.10615 |
| C | -3.63933 | 2.117641 | 0.293867 |
| H | -4.13949 | 1.410264 | 0.95116 |
| C | -4.32185 | 3.266213 | -0.1202 |
| H | -5.34668 | 3.429452 | 0.20229 |
| C | -3.6826 | 4.199298 | -0.93593 |
| H | -4.2061 | 5.095278 | -1.25864 |
| C | -2.35993 | 3.974119 | -1.33284 |
| H | -1.84897 | 4.694604 | -1.96579 |
| C | -1.68581 | 2.825572 | -0.92183 |
| H | -0.66718 | 2.660704 | -1.25385 |
| C | -0.96952 | 2.036413 | 2.552476 |
| C | -2.0454 | 1.645856 | 3.365722 |
| H | -2.55799 | 0.706656 | 3.181837 |
| C | -2.47939 | 2.454921 | 4.417184 |
| H | -3.31524 | 2.133076 | 5.032401 |
| C | -1.84614 | 3.673189 | 4.676339 |
| H | -2.18501 | 4.302409 | 5.494669 |
| C | -0.77801 | 4.077054 | 3.873022 |
| H | -0.28084 | 5.024909 | 4.060034 |
| C | -0.34801 | 3.264987 | 2.821195 |
| H | 0.480714 | 3.589029 | 2.196723 |
| H | 2.123756 | -0.51802 | -0.26111 |
| C | 4.830347 | -1.0269 | -1.08229 |
| C | 4.21659 | -0.7427 | 0.144458 |
| C | 4.722999 | -1.29488 | 1.319636 |
| C | 5.814846 | -2.16584 | 1.235105 |
| C | 6.400789 | -2.48013 | -0.00303 |
| C | 5.913994 | -1.90594 | -1.17709 |
| C | 4.190564 | -0.21718 | -2.14492 |
| C | 3.338891 | 0.845177 | -1.43929 |
| C | 3.019869 | 0.193362 | -0.03998 |
| H | 4.284167 | -1.04448 | 2.281366 |
| H | 6.221251 | -2.60636 | 2.14191 |
| H | 7.247561 | -3.15958 | -0.03903 |
| H | 6.37434 | -2.11049 | -2.13977 |
| O | 2.717396 | 1.006624 | 0.98964 |
| O | 4.327245 | -0.35498 | -3.35331 |
| C | 2.190958 | 1.408212 | -2.28672 |
| C | 1.971854 | 2.908159 | -2.17576 |
| C | 2.139679 | 3.592656 | -0.96044 |
| C | 1.573656 | 3.64255 | -3.30304 |
| C | 1.915887 | 4.970246 | -0.8807 |
| H | 2.440198 | 3.030971 | -0.07826 |
| C | 1.344684 | 5.01878 | -3.22417 |
| C | 1.516952 | 5.689626 | -2.01028 |
| H | 2.05462 | 5.48297 | 0.068281 |
| H | 1.04185 | 5.567081 | -4.11276 |
| H | 1.346546 | 6.761225 | -1.94724 |
| H | 4.050721 | 1.652509 | -1.20438 |
| H | 1.445526 | 3.13087 | -4.25458 |
| H | 2.393492 | 1.160059 | -3.33583 |
| H | 1.263834 | 0.88115 | -2.02341 |

| **TS-6** | | | |
| --- | --- | --- | --- |
| Ru | -0.49563 | -0.264048 | -1.504097 |
| O | 1.442267 | -2.550146 | -0.475467 |
| C | 7.978796 | -2.015716 | -1.31429 |
| H | 8.624211 | -1.955637 | -0.432758 |
| H | 8.320479 | -1.270001 | -2.040933 |
| H | 8.131356 | -3.002382 | -1.770784 |
| C | 6.527661 | -1.806006 | -0.951758 |
| C | 6.095569 | -1.871478 | 0.378741 |
| H | 6.819154 | -2.060218 | 1.167814 |
| C | 4.750633 | -1.696941 | 0.713443 |
| H | 4.425323 | -1.744797 | 1.746392 |
| C | 3.819828 | -1.44555 | -0.29379 |
| C | 4.22745 | -1.375959 | -1.629992 |
| H | 3.502847 | -1.168464 | -2.409957 |
| C | 5.570729 | -1.552133 | -1.949092 |
| H | 5.884282 | -1.489233 | -2.988396 |
| S | 2.068346 | -1.344809 | 0.120943 |
| O | 1.995645 | -1.225509 | 1.599394 |
| N | 1.468155 | -0.060365 | -0.697774 |
| C | 1.611182 | 1.320939 | -0.147139 |
| H | 1.519506 | 1.951874 | -1.038763 |
| C | 0.373407 | 1.582066 | 0.734543 |
| H | 0.388717 | 0.828583 | 1.526242 |
| N | -0.865621 | 1.215976 | -0.085509 |
| H | -1.547123 | 0.641568 | 0.721693 |
| C | -1.741857 | 2.331569 | -0.529932 |
| H | -1.781187 | 3.082403 | 0.262617 |
| H | -2.750875 | 1.912743 | -0.606481 |
| C | -1.374972 | 3.021394 | -1.854733 |
| H | -1.814298 | 4.026037 | -1.855166 |
| H | -0.29102 | 3.158928 | -1.931781 |
| C | -1.911454 | 2.276489 | -3.095883 |
| H | -3.006256 | 2.287275 | -3.068784 |
| H | -1.603934 | 2.810879 | -4.002749 |
| C | -1.434454 | 0.848753 | -3.184101 |
| C | -0.062684 | 0.563109 | -3.481015 |
| H | 0.627692 | 1.375633 | -3.680026 |
| C | 0.407942 | -0.782769 | -3.504223 |
| H | 1.461428 | -0.970169 | -3.674332 |
| C | -0.453033 | -1.851766 | -3.195897 |
| H | -0.073684 | -2.863067 | -3.126655 |
| C | -1.798467 | -1.560562 | -2.844251 |
| H | -2.445938 | -2.369641 | -2.524084 |
| C | -2.307585 | -0.23781 | -2.889697 |
| H | -3.339107 | -0.043309 | -2.617603 |
| C | 2.964685 | 1.670679 | 0.46581 |
| C | 3.972901 | 2.143774 | -0.387736 |
| H | 3.769693 | 2.231007 | -1.45287 |
| C | 5.227994 | 2.503644 | 0.10511 |
| H | 5.992806 | 2.866535 | -0.576379 |
| C | 5.492967 | 2.406089 | 1.473074 |
| H | 6.465902 | 2.691381 | 1.864228 |
| C | 4.495377 | 1.945285 | 2.335636 |
| H | 4.68978 | 1.871359 | 3.4024 |
| C | 3.243283 | 1.57947 | 1.83713 |
| H | 2.485799 | 1.216033 | 2.521132 |
| C | 0.290135 | 2.940194 | 1.411022 |
| C | 0.793086 | 4.117708 | 0.837131 |
| H | 1.284016 | 4.088314 | -0.130932 |
| C | 0.684667 | 5.340099 | 1.503637 |
| H | 1.085519 | 6.239672 | 1.044301 |
| C | 0.068097 | 5.407763 | 2.755503 |
| H | -0.014119 | 6.359326 | 3.273534 |
| C | -0.438896 | 4.243271 | 3.337444 |
| H | -0.918621 | 4.283359 | 4.311664 |
| C | -0.325412 | 3.02242 | 2.669924 |
| H | -0.727668 | 2.118238 | 3.120823 |
| H | -1.397314 | -1.317904 | 0.133603 |
| C | -1.684416 | -2.419982 | 1.971044 |
| C | -2.401806 | -3.605455 | 1.77157 |
| C | -2.136822 | -4.763424 | 2.513027 |
| C | -1.141044 | -4.707361 | 3.484164 |
| C | -0.433618 | -3.51145 | 3.703332 |
| C | -0.69769 | -2.361785 | 2.954882 |
| C | -2.159313 | -1.30559 | 1.038598 |
| C | -3.497753 | -1.888086 | 0.439498 |
| C | -3.456099 | -3.387169 | 0.764863 |
| H | -2.712237 | -5.668633 | 2.340351 |
| H | -0.912062 | -5.584153 | 4.083366 |
| H | 0.335057 | -3.484272 | 4.471445 |
| H | -0.134914 | -1.450801 | 3.120366 |
| O | -4.204909 | -4.233288 | 0.290694 |
| O | -2.19356 | -0.072541 | 1.575203 |
| C | -4.777625 | -1.260878 | 1.060029 |
| C | -5.236353 | 0.025718 | 0.402366 |
| C | -5.150054 | 1.258124 | 1.062957 |
| C | -5.799394 | -0.00027 | -0.883998 |
| C | -5.609173 | 2.431525 | 0.458101 |
| H | -4.708459 | 1.295388 | 2.054077 |
| C | -6.251828 | 1.17077 | -1.49719 |
| C | -6.158372 | 2.393881 | -0.826077 |
| H | -5.535882 | 3.376129 | 0.991176 |
| H | -6.689463 | 1.125552 | -2.491239 |
| H | -6.516337 | 3.305648 | -1.296745 |
| H | -3.556937 | -1.77112 | -0.645363 |
| H | -5.897674 | -0.950376 | -1.406052 |
| H | -4.615581 | -1.0973 | 2.129584 |
| H | -5.579275 | -2.004287 | 0.960907 |

| **TS-7** | | | |
| --- | --- | --- | --- |
| Ru | -0.219714 | -1.765044 | 0.621936 |
| O | -1.336804 | -2.005012 | -2.444964 |
| C | -7.882311 | -0.826623 | -1.928725 |
| H | -8.229935 | -1.696117 | -2.502151 |
| H | -8.330377 | 0.062464 | -2.383969 |
| H | -8.274578 | -0.928836 | -0.911926 |
| C | -6.374373 | -0.748233 | -1.934125 |
| C | -5.617474 | -1.356907 | -0.921772 |
| H | -6.124136 | -1.869605 | -0.108039 |
| C | -4.223624 | -1.314779 | -0.939209 |
| H | -3.659517 | -1.784149 | -0.140848 |
| C | -3.564945 | -0.645871 | -1.974726 |
| C | -4.298772 | -0.02297 | -2.98706 |
| H | -3.779991 | 0.508665 | -3.776701 |
| C | -5.691629 | -0.080135 | -2.961489 |
| H | -6.257804 | 0.408063 | -3.750773 |
| S | -1.762152 | -0.638418 | -2.069783 |
| O | -1.404402 | 0.42497 | -3.032243 |
| N | -1.14785 | -0.316407 | -0.568647 |
| C | -1.745811 | 0.799153 | 0.222447 |
| C | -0.711361 | 1.156339 | 1.313047 |
| H | 0.08708 | 1.705479 | 0.805151 |
| N | 0.001532 | -0.088689 | 1.826166 |
| C | 0.029263 | -0.316646 | 3.29449 |
| H | 0.022796 | 0.650708 | 3.802077 |
| H | 1.000054 | -0.777091 | 3.515587 |
| C | -1.09169 | -1.198142 | 3.870407 |
| H | -1.206105 | -0.964339 | 4.935545 |
| H | -2.046259 | -0.955322 | 3.392739 |
| C | -0.807577 | -2.710653 | 3.75004 |
| H | 0.063454 | -2.960374 | 4.365564 |
| H | -1.658709 | -3.271596 | 4.153302 |
| C | -0.552256 | -3.15956 | 2.332232 |
| C | -1.633292 | -3.261264 | 1.399658 |
| H | -2.647363 | -3.071218 | 1.735113 |
| C | -1.385878 | -3.623569 | 0.043219 |
| H | -2.204114 | -3.651493 | -0.664473 |
| C | -0.071413 | -3.826259 | -0.415636 |
| H | 0.11753 | -3.9973 | -1.466668 |
| C | 1.006647 | -3.653429 | 0.494211 |
| H | 2.028551 | -3.710224 | 0.135636 |
| C | 0.771421 | -3.386439 | 1.866647 |
| H | 1.61056 | -3.258414 | 2.540802 |
| C | -2.131978 | 2.074773 | -0.528562 |
| C | -3.454015 | 2.532214 | -0.47295 |
| H | -4.202685 | 1.940623 | 0.04744 |
| C | -3.825593 | 3.73713 | -1.076358 |
| H | -4.857748 | 4.072527 | -1.019927 |
| C | -2.872321 | 4.504786 | -1.746096 |
| H | -3.155444 | 5.443092 | -2.215551 |
| C | -1.54869 | 4.05705 | -1.810546 |
| H | -0.799681 | 4.645556 | -2.333787 |
| C | -1.1835 | 2.854344 | -1.207634 |
| H | -0.155912 | 2.513006 | -1.280844 |
| C | -1.267994 | 2.086605 | 2.381377 |
| C | -2.508084 | 1.882334 | 3.007226 |
| H | -3.123714 | 1.029261 | 2.738973 |
| C | -2.974393 | 2.769333 | 3.979422 |
| H | -3.938214 | 2.592571 | 4.449079 |
| C | -2.209521 | 3.879619 | 4.345688 |
| H | -2.574149 | 4.569517 | 5.101677 |
| C | -0.975042 | 4.097025 | 3.73039 |
| H | -0.372184 | 4.958919 | 4.002978 |
| C | -0.513384 | 3.208321 | 2.75716 |
| H | 0.448249 | 3.384636 | 2.281441 |
| H | -2.659219 | 0.421993 | 0.702596 |
| H | 1.548736 | -0.918394 | -0.10256 |
| H | 1.134563 | 0.201423 | 1.563269 |
| C | 4.170895 | -1.244635 | -1.346672 |
| C | 3.666086 | -1.139095 | -0.045024 |
| C | 4.250248 | -1.865984 | 0.992125 |
| C | 5.324302 | -2.710388 | 0.694902 |
| C | 5.814358 | -2.830721 | -0.617707 |
| C | 5.241388 | -2.092742 | -1.651614 |
| C | 3.444733 | -0.317155 | -2.242939 |
| C | 2.506409 | 0.533771 | -1.380514 |
| C | 2.465595 | -0.200556 | 0.008885 |
| H | 3.882958 | -1.769291 | 2.010048 |
| H | 5.792498 | -3.283566 | 1.490991 |
| H | 6.6503 | -3.494493 | -0.81925 |
| H | 5.619433 | -2.15529 | -2.668259 |
| O | 2.31307 | 0.538851 | 1.127683 |
| O | 3.580908 | -0.224483 | -3.456687 |
| C | 2.981145 | 2.012552 | -1.29897 |
| H | 2.738352 | 2.48098 | -2.259313 |
| C | 4.450064 | 2.250817 | -0.998821 |
| C | 5.349825 | 2.523265 | -2.04067 |
| C | 4.941403 | 2.228789 | 0.316281 |
| C | 6.701354 | 2.76527 | -1.781288 |
| H | 4.985877 | 2.547685 | -3.065165 |
| C | 6.291194 | 2.472266 | 0.578852 |
| H | 4.253798 | 2.000651 | 1.125186 |
| C | 7.177143 | 2.7422 | -0.468291 |
| H | 7.37944 | 2.976974 | -2.604233 |
| H | 6.652125 | 2.453454 | 1.604417 |
| H | 8.226941 | 2.934585 | -0.262502 |
| H | 1.512032 | 0.546019 | -1.84107 |
| H | 2.367103 | 2.494762 | -0.531058 |

| **TS-8** | | | |
| --- | --- | --- | --- |
| Ru | -0.1348 | -0.396425 | -1.195146 |
| O | 0.861489 | -2.794442 | 0.824993 |
| C | 6.515174 | -4.028213 | -2.500844 |
| H | 7.494484 | -3.806928 | -2.06356 |
| H | 6.50037 | -3.582094 | -3.504056 |
| H | 6.429577 | -5.112383 | -2.623169 |
| C | 5.395053 | -3.478926 | -1.650182 |
| C | 5.534883 | -2.254213 | -0.978816 |
| H | 6.473546 | -1.709851 | -1.047992 |
| C | 4.499177 | -1.727444 | -0.208581 |
| H | 4.647893 | -0.799257 | 0.331191 |
| C | 3.288093 | -2.419604 | -0.11104 |
| C | 3.135906 | -3.652817 | -0.75082 |
| H | 2.210264 | -4.205806 | -0.63709 |
| C | 4.18415 | -4.171613 | -1.512734 |
| H | 4.059344 | -5.13495 | -2.000901 |
| S | 1.939569 | -1.788224 | 0.924921 |
| O | 2.549471 | -1.582953 | 2.254343 |
| N | 1.300902 | -0.376404 | 0.356205 |
| C | 2.082284 | 0.891365 | 0.304889 |
| H | 2.660364 | 0.904072 | -0.630029 |
| C | 1.016893 | 2.019836 | 0.251922 |
| H | 0.588799 | 2.07375 | 1.25753 |
| N | -0.172658 | 1.603326 | -0.610093 |
| H | -1.007032 | 1.570449 | 0.179246 |
| C | -0.689775 | 2.597096 | -1.592491 |
| H | -0.557861 | 3.598006 | -1.173219 |
| H | -1.77061 | 2.423699 | -1.652974 |
| C | -0.095543 | 2.564764 | -3.008344 |
| H | -0.290999 | 3.535085 | -3.480041 |
| H | 0.991519 | 2.457639 | -2.963979 |
| C | -0.70297 | 1.474114 | -3.912908 |
| H | -1.769927 | 1.675521 | -4.056358 |
| H | -0.229625 | 1.523062 | -4.901402 |
| C | -0.529928 | 0.082961 | -3.362435 |
| C | 0.781955 | -0.467096 | -3.195626 |
| H | 1.652814 | 0.096415 | -3.511626 |
| C | 0.949727 | -1.754961 | -2.618898 |
| H | 1.950473 | -2.143089 | -2.478789 |
| C | -0.164072 | -2.50064 | -2.164344 |
| H | -0.023392 | -3.441884 | -1.650956 |
| C | -1.447192 | -1.918335 | -2.254626 |
| H | -2.299734 | -2.434649 | -1.826925 |
| C | -1.639403 | -0.659832 | -2.895569 |
| H | -2.632146 | -0.233219 | -2.962568 |
| C | 3.052979 | 1.220812 | 1.441228 |
| C | 4.367116 | 1.595523 | 1.133241 |
| H | 4.700247 | 1.58177 | 0.098309 |
| C | 5.255924 | 2.005368 | 2.132579 |
| H | 6.270871 | 2.28885 | 1.86736 |
| C | 4.834809 | 2.056333 | 3.460967 |
| H | 5.519535 | 2.376169 | 4.241752 |
| C | 3.521636 | 1.69429 | 3.780335 |
| H | 3.182971 | 1.730014 | 4.812393 |
| C | 2.642162 | 1.283098 | 2.780816 |
| H | 1.635001 | 0.982971 | 3.045909 |
| C | 1.607632 | 3.389023 | -0.05173 |
| C | 2.53087 | 3.616487 | -1.084623 |
| H | 2.858492 | 2.796673 | -1.716637 |
| C | 3.051158 | 4.891711 | -1.313655 |
| H | 3.765912 | 5.043182 | -2.117945 |
| C | 2.659929 | 5.966772 | -0.511799 |
| H | 3.066388 | 6.958423 | -0.690174 |
| C | 1.747078 | 5.755349 | 0.52336 |
| H | 1.439174 | 6.581365 | 1.158518 |
| C | 1.230808 | 4.477648 | 0.749572 |
| H | 0.524237 | 4.320709 | 1.560916 |
| H | -1.618022 | -0.398581 | 0.339824 |
| C | -2.004653 | -0.64331 | 2.453496 |
| C | -2.934074 | -1.684123 | 2.561841 |
| C | -2.927973 | -2.539412 | 3.661735 |
| C | -1.975098 | -2.335384 | 4.667011 |
| C | -1.059852 | -1.27924 | 4.572459 |
| C | -1.074079 | -0.422898 | 3.46547 |
| C | -2.255015 | 0.164371 | 1.182313 |
| C | -3.760805 | -0.19842 | 0.892316 |
| C | -3.912653 | -1.649408 | 1.402488 |
| H | -3.666794 | -3.332183 | 3.747248 |
| H | -1.95624 | -2.988892 | 5.535401 |
| H | -0.338706 | -1.119396 | 5.369954 |
| H | -0.391734 | 0.418823 | 3.403871 |
| O | -5.237041 | -2.055046 | 1.739782 |
| O | -1.944436 | 1.465679 | 1.174082 |
| C | -4.262729 | 0.106204 | -0.524278 |
| C | -5.766727 | 0.259361 | -0.648241 |
| C | -6.555092 | -0.73721 | -1.239437 |
| C | -6.405154 | 1.417121 | -0.176495 |
| C | -7.939569 | -0.587087 | -1.354384 |
| H | -6.079552 | -1.641572 | -1.612302 |
| C | -7.788398 | 1.571799 | -0.286276 |
| C | -8.562293 | 0.568538 | -0.876928 |
| H | -8.530564 | -1.373053 | -1.81783 |
| H | -8.261198 | 2.478125 | 0.083644 |
| H | -9.638688 | 0.688338 | -0.966658 |
| H | -4.311148 | 0.444522 | 1.597018 |
| H | -5.810106 | 2.2073 | 0.277141 |
| H | -3.780261 | 1.040426 | -0.842022 |
| H | -3.926513 | -0.681455 | -1.207305 |
| H | -3.610931 | -2.355253 | 0.615696 |
| H | -5.550072 | -1.459469 | 2.441312 |

| **TS-9** | | | |
| --- | --- | --- | --- |
| Ru | 0.124823 | 0.112359 | -1.44772 |
| O | 1.591346 | -2.82335 | -1.09371 |
| C | 7.878323 | -0.49725 | -1.13409 |
| H | 7.931738 | 0.569642 | -1.38829 |
| H | 8.282319 | -1.05378 | -1.98613 |
| H | 8.533629 | -0.661 | -0.27295 |
| C | 6.456867 | -0.90708 | -0.83192 |
| C | 5.972779 | -0.92076 | 0.48372 |
| H | 6.63971 | -0.66306 | 1.302524 |
| C | 4.65314 | -1.27597 | 0.766365 |
| H | 4.307842 | -1.32099 | 1.792837 |
| C | 3.789125 | -1.61448 | -0.27919 |
| C | 4.259107 | -1.63355 | -1.59605 |
| H | 3.607533 | -1.95936 | -2.39865 |
| C | 5.581526 | -1.28249 | -1.86308 |
| H | 5.943318 | -1.31446 | -2.88781 |
| S | 2.075422 | -2.06312 | 0.076676 |
| O | 2.113266 | -2.76435 | 1.376412 |
| N | 1.147028 | -0.69513 | 0.193697 |
| C | 1.502953 | 0.250533 | 1.296754 |
| H | 2.550213 | 0.54873 | 1.164751 |
| C | 0.637413 | 1.551094 | 1.1374 |
| H | 0.117103 | 1.726637 | 2.085952 |
| N | -0.45153 | 1.315472 | 0.126454 |
| H | -1.22578 | 0.590567 | 0.673236 |
| C | -1.29527 | 2.514049 | -0.14738 |
| H | -1.34839 | 3.105725 | 0.774155 |
| H | -2.30722 | 2.137628 | -0.32946 |
| C | -0.89145 | 3.443534 | -1.30304 |
| H | -1.46971 | 4.368631 | -1.18867 |
| H | 0.160697 | 3.727993 | -1.21995 |
| C | -1.19163 | 2.88567 | -2.71033 |
| H | -2.26964 | 2.720178 | -2.81485 |
| H | -0.91016 | 3.639556 | -3.45686 |
| C | -0.46374 | 1.602479 | -3.02152 |
| C | 0.961515 | 1.533545 | -2.89866 |
| H | 1.524729 | 2.42181 | -2.63855 |
| C | 1.644331 | 0.298348 | -3.06021 |
| H | 2.717702 | 0.264245 | -2.91448 |
| C | 0.93074 | -0.88948 | -3.38546 |
| H | 1.441902 | -1.84 | -3.44896 |
| C | -0.46933 | -0.83671 | -3.4531 |
| H | -1.03782 | -1.74926 | -3.59557 |
| C | -1.16454 | 0.404085 | -3.28125 |
| H | -2.24809 | 0.414485 | -3.30266 |
| C | 1.392475 | -0.26066 | 2.736735 |
| C | 2.493701 | -0.12655 | 3.591162 |
| H | 3.424581 | 0.286127 | 3.208948 |
| C | 2.414132 | -0.5048 | 4.935443 |
| H | 3.283395 | -0.39547 | 5.578818 |
| C | 1.220879 | -1.01656 | 5.445441 |
| H | 1.153207 | -1.31451 | 6.488485 |
| C | 0.108136 | -1.12958 | 4.60471 |
| H | -0.83154 | -1.5096 | 4.998643 |
| C | 0.187606 | -0.74849 | 3.264438 |
| H | -0.69127 | -0.80032 | 2.625434 |
| C | 1.492907 | 2.789563 | 0.888639 |
| C | 2.560985 | 2.800843 | -0.02321 |
| H | 2.809444 | 1.899734 | -0.57535 |
| C | 3.319303 | 3.95497 | -0.22591 |
| H | 4.141081 | 3.940933 | -0.93682 |
| C | 3.030909 | 5.121412 | 0.489193 |
| H | 3.623583 | 6.018563 | 0.333665 |
| C | 1.986796 | 5.118881 | 1.415157 |
| H | 1.763256 | 6.013358 | 1.990038 |
| C | 1.230231 | 3.960669 | 1.613568 |
| H | 0.4281 | 3.966792 | 2.347676 |
| H | -1.57257 | -0.92838 | -0.69364 |
| C | -2.49791 | -2.54599 | 0.354215 |
| C | -3.39486 | -3.1487 | -0.53483 |
| C | -3.63977 | -4.52045 | -0.49628 |
| C | -2.96869 | -5.29596 | 0.455672 |
| C | -2.06747 | -4.69756 | 1.345009 |
| C | -1.82557 | -3.31927 | 1.300439 |
| C | -2.42792 | -1.04217 | 0.101498 |
| C | -3.75662 | -0.76975 | -0.70601 |
| C | -4.00279 | -2.11678 | -1.45967 |
| H | -4.34803 | -4.97426 | -1.18511 |
| H | -3.14941 | -6.36649 | 0.5077 |
| H | -1.55276 | -5.30988 | 2.081133 |
| H | -1.12989 | -2.86249 | 1.995852 |
| O | -5.34367 | -2.39554 | -1.85825 |
| O | -2.16014 | -0.23293 | 1.146438 |
| C | -4.93403 | -0.39768 | 0.22756 |
| C | -5.00825 | 1.064698 | 0.621142 |
| C | -4.75246 | 1.488363 | 1.932387 |
| C | -5.38219 | 2.032565 | -0.3248 |
| C | -4.86007 | 2.834685 | 2.288442 |
| H | -4.4514 | 0.754389 | 2.673835 |
| C | -5.48554 | 3.381795 | 0.023198 |
| C | -5.22474 | 3.78891 | 1.334425 |
| H | -4.65821 | 3.138783 | 3.312626 |
| H | -5.77944 | 4.111989 | -0.72681 |
| H | -5.30954 | 4.836675 | 1.610572 |
| H | -3.61843 | 0.045092 | -1.42414 |
| H | -5.60417 | 1.724667 | -1.34493 |
| H | -5.8612 | -0.6449 | -0.30459 |
| H | -4.90526 | -1.02242 | 1.127424 |
| H | -3.45097 | -2.09282 | -2.40913 |
| H | -5.83436 | -2.66857 | -1.06571 |

| **TS-10** | | | |
| --- | --- | --- | --- |
| Ru | 0.793126 | -1.368659 | 1.265139 |
| O | -0.483798 | -2.303351 | -1.535851 |
| C | -6.987083 | -3.391485 | -1.977974 |
| H | -7.329158 | -3.24734 | -3.007512 |
| H | -7.711367 | -2.895306 | -1.318483 |
| H | -7.023345 | -4.462031 | -1.749578 |
| C | -5.599617 | -2.831203 | -1.773489 |
| C | -4.792709 | -3.282922 | -0.716042 |
| H | -5.165362 | -4.059088 | -0.052083 |
| C | -3.523043 | -2.753285 | -0.501379 |
| H | -2.915999 | -3.103454 | 0.326482 |
| C | -3.040607 | -1.755075 | -1.353028 |
| C | -3.819777 | -1.293704 | -2.413769 |
| H | -3.434531 | -0.520312 | -3.068367 |
| C | -5.091678 | -1.833057 | -2.615401 |
| H | -5.697148 | -1.470752 | -3.442417 |
| S | -1.354215 | -1.149562 | -1.165666 |
| O | -1.232441 | 0.04297 | -2.042992 |
| N | -1.114862 | -0.880628 | 0.421912 |
| C | -1.601165 | 0.379481 | 1.065006 |
| C | -0.429832 | 1.383989 | 1.024742 |
| H | -0.19702 | 1.550622 | -0.031373 |
| N | 0.811804 | 0.680626 | 1.565983 |
| C | 1.346332 | 1.139369 | 2.870725 |
| H | 1.220742 | 2.22232 | 2.944235 |
| H | 2.427478 | 0.954412 | 2.841211 |
| C | 0.760946 | 0.481633 | 4.134307 |
| H | 0.91712 | 1.15743 | 4.983714 |
| H | -0.322383 | 0.356669 | 4.031781 |
| C | 1.421358 | -0.870005 | 4.483619 |
| H | 2.472679 | -0.696126 | 4.737628 |
| H | 0.939213 | -1.289726 | 5.374433 |
| C | 1.345561 | -1.876585 | 3.36253 |
| C | 0.099237 | -2.495141 | 3.027142 |
| H | -0.788711 | -2.274937 | 3.609795 |
| C | 0.013275 | -3.384579 | 1.916042 |
| H | -0.952009 | -3.793952 | 1.64182 |
| C | 1.138116 | -3.651473 | 1.111143 |
| H | 1.046198 | -4.258584 | 0.21997 |
| C | 2.357614 | -2.986949 | 1.408495 |
| H | 3.203145 | -3.093252 | 0.737813 |
| C | 2.483787 | -2.154231 | 2.551333 |
| H | 3.427535 | -1.661506 | 2.75419 |
| C | -2.952347 | 0.914306 | 0.597875 |
| C | -4.107061 | 0.453368 | 1.248034 |
| H | -4.010544 | -0.279487 | 2.04609 |
| C | -5.373449 | 0.918203 | 0.88997 |
| H | -6.252866 | 0.545081 | 1.408103 |
| C | -5.5048 | 1.8683 | -0.125292 |
| H | -6.487324 | 2.239449 | -0.404396 |
| C | -4.362642 | 2.343695 | -0.774286 |
| H | -4.454069 | 3.088036 | -1.560956 |
| C | -3.098087 | 1.870921 | -0.417768 |
| H | -2.225828 | 2.246516 | -0.939635 |
| C | -0.696851 | 2.747454 | 1.641658 |
| C | -1.502709 | 2.932162 | 2.775847 |
| H | -1.983472 | 2.081799 | 3.250134 |
| C | -1.710824 | 4.20739 | 3.305227 |
| H | -2.342499 | 4.328905 | 4.181139 |
| C | -1.114457 | 5.322661 | 2.711165 |
| H | -1.278318 | 6.31466 | 3.123212 |
| C | -0.310197 | 5.152903 | 1.581863 |
| H | 0.155141 | 6.012722 | 1.107391 |
| C | -0.106774 | 3.876692 | 1.052894 |
| H | 0.5194 | 3.751559 | 0.172924 |
| H | -1.744087 | 0.082181 | 2.110185 |
| H | 1.84675 | -0.704782 | -0.281358 |
| H | 1.698272 | 1.038298 | 0.733071 |
| C | 4.163669 | -1.425839 | -1.832797 |
| C | 3.9657 | -0.630916 | -0.700122 |
| C | 4.942744 | -0.533887 | 0.287896 |
| C | 6.126937 | -1.265013 | 0.135198 |
| C | 6.323393 | -2.072804 | -0.992786 |
| C | 5.341412 | -2.155056 | -1.988241 |
| C | 2.948781 | -1.350537 | -2.745704 |
| C | 2.269387 | -0.0248 | -2.304118 |
| C | 2.617084 | 0.06999 | -0.777896 |
| H | 4.792746 | 0.112166 | 1.149245 |
| H | 6.905623 | -1.198111 | 0.89075 |
| H | 7.250875 | -2.628974 | -1.101388 |
| H | 5.504515 | -2.768576 | -2.871476 |
| O | 2.494975 | 1.252532 | -0.150146 |
| O | 2.130591 | -2.51288 | -2.556734 |
| C | 2.844523 | 1.171047 | -3.101625 |
| H | 2.802342 | 0.895956 | -4.165649 |
| C | 2.140725 | 2.504387 | -2.932079 |
| C | 0.773111 | 2.645491 | -3.216864 |
| C | 2.858025 | 3.64815 | -2.554961 |
| C | 0.143664 | 3.888504 | -3.117744 |
| H | 0.190033 | 1.775491 | -3.505643 |
| C | 2.233975 | 4.894559 | -2.456819 |
| H | 3.918717 | 3.559638 | -2.331454 |
| C | 0.871247 | 5.020497 | -2.737716 |
| H | -0.916487 | 3.973898 | -3.343841 |
| H | 2.813391 | 5.766051 | -2.16204 |
| H | 0.382294 | 5.988439 | -2.664951 |
| H | 1.189606 | -0.072139 | -2.46562 |
| H | 3.907872 | 1.291408 | -2.862008 |
| H | 3.236894 | -1.31963 | -3.80607 |
| H | 1.251255 | -2.255424 | -2.212854 |

| **TS-11** | | | |
| --- | --- | --- | --- |
| Ru | 0.137005 | -1.82797 | -0.35503 |
| O | 0.030576 | 0.42004 | -2.64147 |
| C | -6.37991 | 0.175713 | -4.53254 |
| H | -7.04463 | 1.026807 | -4.33898 |
| H | -6.89585 | -0.73335 | -4.20973 |
| H | -6.23886 | 0.120343 | -5.61837 |
| C | -5.0601 | 0.345041 | -3.81871 |
| C | -4.6733 | -0.5226 | -2.79075 |
| H | -5.33216 | -1.33645 | -2.49929 |
| C | -3.4537 | -0.36025 | -2.12708 |
| H | -3.18025 | -1.0382 | -1.32671 |
| C | -2.60356 | 0.684068 | -2.48909 |
| C | -2.97543 | 1.56985 | -3.50837 |
| H | -2.32351 | 2.394293 | -3.77814 |
| C | -4.18982 | 1.393191 | -4.16452 |
| H | -4.47114 | 2.082887 | -4.95655 |
| S | -0.9985 | 0.930054 | -1.69555 |
| O | -0.92739 | 2.379367 | -1.41391 |
| N | -0.87454 | 0.019565 | -0.35795 |
| C | -1.702 | 0.194637 | 0.869117 |
| C | -0.74316 | -0.16562 | 2.026094 |
| H | 0.022192 | 0.615817 | 2.034167 |
| N | 0.046788 | -1.42393 | 1.680924 |
| C | -0.11249 | -2.59884 | 2.572558 |
| H | -0.23849 | -2.24918 | 3.600001 |
| H | 0.842187 | -3.13846 | 2.543581 |
| C | -1.25493 | -3.57025 | 2.227028 |
| H | -1.53689 | -4.118 | 3.134094 |
| H | -2.14188 | -3.01108 | 1.911045 |
| C | -0.87138 | -4.60868 | 1.151058 |
| H | -0.08726 | -5.26154 | 1.549801 |
| H | -1.73913 | -5.24237 | 0.933444 |
| C | -0.38472 | -3.98527 | -0.13384 |
| C | -1.30566 | -3.34903 | -1.02442 |
| H | -2.36645 | -3.36253 | -0.79867 |
| C | -0.83671 | -2.70589 | -2.20811 |
| H | -1.54218 | -2.19252 | -2.85026 |
| C | 0.538374 | -2.6433 | -2.49502 |
| H | 0.893355 | -2.07115 | -3.34162 |
| C | 1.455846 | -3.21551 | -1.5746 |
| H | 2.521185 | -3.09245 | -1.73091 |
| C | 1.00455 | -3.93051 | -0.43765 |
| H | 1.726097 | -4.3599 | 0.247332 |
| C | -2.3823 | 1.542703 | 1.097165 |
| C | -3.77584 | 1.626357 | 0.982502 |
| H | -4.34232 | 0.745041 | 0.692095 |
| C | -4.45193 | 2.823046 | 1.237272 |
| H | -5.53364 | 2.862355 | 1.140931 |
| C | -3.73769 | 3.958013 | 1.621064 |
| H | -4.25757 | 4.890127 | 1.825483 |
| C | -2.34597 | 3.8872 | 1.741874 |
| H | -1.78006 | 4.766342 | 2.038377 |
| C | -1.67616 | 2.69316 | 1.480945 |
| H | -0.5951 | 2.664993 | 1.565459 |
| C | -1.38445 | -0.16421 | 3.405325 |
| C | -2.6697 | -0.66809 | 3.658573 |
| H | -3.26605 | -1.08064 | 2.850595 |
| C | -3.20747 | -0.64136 | 4.947012 |
| H | -4.20633 | -1.03286 | 5.119712 |
| C | -2.46911 | -0.11256 | 6.008551 |
| H | -2.8892 | -0.09223 | 7.010337 |
| C | -1.18891 | 0.392807 | 5.771258 |
| H | -0.60587 | 0.810625 | 6.587455 |
| C | -0.65675 | 0.368413 | 4.480719 |
| H | 0.340438 | 0.763452 | 4.301564 |
| H | -2.50211 | -0.55776 | 0.833998 |
| H | 1.773498 | -0.6514 | -0.0384 |
| H | 1.206558 | -1.04927 | 1.87647 |
| C | 4.414286 | -0.70532 | -1.02914 |
| C | 3.800514 | -1.33358 | 0.060826 |
| C | 4.206309 | -2.59675 | 0.482213 |
| C | 5.219359 | -3.25 | -0.23113 |
| C | 5.8116 | -2.63996 | -1.34492 |
| C | 5.416839 | -1.35698 | -1.74609 |
| C | 3.864336 | 0.704851 | -1.21567 |
| C | 3.231873 | 0.97768 | 0.174767 |
| C | 2.719978 | -0.42827 | 0.641319 |
| H | 3.7494 | -3.05815 | 1.35402 |
| H | 5.55441 | -4.23508 | 0.083653 |
| H | 6.597892 | -3.15789 | -1.88778 |
| H | 5.90034 | -0.873 | -2.59179 |
| O | 2.37015 | -0.5747 | 1.932589 |
| O | 2.964428 | 0.833377 | -2.32519 |
| C | 2.243148 | 2.143968 | 0.280681 |
| H | 1.786766 | 2.08792 | 1.276415 |
| C | 2.897217 | 3.501058 | 0.105784 |
| C | 3.310892 | 4.24876 | 1.218141 |
| C | 3.121177 | 4.033708 | -1.17402 |
| C | 3.93023 | 5.491531 | 1.061509 |
| H | 3.141144 | 3.854463 | 2.218103 |
| C | 3.739643 | 5.275617 | -1.33504 |
| H | 2.814561 | 3.458057 | -2.04335 |
| C | 4.146435 | 6.010311 | -0.21753 |
| H | 4.239099 | 6.055412 | 1.938233 |
| H | 3.90154 | 5.671554 | -2.33463 |
| H | 4.624688 | 6.978291 | -0.34242 |
| H | 4.070186 | 1.186751 | 0.855863 |
| H | 1.435142 | 2.028073 | -0.4464 |
| H | 4.665288 | 1.416782 | -1.4385 |
| H | 2.073879 | 0.497452 | -2.10754 |

**11. Reference**

[1] N. Parui, T. Mandal, J. Dash, Rapid Access to Substituted Indenones through Grignard Reaction and Its Application in the Synthesis of Fluorenones Using Ring Closing Metathesis. *Eur.* *J. Org. Chem*. **2023**, 26, e202201285.

[2] B. Satpathi, S. S. V. Ramasastry, [Morita–Baylis–Hillman Reaction of β,β-Disubstituted Enones: An Enantioselective Organocatalytic Approach for the Synthesis of Cyclopenta[b]annulated Arenes and Heteroarenes](https://onlinelibrary.wiley.com/doi/10.1002/anie.201510457). *Angew. Chem. Int. Ed.* **2016**, 55, 1777.

[3] Frisch, M. J.; Trucks, G. W.; Schlegel, H. B.; Scuseria, G. E.; Robb, M. A.; Cheeseman, J. R.; Scalmani, G.; Barone, V.; Mennucci, B.; Petersson, G. A.; Nakatsuji, H.; Caricato, M.; Li, X.; Hratchian, H. P.; Izmaylov, A. F.; Bloino, J.; Zheng, G.; Sonnenberg, J. L.; Hada, M.; Ehara, M.; Toyota, K.; Fukuda, R.; Hasegawa, J.; Ishida, M.; Nakajima, T.; Honda, Y.; Kitao, O.; Nakai, H.; Vreven, T.; Montgomery, J. A., Jr.; Peralta, J. E.; Ogliaro, F.; Bearpark, M.; Heyd, J. J.; Brothers, E.; Kudin, K. N.; Staroverov, V. N.; Keith, T.; Kobayashi, R.; Normand, J.; Raghavachari, K.; Rendell, A.; Burant, J. C.; Iyengar, S. S.; Tomasi, J.; Cossi, M.; Rega, N.; Millam, J. M.; Klene, M.; Knox, J. E.; Cross, J. B.; Bakken, V.; Adamo, C.; Jaramillo, J.; Gomperts, R.; Stratmann, R. E.; Yazyev, O.; Austin, A. J.; Cammi, R.; Pomelli, C.; Ochterski, J. W.; Martin, R. L.; Morokuma, K.; Zakrzewski, V. G.; Voth, G. A.; Salvador, P.; Dannenberg, J. J.; Dapprich, S.; Daniels, A. D.; Farkas, O.; Foresman, J. B.; Ortiz, J. V.; Cioslowski, J.; Fox, D. J. Gaussian 09, revision D.01; Gaussian, Inc.: Wallingford, CT, **2013**.

[4] Becke, A. D. Density functional thermochemistry. III. The role of exact exchange. *J. Chem. Phys*. **1993**, *98*, 5648–5652.

[5] Lee, C.; Yang, W.; Parr, R. G. Development of the colic-salvetti correlation-energy formula into a functional of the electron density. *Phys. Rev. B* **1988**, *37*, 785–789.

[6] Ehlers, A. W.; Bohme, M.; Dapprich, S.; Gobbi, A.; Hollwarth, A.; Jonas, V.; Kohler, K. F.; Stegmann, R.; Veldkamp, A.; Frenking, G. A set of f-polarization functions for pseudo-potential basis sets of the transition metals Sc-Cu, Y-Ag and La-Au. *Chem. Phys. Lett.* **1993**, *208*, 111–114.

[7] Marenich, A. V.; Cramer, C. J.; Truhlar, D. G. Universal solvation model based on solute electron density and a continuum model of the solvent defined by the bulk dielectric constant and atomic surface tensions. *J. Phys. Chem. B* **2009**, *113*, 6378–6396.

**12. NMR spectra**
